# Contribution of alcohol use to the global burden of cirrhosis and liver cancer from 1990 to 2019 and projections to 2044

**DOI:** 10.1007/s12072-023-10503-2

**Published:** 2023-03-05

**Authors:** Yang Liu, Zhouyi Sun, Qianwen Wang, Kangze Wu, Zhe Tang, Bo Zhang

**Affiliations:** 1 The Fourth Affiliated Hospital, Zhejiang University School of Medicine, Yiwu, 322000 Zhejiang China; 2 The Second Affiliated Hospital, Zhejiang University School of Medicine, 88 Jiefang Road, Hangzhou, 310000 Zhejiang Province China

**Keywords:** Alcohol use, Cirrhosis, Liver cancer, Socioeconomics, Epidemiology, Public health, Global burden of disease, Liver disease, Age-standardized rate, Disability-adjusted life-years

## Abstract

**Background:**

Identifying the temporal trends of cirrhosis and liver cancer attributable to alcohol use in both the past and the future can formulate the control strategies.

**Methods:**

Data on cirrhosis and liver cancer attributable to alcohol use from 1990 to 2019, including mortality and disability-adjusted life year (DALY) rates were collected from the 2019 Global Burden of Disease (GBD) study. To analyze the temporal trends, the average annual percentage change (AAPC) was calculated, and the Bayesian age-period-cohort model was applied.

**Result:**

Deaths and DALY of cirrhosis and liver cancer attributable to alcohol use increased year by years, but the age-standardized death rate (ASDR) and age-standardized DALY rate declined or remained stable from 1990 to 2019 in most regions of the world. The burden of cirrhosis attributable to alcohol increased in low-middle social-development index (SDI) regions, while the burden of liver cancer increased in high-SDI regions. Eastern Europe and Central Asia have the highest burden of cirrhosis and liver cancer caused by alcohol use. Deaths and DALYs are mainly distributed in people aged 40+ years, but there is an increasing trend in people aged under 40 years. New deaths from cirrhosis and liver cancer attributable to alcohol use are predicted to increase in the next 25 years, but the ASDR of cirrhosis in males will increase slightly.

**Conclusions:**

Although the age-standardized rate of cirrhosis and liver cancer due to alcohol use have decreased, the absolute burden increased and will continue to increase. Therefore, alcohol control measures should be further strengthened and improved through effective national policies.

**Supplementary Information:**

The online version contains supplementary material available at 10.1007/s12072-023-10503-2.

## Introduction

Alcohol use has long been considered as a behavior for purpose of entertainment [1]. Although moderate alcohol use may play a protective role on our body, unhealthy alcohol use leads to the development or deteriorating of numerous medical conditions, such as cardiovascular disease, digestive disease, neurological disease, and various types of cancer, with a clear dose–response relationship [2]. In 2019, alcohol use was the second leading risk factor attributed to cancer deaths and disability-adjusted life-years (DALYs) [3]. Despite a series of nationally and global campaigns to reduce alcohol use, the global pattern in alcohol consumption still shows an increasing trend, and will continue to grow, causing enormous global burden [4].

Liver cirrhosis is the common consequence of various chronic liver diseases, including degeneration and necrosis of hepatocytes, hyperplasia of fibrous tissue, destruction of hepatic lobules, and formation of pseudolobuli [5]. According to the global burden of disease study report, cirrhosis caused more than 1.32 million deaths in 2017 and ranked 16th among the leading cause of DALYs in 2019 [6]. Alcohol, a leading risk factor in the progression of liver cirrhosis, largely affected mortality and morbidity of liver cirrhosis [7]. The International Classification of Diseases recognizes a series of alcohol-related liver diseases, such as alcoholic hepatitis and alcoholic liver cirrhosis. In 2017, there were more than 2 million cases of cirrhosis linked to alcohol use in the US. The main treatments for alcohol-related cirrhosis are corticosteroid therapy and liver transplantation [8]. In recent years, the number of patients with alcohol-related cirrhosis waiting for liver transplants has increased worldwide [9].

Liver cancer remains a public health concern around the world. Approximately, 906,000 newly diagnosed liver cancer cases and 830,000 deaths globally were recorded in 2020 [10]. There are many risk factors for liver cancer, of which HBV and HCV are still the most important. However, liver cancer is usually the result of multiple factors in the context of chronic liver disease [11]. Alcohol use promotes the development of liver cancer by acting independently or synergistically with other risk factors. For example, alcohol use increases risk of liver cancer among HBV carriers and persons with active HCV infections [12]. In 2017, a total of 129,287 deaths related to liver cancer could be attributed to alcohol use [11]. Liver cancer caused by alcohol has direct costs to the health care system in terms of medical treatment and indirect costs to social development attributable to loss of labor productivity.

Alcohol consumption has been shown to vary by age and location [13], so liver cirrhosis and liver cancer burden caused by alcohol use must have its unique age and regional pattern. However, few studies describe trends in the global burden of cirrhosis and liver cancer ascribe to alcohol use and relation to socio-demographic index (SDI). And alcohol public health policies have been shown to reduce the prevalence and mortality of alcohol-associated liver disease [14]. Therefore, we used the data from the Global Burden of Disease (GBD) to analyse the global pattern of liver cirrhosis and liver cancer attributable to alcohol use from 1990 to 2019 in 204 countries and territories and their associations with socioeconomic status, to provide a more comprehensive perspective to make global and regional targeted intervention and health policies for alcohol consumption control.

## Methods

### Study data

The liver cirrhosis and liver cancer burden data attributable to alcohol were downloaded from the GBD Results Tool (http://ghdx.healthdata.org/gbd-results-tool), which fully estimated the burden of 369 diseases and injuries alongside attributable 87 risk factors by gender, age, region, and country. Based on the socio-demographic index (SDI), 204 countries and territories can be divided into 5 regions, including low, low-middle, middle, high-middle, and high. And these countries and territories can be separated into 21 regions considering geological congruity, such as Western Pacific and Central Europe. The data results were presented as numbers with 95% uncertainty intervals (UIs).

### SDI

A composite indicator of development status with a high correlation to health outcomes is called the SDI, which was calculated using several social factors, including the fertility rate of the population aged < 25 years, the education level of the population aged > 15 years, and per capita income. Countries and territories with SDI values of 0 and 1 represent the potential minimum and maximum levels of development status relevant to health outcomes, respectively.

### Estimation of alcohol use exposure

Alcohol exposure was defined as the grams of pure alcohol per day among individuals who have drunk at least one alcoholic beverage in the past years. The assessment methods of alcohol use have been detailed on the following website: http://ghdx.healthdata.org/gbd-2019/code/risk-1. To quantify the proportion of the disease-specific burden caused by each risk factor, the comparative risk assessment approach was incorporated into the GBD study [15]. The framework offers synthesizing evidence on risks and risk-outcome pairs and typically consists of six key steps. First, identification of convincing risk-outcome pairs was done using the World Cancer Research Fund criteria. Second, calculate the summary relative risk based on quantitative systematic reviews, and relax the log-linear assumption for relevant risk factors using meta-regression Bayesian, regularized, trimmed (MRBRT). Third, to estimate risk factor exposure levels and distributions across the world, data from different sources were aggregated and standardized using a Bayesian meta-regression model (DisMod-MR2.1) and a spatio-temporal Gaussian process regression model (ST-GPR) [16]. Fourth, the theoretical minimum-risk exposure level (TMREL) was defined for each risk factor. Fifth, calculate the population attributable fraction for each risk-outcome pair. Sixth, considering possible mediation of some risk factors through other risk factors, the mediation matrix was used to compute the burden attributable to various combinations of risk factors.

### Statistical analysis

Mortality, DALYs and the corresponding age-standardized rate (ASR) were used to assess trends in alcohol-related cirrhosis and liver cancer. By adding the years of life (YLDs) with disabilities and years of life lost (YLLs), the DALYs is calculated [17]. When comparing morbidity, mortality, and DALYs, ASR is an important indicator that effectively excludes the influence of age factors between regions or countries with different age structures. The ASRs were calculated using the following formula [18], $\mathrm{ASR}=\frac{\sum_{i=1}^{A}{a}_{i}{w}_{i}}{\sum_{i=1}^{A}{w}_{i}}\times \mathrm{100,000}$ (*a*_*i*_ represents the specific age ratio of the *i*th age group, *w*_*i*_ represents the number (or weight) of the corresponding age group in the selected reference standard population, and *A* represents the number of age groups). Each rate was reported per 100,000 population and the corresponding 95% uncertainty interval (UI) was provided in our study. Moreover, we used the average annual percentage change (AAPC) to quantify temporal trend of the age-standardized death/DALY rates (ASRs) for alcohol-related cirrhosis and liver cancer in 1990–2019. The AAPCs, which could identify the continuous changes of disease data over the complete study period, were evaluated by Joinpoint regression analysis. When the AAPC value and its 95% CI > 0, the trend is defined as increasing. Conversely, when the AAPC value and its 95% CI < 0, the trend is decreasing. Otherwise, the burden is considered relatively stable over time. Joinpoint regression analysis was performed by the Joinpoint Regression Program v4.9.1.0 provided by the United States National Cancer Institute Surveillance Research Program. To identify the influencing factors of cirrhosis and liver cancer burden attributable to alcohol, the Pearson’s correlation analysis was used to analyze the association of AAPCs, ASRs and SDI (2019), respectively. The detailed analysis process can be found in the previous article [19].

### The burden prediction

We used the Bayesian age-period-cohort (BAPC) model integrated nested Laplace approximations, which was shown to have a better accuracy than other prediction model, to predict the future burden pattern of liver cirrhosis and liver cancer attributable to alcohol use [20–22]. The BAPC model is based on the age-period-cohort (APC) model, which assumes an association between morbidity or mortality and age structure and population size. In short, the APC model can be understood through the log-linear Poisson model: ${n}_{ij}=\mathrm{log}\left({\lambda }_{ij}\right)=\mu +{\alpha }_{i}+{\beta }_{j}+{\gamma }_{k}+\varepsilon$. In the model, μ represents intercept; ε represents random error; α, β and γ represent the age, period, and cohort, respectively; *i*, *j*, and *k* represent the coefficients of age, period, and cohort effects. Based on the assumption that the effects of age, period, and cohort adjacent in time might be similar, the BAPC model applied a second-order random walk with inverse-gamma prior distribution for age, period, and cohort effect. To approximate marginal posterior distributions, BAPC model uses an integrated layered Laplacian approximation. In addition, to better present the prediction results, we set the baseline reference, negative reference, and positive reference. Baseline reference is based on mortality in 2019; negative reference is a 1% annual increase in mortality in 2019; positive reference is a 1% annual decrease in mortality in 2019. We calculated the absolute number of deaths based on the changes in mortality. The BAPC model integrated nested Laplace approximations was performed by R package BAPC and INLA-based R (version 4.4.1).

## Result

### Temporal trends of burden of cirrhosis and liver cancer attributable to alcohol use

**Fig. 1:**
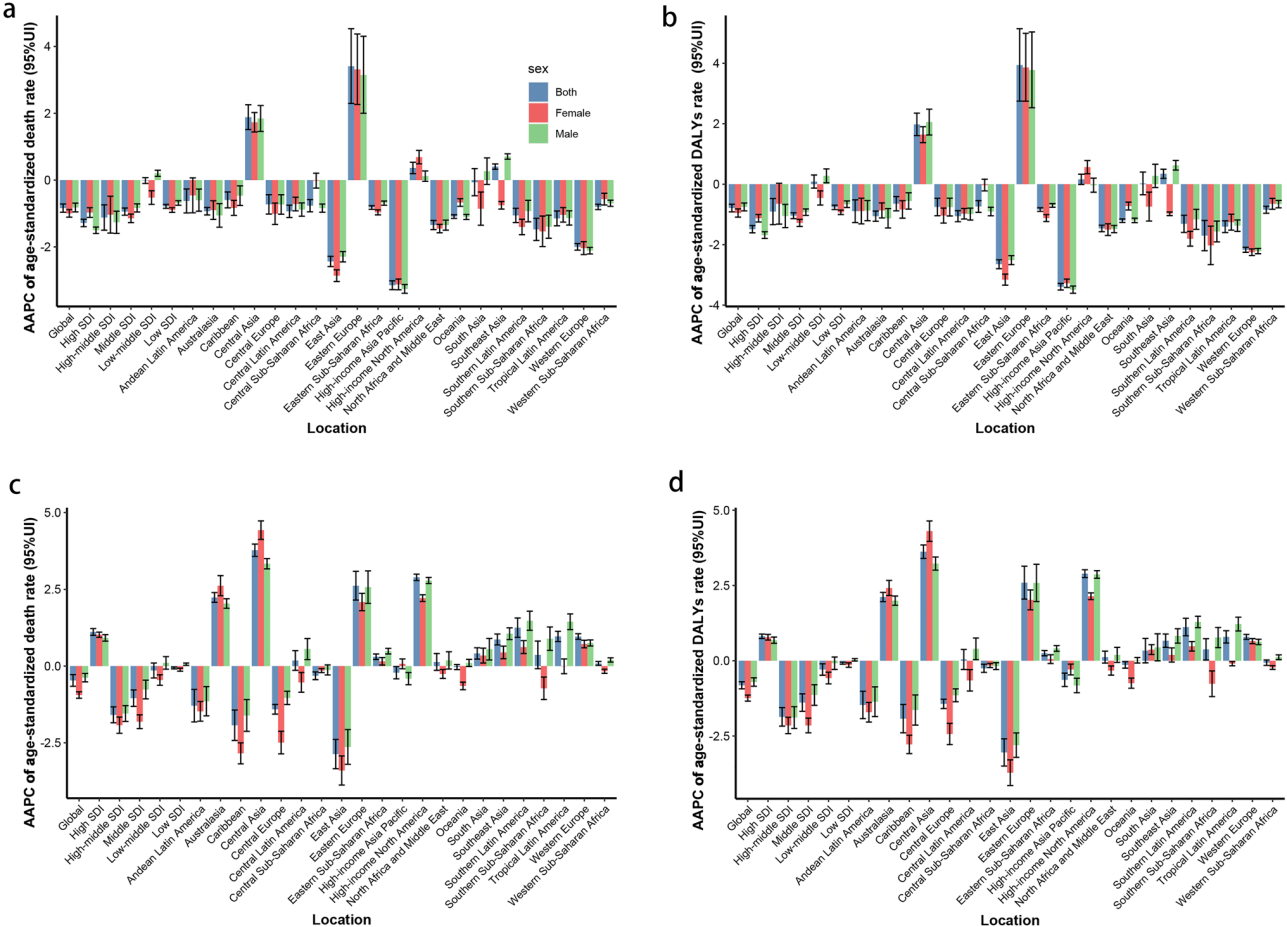
The AAPC of ASR burden for cirrhosis and liver cancer attributable to alcohol use from 1990 to 2019 in global, SDI regions, and 21 GBD regions by sex. **A** AAPC of ASDR in cirrhosis; **B** AAPC of age-standardized DALY rate in cirrhosis; **C** AAPC of ASDR in liver cancer; **D** AAPC of age-standardized DALY rate in liver cancer. *AAPC* average annual percentage change, *ASR* age-standardized rate; *GBD* Global Burden of Diseases, Injuries, and Risk Factors Study; *SDI* socio-demographic index; *ASDR* age-standardized death rate

**Fig. 2:**
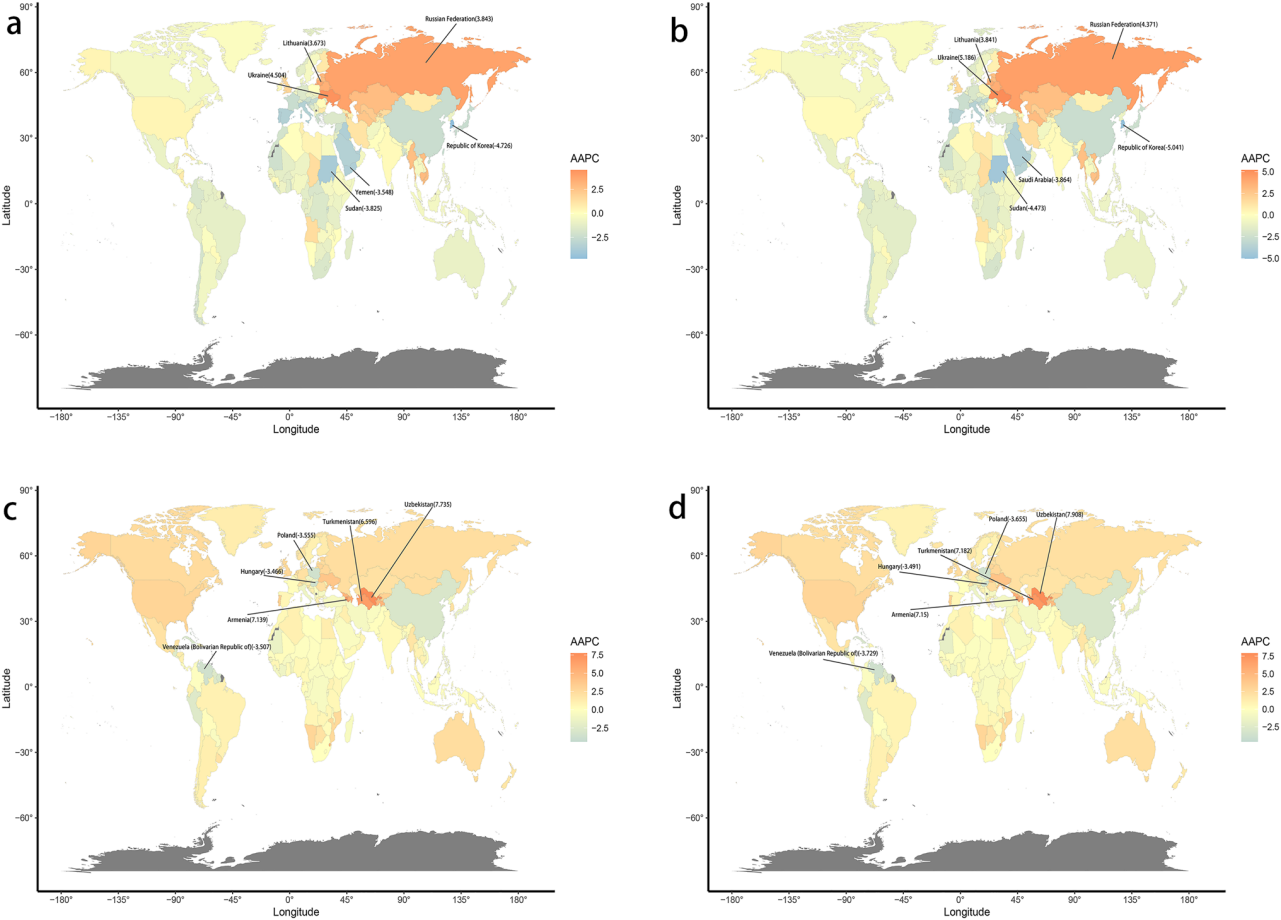
The global disease burden of cirrhosis and liver cancer attributable to alcohol consumption for both genders in 204 countries and territories from 1990 to 2019. **A** AAPC of ASDR for cirrhosis; **B** AAPC of age-standardized DALY rate for cirrhosis; **C** AAPC of ASDR for liver cancer **D** AAPC of age-standardized DALY rate for liver cancer. *AAPC* average annual percentage change; *DALYs* disability-adjusted life-years; *ASDR* age-standardized death rate

In 2019, there were 0.46 million (95% UI, 0.37–0.55) cirrhosis deaths attributed to alcohol use, accounting for 55.98% of the total number of cirrhosis deaths. The corresponding age-standardized death rate (ASDR) decreased by 0.84% (95% UI, −0.96 to −0.72) per year from 1990 to 2019 (Table 1). In the same years, there are 22.8 million (95% UI, 17.9–27.9) DALYs globally from cirrhosis attributable to alcohol use. Meanwhile, the age-standardized DALY rate decreased with an AAPC of −0.79%. Regarding the SDI regions, the low SDI region had the highest ASDR and age-standardized DALY rate of cirrhosis. Fortunately, the cirrhosis burden ascribed to alcohol use in all SDI regions showed significant downward trends, except for the low-middle SDI region (Fig. 1A, B). Regionally, the top three regions with the highest ASR burden are Central Asia, Eastern Sub-Saharan Africa, and Western Sub-Saharan Africa (Figure S1 a, b). The largest increases in ASR of cirrhosis attributable to alcohol use were Eastern Europe and Central Asia, while the largest decreases were found in High-income Asia Pacific an East Asia (Table S1). Meanwhile, the three countries with the highest increase in the burden of cirrhosis attributable to alcohol use are Ukraine, Russian Federation, and Lithuania, all located in Eastern Europe (Fig. 2A, B). The cirrhosis burden in terms of the ASDR and age-standardized DALY rates in Mongolia, Cambodia, and Turkmenistan were among the top three in the world (Table S2). More details were shown in Additional file 1: Table S5–S6.

**Table 1:** The number, age-standardized rate, and temporal trend of cirrhosis burden attributable to alcohol use from 1990 to 2019

	Death					DALYs				
	1990		2019		Average annual percentage change (95% CI), 1990–2019	1990		2019		Average annual percentage change (95% CI), 1990–2019
	Case (10^3^)	Age-standardized death rate (per 100 000 person-years)	Case (10^3^)	Age-standardized death rate (per 100 000 person-years)		Count (10^3^)	Age-standardized DALY rate (per 100 000 person-years)	Count (10^3^)	Age-standardized DALY rate (per 100 000 person-years)	
Overall	457,058.1	11	712,815.4	8.62	− 0.84 (− 0.963 to − 0.717)	15,137,520	343.09	22,767,018.7	272.75	− 0.789 (− 0.915 to − 0.664)
Sex										
Female	97,448	4.58	147,622.4	3.4	− 0.997 (− 1.111 to − 0.883)	2,811,896.7	127.07	4,083,502.2	95.38	− 0.954 (− 1.09 to − 0.818)
Male	359,610.1	18.06	565,193	14.24	− 0.822 (− 0.953 to − 0.69)	12,325,623	566.14	18,683,516.5	455.63	− 0.753 (− 0.884 to − 0.622)
Socio-demographic index										
High SDI	105,155.3	10.65	123,365.4	7.32	− 1.285 (− 1.397 to − 1.173)	3,183,588.8	339.57	3,273,548.6	259.49	− 1.488 (− 1.609 to − 1.367)
High–middle SDI	120,406.2	11.05	157,028	7.95	− 1.122 (− 1.502 to − 0.741)	3,832,729.6	332.83	4,982,809	215.8	− 0.908 (− 1.339 to − 0.475)
Middle SDI	116,549.3	10.2	199,389	7.77	− 0.943 (− 1.051 to − 0.836)	4,118,488.8	322.28	6,410,232.7	237.98	− 1.043 (− 1.14 to − 0.947)
Low–middle SDI	74,140.9	10.96	161,272.8	10.89	− 0.014 (− 0.101 to 0.073)	2,641,835.3	348.41	5,646,597.5	355.15	0.087 (− 0.13 to 0.305)
Low SDI	40,569.5	15.68	71,376.6	12.41	− 0.787 (− 0.846 to − 0.729)	1,353,298.2	459.54	2,441,955.7	366.02	− 0.766 (− 0.839 to − 0.694)
Region										
Andean Latin America	3292.4	14.79	6965.4	12.23	− 0.625 (− 0.984 to − 0.266)	109,583.7	449.38	201,861.1	342.24	− 0.893 (− 1.269 to − 0.517)
Australasia	1225.3	5.42	1829.9	4.04	− 0.933 (− 1.044 to − 0.822)	35,709	159.9	47,628.8	115.86	− 1.059 (− 1.228 to − 0.89)
Caribbean	3347.7	12.46	5379.3	10.39	− 0.594 (− 0.832 to − 0.356)	108,069.8	385.43	163,521.3	317.13	− 0.646 (− 0.882 to − 0.409)
Central Asia	6538.2	13.23	18,508.5	22.49	1.883 (1.512–2.256)	215,753	410.68	652,788	719.95	1.978 (1.607–2.351)
Central Europe	23,305.3	15.88	24,266.6	12.89	− 0.727 (− 1.018 to − 0.436)	734,194.8	507.38	711,295.4	409.57	− 0.755 (− 1.044 to − 0.465)
Central Latin America	19,722.4	20.79	39,172.1	16.06	− 0.937 (− 1.121 to − 0.752)	702,493.7	667.42	1,241,334.1	494.01	− 1.059 (− 1.241 to − 0.877)
Central Sub-Saharan Africa	5061	19.82	9834	15.86	− 0.767 (− 0.95 to − 0.584)	174,122.4	586.91	350,719.7	473.91	− 0.738 (− 0.933 to − 0.543)
East Asia	92,283.7	9.63	98,208.5	4.75	− 2.436 (− 2.59 to − 2.282)	3,201,819.3	305.94	2,957,123.5	141.33	− 2.648 (− 2.796 to − 2.5)
Eastern Europe	18,463.8	6.67	50,439.7	17.04	3.408 (2.294–4.534)	603,741	222.87	1,843,558.9	662.39	3.939 (2.748–5.144)
Eastern Sub-Saharan Africa	19,733.2	24.66	34,614.6	19.46	− 0.82 (− 0.875 to − 0.766)	635,027.1	687.48	1,145,722.3	539.6	− 0.84 (− 0.9 to − 0.78)
High-income Asia Pacific	28,253.7	13.96	21,787.5	5.55	− 3.149 (− 3.281 to − 3.017)	885,901.8	428.78	512,956.5	158.73	− 3.392 (− 3.501 to − 3.283)
High-income North America	25,809.5	7.88	49,098.9	8.68	0.363 (0.193–0.534)	793,078.5	251.33	1,371,291	262.82	0.161 (− 0.008–0.33)
North Africa and Middle East	7003.5	3.97	11,797.7	2.69	− 1.346 (− 1.484 to − 1.208)	214,658.4	107.04	344,755.3	69.63	− 1.466 (− 1.573 to − 1.359)
Oceania	203.6	5.2	349.6	3.8	− 1.093 (− 1.148 to − 1.038)	8226.4	186.91	13,804.6	132.58	− 1.208 (− 1.266 to − 1.15)
South Asia	59,258.9	8.81	134,688	8.74	− 0.063 (− 0.469–0.344)	2,223,177	290.61	4,888,192.4	293.55	0.025 (− 0.349–0.401)
Southeast Asia	28,429.2	9.84	71,892.7	10.97	0.405 (0.325–0.484)	999,076.5	309.92	2,408,246.6	340.77	0.344 (0.193–0.495)
Southern Latin America	7964.9	17.18	10,205.3	12.53	− 1.059 (− 1.272 to − 0.845)	241,043.9	513.95	274,951.3	347.86	− 1.312 (− 1.597 to − 1.026)
Southern Sub-Saharan Africa	4385.1	14.04	5602.5	9.1	− 1.477 (− 1.809 to − 1.144)	160,412.1	456.12	192,002.9	281.12	− 1.702 (− 2.202 to − 1.199)
Tropical Latin America	13,998.3	13.11	23,289.3	9.31	− 1.148 (− 1.369 to − 0.926)	531,827.5	451.42	760,191.7	298.81	− 1.406 (− 1.6 to − 1.211)
Western Europe	66,668.1	12.51	56,347.6	7.01	− 2.001 (− 2.099 to − 1.902)	1,847,330.3	371.15	1,386,565.3	198.43	− 2.171 (− 2.254 to − 2.089)
Western Sub-Saharan Africa	22,110.4	23.56	38,537.8	18.58	− 0.804 (− 0.884 to − 0.723)	712,273.7	672.55	1,298,508.1	523.76	− 0.847 (− 0.962 to − 0.731)

*DALYs* disability-adjusted life-years

Globally, the number of liver cancer deaths and DALYs attributable to alcohol use continued to increase from 1990 to 2019 (Table 2). The ASDR and standardized DALY rates for liver cancer due to alcohol use showed a decreasing trend with AAPC of −0.47 (95% CI: −0.66, −0.28) and −0.82 (95% CI: −0.93, −0.70), respectively. The burden of liver cancer attributable to alcohol use increased in high-SDI regions, decreased in high-middle SDI and middle SDI regions, and remained stable in low-middle SDI and low SDI regions (Fig. 1C, D). At present, the three regions with the heaviest burden of liver cancer attributable to alcohol use were Central Asia, Australasia, and Southeast Asia (Figure S1 C, D). And Central Asia and Eastern Europe also have the largest burden growth. For 204 countries and territories, Turkmenistan, Armenia, and Uzbekistan, all located in Central Asia, were the three countries with highest increase in the burden of liver cancer related to alcohol use, while Poland, Venezuela (Bolivarian Republic of), and Hungary were the 3 countries with the least (Fig. 2C, D). Mongolia, Gambia, and Thailand were three countries with the highest liver cancer burden attributable to alcohol use **(**Table S4). And the details were listed in Additional file 1: Table S7–S8.

**Table 2:** The number, age-standardized rate, and temporal trend of liver cancer burden attributable to alcohol use from 1990 to 2019

	Death					DALYs				
	1990		2019		Average annual percentage change (95% CI), 1990–2019	1990		2019		Average annual percentage change (95% CI), 1990–2019
	Case (10^3^)	Age-standardized death rate (per 100 000 person-years)	Case (10^3^)	Age-standardized death rate (per 100 000 person-years)		Count (10^3^)	Age-standardized DALY rate (per 100 000 person-years)	Count (10^3^)	Age-standardized DALY rate (per 100 000 person-years)	
Overall	54,066	1.34	96,052.6	1.17	− 0.467 (− 0.657 to − 0.276)	1,524,731	36.04	2,379,072	28.43	− 0.815 (− 0.934 to − 0.695)
Sex										
Female	12,241.5	0.57	19,231.8	0.44	− 0.942 (− 1.047 to − 0.836)	312,956.6	14.41	438,597.2	10.06	− 1.241 (− 1.342 to − 1.139)
Male	41,824.6	2.23	76,820.8	2.01	− 0.377 (− 0.513 to − 0.241)	1,211,775	59.48	1,940,475	48.37	− 0.706 (− 0.846 to − 0.565)
Socio-demographic index										
High SDI	11,354.8	1.09	27,972	1.5	1.111 (1–1.223)	279,116.1	27.8	606,895.8	35.28	0.813 (0.739–0.887)
High–middle SDI	16,231.6	1.5	19,514.9	0.95	− 1.588 (− 1.846 to − 1.328)	453,842.3	40.67	483,137.9	23.83	− 1.864 (-2.172 to − 1.555)
Middle SDI	19,050	1.76	32,426.1	1.29	− 1.045 (− 1.313 to − 0.777)	583,649.2	49.25	866,662	32.64	− 1.389 (− 1.681 to − 1.097)
Low–middle SDI	5459.8	0.91	11,923.4	0.88	− 0.143 (− 0.386–0.1)	155,156	23.35	309,938.4	21.48	− 0.291 (− 0.481 to − 0.101)
Low SDI	1939.5	0.86	4163.8	0.84	− 0.061 (− 0.092 to − 0.03)	52,230	20.53	111,202.7	19.96	− 0.082 (− 0.113 to − 0.051)
Region										
Andean Latin America	314.7	1.61	594.2	1.09	− 1.291 (− 1.82 to − 0.759)	7579.3	36.22	12,839.1	22.91	− 1.469 (-1.923 to − 1.013)
Australasia	197.1	0.83	765.5	1.59	2.237 (2.078–2.396)	4846.2	20.9	17,287.5	38.37	2.117 (1.963–2.272)
Caribbean	535.6	2.06	594.7	1.15	− 1.928 (− 2.422 to − 1.431)	12,592.7	47.5	13,760.4	26.58	− 1.924 (− 2.394 to − 1.451)
Central Asia	404	0.88	1864.2	2.6	3.775 (3.574–3.975)	10,714.6	22.3	50,593.5	63.03	3.622 (3.399–3.846)
Central Europe	3159.4	2.13	3092.7	1.43	− 1.406 (− 1.563 to − 1.248)	75,262.2	50.06	68,144.6	33.35	− 1.432 (− 1.588 to − 1.276)
Central Latin America	861.3	1.06	2587.7	1.12	0.181 (− 0.139–0.502)	21,931	25.05	60,116.3	25.2	0.042 (− 0.29–0.375)
Central Sub-Saharan Africa	85.2	0.39	184.8	0.36	− 0.315 (− 0.44 to − 0.19)	2410.1	9.5	5379.7	8.83	− 0.251 (− 0.386 to − 0.115)
East Asia	23,643.4	2.49	22,640.1	1.07	− 2.867 (− 3.342 to − 2.39)	754,030.6	74.28	646,687.5	30.24	− 3.046 (− 3.497 to − 2.593)
Eastern Europe	1416.3	0.5	3642.7	1.06	2.619 (2.151–3.088)	36,139.7	12.69	88,262.7	26.62	2.593 (2.049–3.141)
Eastern Sub-Saharan Africa	589.5	0.82	1381.6	0.89	0.305 (0.215–0.395)	15,783.8	19.59	37,087.6	21.08	0.251 (0.164–0.339)
High-income Asia Pacific	3441.1	1.67	6737.4	1.55	− 0.215 (− 0.414 to − 0.015)	93,054.4	44.34	137,540.9	36.68	− 0.633 (-0.855 to − 0.411)
High-income North America	2227.6	0.64	8874.8	1.44	2.892 (2.788–2.996)	52,843.9	15.84	209,827.5	35.72	2.893 (2.764–3.022)
North Africa and Middle East	969.1	0.58	2572	0.61	0.134 (− 0.14–0.408)	25,551.7	13.97	67,567.5	14.63	0.12 (− 0.077–0.318)
Oceania	13.2	0.47	30.2	0.47	–0.033 (− 0.114–0.049)	368.8	11.3	823.3	10.9	− 0.139 (− 0.248 to − 0.03)
South Asia	3593	0.66	10,077.1	0.73	0.411 (0.228–0.596)	98,386.4	16	257,846.7	17.42	0.336 (− 0.069–0.742)
Southeast Asia	3656.1	1.45	11,223.5	1.9	0.867 (0.69–1.045)	102,078.1	36.39	286,101.6	44.49	0.667 (0.447–0.888)
Southern Latin America	251.4	0.54	652.2	0.77	1.252 (0.937–1.567)	5899.6	12.52	14,231.9	17.27	1.12 (0.829–1.412)
Southern Sub-Saharan Africa	367.7	1.32	802.2	1.41	0.364 (− 0.085–0.815)	10,640.9	34.58	22,895.6	36.56	0.378 (0.024–0.734)
Tropical Latin America	517.9	0.58	1822.3	0.76	0.966 (0.8–1.132)	13,695.7	13.98	43,701.3	17.65	0.793 (0.589–0.998)
Western Europe	6941.4	1.2	14,057.7	1.58	0.962 (0.871–1.053)	157,779.5	28.56	289,770.7	36.02	0.792 (0.709–0.875)
Western Sub-Saharan Africa	880.9	1.04	1855	1.06	0.087 (0.024–0.15)	23,142	24.7	48,606.4	24.24	− 0.065 (− 0.155–0.025)

*DALYs* disability-adjusted life-years

### Age composition of the burden of cirrhosis and liver cancer attributable to alcohol use

**Fig. 3:**
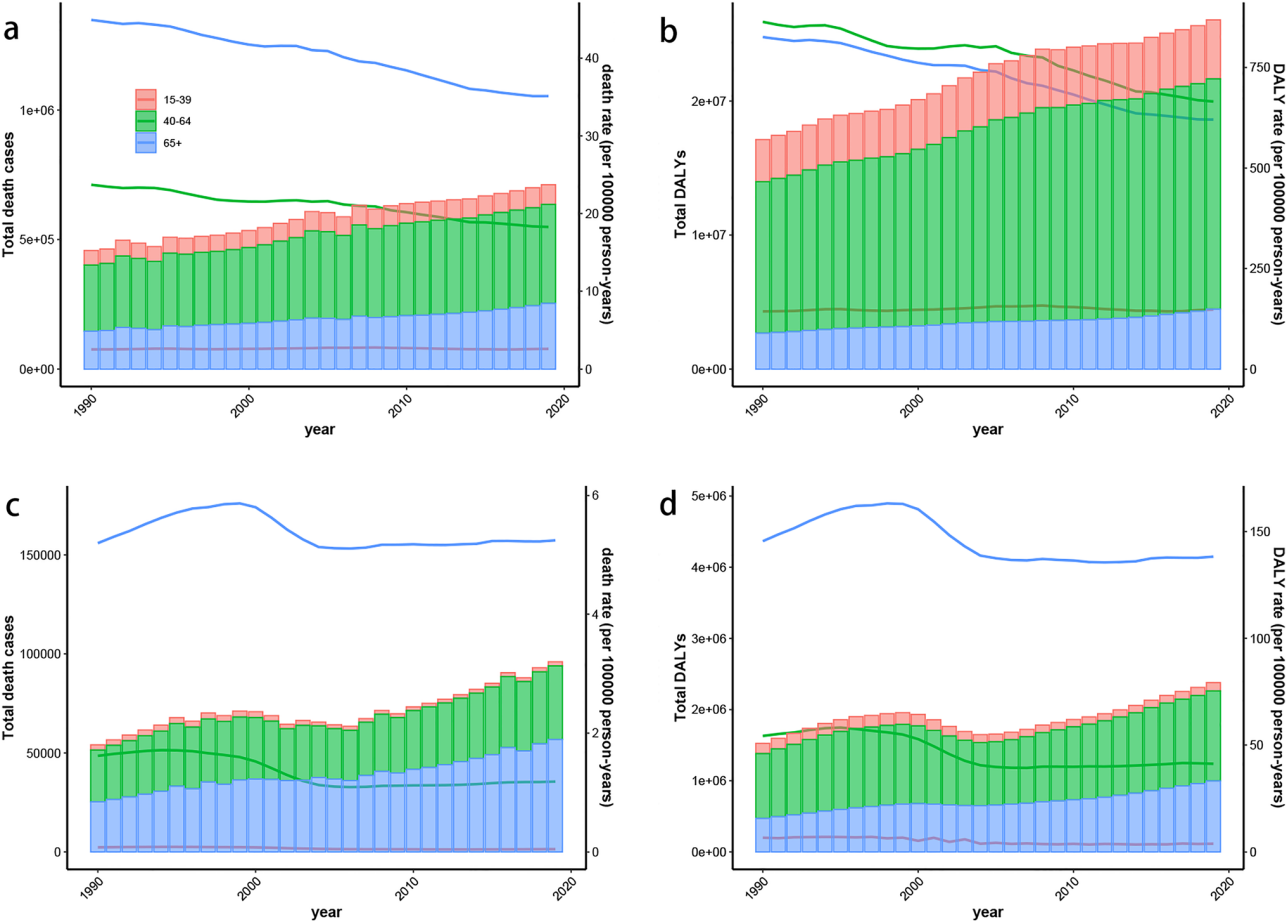
The burden of cirrhosis and liver cancer attributable to alcohol use for both genders in global by age group from 1990 to 2019. **A** Global number of death cases and death rate of cirrhosis; **B** Global number of DALYs and DALY rate of cirrhosis; **C** Global number of death cases and death rate of liver cancer; **D** Global number of DALYs and DALY rate of liver cancer. Each lines represent death/DALY rates for age group, and each bars represent the number of deaths/DALYs. *DALYs* disability-adjusted life-years

Simply, the populations were divided into three age groups: 15–39 years, 40–64 years and 65 + years. Globally, the number of cirrhosis-related deaths and DALYs ascribed to alcohol use was increased over time in all ages globally and was highest in the 40–64 age group, accounting for more than 50% of all (Fig. 3A, B). And the mortality of alcohol-associated cirrhosis was highest in 65 + age group, whereas the DALYs rate was highest in 40–64 age group, both have declined from 1990 to 2019. Only in 15–39 age group, the burden of cirrhosis attributable to alcohol use has remained stable. In 2019, the number of cirrhosis deaths and DALYs attributable to alcohol in the 40–64 age group was highest in most GBD regions. (Figure S2 A, B). The Eastern Europe and Central Asia showed a completely different age composition of burden growth than other regions, in which 15–39 age group plays a key role (Figure S3 A, B).

In 2019, the 65 + age group had the highest death cases of liver cancer attributable to alcohol use in 2019 and the 40–64 age group had the highest number of DALYs (Fig. 3C, D). In addition, the mortality and DALYS of liver cancer attributable to alcohol use increased significantly with age, peaking in the 65 + age group. However, in East Asia, Central Sub-Saharan Africa and Central Asia, the number of deaths and DALYs were the highest in the 40–64 age group (Figure S2 C, D). The proportion of elderly liver cancer deaths and DALYs attributable to alcohol use was the largest in the high-SDI region. The highest increase in burden in 15–39 age group was found in Eastern Europe and Central Asia (Figure S3 C, D).

### Sex disparity of the burden of cirrhosis and liver cancer attributable to alcohol use

**Fig. 4:**
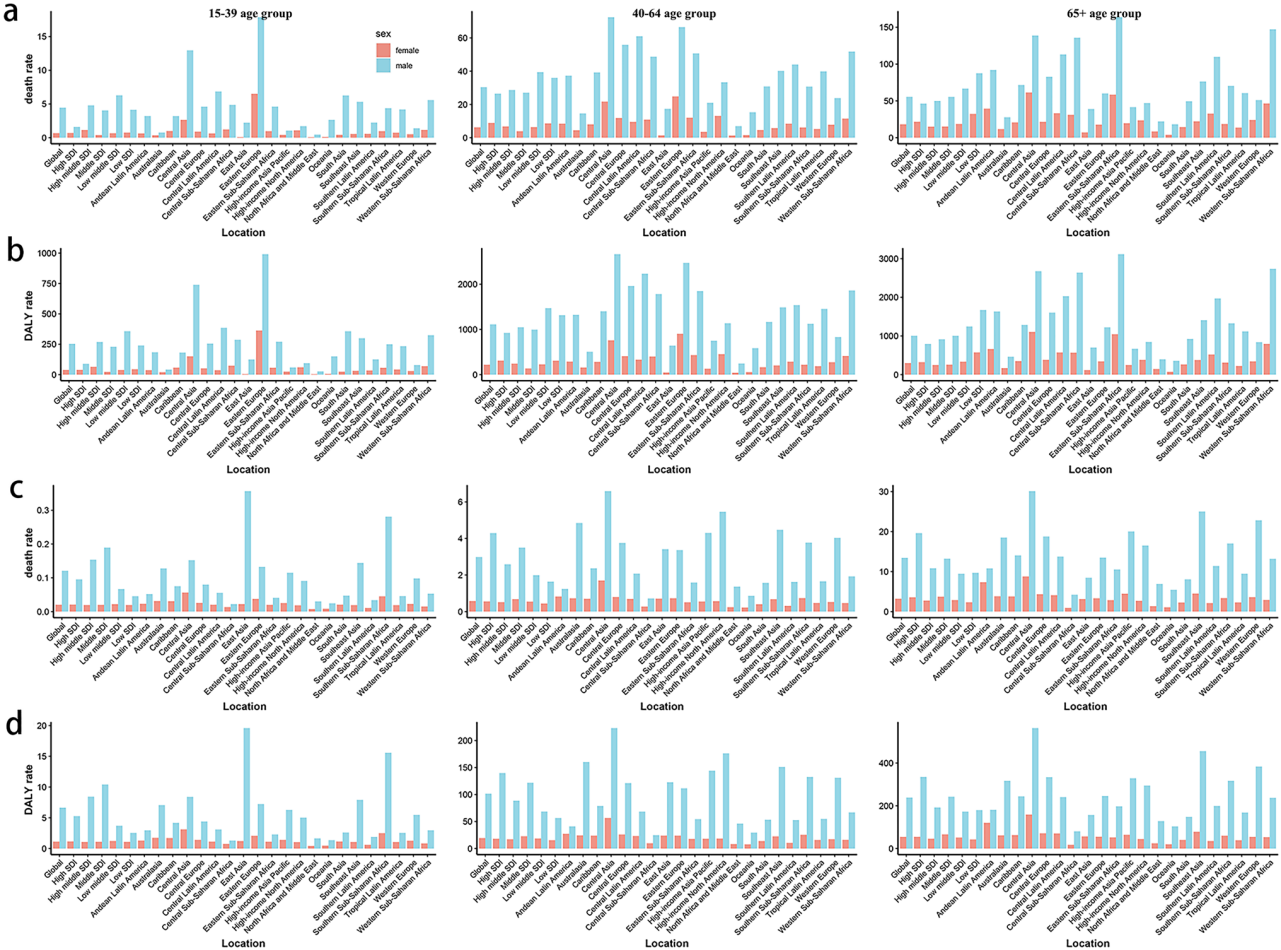
The death and DALY rate of cirrhosis and liver cancer attributable to alcohol use in global, SDI regions, and 21 GBD regions in 2019 by age group and sex. **A** The death rate of cirrhosis attributable to alcohol use; **B** The DALY rate of cirrhosis attributable to alcohol use; **C** The death rate of liver cancer attributable to alcohol use; **D** The DALY rate of liver cancer attributable to alcohol use. The first column represents the 15–39 age group; the second column represents the 40–64 age group; the third column represents the 65 + age group. *SDI* socio-demographic index; *GBD* Global Burden of Diseases, Injuries, and Risk Factors Study; *DALYs* disability-adjusted life-years

There are significant differences between male and female regarding the cirrhosis and liver cancer burden related to alcohol use (Fig. 4). Globally, 22.70% (14.76*10^4^) of the deaths of cirrhosis attributable to alcohol use occurred in female, compared with 77.30% (56.52*10^4^) in male in 2019 (Table 1). And the number of DALYs of cirrhosis attributable to alcohol use in male was 4.5 times than that in females. In all regions, the burden of cirrhosis attributable to alcohol use was much higher in male than that in female.

The ASDR and age-standardized DALY rate of liver cancer ascribed to alcohol use dropped in both sex over the past 30 years, especially in female with AAPCs −0.942 (95% CI = −1.047 to −0.836) and −1.241 (95% CI = −1.342 to −1.139), respectively. And the burden of liver cancer attributable to alcohol consumption also was higher in males than females in the world. More details are shown in Additional file 1: Table S9–S12.

### The projection in the burden of cirrhosis and liver cancer attributable to alcohol use

**Fig. 5:**
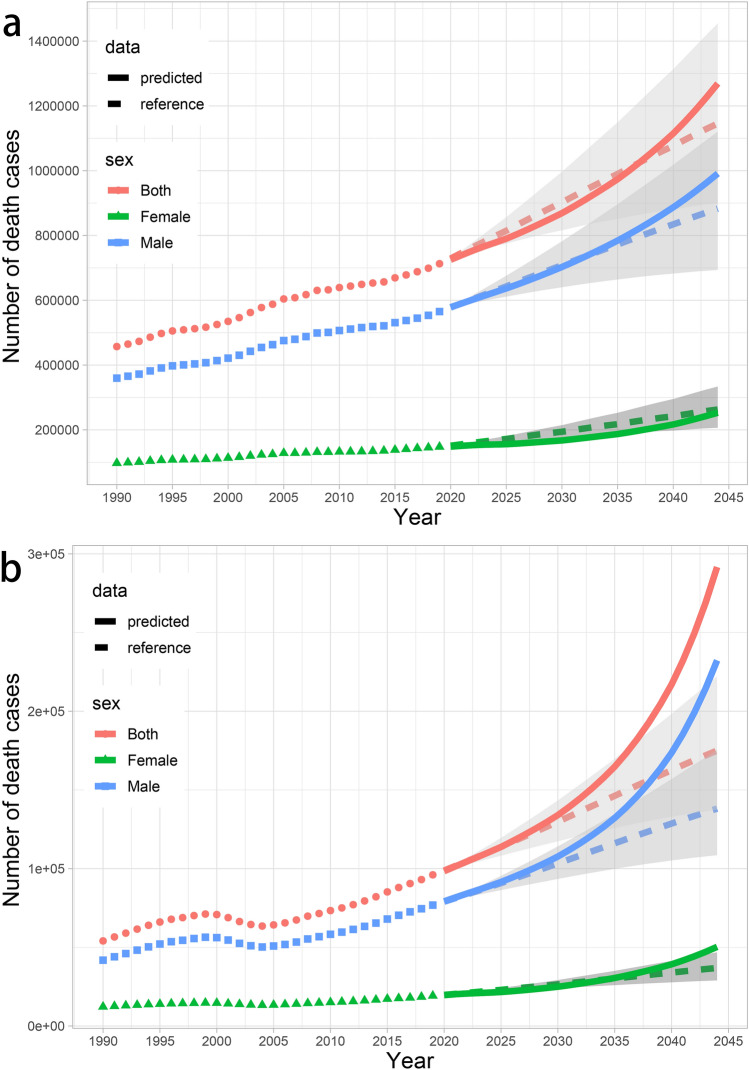
The observed (dashed line) and predicted (solid line) deaths of cirrhosis (**A**) and liver cancer (**B**) attributable to alcohol consumption from 1990 to 2044. The upper bound of Shading represents the rate increased by 1% per year (pessimistic reference) and the lower bound represents decreased by 1% per year (optimistic reference) based on the rate observed in 2019

From 2020 to 2044, the number of deaths from cirrhosis attributable to alcohol consumption would continue to increase in the world, especially among males. In Fig. 5A, our predicted results for both sexes were slightly lower than negative reference that 1% increased rate annually. And the number of female deaths would increase from 0.15 million in 2019 to 0.25 million, always be below the baseline (Table S13). But after 2033, the number of male deaths will be higher than the baseline, reaching 0.99 million in 2044. Although the predicted number of cirrhosis deaths attributable to alcohol use will continue to increase, the ASDR will gradually decrease year by year globally (Figure S4). However, the ASDR for male would remain stable for a period of time and then begin to increase.

Deaths from liver cancer attributable to alcohol use would increase rapidly over the next 25 years, especially in male, reaching 0.23 million (Fig. 5B). Among the female, the number of deaths would increase to 0.05 million in 2044. In 2035, our predicted number of liver cancer deaths will be higher than the negative reference, which need to be taken seriously. Fortunately, the ASDR would continue to decrease for both sexes (Figure S4).

### The relationship between AAPC and ASR of cirrhosis and liver cancer attributable to alcohol use and SDI

**Fig. 6:**
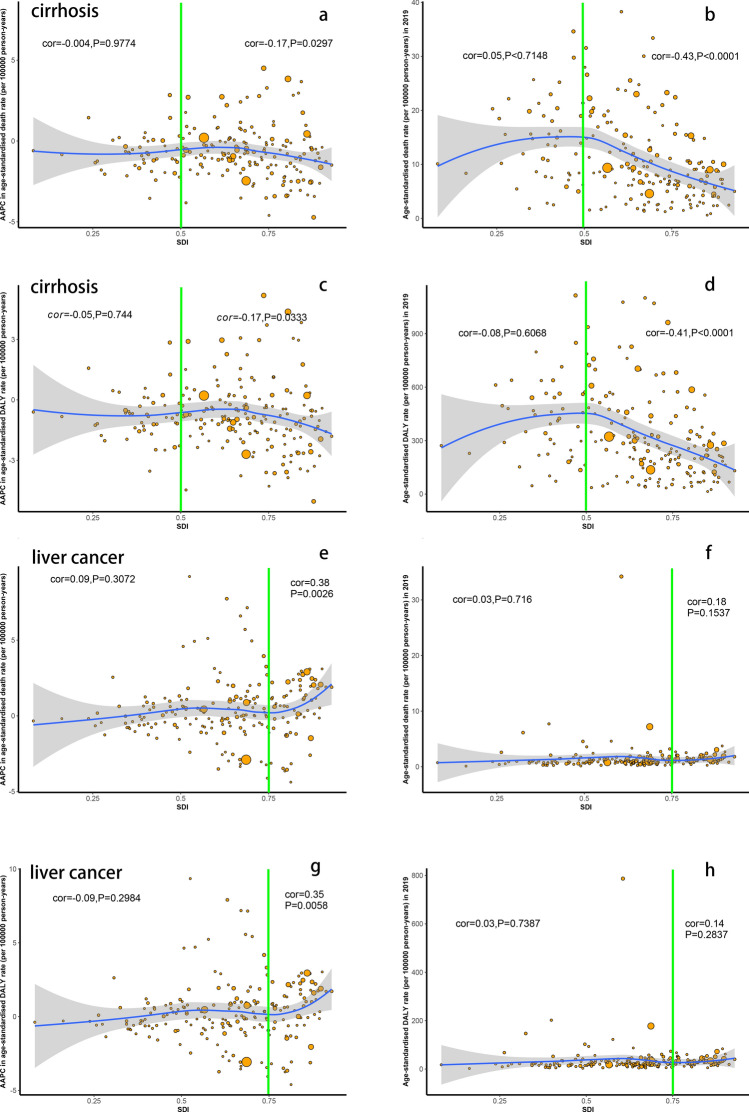
The relationship between SDI in 2019 and ASR and AAPC of cirrhosis and liver cancer attributable to alcohol use in 2019. **A** The correlation between AAPC of ASDR for cirrhosis and SDI; **B** The correlation between ASDR for cirrhosis in 2019 and SDI; **C** The correlation between AAPC of age-standardized DALY rate for cirrhosis and SDI; **D** The correlation between age-standardized DALY rate for cirrhosis in 2019 and SDI; **E** The correlation between AAPC of ASDR for liver cancer and SDI; **F** The correlation between ASDR for liver cancer in 2019 and SDI; **G** The correlation between AAPC of age-standardized DALY rate for liver cancer and SDI; **H** The correlation between age-standardized DALY rate for liver cancer in 2019 and SDI. Each point represents a country or a region that were available on SDI data, and the size of the point represents the number of cirrhosis or liver cancer deaths attributable to alcohol use. The cor indices Pearson’s correlation coefficient and *P* values were derived from Pearson’s correlation analysis. *AAPC* average annual percentage change; *ASR* age-standardized rate; *SDI* socio-demographic index; *ASDR* age-standardized death rate

Figure 6 A and C shows a negative association between AAPC of ASR of cirrhosis attributable to alcohol use and SDI in 2019 in the countries and territories with SDI level ≥ 0.5 (AAPC of ASDR: cor = −0.17, *p* = 0.0297; AAPC of age-standardized DALY rate: cor = −0.17, *p* = 0.0333), whereas there was a non-significant association in the countries and territories with SDI level < 0.5. Surprisingly, we found a more negative association between ASR of cirrhosis attributable to alcohol use and SDI in 2019, especially when SDI level exceeded 0.5 (Fig. 6B, D). In addition, Figure S5 shows a similar association between ASR of cirrhosis attributable to alcohol use and SDI in GBD regions.

We found a significant positive association between AAPC of ASR of liver cancer ascribed to alcohol use and SDI in 2019 in the countries and territories with SDI level ≥ 0.75 (AAPC of ASDR: cor = 0.38, *p* = 0.0026; AAPC of age-standardized DALY rate: cor = 0.35 *p* = 0.0058), but a non-significant association in the countries and territories with a SDI < 0.75 (Fig. 6E, G). Moreover, there was a non-significant association between the ASR of liver cancer ascribed to alcohol use and SDI in 2019 (Fig. 6F, H).

## Discussion

In this study, we revealed the cirrhosis and liver cancer burden ascribed to alcohol consumption along with their temporal trends in the world from 1990 to 2019 and summarized projections till 2044. Although the number of deaths and DALYs of cirrhosis and liver cancer attributable to alcohol use continued to increase in most regions from 1990 to 2019, the ASDR and age-standardized DALY rates decreased incrementally with substantial regional heterogeneity. On the SDI level, the ASR of cirrhosis attributable to alcohol use decreased in all SDI regions except for low-middle SDI regions where ASR remained stable, whereas the ASR of liver cancer attributable to alcohol use only increased in high-SDI regions. In addition, the burden of cirrhosis and liver cancer attributable to alcohol was greater in group aged 40 + years than that in younger population group. And the burden in male was higher than that in female over the past 30 years. It is calculated that, if there is no further intervention, the death cases and DALY number of cirrhosis and liver cancer attributable to alcohol will continue to increase over the next 25 years, especially among men. Therefore, given the unfavorable trend in the burden of cirrhosis and liver cancer attributable to alcohol, there is a need to reallocate limited medical resources and update prevention strategies to control these diseases.

With the growth and aging of the population, the number of deaths and DALYs of cirrhosis and liver cancer attributable to alcohol use inevitably increased [23]. Fortunately, the ASDR and age-standardized DALY rate decreased. One potential explanation for this decrease in ASR with regional heterogeneity is that temporal trends of alcohol consumption. Of note, we found that Central Asia and Eastern Europe have the highest rates of ASDR and age-standardized DALY for cirrhosis attributable to alcohol consumption, which is comparable to previous studies [24]. These conditions are largely attributed to the distribution of alcohol consumption in these regions [25]. In previous studies, high per capita alcohol consumption and a high prevalence of heavy episodic drinking were observed in Eastern Europe [26]. And the life expectancy in men aged 20–64 years in Eastern Europe decreased by about 25% compared to Western Europe [27]. Although the alcohol-related burden in Eastern Europe has declined following the implementation of a number of alcohol-related policies [28], the prevalence of harmful alcohol consumption in Eastern Europe has still increased in recent studies [13]. This means that Eastern Europe still has a long way to go on the road to alcohol control. Central Asia is a region of greatest concern, with the highest burden of both alcohol-related cirrhosis and liver cancer. In Central Asia, alcohol per capita consumption was not high, but the prevalence of alcohol use disorders was high, highlighting the need to strengthen alcohol control in this region [4, 26].

Typically, the prevalence of alcohol use is highest in high-SDI regions [25]. However, we found that the burden of cirrhosis from alcohol was highest in low–middle and low-SDI regions. One potential explanation for this is that high-SDI regions generate less disease burden per liter of alcohol consumed compared to low and low–middle SDI regions [29]. In general, the higher the level of SDI of a country, the higher the level of economic wealth, education, and health care [30]. Many studies demonstrated individuals with low socioeconomic status (low education and income) have a higher risk of death, particularly alcohol-attributable mortality [31, 32]. In addition, environmental and other risk factors have been identified to increase the harm caused by alcohol in low SDI countries, such as smoking and hepatitis B and C infections [33]. For example, despite relatively low per capita alcohol consumption [34], the sub-Saharan African region has a high burden of cirrhosis and liver cancer attributable to alcohol use owing to its large share of the global burden of chronic hepatitis B and C viruses [35, 36]. Furthermore, cirrhosis in most parts of underdeveloped countries was difficult to get formal treatment due to poor medical care, leading to increased mortality and DALY rates [37]. Currently, there is no significant downward trend in the burden of alcohol-induced cirrhosis in the low–middle regions. On one hand, due to growing economic wealth in the low–middle SDI region, disposable income per capita has increased and the alcohol industry has developed, ultimately causing an increase in alcohol consumption [4]. On the other hand, there is a lack of effective alcohol regulation measures in the low–middle SDI region [38]. In one latest survey, the prevalence of current drinking and heavy episodic drinking was highest in lower middle-income countries [39]. Therefore, implementing of effective alcohol policies in rapidly developing low-income countries is warranted. Meanwhile, increased investment in health and education is also an effective means of reducing the burden.

In addition, we found a positive correlation between the burden of liver cancer ascribed to alcohol use and SDI in countries or territories with SDI level ≥ 0.75. The reason for this phenomenon may be the relatively high per capita alcohol consumption and alcohol prevalence in high-SDI regions [29, 34]. In addition, the aging population can also be used to explain this phenomenon. Population aging tends to be more severe in high-income countries due to advances in health care. And liver cancer occurs more frequently in the elderly for sex [40]. Therefore, the worse the aging of the population, the greater the burden of liver cancer [23]. This interpretation is further supported by our findings that death and DALY rates in the 65 + age group changed the most from 1990 to 2019 in the high-SDI region. There is a need to implement alcohol public health policies for elderly in the high-SDI region. Early screening and early intervention for high-risk population might be served as an effective preventive measure to reduce the liver cancer burden ascribed to alcohol [41].

Most of the burden of cirrhosis and liver cancer attributable to alcohol consumption is distributed among people aged 40 years and older. However, the death and DALY rate from cirrhosis and liver cancer attributable to alcohol use in people under 40 years has increased from 1990 to 2019, especially in the Central Asia and Eastern Europe. The reason for this phenomenon might be that increasing number of people under 40 are consuming harmful amounts of alcohol [13]. Therefore, there is a need for targeted alcohol control policies and interventions for young population. In general, women are more vulnerable to alcohol-related harm than men [42, 43]. A large prospective study has demonstrated that female had a higher relative risk of developing alcohol-associated cirrhosis with increasing alcohol consumption than male [44]. However, our results confirmed that the burden of cirrhosis and liver cancer caused by alcohol was greater in male, compared with female, owing to their substantially higher levels of alcohol consumption [45]. And the ASDR from cirrhosis and liver cancer attributable to alcohol use in male will increase and in female will decrease, which means gender differences are more pronounced in alcohol-related cirrhosis and liver cancer. Thus, it is necessary to strengthen restrictions on alcohol use in male.

Alcohol consumption increases the risk of cirrhosis and liver cancer in patient with chronic liver disease, such as hepatitis B and hepatitis C [46, 47]. In addition, many risk factors interact with alcohol in a synergistic manner for development of cirrhosis and liver cancer [48]. Obesity, affecting more than 2 billion people in the world, is an independent risk factor for cirrhosis and liver cancer [49]. In a cross-section study from United States found that consuming more than 30 g of alcoholic drinks per day and the presence of obesity have a synergistic effect on elevated serum alanine and aspartate [50]. In a prospective cohort study of 23,712 Taiwanese residents, alcohol use and obesity showed a synergistic association with the risk of development liver cancer (adjusted HR 3.82; 95% CI 1.94–7.52). Moreover, diabetes, smoking and gut microbiota increase the risk of alcohol consumption leading to liver cirrhosis and liver cancer [51–53].

Although the burden of alcohol-induced cirrhosis and liver cancer is high, it is completely preventable. Reducing alcohol consumption is, therefore, expected to reduce the global burden of cirrhosis and liver cancer. According to the 2016 Global Alcohol and Health Survey, most countries have already introduced national alcohol policies, but these are not been fully implemented [54]. Here are some recommendations for policies to reduce the alcohol-related burden: (1) raising taxes and prices on alcohol is one of the most effective strategies, especially in low-income countries with rapidly growing economies [55]; (2) raising the national legal age for drinking to prevent early drinking by young people, who accounts for the majority of individuals consuming harmful amounts of alcohol [13]; (3) regulating the alcohol industry's intervention in alcohol policy and raising public awareness of alcohol through media and campaigns.

Alcohol consumption was modified by Coronavirus disease 2019. Prolonged quarantine and social isolation have led to an increase in stress and anxiety, which may affect mental health and lead to alcohol abuse [56, 57]. A number of studies have reported an increase in alcohol consumption during the COVID-19 pandemic, especially among those experiencing more stress [58, 59]. In addition, patients with alcohol-related liver disease have a worse outcome when combined with COVID-19 infection compared to other diseases [60, 61]. And approximately 20% increase in mortality from alcohol-related liver disease during COVID-19 pandemic has been reported [62]. Public health policy makers need to address this phenomenon with caution, adopt optimal intervention to decrease the burden.

There are limitations to this study, which mainly exist in all GBD studies [63]. First, although the GBD study provides estimates data based on robust statistical methods, because real data are not available, it is inevitable to introduce bias in our current study. Second, the data were collected by cancer-related authorities in different countries and their diagnostic criteria may be different, so there was uncertainty existed. Third, our prediction results may underestimate the contribution of alcohol use to cirrhosis and liver cancer burden. On one hand, the instability of the prediction model, on the other hand, unrecorded alcohol consumption was not considered. Fourth, the short-term trend of cirrhosis and liver cancer attributable to may be masked by the assessment of the long-term trend from 1990 to 2019. Finally, the GBD study used countries as its basic unit and lacked information about races, which may lead to the effect of race being overlooked.

## Conclusion

Overall, developed regions had higher burden of liver cancer attributable to alcohol use, whereas underdeveloped regions had higher burden of cirrhosis attributable to alcohol use. Globally, deaths and DALYs of cirrhosis and liver cancer attributable to alcohol use were on the rise gradually, but the ASDR and age-standardized DALY rates are on the decline gradually. This trend is expected to continue through 2044. Therefore, steps against alcohol use should be further strengthened and improved through effective national policies, which should take into account different genders, age groups, and regions based on the results of our study. In the meantime, plans and measures to monitor and treat chronic liver disease should be formulated in underdeveloped regions to reduce the burden of cirrhosis and liver cancer ascribed to alcohol.

## Supplementary Information

Below is the link to the electronic supplementary material.

Supplementary file1 (DOCX 2846 KB)

## Data Availability

The data described in this article are openly available in the GBD database (http://ghdx.healthdata.org/gbd-results-tool).
