# Supplementary material for: Contribution of alcohol use to the global burden of cirrhosis and liver cancer from 1990 to 2019 and projections to 2044
Source: Hepatol Int. 2023 Mar 5;17(4):1028–44. doi: 10.1007/s12072-023-10503-2 (PMC9985909; doi:10.1007/s12072-023-10503-2)

*1.****Supplementary Figures and Tables***

***Supplementary Tables***

**Table** **S1** Three regions with the largest and lowest number of deaths or DALYs of cirrhosis attributable to alcohol use.

**Table** **S2** Three countries with the largest and lowest number of deaths or DALYs of cirrhosis attributable to alcohol use.

**Table** **S3** Three regions with the largest and lowest number of deaths or DALYs of liver cancer attributable to alcohol use.

**Table** **S4** Three countries with the largest and lowest number of deaths or DALYs of liver cancer attributable to alcohol use.

**Table S5** The death cases and age-standardized death rate of cirrhosis attributable to alcohol use in 1990 and 2019, and its temporal trends from 1990 to 2019.

**Table S6** The DALYs and age-standardized DALY rate of cirrhosis attributable to alcohol use in 1990 and 2019, and its temporal trends from 1990 to 2019.

**Table S7** The death cases and age-standardized death rate of liver cancer attributable to alcohol use in 1990 and 2019, and its temporal trends from 1990 to 2019.

**Table S8** The DALYs and age-standardized DALY rate of liver cancer attributable to alcohol use in 1990 and 2019, and its temporal trends from 1990 to 2019.

**Table** **S9** Age distribution of death rate (per 100,000) for cirrhosis attributable to alcohol use in different region by sex in 2019.

**Table** **S10** Age distribution of DALY rate (per 100,000) for cirrhosis attributable to alcohol use in different region by sex in 2019.

**Table** **S11** Age distribution of death rate (per 100,000) for liver cancer attributable to alcohol use in different region by sex in 2019.

**Table** **S12** Age distribution of DALY rate (per 100,000) for liver cancer attributable to alcohol use in different region by sex in 2019.

**Table** **S13** Prediction the burden of cirrhosis and liver cancer attributable to alcohol use.

***Supplementary Figures***

**Figure S1** The rank of ASR burden for cirrhosis and liver cancer attributable to alcohol use over the past 30 years. **A**: ASDR in cirrhosis; **B**: age-standardized DALY rate in cirrhosis; **C**: ASDR in liver cancer; **D**: age-standardized DALY rate in liver cancer.

ASR: age-standardized rate; ASDR: age-standardized death rate.

**Figure S2** The proportion of deaths or DALYs of cirrhosis (**A, B**) and liver cancer (**C, D**) attributable to alcohol use in different age groups across global, SDI regions, and 21 GBD regions.

GBD: Global Burden of Diseases, Injuries, and Risk Factors Study; SDI: socio-demographic index

**Figure S3** The percentage change in death and DALY rate of cirrhosis (**A, B**) and liver cancer (**C, D**) attributable to alcohol use in different age groups for global, SDI and 21 GBD regions

GBD: Global Burden of Diseases, Injuries, and Risk Factors Study; SDI: socio-demographic index

**Figure S4 The** predicted trend of ASDR of cirrhosis and liver cancer attributable to alcohol use by sex until 2044. **A**: male’s ASDR of cirrhosis attributable to alcohol use; **B**: female’s ASDR of cirrhosis attributable to alcohol use; **C**: male’s ASDR of liver cancer attributable to alcohol use; **D**: female’s ASDR of liver cancer attributable to alcohol use.

ASDR: age-standardized death rate.

**Figure S5** The trend of cirrhosis and liver cancer burden attributable to alcohol use cross 21 GBD regions, 1990-2019. **A**: The trend of ASDR of cirrhosis; **B**: The trend of ASDR of liver cancer; **C**:The trend of age-standardized DALY rate in cirrhosis; **D**:The trend of age-standardized DALY rate in liver cancer. For each region, points from left to right depict estimates from each year from 1990 to 2019.

GBD: Global Burden of Diseases, Injuries, and Risk Factors Study; ASDR: age-standardized death rate.

**Table** S1 Three regions with the largest and lowest number of deaths or DALYs of cirrhosis attributable to alcohol use.

| measure | sex | | | top three countries | | |  | buttom three countries | | |
| --- | --- | --- | --- | --- | --- | --- | --- | --- | --- | --- |
| 2019 ASR(per100,000 people) | | | | | | | | | | |
| ASDR | | | | | | | | | | |
|  | both | | Central Asia(22.49) | | Eastern Sub-Saharan Africa(19.46) | Western Sub-Saharan Africa(18.58) | | North Africa and Middle East(2.69) | Oceania(3.8) | Australasia(4.04) |
|  | female | | Central Asia(11.82) | | Eastern Europe(9.41) | Eastern Sub-Saharan Africa(9.18) | | Oceania(0.84) | East Asia(1.05) | North Africa and Middle East(1.2) |
|  | male | | Central Asia(35.42) | | Eastern Sub-Saharan Africa(30.79) | Western Sub-Saharan Africa(30.21) | | North Africa and Middle East(4.11) | Australasia(6.13) | Oceania(6.63) |
| Age Standardized DALY Rate | | | | | | | | | | |
|  | both | | Central Asia(719.95) | | Eastern Europe(662.39) | Eastern Sub-Saharan Africa(539.6) | | North Africa and Middle East(69.63) | Australasia(115.86) | Oceania(132.58) |
|  | female | | Eastern Europe(362.43) | | Central Asia(335.01) | Eastern Sub-Saharan Africa(231.72) | | Oceania(23.92) | East Asia(24.43) | North Africa and Middle East(26.79) |
|  | male | | Central Asia(1151.91) | | Eastern Europe(1009.97) | Central Latin America(881.82) | | North Africa and Middle East(109.81) | Australasia(175.5) | Oceania(235.74) |
| 1990-2019 increase times | | | | | | | | | | |
| Death(cases) | | | | | | | | | | |
|  | both | | Central Asia(2.83) | | Eastern Europe(2.73) | Southeast Asia(2.53) |  | High-income Asia Pacific(0.77) | Western Europe(0.85) | Central Europe(1.04) |
|  | female | | Eastern Europe(2.54) | | Central Asia(2.5) | Central Sub-Saharan Africa(2.42) | | Central Europe(0.99) | High-income Asia Pacific(0.96) | Western Europe(0.84) |
|  | male | | Central Asia(2.98) | | Eastern Europe(2.83) | Southeast Asia(2.73) |  | High-income Asia Pacific(0.72) | Western Europe(0.85) | Central Europe(1.06) |
| DALY(Year) | | | | | | | | | | |
|  | both | | Eastern Europe(3.05) | | Central Asia(3.03) | Southeast Asia(2.41) |  | High-income Asia Pacific(0.58) | Western Europe(0.75) | East Asia(0.92) |
|  | female | | Eastern Europe(2.87) | | Central Asia(2.6) | Central Sub-Saharan Africa(2.37) | | High-income Asia Pacific(0.65) | Western Europe(0.73) | East Asia(0.9) |
|  | male | | Central Asia(3.19) | | Eastern Europe(3.14) | Southeast Asia(2.6) |  | High-income Asia Pacific(0.56) | Western Europe(0.76) | East Asia(0.93) |
| AAPC | | | | | | | | | | |
| Death | | | | | | | | | | |
|  | both | Eastern Europe(3.408) | | | Central Asia(1.883) | Southeast Asia(0.405) |  | High-income Asia Pacific(-3.149) | East Asia(-2.436) | Western Europe(-2.001) |
|  | female | Eastern Europe(3.315) | | | Central Asia(1.731) | High-income North America(0.688) | | High-income Asia Pacific(-3.124) | East Asia(-2.863) | Western Europe(-2.038) |
|  | male | Eastern Europe(3.147) | | | Central Asia(1.844) | Southeast Asia(0.705) |  | High-income Asia Pacific(-3.259) | East Asia(-2.296) | Western Europe(-2.114) |
| DALY | | | | | | | | | | |
|  | both | Eastern Europe(3.939) | | | Central Asia(1.978) | Southeast Asia(0.344) |  | High-income Asia Pacific(-3.392) | East Asia(-2.648) | Western Europe(-2.171) |
|  | female | Eastern Europe(3.862) | | | Central Asia(1.638) | High-income North America(0.564) | | High-income Asia Pacific(-3.279) | East Asia(-3.159) | Western Europe(-2.25) |
|  | male | Eastern Europe(3.776) | | | Central Asia(2.054) | Southeast Asia(0.626) |  | High-income Asia Pacific(-3.489) | East Asia(-2.513) | Western Europe(-2.213) |

**Table** S2 Three countries with the largest and lowest number of deaths or DALYs of cirrhosis attributable to alcohol use.

| measure | sex | top three countries | | |  | buttom three countries | | |
| --- | --- | --- | --- | --- | --- | --- | --- | --- |
| 2019 ASR(per100,000 people) | | | | | | | | |
| ASDR | | | | | | | | |
|  | both | Mongolia(38.25) | Cambodia(34.61) | Republic of Moldova(33.39) | | Kuwait(0.61) | Singapore(0.89) | Jordan(1.26) |
|  | female | Mongolia(23.04) | Republic of Moldova(22.47) | Uzbekistan(15.33) |  | Kuwait(0.27) | Papua New Guinea(0.4) | Singapore(0.41) |
|  | male | Cambodia(61.38) | Mongolia(57.31) | Zambia(49.49) |  | Kuwait(0.85) | Singapore(1.36) | Jordan(1.91) |
| Age Standardized DALY Rate | | | | | | | | |
|  | both | Cambodia(1114.54) | Turkmenistan(1100.71) | Mongolia(1076.76) |  | Kuwait(14.52) | Singapore(25.71) | Jordan(34.42) |
|  | female | Republic of Moldova(703.56) | Mongolia(554.2) | Ukraine(469.96) |  | Kuwait(6) | Singapore(11.26) | Jordan(11.29) |
|  | male | Cambodia(1941.09) | Turkmenistan(1812.3) | Mongolia(1677.98) |  | Kuwait(20.52) | Singapore(39.25) | Sudan(50.29) |
| 1990-2019 increase times | | | | | | | | |
| Death(cases) | | | | | | | | |
|  | both | Cambodia(5.78) | Viet Nam(5.35) | Turkmenistan(4.38) | | Portugal(0.54) | Hungary(0.56) | Sudan(0.58) |
|  | female | Angola(6.41) | Qatar(4.75) | Nicaragua(4.27) |  | Slovenia(0.41) | Croatia(0.44) | Portugal(0.46) |
|  | male | Cambodia(7.04) | Viet Nam(6.62) | Turkmenistan(5.31) | | Sudan(0.51) | Italy(0.57) | Portugal(0.57) |
| DALY(Year) | | | | | | | | |
|  | both | Viet Nam(5.87) | Cambodia(5.71) | Turkmenistan(4.99) | | Hungary(0.48) | Sudan(0.48) | Italy(0.49) |
|  | female | Angola(6.32) | Qatar(4.48) | Equatorial Guinea(3.95) | | Slovenia(0.35) | Croatia(0.36) | Portugal(0.38) |
|  | male | Viet Nam(6.94) | Cambodia(6.72) | Turkmenistan(5.84) |  | Sudan(0.41) | Republic of Korea(0.48) | Italy(0.49) |
| AAPC | | | | | | | | |
| Death | | | | | | | | |
|  | both | Ukraine(4.504) | Russian Federation(3.843) | Lithuania(3.673) |  | Republic of Korea(-4.726) | Bahrain(-4.606) | Sudan(-3.825) |
|  | female | Ukraine(4.208) | Russian Federation(4.1) | Lithuania(3.774) |  | Bahrain(-5.604) | Bermuda(-4.441) | Slovenia(-4.345) |
|  | male | Ukraine(4.262) | Cambodia(3.681) | Lithuania(3.45) |  | Republic of Korea(-5.029) | Bahrain(-4.737) | Sudan(-4.335) |
| DALY | | | | | | | | |
|  | both | Ukraine(5.186) | Russian Federation(4.371) | Lithuania(3.841) |  | Republic of Korea(-5.041) | Sudan(-4.473) | Bahrain(-4.287) |
|  | female | Ukraine(4.894) | Russian Federation(4.678) | Belarus(4.001) |  | Bahrain(-6.077) | Slovenia(-4.736) | Republic of Korea(-4.714) |
|  | male | Ukraine(4.907) | Russian Federation(4.055) | Lithuania(3.663) |  | Republic of Korea(-5.26) | Sudan(-5.1) | Bahrain(-4.373) |

**Table** S3 Three regions with the largest and lowest number of deaths or DALYs of liver cancer attributable to alcohol use.

| measure | sex | top three countries | | |  | buttom three countries | | |
| --- | --- | --- | --- | --- | --- | --- | --- | --- |
| 2019 ASR(per100,000 people) | | | | | | | | |
| ASDR | | | | | | | | |
|  | both | Central Asia(2.6) | Southeast Asia(1.9) | Australasia(1.59) |  | Central Sub-Saharan Africa(0.36) | Oceania(0.47) | North Africa and Middle East(0.61) |
|  | female | Central Asia(1.23) | Andean Latin America(0.88) | Southeast Asia(0.59) | | Oceania(0.17) | Central Sub-Saharan Africa(0.17) | North Africa and Middle East(0.19) |
|  | male | Central Asia(4.52) | Southeast Asia(3.53) | Western Europe(2.92) | | North Africa and Middle East(1.01) | Oceania(0.76) | Central Sub-Saharan Africa(0.61) |
| Age Standardized DALY Rate | | | | | | | | |
|  | both | Central Asia(63.03) | Southeast Asia(44.49) | Australasia(38.37) | | Central Sub-Saharan Africa(8.83) | Oceania(10.9) | North Africa and Middle East(14.63) |
|  | female | Central Asia(28.77) | Andean Latin America(18.3) | Southeast Asia(13.01) | | Oceania(4.09) | Central Sub-Saharan Africa(4.44) | North Africa and Middle East(4.52) |
|  | male | Central Asia(107.18) | Southeast Asia(80.94) | Southern Sub-Saharan Africa(67.81) | | Central Sub-Saharan Africa(14.31) | Oceania(17.3) | North Africa and Middle East(24.3) |
| 1990-2019 increase times | | | | | | | | |
| Death(cases) | | | | | | | | |
|  | both | Central Asia(4.61) | High-income North America(3.98) | Australasia(3.88) |  | East Asia(0.96) | Central Europe(0.98) | Caribbean(1.11) |
|  | female | Central Asia(5.17) | Australasia(4.25) | High-income North America(3.12) | | Central Europe(0.73) | Caribbean(0.88) | East Asia(0.89) |
|  | male | Central Asia(4.44) | High-income North America(4.17) | Tropical Latin America(3.86) | | East Asia(0.98) | Central Europe(1.09) | Caribbean(1.21) |
| DALY(Year) | | | | | | | | |
|  | both | Central Asia(4.72) | High-income North America(3.97) | Australasia(3.57) |  | East Asia(0.86) | Central Europe(0.91) | Caribbean(1.09) |
|  | female | Central Asia(5.35) | Australasia(3.87) | High-income North America(3.08) | | Central Europe(0.68) | East Asia(0.78) | Caribbean(0.88) |
|  | male | Central Asia(4.55) | High-income North America(4.14) | Australasia(3.51) |  | East Asia(0.88) | Central Europe(1) | Caribbean(1.19) |
| AAPC | | | | | | | | |
| Death | | | | | | | | |
|  | both | Central Asia(3.775) | High-income North America(2.892) | Eastern Europe(2.619) | | East Asia(-2.867) | Caribbean(-1.928) | Central Europe(-1.406) |
|  | female | Central Asia(4.427) | Australasia(2.619) | High-income North America(2.215) | | East Asia(-3.401) | Caribbean(-2.843) | Central Europe(-2.492) |
|  | male | Central Asia(3.336) | High-income North America(2.789) | Eastern Europe(2.573) | | Andean Latin America(-1.145) | Caribbean(-1.609) | East Asia(-2.635) |
| DALY | | | | | | | | |
|  | both | Central Asia(3.622) | High-income North America(2.893) | Eastern Europe(2.593) | | East Asia(-3.046) | Caribbean(-1.924) | Andean Latin America(-1.469) |
|  | female | Central Asia(4.304) | Australasia(2.418) | High-income North America(2.144) | | East Asia(-3.719) | Caribbean(-2.779) | Central Europe(-2.434) |
|  | male | Central Asia(3.228) | High-income North America(2.866) | Eastern Europe(2.584) | | East Asia(-2.804) | Caribbean(-1.637) | Andean Latin America(-1.361) |

**Table** S4 Three countries with the largest and lowest number of deaths or DALYs of liver cancer attributable to alcohol use.

| measure | sex | top three countries | | | buttom three countries | | |
| --- | --- | --- | --- | --- | --- | --- | --- |
| 2019 ASR(per100,000 people) | | | | | | | |
| ASDR | | | | | | | |
|  | both | Mongolia(34.21) | Gambia(7.71) | Thailand(7.2) | Niger(0.11) | Cameroon(0.15) | Tunisia(0.21) |
|  | female | Mongolia(18.44) | Gambia(2.13) | Thailand(2.09) | Niger(0.04) | Cameroon(0.05) | Tunisia(0.06) |
|  | male | Mongolia(56.79) | Gambia(13.69) | Thailand(13.26) | Niger(0.18) | Cameroon(0.25) | Senegal(0.36) |
| Age Standardized DALY Rate | | | | | | | |
|  | both | Mongolia(787.04) | Gambia(202.03) | Thailand(177.93) | Niger(2.45) | Cameroon(3.53) | Tunisia(4.84) |
|  | female | Mongolia(394.25) | Gambia(52.85) | Thailand(47.32) | Niger(0.89) | Cameroon(1.3) | Tunisia(1.39) |
|  | male | Mongolia(1295.31) | Gambia(354.23) | Thailand(326.9) | Niger(4.11) | Cameroon(5.92) | Papua New Guinea(8) |
| 1990-2019 increase times | | | | | | | |
| Death(cases) | | | | | | | |
|  | both | Cabo Verde(21.65) | Uzbekistan(15.86) | Turkmenistan(14.39) | Hungary(0.49) | Dominica(0.5) | Saint Kitts and Nevis(0.52) |
|  | female | Cabo Verde(18.37) | Uzbekistan(14.49) | Armenia(11.71) | Poland(0.34) | Saint Kitts and Nevis(0.34) | Dominica(0.37) |
|  | male | Cabo Verde(22.9) | Uzbekistan(16.43) | Turkmenistan(15.49) | Hungary(0.51) | Dominica(0.57) | Saint Kitts and Nevis(0.6) |
| DALY(Year) | | | | | | | |
|  | both | Cabo Verde(25.23) | Uzbekistan(18.69) | Turkmenistan(16.84) | Hungary(0.45) | Bermuda(0.51) | Poland(0.51) |
|  | female | Cabo Verde(18.64) | Uzbekistan(17.78) | Turkmenistan(13.88) | Poland(0.32) | Dominica(0.38) | Saint Kitts and Nevis(0.38) |
|  | male | Cabo Verde(27.75) | Uzbekistan(19.03) | Turkmenistan(17.9) | Hungary(0.47) | Bermuda(0.56) | Dominica(0.58) |
| AAPC | | | | | | | |
| Death | | | | | | | |
|  | both | Uzbekistan(7.735) | Armenia(7.139) | Turkmenistan(6.596) | Poland(-3.555) | Venezuela (Bolivarian Republic of)(-3.507) | Hungary(-3.466) |
|  | female | Uzbekistan(7.706) | Armenia(6.99) | Turkmenistan(6.089) | Poland(-5.125) | Venezuela (Bolivarian Republic of)(-5.037) | Cuba(-4.697) |
|  | male | Uzbekistan(7.483) | Armenia(7.244) | Turkmenistan(6.333) | Hungary(-3.194) | Venezuela (Bolivarian Republic of)(-3.118) | Poland(-2.852) |
| DALY | | | | | | | |
|  | both | Uzbekistan(7.908) | Turkmenistan(7.182) | Armenia(7.15) | Venezuela (Bolivarian Republic of)(-3.729) | Poland(-3.655) | Hungary(-3.491) |
|  | female | Cabo Verde(8.476) | Uzbekistan(7.931) | Armenia(6.757) | Venezuela (Bolivarian Republic of)(-5.196) | Poland(-5.179) | Cuba(-4.847) |
|  | male | Uzbekistan(7.671) | Armenia(7.091) | Turkmenistan(6.955) | Hungary(-3.447) | Venezuela (Bolivarian Republic of)(-3.268) | Taiwan (Province of China)(-3.172) |

**Table S5** The death cases and age-standardized death rate of cirrhosis attributable to alcohol use in 1990 and 2019, and its temporal trends from 1990 to 2019.

| nation | sex | Death Case NO(95% UI) | | Change in absolute number (%) | age-standardized death rate per 100,000 No. (95% UI) | | 1990-2019 AAPC No.(95 CI) |
| --- | --- | --- | --- | --- | --- | --- | --- |
|  |  | 1990 | 2019 |  | 1990 | 2019 |  |
| Afghanistan | Both | 159.2(83.04-264.98) | 227.51(116.96-388.91) | 42.91 | 2.26(3.62-1.25) | 1.84(3-1.03) | -0.646(-0.775--0.518) |
| Albania | Both | 119.48(93.98-146.56) | 190.4(127.31-272.35) | 59.36 | 5.79(7.09-4.49) | 4.64(6.59-3.11) | -0.79(-1.154--0.425) |
| Algeria | Both | 215.12(122.22-337.94) | 626.5(352.02-1053.83) | 191.23 | 1.79(2.73-1.09) | 1.82(3.02-1.04) | 0.06(-0.028-0.147) |
| American Samoa | Both | 1.12(0.65-1.87) | 1.32(0.82-2.14) | 17.86 | 4.01(6.52-2.49) | 2.62(4.13-1.66) | -1.447(-1.707--1.187) |
| Andorra | Both | 4.88(2.93-7.55) | 9.04(6.22-12.32) | 85.25 | 8.78(13.54-5.27) | 6.45(8.9-4.42) | -1.059(-1.096--1.021) |
| Angola | Both | 901.79(547.29-1405.04) | 3775.89(2603.7-5192.34) | 318.71 | 19.75(30.11-12.05) | 29.76(40.54-20.95) | 1.432(1.255-1.609) |
| Antigua and Barbuda | Both | 3.68(2.83-4.55) | 7.22(5.2-9.45) | 96.20 | 7.26(9.07-5.49) | 6.95(9.08-5.03) | -0.089(-0.905-0.733) |
| Argentina | Both | 4669.76(3910.52-5382) | 6143.1(4813.18-7456.12) | 31.55 | 14.53(16.73-12.17) | 11.68(14.13-9.2) | -0.668(-0.797--0.539) |
| Armenia | Both | 172.85(135.07-220.46) | 524.28(400.17-675.06) | 203.32 | 6.19(7.76-4.82) | 13.12(16.93-10) | 2.707(2.16-3.256) |
| Australia | Both | 1079.85(905.52-1220.86) | 1619(1337.33-1899.69) | 49.93 | 5.73(6.47-4.81) | 4.27(4.98-3.55) | -0.984(-1.313--0.654) |
| Austria | Both | 1786.43(1478.57-2021.52) | 1368.38(1090.09-1605.78) | -23.40 | 17.01(19.22-14.16) | 8.65(10.11-6.95) | -2.414(-2.774--2.053) |
| Azerbaijan | Both | 898.16(676.96-1158.22) | 1822.52(1246.47-2529.36) | 102.92 | 17.2(22.46-12.98) | 20.55(29.15-13.82) | 0.631(0.408-0.854) |
| Bahamas | Both | 26.28(20.39-32.14) | 34.04(22.85-47.78) | 29.53 | 15.37(18.92-11.8) | 8.08(11.32-5.47) | -2.196(-2.573--1.818) |
| Bahrain | Both | 21.54(13.86-30.51) | 37.24(20.44-61.53) | 72.89 | 11.33(16.74-7.02) | 3.02(5.09-1.7) | -4.606(-5.166--4.043) |
| Bangladesh | Both | 4739.88(3178.52-6701.99) | 6476.17(4290.15-9328.44) | 36.63 | 9.43(13.44-6.25) | 5.01(7.13-3.3) | -1.996(-2.201--1.79) |
| Barbados | Both | 20.53(16.18-24.85) | 29.66(21.34-38.88) | 44.47 | 7.85(9.51-6.26) | 6.3(8.25-4.56) | -0.788(-1.135--0.44) |
| Belarus | Both | 559.98(438.17-685.47) | 1594.53(1108.73-2231.39) | 184.75 | 4.35(5.33-3.43) | 11.1(15.6-7.76) | 3.285(2.127-4.456) |
| Belgium | Both | 1396.41(1140.14-1599.78) | 1617(1281.86-1934.97) | 15.80 | 9.82(11.21-8.08) | 7.84(9.34-6.26) | -0.794(-0.938--0.649) |
| Belize | Both | 9.77(7.44-12.17) | 38.88(29.53-49.79) | 297.95 | 10.14(12.61-7.62) | 12.57(16.06-9.53) | 0.786(0.457-1.116) |
| Benin | Both | 385.57(244.3-561.71) | 829.61(514.8-1254.17) | 115.16 | 18.12(26.45-11.54) | 14.77(22.12-9.31) | -0.702(-0.865--0.538) |
| Bermuda | Both | 6.7(5.25-8.12) | 5.18(3.62-6.79) | -22.69 | 10.6(12.91-8.32) | 4.27(5.6-3) | -3.07(-3.216--2.923) |
| Bhutan | Both | 47.32(26.95-81.14) | 50.26(28.13-100.31) | 6.21 | 14.8(25.26-8.64) | 8.41(17.05-4.85) | -1.954(-2.107--1.8) |
| Bolivia (Plurinational State of) | Both | 659.38(415.47-981.35) | 1602.44(1061-2277.65) | 143.02 | 19.07(28.28-12.09) | 17.74(25.42-11.96) | -0.237(-0.39--0.084) |
| Bosnia and Herzegovina | Both | 463.99(370.51-556.36) | 390.79(277.61-522.91) | -15.78 | 10.31(12.37-8.35) | 6.81(9.13-4.85) | -1.466(-1.796--1.135) |
| Botswana | Both | 135.21(76.95-223.73) | 251.53(156.36-369.67) | 86.03 | 20.69(33.78-12.06) | 15.09(21.98-9.45) | -1.044(-1.227--0.86) |
| Brazil | Both | 13798.53(11212.51-16701.64) | 22777.81(18749.96-27979.69) | 65.07 | 13.21(15.96-10.84) | 9.32(11.47-7.69) | -1.171(-1.391--0.951) |
| Brunei Darussalam | Both | 4.85(3.07-7.25) | 8.35(5.62-11.5) | 72.16 | 3.76(5.55-2.44) | 2.33(3.19-1.63) | -1.588(-1.879--1.297) |
| Bulgaria | Both | 1666.27(1397.25-1907.05) | 2076.62(1516.03-2739.16) | 24.63 | 13.69(15.64-11.54) | 17.39(22.75-12.64) | 0.975(0.657-1.293) |
| Burkina Faso | Both | 1205.88(850.74-1608.14) | 1774.86(923.05-2834.11) | 47.18 | 27.73(36.8-19.24) | 18.48(29.05-9.54) | -1.34(-1.582--1.096) |
| Burundi | Both | 1004.45(582.04-1518.66) | 1144.97(611-2124.43) | 13.99 | 40.32(60.15-23.66) | 22.14(40.05-11.83) | -2.053(-2.244--1.861) |
| Cabo Verde | Both | 30.47(19.59-47.73) | 69.94(47.68-94.4) | 129.54 | 13.88(21.38-8.87) | 15.31(20.81-10.53) | 0.469(-0.173-1.115) |
| Cambodia | Both | 793.54(508.5-1210.22) | 4585.11(3103.66-6237.83) | 477.80 | 15.27(22.8-10.1) | 34.61(46.84-23.74) | 2.841(2.729-2.953) |
| Cameroon | Both | 1339.79(929.47-1840.14) | 2614.32(1546.33-4084.41) | 95.13 | 27.8(38.69-19.16) | 18.97(29.61-11.5) | -1.302(-1.395--1.209) |
| Canada | Both | 2151.75(1672.96-2633) | 3728.7(2861.27-4704.76) | 73.29 | 6.79(8.31-5.31) | 5.81(7.23-4.49) | -0.527(-0.596--0.457) |
| Central African Republic | Both | 354.98(197.87-577.16) | 407.87(186.16-744.8) | 14.90 | 26.26(42.31-14.72) | 15.55(27.5-7.6) | -1.796(-1.878--1.714) |
| Chad | Both | 397.97(197.94-695.98) | 1309.05(611.93-2103.37) | 228.93 | 13.5(23.22-6.86) | 20.29(32.77-9.91) | 1.444(1.206-1.683) |
| Chile | Both | 2925.81(2380.63-3428.75) | 3732.06(2945.69-4502.88) | 27.56 | 27.78(32.65-22.6) | 15.66(18.9-12.4) | -2.002(-2.525--1.477) |
| China | Both | 86760.36(62146.23-112911.82) | 91747.21(65438.24-120223.02) | 5.75 | 9.37(12.13-6.7) | 4.59(6.03-3.27) | -2.463(-2.616--2.31) |
| Colombia | Both | 1134.44(923.56-1350) | 1894.37(1313.23-2646.01) | 66.99 | 6.05(7.25-4.89) | 3.57(4.99-2.46) | -1.804(-2.79--0.808) |
| Comoros | Both | 17.54(7.98-33.32) | 35.06(20.15-58.86) | 99.89 | 7.87(14.78-3.72) | 7.12(11.65-4.11) | -0.302(-0.745-0.142) |
| Congo | Both | 318.56(193.13-465.91) | 586.13(317.37-908.09) | 83.99 | 26.96(38.92-16.62) | 19.89(30.4-10.81) | -1.039(-1.261--0.817) |
| Cook Islands | Both | 0.54(0.28-0.97) | 1.31(0.85-1.75) | 142.59 | 3.89(6.96-2.05) | 5.48(7.35-3.61) | 1.21(1.087-1.332) |
| Costa Rica | Both | 183.77(150.16-219.11) | 499.41(344.2-693.69) | 171.76 | 9.81(11.75-7.99) | 9.55(13.25-6.58) | -0.07(-0.549-0.41) |
| Croatia | Both | 1393.55(1141.19-1635.07) | 830.05(596.04-1102.83) | -40.44 | 21.57(25.23-17.74) | 10.64(14.2-7.65) | -2.494(-2.985--2.001) |
| Cuba | Both | 549(426.55-685.83) | 1189.75(829.43-1610.68) | 116.71 | 5.31(6.64-4.12) | 6.53(8.83-4.59) | 0.788(0.067-1.515) |
| Cyprus | Both | 65.64(45.41-90.24) | 93.61(71.38-117.35) | 42.61 | 8.83(12.54-5.98) | 5.09(6.42-3.87) | -1.914(-2.069--1.759) |
| Czechia | Both | 1826.21(1533.34-2090.33) | 1823.27(1357.05-2378.2) | -0.16 | 13.97(15.97-11.77) | 10.2(13.3-7.61) | -1.068(-1.532--0.601) |
| C么te d'Ivoire | Both | 1119.87(680.94-1656.79) | 2310.37(1425.96-3438.95) | 106.31 | 22.85(34.29-13.99) | 18.02(26.82-11.11) | -0.828(-0.987--0.669) |
| Democratic People's Republic of Korea | Both | 1834.23(1110.1-2712.82) | 2571.34(1361.92-3786.95) | 40.19 | 10.07(14.59-6.24) | 7.77(11.36-4.1) | -0.873(-0.915--0.831) |
| Democratic Republic of the Congo | Both | 3232.68(1870.47-4861.42) | 4705.29(2440-8197.08) | 45.55 | 18.29(27.81-10.54) | 11.06(18.93-5.75) | -1.682(-1.99--1.373) |
| Denmark | Both | 615.15(509.92-700.53) | 720.11(565.1-867.95) | 17.06 | 9.1(10.38-7.56) | 7.23(8.68-5.67) | -0.754(-1.018--0.49) |
| Djibouti | Both | 16.17(9.22-30.06) | 50.17(28.17-89) | 210.27 | 9.95(17.66-5.94) | 8.03(14.02-4.48) | -0.64(-0.782--0.498) |
| Dominica | Both | 6.37(4.62-8.35) | 6.4(4.47-8.82) | 0.47 | 9.72(12.69-7.08) | 7.31(10.06-5.09) | -1.005(-1.125--0.885) |
| Dominican Republic | Both | 739.16(567.6-929.18) | 1556.05(966.61-2317.33) | 110.52 | 18.32(22.98-14.07) | 16.36(24.05-10.31) | -0.35(-0.756-0.058) |
| Ecuador | Both | 641.48(507.55-811.1) | 1823.15(1275.53-2522.32) | 184.21 | 11.04(13.89-8.71) | 12(16.61-8.45) | 0.186(-0.117-0.491) |
| Egypt | Both | 2512.97(1457.84-3980.55) | 4906.72(2402.52-8852.6) | 95.26 | 9.34(14.61-5.47) | 8.46(14.55-4.35) | -0.347(-0.752-0.059) |
| El Salvador | Both | 454.45(358.79-560.29) | 725.08(496.72-1014.09) | 59.55 | 13.9(17.27-10.93) | 12.27(17.28-8.36) | -0.29(-0.875-0.297) |
| Equatorial Guinea | Both | 33.37(15.87-60.02) | 80.35(45.49-130.9) | 140.79 | 15.11(26.61-7.36) | 14.48(23.03-8.47) | -0.146(-0.478-0.186) |
| Eritrea | Both | 147.23(77.55-247.99) | 418.95(245.24-708.79) | 184.55 | 12.83(20.76-7.12) | 13.64(22.11-8.38) | 0.204(0.092-0.315) |
| Estonia | Both | 90.02(72.11-109.47) | 205.73(142.15-277.31) | 128.54 | 4.6(5.61-3.71) | 10.46(14.09-7.21) | 2.966(1.329-4.63) |
| Eswatini | Both | 70.69(47.25-100.4) | 119.51(74.1-182.98) | 69.06 | 21.14(30.05-14.1) | 17.82(26.96-11.26) | -0.615(-0.853--0.377) |
| Ethiopia | Both | 4834.5(2289.25-9087.29) | 8897.23(5441.37-13114.19) | 84.04 | 21.78(39.63-10.77) | 19.83(29.28-12.21) | -0.344(-0.459--0.23) |
| Fiji | Both | 23.94(15.89-33.15) | 36.83(22.95-54.15) | 53.84 | 5.09(7.02-3.41) | 4.44(6.46-2.85) | -0.416(-0.773--0.057) |
| Finland | Both | 438.29(360.84-516.73) | 827.6(658.91-989.83) | 88.82 | 6.79(7.97-5.62) | 9.08(10.8-7.26) | 0.971(0.379-1.566) |
| France | Both | 11102.34(9295.22-12441.47) | 8352.86(6716.18-9736.46) | -24.76 | 14.73(16.47-12.41) | 7.05(8.18-5.73) | -2.492(-2.725--2.26) |
| Gabon | Both | 219.62(143.84-305.78) | 278.46(174.82-399.36) | 26.79 | 37.53(52.69-24.32) | 24.75(35.63-15.77) | -1.423(-1.623--1.222) |
| Gambia | Both | 54.9(30.91-89.86) | 165.89(99.33-255.84) | 202.17 | 13.96(22.41-7.84) | 15.63(24.17-9.43) | 0.516(0.076-0.958) |
| Georgia | Both | 929.61(708.77-1158.17) | 1019.58(756.09-1296.19) | 9.68 | 14.87(18.52-11.39) | 19.62(24.84-14.59) | 0.867(0.55-1.185) |
| Germany | Both | 18509.65(15634.62-20552.66) | 16574.57(13449.56-19272.65) | -10.45 | 16(17.71-13.48) | 10.03(11.58-8.24) | -1.622(-1.892--1.351) |
| Ghana | Both | 1978.15(1227.75-3125.12) | 3408.09(2204.5-4858.57) | 72.29 | 28.94(46.13-17.49) | 19.07(26.93-12.49) | -1.452(-1.591--1.314) |
| Greece | Both | 1194.98(981.63-1382.98) | 868.66(710-1016.56) | -27.31 | 8.08(9.39-6.7) | 4.16(4.83-3.42) | -2.256(-2.485--2.027) |
| Greenland | Both | 3.53(2.07-5.22) | 5.35(3.2-7.62) | 51.56 | 7.51(11.14-4.45) | 7.05(10.11-4.18) | -0.213(-0.351--0.074) |
| Grenada | Both | 9.25(7.09-11.58) | 12.43(9.3-15.47) | 34.38 | 14.13(17.52-10.82) | 10.63(13.34-7.97) | -1.006(-1.397--0.614) |
| Guam | Both | 5.19(2.74-9.86) | 10.75(4.64-19.48) | 107.13 | 5.87(10.65-3.4) | 5.69(10.27-2.46) | -0.122(-0.404-0.161) |
| Guatemala | Both | 1111.41(831.37-1441.11) | 2575.83(1796.71-3472.28) | 131.76 | 24.51(31.48-18.4) | 20.41(27.58-14.25) | -0.68(-1.163--0.195) |
| Guinea | Both | 385.15(250.62-594.83) | 716.96(427.55-1145.59) | 86.15 | 11.27(17.15-7.42) | 11.96(19.08-7.2) | 0.212(0.086-0.338) |
| Guinea-Bissau | Both | 145.81(81.92-226.12) | 225.86(127.67-347.26) | 54.90 | 32.06(49.23-18.46) | 25.68(39.38-14.9) | -0.77(-0.821--0.719) |
| Guyana | Both | 146.28(114.57-177.26) | 164.62(118.7-217.52) | 12.54 | 33.56(40.85-26.24) | 23.67(30.95-17.04) | -1.121(-1.786--0.452) |
| Haiti | Both | 814.06(430.23-1179.99) | 1255.4(671.03-1921.46) | 54.21 | 22.6(32.19-12.41) | 16.19(24.85-8.72) | -1.108(-1.273--0.943) |
| Honduras | Both | 450.48(315.53-611.54) | 1322.3(790.11-2010.07) | 193.53 | 19.66(26.64-13.96) | 21.36(32.5-12.73) | 0.309(0.096-0.522) |
| Hungary | Both | 4575.57(3797.12-5249.21) | 2562.79(1947.28-3259.87) | -43.99 | 33.66(38.63-27.93) | 15.46(19.69-11.69) | -2.732(-3.255--2.206) |
| Iceland | Both | 7.06(5.5-8.89) | 11.98(9.11-14.75) | 69.69 | 2.65(3.32-2.06) | 2.35(2.9-1.8) | -0.409(-0.575--0.242) |
| India | Both | 49468.4(35179.65-70681.28) | 117264.21(77164.56-165577.27) | 137.05 | 8.94(12.74-6.45) | 9.37(13.36-6.2) | 0.2(-0.229-0.63) |
| Indonesia | Both | 8395.19(6698.18-10586.97) | 14412.81(11259.2-18250.48) | 71.68 | 7.98(9.81-6.34) | 6.77(8.36-5.37) | -0.564(-0.682--0.445) |
| Iran (Islamic Republic of) | Both | 275.63(194.78-411.16) | 1155.97(653.09-1998.67) | 319.39 | 1.06(1.51-0.76) | 1.5(2.63-0.85) | 1.155(0.664-1.648) |
| Iraq | Both | 330.13(183.89-543.46) | 346.77(178.94-608.72) | 5.04 | 3.82(6.26-2.15) | 1.35(2.34-0.73) | -3.532(-3.663--3.4) |
| Ireland | Both | 141.87(111.2-170.31) | 284.67(217.73-348.85) | 100.66 | 3.67(4.38-2.89) | 4.07(4.97-3.14) | 0.276(-0.024-0.577) |
| Israel | Both | 148.72(114.27-187.22) | 294.96(223.31-387.46) | 98.33 | 3.14(3.92-2.43) | 2.61(3.37-1.99) | -0.781(-0.992--0.57) |
| Italy | Both | 13747.11(11497.92-15496.88) | 8186.59(6250.48-9929.51) | -40.45 | 16.12(18.13-13.54) | 6(7.12-4.64) | -3.392(-3.63--3.154) |
| Jamaica | Both | 67.26(51.36-83.35) | 90.78(61.95-127.1) | 34.97 | 3.92(4.87-2.99) | 3.02(4.24-2.04) | -0.569(-1.938-0.819) |
| Japan | Both | 15814.89(12717.15-19068.35) | 13966.66(10437.56-18036.73) | -11.69 | 9.48(11.44-7.61) | 4.5(5.55-3.51) | -2.539(-2.83--2.246) |
| Jordan | Both | 25.53(13.72-44.98) | 89.17(47.39-151.13) | 249.28 | 1.77(3.07-1) | 1.26(2.09-0.71) | -1.163(-1.444--0.881) |
| Kazakhstan | Both | 1264.67(951.19-1619.77) | 3878.06(2912.7-5032.96) | 206.65 | 9.54(12.3-7.16) | 20.98(27.07-15.91) | 2.781(2.117-3.448) |
| Kenya | Both | 2971.04(1738.86-5239.88) | 6307.91(3858.76-9656.04) | 112.31 | 34.41(60.42-20.06) | 26.59(40.93-16.53) | -0.883(-0.992--0.773) |
| Kiribati | Both | 6.53(2.56-13.7) | 7.27(2.6-16.95) | 11.33 | 14.22(28.36-6.47) | 8.42(18.5-3.49) | -1.78(-1.885--1.675) |
| Kuwait | Both | 4.02(2.55-6.03) | 16.3(10.06-24.77) | 305.47 | 0.6(0.91-0.38) | 0.61(0.95-0.37) | 0.085(-0.991-1.173) |
| Kyrgyzstan | Both | 507.83(403.67-617.14) | 1293.96(985.82-1638.94) | 154.80 | 15.95(19.35-12.74) | 23.91(30.21-18.6) | 1.497(0.609-2.392) |
| Lao People's Democratic Republic | Both | 396.56(231.78-600.15) | 727.38(454.13-1064.27) | 83.42 | 16(24.1-9.48) | 13.94(20.87-8.78) | -0.479(-0.676--0.282) |
| Latvia | Both | 142.3(114.84-170.64) | 266.89(191.8-347.53) | 87.55 | 4.16(4.98-3.38) | 9.12(11.97-6.57) | 2.834(1.499-4.187) |
| Lebanon | Both | 121.94(65.04-190.86) | 129.61(49.61-236.3) | 6.29 | 5.43(8.6-2.87) | 2.51(4.56-0.97) | -2.64(-2.789--2.491) |
| Lesotho | Both | 154.07(87.2-257.22) | 216.89(120.21-335.87) | 40.77 | 14.27(23.85-8.12) | 14.85(22.92-8.49) | 0.162(0.044-0.281) |
| Liberia | Both | 379.29(256.31-519.41) | 521.52(306.36-800.06) | 37.50 | 32.89(45.15-21.96) | 21.3(32.67-12.35) | -1.514(-1.69--1.338) |
| Libya | Both | 25.36(12.79-44.14) | 88.66(38.2-174.73) | 249.61 | 1.39(2.45-0.68) | 1.61(3.1-0.74) | 0.486(-0.019-0.993) |
| Lithuania | Both | 224.69(177.04-271) | 618.56(446.62-821.27) | 175.29 | 5.19(6.25-4.13) | 14.81(19.56-10.73) | 3.673(2.774-4.579) |
| Luxembourg | Both | 80.11(66.54-90.44) | 68.9(53.26-84.66) | -13.99 | 15.6(17.62-12.99) | 7.22(8.87-5.61) | -2.617(-2.789--2.444) |
| Madagascar | Both | 909.85(574.69-1390.12) | 1328.5(766.08-2185.97) | 46.01 | 16.05(24.53-10.21) | 10.74(17.16-6.38) | -1.367(-1.486--1.248) |
| Malawi | Both | 917.38(559.41-1363.17) | 1589.99(946.63-2454.89) | 73.32 | 21.35(31.17-13.26) | 18.98(29.43-11.24) | -0.386(-0.544--0.228) |
| Malaysia | Both | 624.02(393.98-925.7) | 1430.47(789.88-2334.4) | 129.23 | 5.97(9.02-3.74) | 5.02(8.13-2.84) | -0.593(-0.847--0.339) |
| Maldives | Both | 3.26(1.76-5.16) | 7.52(4.79-12.09) | 130.67 | 3.32(5.21-1.87) | 2.13(3.25-1.4) | -1.626(-2.155--1.095) |
| Mali | Both | 648.28(409.21-1002.75) | 948.45(558.41-1602.15) | 46.30 | 14.89(22.9-9.39) | 10.2(16.68-6.13) | -1.243(-1.442--1.044) |
| Malta | Both | 23.19(17.99-28.15) | 27.12(20.37-34.43) | 16.95 | 5.44(6.6-4.25) | 3.41(4.26-2.59) | -1.583(-1.812--1.353) |
| Marshall Islands | Both | 2.42(1.18-4.14) | 3.85(1.68-7.47) | 59.09 | 11.67(19.6-6.07) | 8.62(16.46-3.96) | -1.038(-1.091--0.986) |
| Mauritania | Both | 120.48(75.08-176.34) | 131.51(76.35-207.3) | 9.16 | 11.94(17.6-7.4) | 6.22(9.59-3.68) | -2.235(-2.449--2.021) |
| Mauritius | Both | 154.35(121.06-187.88) | 141.88(96.58-191.31) | -8.08 | 18.2(22.19-14.28) | 8.06(10.83-5.5) | -2.798(-3.189--2.405) |
| Mexico | Both | 14648.68(12118.01-16912.23) | 28081.01(20852.04-36007.26) | 91.70 | 30.1(34.96-24.68) | 23.01(29.43-17.14) | -0.951(-1.17--0.731) |
| Micronesia (Federated States of) | Both | 9.38(4.93-15.57) | 7.88(3.11-14.81) | -15.99 | 16.46(26.7-9.01) | 9.14(16.95-3.9) | -2.02(-2.092--1.948) |
| Monaco | Both | 3.57(2.08-5.56) | 4.29(2.49-6.64) | 20.17 | 6.14(9.44-3.59) | 5.45(8.32-3.2) | -0.39(-0.476--0.305) |
| Mongolia | Both | 332.16(245.36-431.81) | 956.86(694.57-1284) | 188.07 | 30.14(38.77-22.41) | 38.25(50.47-27.74) | 0.837(0.721-0.952) |
| Montenegro | Both | 27.53(21.25-34.06) | 36.35(26.78-48.63) | 32.04 | 4.25(5.26-3.3) | 3.89(5.22-2.87) | -0.274(-0.438--0.109) |
| Morocco | Both | 448.35(269.28-668.7) | 534.72(318.97-850.88) | 19.26 | 3.01(4.51-1.78) | 1.67(2.58-1) | -2.047(-2.148--1.945) |
| Mozambique | Both | 316.6(204.23-462.1) | 652.29(332.46-1222.44) | 106.03 | 5.41(7.72-3.55) | 5.46(10.02-2.84) | 0.053(-0.126-0.233) |
| Myanmar | Both | 2536.11(1576.24-3851.89) | 10806.02(7071.12-15070.83) | 326.09 | 9.2(13.86-5.74) | 19.77(27.57-13.12) | 2.708(2.53-2.887) |
| Namibia | Both | 109.84(60.44-173.46) | 216.03(135.35-313.87) | 96.68 | 14.23(22.69-7.89) | 13.8(19.95-8.77) | -0.011(-0.328-0.308) |
| Nauru | Both | 0.93(0.37-1.59) | 0.84(0.36-1.45) | -9.68 | 16.12(26.96-6.9) | 13.02(22.05-6.2) | -0.723(-0.842--0.605) |
| Nepal | Both | 1541.85(1040.33-2286.5) | 3416.07(2241.97-5181.29) | 121.56 | 14.73(22.13-9.82) | 14.97(22.23-10.03) | 0.07(-0.202-0.343) |
| Netherlands | Both | 1078.88(865.2-1264.33) | 1302.6(1002.92-1581.05) | 20.74 | 5.63(6.58-4.52) | 3.94(4.73-3.08) | -1.216(-1.372--1.06) |
| New Zealand | Both | 145.48(122.91-166.94) | 210.86(168.08-251.16) | 44.94 | 3.88(4.45-3.29) | 2.82(3.34-2.26) | -1.176(-1.292--1.06) |
| Nicaragua | Both | 248.17(188.95-310.04) | 885.7(632.77-1196.28) | 256.89 | 14.52(18.32-11.02) | 18.96(25.55-13.59) | 0.899(0.065-1.739) |
| Niger | Both | 317.64(198-493.4) | 692.5(406.36-1131.89) | 118.01 | 10.7(16.67-6.7) | 8.35(13.48-4.95) | -0.843(-1.008--0.679) |
| Nigeria | Both | 12289.84(7344.93-19092.42) | 20939.6(12773.74-33467.94) | 70.38 | 26.23(40.27-15.68) | 22.24(34.52-14.16) | -0.564(-0.678--0.449) |
| Niue | Both | 0.24(0.13-0.36) | 0.17(0.08-0.25) | -29.17 | 11.48(17.4-6.19) | 7.87(11.86-3.92) | -1.308(-1.391--1.226) |
| North Macedonia | Both | 141.62(116.57-168.29) | 217.44(157.17-291.72) | 53.54 | 7.22(8.6-5.93) | 6.79(9.05-4.96) | -0.233(-0.399--0.066) |
| Northern Mariana Islands | Both | 2.97(1.23-5.99) | 3.88(1.71-7.41) | 30.64 | 10.7(20.19-5.35) | 6.82(12.85-3.15) | -1.498(-1.671--1.324) |
| Norway | Both | 206.71(168.59-248.03) | 221.81(173.54-276.29) | 7.30 | 3.53(4.2-2.89) | 2.47(3.05-1.96) | -1.274(-1.54--1.007) |
| Oman | Both | 12.28(6.51-20.17) | 28.1(14.2-50.32) | 128.83 | 1.76(2.87-0.96) | 1.49(2.35-0.87) | -0.559(-0.771--0.347) |
| Pakistan | Both | 3461.41(1795.12-6020.61) | 7481.27(4385.12-12607.67) | 116.13 | 5.78(10.16-2.96) | 5.87(9.89-3.45) | 0.029(-0.083-0.141) |
| Palau | Both | 0.82(0.33-1.71) | 1.5(0.63-2.91) | 82.93 | 7.22(14.55-3.08) | 6.31(12.01-2.8) | -0.463(-0.502--0.424) |
| Palestine | Both | 37.15(17.07-74.31) | 95.14(50.2-159.91) | 156.10 | 4.21(8.4-1.95) | 3.98(6.86-2.01) | -0.231(-0.421--0.041) |
| Panama | Both | 110.04(86.61-133.84) | 279.92(192.52-390.78) | 154.38 | 7.12(8.71-5.58) | 6.71(9.35-4.63) | -0.148(-0.496-0.202) |
| Papua New Guinea | Both | 101.17(60.28-152.21) | 189.25(99.82-321.88) | 87.06 | 3.93(5.92-2.37) | 2.83(4.71-1.57) | -1.142(-1.31--0.975) |
| Paraguay | Both | 199.77(153.33-246.61) | 511.53(335.39-720.17) | 156.06 | 8.49(10.51-6.45) | 8.79(12.42-5.78) | 0.172(-0.27-0.615) |
| Peru | Both | 1991.58(1479.21-2565.53) | 3539.76(2331.06-5195.96) | 77.74 | 15.3(19.82-11.47) | 10.88(16.03-7.16) | -1.19(-1.786--0.59) |
| Philippines | Both | 4635.16(3419.92-6205.75) | 8885(6264.73-11678.56) | 91.69 | 12.81(17.61-9.16) | 10.08(13.22-7.12) | -0.829(-0.985--0.674) |
| Poland | Both | 4206.12(3639.18-4690.51) | 6159.68(4800.08-7779.84) | 46.45 | 9.74(10.86-8.41) | 10.31(13.09-8.02) | 0.015(-0.601-0.635) |
| Portugal | Both | 2889.19(2379.02-3275.69) | 1566.52(1253.86-1845.63) | -45.78 | 22.07(25.01-18.31) | 7.82(9.15-6.31) | -3.526(-3.811--3.241) |
| Puerto Rico | Both | 671.63(509.45-833.72) | 559.78(377.29-785.22) | -16.65 | 18.79(23.27-14.25) | 9.06(12.67-6.08) | -2.504(-2.915--2.091) |
| Qatar | Both | 7.8(4.43-12.44) | 30.44(16.11-50.01) | 290.26 | 5.02(8.1-2.93) | 3.04(4.85-1.72) | -1.769(-2.865--0.661) |
| Republic of Korea | Both | 12374.63(9931.99-14451.9) | 7740.17(5986.94-9462.92) | -37.45 | 35.84(42.47-28.55) | 8.87(10.94-6.83) | -4.726(-4.878--4.574) |
| Republic of Moldova | Both | 2297.32(1820.52-2735.5) | 1862.67(1423.43-2330.03) | -18.92 | 49.61(58.99-39.44) | 33.39(41.66-25.51) | -1.306(-2.622-0.028) |
| Romania | Both | 5996.71(4929.71-6964.22) | 7390.85(5615.64-9496.91) | 23.25 | 21.19(24.62-17.51) | 22.42(28.82-16.95) | 0.114(-0.253-0.483) |
| Russian Federation | Both | 10344.15(8422.67-12298.04) | 31647.77(24201.67-39162.51) | 205.95 | 5.73(6.81-4.66) | 15.31(18.99-11.71) | 3.843(2.769-4.928) |
| Rwanda | Both | 1600.92(1108.69-2144.24) | 1667.05(1122.59-2384.77) | 4.13 | 51.18(68.93-35.4) | 25.21(35.79-17.13) | -2.423(-2.734--2.111) |
| Saint Kitts and Nevis | Both | 5.76(3.96-7.43) | 6.49(4.19-10.14) | 12.67 | 17.15(22.14-11.86) | 8.99(13.92-5.95) | -2.331(-3.033--1.624) |
| Saint Lucia | Both | 15.05(11.69-18.36) | 20.98(15.38-27.3) | 39.40 | 17.21(21.23-13.29) | 9.63(12.54-7.04) | -1.989(-2.272--1.705) |
| Saint Vincent and the Grenadines | Both | 6.63(4.97-8.43) | 12.7(9.41-16.31) | 91.55 | 9.35(11.89-7.03) | 9.3(11.91-6.91) | -0.074(-0.321-0.174) |
| Samoa | Both | 9.48(5.94-14.52) | 11.76(6.98-17.3) | 24.05 | 9.7(14.75-6.15) | 7.21(10.73-4.33) | -1.033(-1.108--0.959) |
| San Marino | Both | 3.21(1.9-4.36) | 5.52(2.74-8.59) | 71.96 | 10.17(13.78-5.95) | 8.71(13.73-4.41) | -0.504(-0.63--0.378) |
| Sao Tome and Principe | Both | 20.89(12.62-31.56) | 32.09(20.95-46.29) | 53.61 | 31.44(47.59-19.09) | 27.98(39.95-18.02) | -0.392(-0.603--0.18) |
| Saudi Arabia | Both | 317.96(104.59-694.34) | 317.52(166.09-624.67) | -0.14 | 5.29(11.29-1.89) | 1.98(3.36-1.13) | -3.365(-3.532--3.199) |
| Senegal | Both | 374.14(238.26-585.67) | 629.3(384.86-1027.21) | 68.20 | 10.64(16.63-6.83) | 7.75(12.46-4.75) | -0.909(-1.274--0.543) |
| Serbia | Both | 1089.82(861.53-1345.46) | 992.68(707.1-1330.24) | -8.91 | 9.15(11.25-7.23) | 6.87(9.21-4.92) | -0.984(-1.363--0.604) |
| Seychelles | Both | 9.02(6.25-12.03) | 20.81(15.04-26.71) | 130.71 | 16.21(21.71-11.21) | 17.2(22.09-12.45) | 0.227(0.014-0.442) |
| Sierra Leone | Both | 587.1(395.87-852) | 636.54(393.14-959.78) | 8.42 | 29.51(42.91-19.84) | 15.62(23.78-9.77) | -2.156(-2.411--1.901) |
| Singapore | Both | 59.33(40.35-79.47) | 72.29(47.29-102.6) | 21.84 | 2.38(3.24-1.6) | 0.89(1.26-0.59) | -3.339(-3.686--2.991) |
| Slovakia | Both | 1225.59(1006.51-1441.77) | 1235.33(906.65-1630.29) | 0.79 | 21.1(24.82-17.23) | 14.52(19.24-10.61) | -1.374(-1.671--1.075) |
| Slovenia | Both | 572.81(390.19-783.75) | 360.32(231.88-534.73) | -37.10 | 23.63(32.28-16.13) | 9.43(13.99-6.12) | -3.055(-3.498--2.609) |
| Solomon Islands | Both | 13.54(8.12-21.03) | 33.52(20.22-51.46) | 147.56 | 7.56(11.78-4.61) | 7.54(11.39-4.78) | -0.015(-0.155-0.126) |
| Somalia | Both | 320.6(183.07-506.7) | 708.7(408.8-1150.96) | 121.05 | 12.1(18.77-7.09) | 10.11(16.28-5.91) | -0.601(-0.741--0.461) |
| South Africa | Both | 3149.45(2284.25-4371.19) | 3809.79(2945.68-4815.64) | 20.97 | 13.16(18.55-9.45) | 7.97(10.13-6.06) | -1.732(-2.198--1.264) |
| South Sudan | Both | 243.69(144.34-420.74) | 332.31(192.57-557.23) | 36.37 | 10.08(17.28-6.03) | 8.64(14.51-5.15) | -0.536(-0.63--0.443) |
| Spain | Both | 8669.27(6940.95-10182.92) | 5490.05(4125.52-6924.09) | -36.67 | 16.94(19.86-13.66) | 6.18(7.63-4.74) | -3.418(-3.586--3.25) |
| Sri Lanka | Both | 1701.57(1150.85-2374.68) | 2676.34(1774.79-3834.52) | 57.29 | 13.41(18.84-8.8) | 10.56(15.08-7.08) | -0.707(-1.286--0.125) |
| Sudan | Both | 472.05(184.27-940.68) | 276.1(141.41-505.17) | -41.51 | 4.76(9.45-1.85) | 1.56(2.86-0.79) | -3.825(-4.017--3.632) |
| Suriname | Both | 41.33(31.3-52.04) | 72.88(51.92-97.96) | 76.34 | 14.91(18.67-11.23) | 11.76(15.7-8.34) | -0.764(-1.361--0.163) |
| Sweden | Both | 615.9(504.86-722.3) | 793.75(639.53-949.84) | 28.88 | 4.7(5.51-3.86) | 4.17(4.95-3.39) | -0.418(-0.646--0.189) |
| Switzerland | Both | 722.07(596.63-819.68) | 820.1(646.11-1020.06) | 13.58 | 7.57(8.55-6.23) | 5.01(6.11-3.97) | -1.488(-1.593--1.384) |
| Syrian Arab Republic | Both | 273.01(145.8-476.57) | 316.65(134.05-583.19) | 15.98 | 4.89(8.55-2.64) | 2.64(4.86-1.22) | -2.139(-2.481--1.797) |
| Taiwan (Province of China) | Both | 3689.14(2976.52-4414.89) | 3889.92(2681.36-5385.18) | 5.44 | 22.57(27.49-17.93) | 10.46(14.43-7.23) | -2.568(-2.856--2.279) |
| Tajikistan | Both | 417.85(319.67-530.15) | 999.35(731.62-1344.32) | 139.16 | 13.57(17.23-10.31) | 16.94(22.67-12.49) | 0.776(0.435-1.118) |
| Thailand | Both | 6259.44(4796.03-7885.35) | 12730.13(8761.38-17833.24) | 103.37 | 14.71(18.62-11.23) | 12.72(17.79-8.77) | -0.454(-0.629--0.278) |
| Timor-Leste | Both | 26.62(12.71-51.37) | 97.3(47.37-165.8) | 265.51 | 6.84(12.81-3.35) | 11.31(19.33-5.58) | 1.769(1.511-2.027) |
| Togo | Both | 328.43(219.14-477.48) | 580.81(337.99-928.32) | 76.84 | 23.27(33.45-15.23) | 13.4(20.9-8.06) | -1.885(-2.108--1.662) |
| Tokelau | Both | 0.1(0.05-0.15) | 0.08(0.05-0.12) | -20.00 | 7.7(11.91-4.3) | 6.11(9.2-3.47) | -0.784(-0.86--0.709) |
| Tonga | Both | 3.57(2.19-5.48) | 5.05(2.96-8.08) | 41.46 | 6.04(9.17-3.75) | 6.15(9.8-3.62) | 0.081(-0.157-0.319) |
| Trinidad and Tobago | Both | 86.54(66.73-107.74) | 115.16(75.48-165.05) | 33.07 | 9.74(12.16-7.49) | 6.24(8.88-4.1) | -1.474(-2.152--0.792) |
| Tunisia | Both | 112.4(63.31-177.78) | 322.41(167.47-557.48) | 186.84 | 2.07(3.27-1.18) | 2.53(4.38-1.34) | 0.704(0.608-0.801) |
| Turkey | Both | 1209.11(687.15-1979.68) | 1727.32(1034.31-2679.73) | 42.86 | 3.05(4.95-1.73) | 1.92(3-1.15) | -1.641(-1.926--1.356) |
| Turkmenistan | Both | 320.77(244.1-402.79) | 1405.89(1011.03-1862.88) | 338.29 | 15.24(19.08-11.61) | 30.03(39.54-21.76) | 2.418(1.616-3.228) |
| Tuvalu | Both | 0.68(0.35-1.12) | 0.71(0.37-1.2) | 4.41 | 9.01(14.61-4.86) | 6.57(11.06-3.47) | -1.077(-1.122--1.032) |
| Uganda | Both | 1941.77(1365.46-2702.27) | 3571.22(2389.59-4886.67) | 83.92 | 28.36(39.41-19.79) | 22.97(31.33-15.31) | -0.731(-0.832--0.63) |
| Ukraine | Both | 4805.33(3778.71-5876.1) | 14243.5(10457.84-18157.21) | 196.41 | 7.01(8.56-5.53) | 23.29(29.6-17.24) | 4.504(2.241-6.818) |
| United Arab Emirates | Both | 34.24(20.49-54.67) | 141.21(63.32-262.22) | 312.41 | 6.45(10.13-3.59) | 2.38(4.35-1.1) | -3.431(-4.041--2.817) |
| United Kingdom | Both | 3162.11(2693.21-3619.58) | 6787.76(5685.2-7930.83) | 114.66 | 4.06(4.65-3.43) | 6.6(7.7-5.56) | 1.751(1.492-2.011) |
| United Republic of Tanzania | Both | 3344.22(2347.68-4389.53) | 5373.51(3390.28-7788.19) | 60.68 | 29.13(38.74-20.33) | 20.39(29.73-12.8) | -1.2(-1.371--1.029) |
| United States of America | Both | 23653.61(18388-28931.83) | 45364.04(35902.4-55165.42) | 91.78 | 8.01(9.78-6.28) | 9.02(10.88-7.19) | 0.439(0.264-0.614) |
| United States Virgin Islands | Both | 10.9(6.53-16.38) | 18.72(11.72-28.25) | 71.74 | 11.88(18.04-7.16) | 10.71(16.24-6.62) | -0.349(-0.7-0.003) |
| Uruguay | Both | 368.97(290.82-446.74) | 329.64(252.14-410.32) | -10.66 | 9.78(11.81-7.7) | 6.43(8.01-4.94) | -1.515(-1.823--1.206) |
| Uzbekistan | Both | 1694.29(1300.09-2100.17) | 6608.03(4893.95-8595.94) | 290.02 | 13.78(17.15-10.65) | 25.53(32.55-19.44) | 2.212(1.837-2.588) |
| Vanuatu | Both | 9.73(5.42-15.56) | 17.15(8.88-29.29) | 76.26 | 11.77(18.88-6.8) | 8.41(14.04-4.42) | -1.164(-1.411--0.916) |
| Venezuela (Bolivarian Republic of) | Both | 1380.91(1104.61-1633.15) | 2908.48(1912.78-4045.52) | 110.62 | 13.03(15.44-10.36) | 9.6(13.36-6.38) | -1.164(-2.403-0.09) |
| Viet Nam | Both | 2856.55(1754.87-4366.14) | 15277.79(10680.18-20112.23) | 434.83 | 7.09(10.75-4.38) | 15.41(20.11-10.75) | 2.736(2.521-2.951) |
| Yemen | Both | 383.02(180.64-678.36) | 371.66(178.91-674.87) | -2.97 | 7.18(13.43-3.29) | 2.52(4.47-1.24) | -3.548(-3.661--3.434) |
| Zambia | Both | 1132.71(712.49-1611.33) | 2509.04(1526.55-3700.4) | 121.51 | 35.56(51.63-22.4) | 31.55(46.58-18.67) | -0.403(-0.564--0.242) |
| Zimbabwe | Both | 765.87(532.69-1032.7) | 988.73(549.61-1579.59) | 29.10 | 16.59(22.27-11.36) | 11.95(19.23-6.82) | -1.102(-1.455--0.748) |
| Afghanistan | Female | 51.55(23.71-89.32) | 81.81(36.32-148.69) | 58.70 | 1.53(2.53-0.72) | 1.32(2.25-0.66) | -0.466(-0.582--0.35) |
| Albania | Female | 36.29(27.7-44.06) | 46.12(30.79-65.23) | 27.09 | 3.41(4.19-2.56) | 2.06(2.9-1.39) | -1.743(-2.17--1.315) |
| Algeria | Female | 44.39(26.46-71.02) | 89.86(55.19-140.67) | 102.43 | 0.83(1.27-0.49) | 0.61(0.94-0.38) | -1.02(-1.133--0.908) |
| American Samoa | Female | 0.15(0.1-0.22) | 0.3(0.19-0.45) | 100.00 | 1.33(1.94-0.86) | 1.22(1.81-0.77) | -0.299(-0.579--0.017) |
| Andorra | Female | 1.21(0.63-2.03) | 2.54(1.55-3.88) | 109.92 | 4.62(7.81-2.41) | 3.56(5.49-2.21) | -0.889(-0.978--0.8) |
| Angola | Female | 150.89(78.13-266.99) | 967.18(592.41-1473.49) | 540.98 | 6.72(12.08-3.51) | 14.21(21.96-8.81) | 2.665(2.442-2.888) |
| Antigua and Barbuda | Female | 0.83(0.62-1.09) | 1.77(1.22-2.45) | 113.25 | 2.78(3.7-2.09) | 3.29(4.55-2.28) | 0.599(0.291-0.909) |
| Argentina | Female | 1139.78(871.23-1418.12) | 1340.22(913.35-1838.05) | 17.59 | 6.5(8.07-4.99) | 4.44(6.02-3.04) | -1.28(-1.757--0.801) |
| Armenia | Female | 52.22(39.14-67.58) | 170.8(122.63-227.62) | 227.08 | 3.43(4.43-2.57) | 7.12(9.5-5.19) | 2.611(1.759-3.471) |
| Australia | Female | 291(227.16-356.8) | 458.68(333.74-600.87) | 57.62 | 2.85(3.49-2.23) | 2.23(2.86-1.64) | -0.804(-1.031--0.577) |
| Austria | Female | 491.48(377.26-603.72) | 371.98(269.23-482.38) | -24.31 | 7.95(9.71-6.14) | 4.16(5.26-3.09) | -2.283(-2.673--1.89) |
| Azerbaijan | Female | 286.65(196.45-423.25) | 530.18(331.05-851.36) | 84.96 | 9.56(14.21-6.56) | 11.79(18.65-7.12) | 0.746(0.529-0.963) |
| Bahamas | Female | 6.36(4.3-8.98) | 7.05(4.66-10.48) | 10.85 | 6.91(9.8-4.64) | 3.23(4.74-2.13) | -2.635(-2.978--2.291) |
| Bahrain | Female | 3.25(1.75-5.44) | 2.48(1.31-4.3) | -23.69 | 4.19(7.19-2.22) | 0.8(1.34-0.45) | -5.604(-6.028--5.179) |
| Bangladesh | Female | 953.38(565.4-1464.56) | 1589.77(962.83-2483.98) | 66.75 | 4.17(6.45-2.46) | 2.44(3.81-1.48) | -1.673(-1.994--1.352) |
| Barbados | Female | 3.29(2.41-4.33) | 4.72(3.18-6.72) | 43.47 | 2.09(2.7-1.53) | 1.79(2.54-1.21) | -0.488(-0.875--0.1) |
| Belarus | Female | 168.56(119.3-228.69) | 508.78(331.44-739.54) | 201.84 | 2.21(2.97-1.56) | 6.17(8.95-3.89) | 3.615(2.657-4.583) |
| Belgium | Female | 566.52(440.85-680.75) | 595.68(443.89-760.73) | 5.15 | 6.85(8.09-5.42) | 5.06(6.32-3.88) | -1.075(-1.402--0.746) |
| Belize | Female | 2.41(1.82-3.12) | 6.5(4.68-8.64) | 169.71 | 5.04(6.54-3.83) | 4.42(5.84-3.22) | -0.401(-0.831-0.031) |
| Benin | Female | 78.43(49.57-123.81) | 190.41(110.68-315.95) | 142.78 | 7.21(11.39-4.56) | 6.56(10.62-3.92) | -0.307(-0.382--0.232) |
| Bermuda | Female | 1.61(1.08-2.25) | 0.85(0.52-1.37) | -47.20 | 4.59(6.44-3.09) | 1.22(1.88-0.74) | -4.441(-4.697--4.184) |
| Bhutan | Female | 5.08(2.88-7.91) | 8.92(5.53-13.96) | 75.59 | 3.77(5.79-2.16) | 3.27(5.15-1.99) | -0.526(-0.842--0.209) |
| Bolivia (Plurinational State of) | Female | 166.71(93.06-259.72) | 415.36(266.47-603.36) | 149.15 | 9.55(14.67-5.44) | 9.12(13.16-6) | -0.147(-0.266--0.028) |
| Bosnia and Herzegovina | Female | 86.44(66.1-107.35) | 75.71(53.08-105.84) | -12.41 | 3.7(4.6-2.86) | 2.32(3.26-1.65) | -1.601(-2.236--0.962) |
| Botswana | Female | 23.29(10.72-42.65) | 44.07(21.19-76.82) | 89.22 | 6.42(11.58-3.02) | 4.85(8.49-2.41) | -0.977(-1.331--0.622) |
| Brazil | Female | 1963.59(1579.7-2480.88) | 3650.01(2847.02-4712.82) | 85.88 | 3.8(4.77-3.1) | 2.81(3.61-2.2) | -1.034(-1.252--0.815) |
| Brunei Darussalam | Female | 1.3(0.8-2.01) | 2.49(1.64-3.61) | 91.54 | 2.3(3.47-1.41) | 1.53(2.17-1.02) | -1.392(-1.487--1.296) |
| Bulgaria | Female | 278.67(214.13-353.94) | 337.01(230.42-470.93) | 20.94 | 4.3(5.42-3.36) | 5.01(7.05-3.39) | 0.56(0.289-0.832) |
| Burkina Faso | Female | 376.14(230.98-548.69) | 625.36(219.78-1089.56) | 66.26 | 16.7(24.54-10.19) | 12.11(21.25-4.06) | -1.052(-1.291--0.813) |
| Burundi | Female | 271.75(139.13-470.83) | 192.33(83.21-364.41) | -29.23 | 20.54(35.63-10.78) | 8.19(15.66-3.48) | -3.143(-3.346--2.938) |
| Cabo Verde | Female | 9.16(5.09-15.35) | 14.88(8.35-23.67) | 62.45 | 7(11.56-3.95) | 5.99(9.54-3.36) | -0.537(-0.728--0.345) |
| Cambodia | Female | 287.95(189.41-414.96) | 1026.09(589.38-1719.68) | 256.34 | 10.57(15.26-6.99) | 14.25(23.83-8.31) | 1.047(0.843-1.251) |
| Cameroon | Female | 360.71(219.87-546.19) | 679.88(319.48-1190.05) | 88.48 | 14.87(22.76-9.08) | 10.01(17.74-4.66) | -1.345(-1.444--1.246) |
| Canada | Female | 651.64(475.94-855.77) | 1232.94(862.69-1684.58) | 89.21 | 3.71(4.86-2.75) | 3.54(4.69-2.58) | -0.153(-0.366-0.06) |
| Central African Republic | Female | 60.3(31.22-104.89) | 84.42(38.75-169.67) | 40.00 | 8.43(14.84-4.26) | 6.26(12.42-2.97) | -1.044(-1.267--0.82) |
| Chad | Female | 121.3(57.42-230.94) | 336.99(133.23-596.84) | 177.82 | 7.98(14.81-3.9) | 11.35(19.73-4.78) | 1.288(1.209-1.366) |
| Chile | Female | 702.78(516.42-910.63) | 923(638.9-1244.06) | 31.34 | 12.64(16.36-9.31) | 7.05(9.48-4.88) | -2.078(-2.64--1.513) |
| China | Female | 9926.42(6677.78-14362.99) | 10436.56(6397.44-16664.84) | 5.14 | 2.34(3.37-1.57) | 1.02(1.64-0.62) | -2.836(-3.041--2.63) |
| Colombia | Female | 256.22(199.78-323.51) | 508.32(346.01-716.78) | 98.39 | 2.74(3.41-2.14) | 1.74(2.47-1.18) | -1.596(-1.874--1.318) |
| Comoros | Female | 5.32(2.3-9.22) | 11.42(5.19-18.99) | 114.66 | 4.72(8.15-2.13) | 4.37(7.36-1.97) | -0.229(-0.556-0.099) |
| Congo | Female | 64.97(33.95-105.11) | 161.82(73.99-294.18) | 149.07 | 10.38(17.02-5.44) | 10.89(20.41-4.89) | 0.19(-0.134-0.515) |
| Cook Islands | Female | 0.04(0.02-0.06) | 0.06(0.04-0.1) | 50.00 | 0.61(0.94-0.37) | 0.53(0.84-0.31) | -0.495(-0.586--0.404) |
| Costa Rica | Female | 36.08(27.43-45.66) | 112.67(77.06-155.04) | 212.28 | 3.9(4.95-2.95) | 4.04(5.59-2.76) | 0.182(-0.546-0.916) |
| Croatia | Female | 319.43(232.95-414.5) | 141.09(98.9-197.37) | -55.83 | 8.79(11.4-6.48) | 3.11(4.44-2.16) | -3.528(-4.101--2.952) |
| Cuba | Female | 154.1(113.17-201.57) | 208.57(139.4-300.1) | 35.35 | 2.94(3.82-2.17) | 2.13(3.1-1.43) | -0.955(-1.365--0.544) |
| Cyprus | Female | 17.46(10.76-28.53) | 21(14.09-30.49) | 20.27 | 4.71(7.88-2.87) | 2.2(3.22-1.5) | -2.598(-2.935--2.26) |
| Czechia | Female | 447.51(347.33-560.04) | 502.24(363.09-675.52) | 12.23 | 5.95(7.33-4.66) | 5.26(7.11-3.81) | -0.403(-0.738--0.067) |
| C么te d'Ivoire | Female | 160.63(87.27-272.57) | 405.62(193.89-725.67) | 152.52 | 7.81(12.91-4.26) | 7.33(13.22-3.53) | -0.201(-0.285--0.117) |
| Democratic People's Republic of Korea | Female | 227.26(117.44-378.63) | 301.97(159.83-515.62) | 32.87 | 2.32(3.8-1.21) | 1.62(2.76-0.86) | -1.23(-1.335--1.124) |
| Democratic Republic of the Congo | Female | 504.43(269.72-863.23) | 711.83(387.21-1304.85) | 41.12 | 5.34(9.15-2.85) | 3.31(6.2-1.82) | -1.639(-1.935--1.343) |
| Denmark | Female | 185.24(148.8-219.36) | 234.9(176-299.93) | 26.81 | 5.08(6.04-4.1) | 4.33(5.48-3.28) | -0.498(-0.829--0.167) |
| Djibouti | Female | 3.24(1.88-5.23) | 10.08(4.89-18.31) | 211.11 | 4.77(7.81-2.83) | 3.84(6.94-1.92) | -0.667(-0.817--0.516) |
| Dominica | Female | 2.03(1.37-2.95) | 1.53(0.97-2.28) | -24.63 | 5.25(7.57-3.5) | 3.43(5.15-2.2) | -1.549(-1.632--1.465) |
| Dominican Republic | Female | 193.21(142.19-249.33) | 374.69(243.94-545.19) | 93.93 | 9.45(12.21-6.89) | 7.68(11.11-5.04) | -0.765(-1.268--0.259) |
| Ecuador | Female | 133.5(99.67-178.24) | 468.66(325.26-661.68) | 251.06 | 4.91(6.7-3.64) | 6.08(8.57-4.2) | 0.755(0.466-1.044) |
| Egypt | Female | 642.23(397.69-998.1) | 1106.15(582.47-1958.2) | 72.24 | 5.46(8.33-3.32) | 5.35(8.95-2.87) | -0.059(-0.408-0.291) |
| El Salvador | Female | 81.18(60.43-105.1) | 163.84(105.42-231.07) | 101.82 | 5.02(6.56-3.71) | 4.79(6.76-3.08) | -0.143(-0.469-0.185) |
| Equatorial Guinea | Female | 5.88(2.61-11.54) | 24.03(11.61-41.64) | 308.67 | 4.83(9.48-2.16) | 7.95(13.79-4) | 1.78(1.385-2.176) |
| Eritrea | Female | 36.36(17.07-65.01) | 109.89(65.48-172.63) | 202.23 | 6.3(11.05-3.05) | 7.32(11.56-4.5) | 0.537(0.284-0.791) |
| Estonia | Female | 23.81(17.09-32.28) | 51.66(33.42-73.08) | 116.97 | 2.07(2.78-1.49) | 4.75(6.72-3.1) | 2.895(1.116-4.704) |
| Eswatini | Female | 14.29(8.02-23.71) | 22.53(10.37-40.84) | 57.66 | 7.88(12.99-4.42) | 6.21(11.23-2.87) | -0.853(-1.204--0.501) |
| Ethiopia | Female | 1011.29(522.5-1891.07) | 1637.81(927.76-2742.43) | 61.95 | 9.92(17.99-5.37) | 7.89(12.87-4.68) | -0.736(-0.925--0.547) |
| Fiji | Female | 2.57(1.59-3.74) | 4.78(2.93-7.47) | 85.99 | 1.32(1.94-0.83) | 1.21(1.86-0.76) | -0.316(-0.582--0.05) |
| Finland | Female | 106.29(80.45-138.11) | 186.63(133.67-245.99) | 75.59 | 2.87(3.69-2.18) | 3.77(4.88-2.71) | 0.856(0.382-1.332) |
| France | Female | 3394.08(2622.6-4043.75) | 2514.56(1859.61-3156.9) | -25.91 | 8.03(9.47-6.34) | 3.62(4.47-2.72) | -2.675(-2.98--2.37) |
| Gabon | Female | 44.66(27.82-69.38) | 62.34(34.51-101.78) | 39.59 | 14.48(22.83-8.79) | 10.78(18.01-5.75) | -1.024(-1.261--0.787) |
| Gambia | Female | 9.63(5.15-15.4) | 35.23(18.52-66.31) | 265.84 | 5.59(8.89-3.04) | 6.72(12.56-3.51) | 0.719(-0.514-1.966) |
| Georgia | Female | 192.4(147.68-239.43) | 141.28(101.35-187.66) | -26.57 | 5.19(6.41-4.01) | 3.98(5.27-2.91) | -0.922(-1.767--0.069) |
| Germany | Female | 6577.44(5311.08-7522.7) | 5654.72(4376.21-6900.53) | -14.03 | 9.71(11-7.91) | 6.02(7.2-4.76) | -1.651(-1.909--1.392) |
| Ghana | Female | 436.35(226.87-754.74) | 666.54(337.96-1107.34) | 52.75 | 12.4(21.56-6.53) | 7(11.63-3.61) | -1.953(-2.119--1.785) |
| Greece | Female | 272.91(193.01-368.95) | 166.28(119.91-225.27) | -39.07 | 3.34(4.5-2.41) | 1.33(1.71-0.98) | -3.13(-3.37--2.89) |
| Greenland | Female | 1.56(0.83-2.43) | 2.01(1.06-3.09) | 28.85 | 7.19(11.28-3.76) | 5.99(9.21-3.2) | -0.596(-0.736--0.457) |
| Grenada | Female | 2.14(1.52-3.01) | 2.02(1.35-2.89) | -5.61 | 5.53(7.73-3.9) | 3.47(4.95-2.33) | -1.573(-1.953--1.191) |
| Guam | Female | 0.66(0.43-0.96) | 1.21(0.79-1.81) | 83.33 | 1.94(2.86-1.28) | 1.23(1.84-0.8) | -1.566(-2.24--0.887) |
| Guatemala | Female | 226.35(162.47-301.36) | 541.58(373.03-742.69) | 139.27 | 10.67(14.08-7.83) | 8.71(11.93-6) | -0.668(-1.265--0.067) |
| Guinea | Female | 100.86(58.92-161.19) | 137.92(72.24-239.51) | 36.74 | 6.13(9.78-3.59) | 4.94(8.6-2.61) | -0.745(-0.905--0.584) |
| Guinea-Bissau | Female | 31.22(16.31-55.61) | 52.96(27.88-91.87) | 69.63 | 13.2(22.76-6.98) | 11.45(19.39-6.15) | -0.525(-0.662--0.389) |
| Guyana | Female | 16.36(11.94-21.34) | 18.58(12.79-25.84) | 13.57 | 7.89(10.24-5.75) | 5.33(7.37-3.68) | -1.125(-2.113--0.126) |
| Haiti | Female | 189.57(95.6-305.44) | 287.92(127.35-519.44) | 51.88 | 10.48(16.67-5.6) | 7.17(12.59-3.22) | -1.279(-1.428--1.129) |
| Honduras | Female | 113.94(81.18-153.34) | 428.13(239.73-671.34) | 275.75 | 10.11(13.55-7.16) | 13.37(20.93-7.65) | 1.037(0.417-1.66) |
| Hungary | Female | 1043.08(774.65-1325.96) | 490.79(346.1-692.74) | -52.95 | 13.93(17.76-10.34) | 5.2(7.31-3.6) | -3.409(-3.948--2.867) |
| Iceland | Female | 1.95(1.45-2.57) | 2.83(1.95-3.84) | 45.13 | 1.43(1.88-1.06) | 1.07(1.46-0.73) | -0.972(-1.09--0.854) |
| India | Female | 6861.37(4994.19-9800.83) | 14507.8(10287.34-20215.32) | 111.44 | 3.07(4.31-2.25) | 2.46(3.43-1.76) | -0.684(-1.413-0.05) |
| Indonesia | Female | 3138.63(2387.61-3983.51) | 5254.06(3875.36-6930.16) | 67.40 | 6(7.69-4.56) | 4.97(6.48-3.7) | -0.637(-0.738--0.536) |
| Iran (Islamic Republic of) | Female | 65.1(45.67-91.59) | 172.98(115.1-312.18) | 165.71 | 0.59(0.87-0.4) | 0.5(0.89-0.33) | -0.624(-0.882--0.365) |
| Iraq | Female | 39.81(22.34-68.29) | 52.54(31.39-82.1) | 31.98 | 0.92(1.57-0.51) | 0.45(0.69-0.28) | -2.404(-2.619--2.189) |
| Ireland | Female | 53.65(39.23-69.34) | 94.69(68.02-124.22) | 76.50 | 2.58(3.28-1.91) | 2.57(3.35-1.87) | 0.053(-0.308-0.415) |
| Israel | Female | 47.82(34.47-63.36) | 92.38(67.34-125.37) | 93.18 | 1.85(2.42-1.35) | 1.43(1.91-1.05) | -0.963(-1.241--0.684) |
| Italy | Female | 4503.12(3458.62-5511.52) | 2879.94(1874.22-3982.67) | -36.05 | 9.06(10.97-7.07) | 3.35(4.51-2.28) | -3.362(-3.643--3.08) |
| Jamaica | Female | 14.17(10.74-18.15) | 20.7(14.27-29.34) | 46.08 | 1.51(1.95-1.13) | 1.31(1.86-0.89) | -0.493(-1.433-0.457) |
| Japan | Female | 4268.73(3146.34-5567.13) | 4467.4(2790.78-6613.69) | 4.65 | 4.58(5.94-3.37) | 2.08(2.87-1.45) | -2.695(-3.11--2.279) |
| Jordan | Female | 5.54(3.34-8.9) | 14.3(8.39-23.35) | 158.12 | 0.93(1.44-0.55) | 0.52(0.84-0.3) | -1.961(-2.373--1.547) |
| Kazakhstan | Female | 455.91(327.64-627.78) | 1149.27(867.05-1523.03) | 152.08 | 5.86(8.07-4.24) | 11.18(14.6-8.51) | 2.266(1.82-2.713) |
| Kenya | Female | 726.42(386.65-1281.37) | 1426.83(726.59-2518.12) | 96.42 | 17.14(29.56-9.24) | 12.47(21.78-6.44) | -1.088(-1.218--0.958) |
| Kiribati | Female | 0.94(0.55-1.48) | 1.24(0.65-2.03) | 31.91 | 4.56(7.4-2.71) | 3.2(5-1.84) | -1.197(-1.35--1.043) |
| Kuwait | Female | 0.76(0.47-1.15) | 2.61(1.6-4.08) | 243.42 | 0.34(0.53-0.21) | 0.27(0.42-0.16) | -0.845(-1.397--0.29) |
| Kyrgyzstan | Female | 147.65(114.07-181.2) | 276.92(212.57-362.99) | 87.55 | 8.14(9.99-6.22) | 10.07(13-7.78) | 0.711(0.052-1.375) |
| Lao People's Democratic Republic | Female | 78(38.99-138.33) | 135.23(76.79-222.16) | 73.37 | 6.19(10.84-3.24) | 5.25(8.61-3) | -0.566(-0.77--0.361) |
| Latvia | Female | 41.97(31.09-54.01) | 75.61(49.9-111.66) | 80.15 | 2.05(2.63-1.53) | 4.59(6.84-2.9) | 2.783(1.635-3.944) |
| Lebanon | Female | 19.68(9.17-38.09) | 16.46(7.76-30.49) | -16.36 | 1.82(3.63-0.82) | 0.58(1.07-0.27) | -3.869(-3.954--3.783) |
| Lesotho | Female | 25.74(11.02-48.81) | 42.22(17.63-77.02) | 64.02 | 4.43(8.28-1.93) | 5.29(9.49-2.28) | 0.526(0.155-0.899) |
| Liberia | Female | 98.27(57.43-154.27) | 138.04(78.15-224.65) | 40.47 | 18.29(29.26-10.55) | 11.7(18.84-6.6) | -1.561(-1.807--1.314) |
| Libya | Female | 7.14(3.65-12.7) | 16.16(9.57-25.93) | 126.33 | 0.85(1.55-0.42) | 0.67(1.07-0.38) | -0.82(-1.148--0.491) |
| Lithuania | Female | 64.05(44.87-84.68) | 188.52(128.86-265.28) | 194.33 | 2.57(3.43-1.81) | 7.9(11.03-5.49) | 3.774(3.111-4.441) |
| Luxembourg | Female | 24.98(19.21-30.55) | 20.33(14.52-26.41) | -18.61 | 8.62(10.43-6.65) | 3.95(5.13-2.86) | -2.673(-3.163--2.18) |
| Madagascar | Female | 173.85(106.39-281.86) | 316.97(187.45-502.58) | 82.32 | 6.53(10.52-4.02) | 5.49(8.62-3.37) | -0.564(-0.896--0.231) |
| Malawi | Female | 177.43(106.57-275.97) | 274.45(149.46-487.67) | 54.68 | 8.52(13.14-5.14) | 6.63(11.63-3.56) | -0.825(-1.114--0.535) |
| Malaysia | Female | 67.7(42.54-108.17) | 218.85(128.58-341.56) | 223.26 | 1.43(2.29-0.9) | 1.71(2.59-1.02) | 0.638(0.116-1.164) |
| Maldives | Female | 1.03(0.48-1.77) | 1.53(1.04-2.21) | 48.54 | 2.64(4.34-1.33) | 1.18(1.74-0.77) | -2.87(-3.293--2.445) |
| Mali | Female | 211.64(120.23-360.94) | 313.99(152.24-568.58) | 48.36 | 9.68(16.41-5.58) | 6.92(12.24-3.39) | -1.135(-1.288--0.982) |
| Malta | Female | 5.73(4.25-7.6) | 6.6(4.45-9.52) | 15.18 | 2.46(3.26-1.83) | 1.55(2.18-1.08) | -1.598(-1.932--1.263) |
| Marshall Islands | Female | 0.24(0.14-0.37) | 0.42(0.18-0.81) | 75.00 | 2.81(4.19-1.73) | 2.35(4.27-1.08) | -0.621(-0.675--0.566) |
| Mauritania | Female | 37.79(23.96-56.89) | 39.63(23.37-60.44) | 4.87 | 7.3(10.91-4.65) | 3.88(5.95-2.29) | -2.148(-2.298--1.998) |
| Mauritius | Female | 7.57(5.38-10.34) | 11.24(7.44-16.22) | 48.48 | 1.85(2.55-1.3) | 1.2(1.72-0.8) | -1.508(-1.957--1.058) |
| Mexico | Female | 2129.77(1765.59-2620.88) | 4824.36(3547.96-6661.24) | 126.52 | 9.17(11.26-7.63) | 7.68(10.57-5.68) | -0.61(-0.961--0.257) |
| Micronesia (Federated States of) | Female | 0.75(0.44-1.22) | 0.83(0.37-1.54) | 10.67 | 3.17(5-1.92) | 2.22(3.92-1.05) | -1.236(-1.43--1.042) |
| Monaco | Female | 1.05(0.55-1.93) | 1.39(0.72-2.49) | 32.38 | 3.01(5.31-1.64) | 3.08(5.39-1.61) | 0.083(0.004-0.162) |
| Mongolia | Female | 103.78(72.91-136.51) | 282.05(194.8-384.77) | 171.78 | 18.13(23.89-12.78) | 23.04(31.09-15.81) | 0.821(0.664-0.98) |
| Montenegro | Female | 5.09(3.11-7.36) | 5.66(3.91-7.7) | 11.20 | 1.48(2.13-0.91) | 1.15(1.58-0.79) | -0.838(-1.02--0.656) |
| Morocco | Female | 54.17(30.73-88.23) | 99.09(59.03-157.53) | 82.92 | 0.84(1.39-0.45) | 0.67(1.04-0.4) | -0.771(-0.844--0.699) |
| Mozambique | Female | 102.13(60.2-164.21) | 167.65(52.35-292.3) | 64.15 | 3.45(5.51-2.04) | 2.87(5.05-0.92) | -0.591(-0.696--0.487) |
| Myanmar | Female | 350.12(202.34-552.9) | 874.78(567.49-1290.15) | 149.85 | 2.55(4.04-1.51) | 3.07(4.47-2.01) | 0.696(0.195-1.199) |
| Namibia | Female | 18.76(8.86-34.57) | 45.68(21.26-84.98) | 143.50 | 4.51(8.21-2.18) | 5.16(9.41-2.39) | 0.49(0.222-0.759) |
| Nauru | Female | 0.05(0.03-0.09) | 0.06(0.03-0.12) | 20.00 | 2.71(4.47-1.45) | 2.37(4.15-1.16) | -0.487(-0.609--0.364) |
| Nepal | Female | 412.19(269.13-587.26) | 872.4(567.53-1294.87) | 111.65 | 8.38(12.16-5.44) | 7.48(10.99-4.95) | -0.392(-0.592--0.192) |
| Netherlands | Female | 447.77(332.25-564.91) | 517.26(361.28-682.2) | 15.52 | 3.9(4.85-2.95) | 2.67(3.43-1.96) | -1.296(-1.585--1.005) |
| New Zealand | Female | 52.68(42.46-63.76) | 69.71(51.6-90.14) | 32.33 | 2.53(3.03-2.05) | 1.68(2.13-1.27) | -1.435(-1.578--1.291) |
| Nicaragua | Female | 32.34(24.55-41.45) | 138.23(100.96-183.61) | 327.43 | 3.78(4.82-2.84) | 5.79(7.68-4.27) | 1.496(0.7-2.298) |
| Niger | Female | 78.02(48.52-119.61) | 195.33(106.01-306.9) | 150.36 | 5.8(8.97-3.54) | 4.85(7.8-2.64) | -0.621(-0.738--0.504) |
| Nigeria | Female | 1766.95(1042-2874.53) | 3896.37(2180.88-6570.18) | 120.51 | 8.42(13.63-5.06) | 8.61(14.07-4.85) | 0.067(-0.127-0.262) |
| Niue | Female | 0.02(0.01-0.04) | 0.02(0.01-0.03) | 0.00 | 1.99(3.2-1.19) | 1.59(2.6-0.91) | -0.767(-1.043--0.49) |
| North Macedonia | Female | 21.32(15.83-29.59) | 34.33(24.16-46.62) | 61.02 | 2.19(3.01-1.62) | 2.09(2.82-1.48) | -0.037(-0.403-0.33) |
| Northern Mariana Islands | Female | 0.27(0.17-0.39) | 0.52(0.32-0.77) | 92.59 | 3.43(5.02-2.17) | 2.05(2.97-1.32) | -1.749(-1.857--1.641) |
| Norway | Female | 59.06(47.13-72.85) | 67.62(49.56-91.8) | 14.49 | 1.76(2.14-1.42) | 1.35(1.78-1.02) | -0.981(-1.344--0.617) |
| Oman | Female | 3.03(1.68-5.11) | 4.92(2.77-7.92) | 62.38 | 1.05(1.76-0.57) | 0.72(1.15-0.42) | -1.246(-1.487--1.004) |
| Pakistan | Female | 1445.33(774.19-2575.57) | 2534.83(1563.07-3954.55) | 75.38 | 5.78(10.63-3.02) | 4.89(7.8-2.98) | -0.584(-0.734--0.434) |
| Palau | Female | 0.07(0.03-0.13) | 0.13(0.06-0.24) | 85.71 | 1.43(2.48-0.69) | 1.23(2.15-0.6) | -0.511(-0.608--0.413) |
| Palestine | Female | 6.99(3.2-15.02) | 21.47(9.7-43.67) | 207.15 | 1.47(3.14-0.66) | 1.86(3.77-0.84) | 0.842(0.663-1.021) |
| Panama | Female | 23.31(17.69-29.83) | 65.32(43.7-94.42) | 180.22 | 3.06(3.92-2.3) | 3.05(4.42-2.02) | 0.001(-0.731-0.738) |
| Papua New Guinea | Female | 4.39(2.46-7.08) | 11.23(6.24-18.42) | 155.81 | 0.41(0.68-0.23) | 0.4(0.68-0.22) | -0.032(-0.099-0.035) |
| Paraguay | Female | 37.49(25.72-52.67) | 57.05(34.29-93.37) | 52.17 | 3.03(4.29-2.06) | 1.92(3.14-1.15) | -1.581(-1.881--1.28) |
| Peru | Female | 436.09(322-566.9) | 839.98(523.13-1249.61) | 92.62 | 6.75(8.83-4.95) | 5.04(7.53-3.12) | -1.053(-1.626--0.477) |
| Philippines | Female | 699.98(443.28-1106.39) | 1375.89(883.35-2113.99) | 96.56 | 4.17(6.83-2.64) | 3.21(4.91-2.05) | -0.892(-1.129--0.655) |
| Poland | Female | 948.64(848.37-1062.97) | 1175.21(901.15-1503.82) | 23.88 | 3.77(4.22-3.38) | 3.49(4.53-2.63) | -0.439(-0.696--0.181) |
| Portugal | Female | 709.41(521.8-898.28) | 329.75(227.72-458.28) | -53.52 | 9.94(12.54-7.41) | 2.8(3.82-1.99) | -4.292(-4.523--4.06) |
| Puerto Rico | Female | 100.35(68.64-146.27) | 110.75(70.83-164.78) | 10.36 | 5.21(7.64-3.58) | 2.94(4.36-1.91) | -2.046(-2.672--1.417) |
| Qatar | Female | 0.44(0.27-0.69) | 2.09(1.11-3.61) | 375.00 | 1.39(2.2-0.81) | 1.66(2.78-0.89) | 0.614(-0.117-1.351) |
| Republic of Korea | Female | 2281.28(1649.22-2886.62) | 1782.26(1191.21-2427.77) | -21.87 | 13.23(17.2-9.37) | 3.72(5.03-2.53) | -4.285(-4.531--4.038) |
| Republic of Moldova | Female | 1158.66(857.57-1459.4) | 716.98(512.6-960.25) | -38.12 | 43.49(54.73-32.49) | 22.47(30.13-16.07) | -2.317(-3.614--1.002) |
| Romania | Female | 1580.58(1178.83-2064.8) | 1985.29(1413.94-2707.88) | 25.61 | 10.29(13.49-7.7) | 10.38(14.15-7.39) | 0.093(-0.487-0.676) |
| Russian Federation | Female | 3281.38(2601.8-4125.04) | 10411.82(7476.84-14204.94) | 217.30 | 3.01(3.81-2.36) | 8.89(12.15-6.42) | 4.1(3.084-5.126) |
| Rwanda | Female | 481.97(265.42-727.57) | 444.62(243.04-690.85) | -7.75 | 28.64(43.97-15.96) | 12.51(19.46-6.7) | -2.822(-3.108--2.536) |
| Saint Kitts and Nevis | Female | 1.21(0.85-1.62) | 0.88(0.54-1.44) | -27.27 | 6.1(8.22-4.32) | 2.53(4.12-1.57) | -2.97(-3.26--2.679) |
| Saint Lucia | Female | 4.87(3.41-6.51) | 4.95(3.34-7.02) | 1.64 | 10.04(13.48-7.04) | 4.38(6.17-2.97) | -2.797(-3.077--2.516) |
| Saint Vincent and the Grenadines | Female | 1.16(0.84-1.6) | 1.64(1.1-2.4) | 41.38 | 2.96(4.07-2.1) | 2.51(3.66-1.69) | -0.575(-0.8--0.35) |
| Samoa | Female | 0.91(0.56-1.41) | 1.29(0.72-2.09) | 41.76 | 2.02(3.1-1.25) | 1.73(2.78-0.96) | -0.535(-0.604--0.467) |
| San Marino | Female | 1.17(0.61-1.73) | 2.14(0.95-3.69) | 82.91 | 6.61(9.81-3.49) | 6.07(10.46-2.79) | -0.294(-0.4--0.188) |
| Sao Tome and Principe | Female | 4.33(2.38-7.04) | 6.41(3.12-11.29) | 48.04 | 12.69(20.62-7.01) | 11.33(20.45-5.3) | -0.457(-0.954-0.043) |
| Saudi Arabia | Female | 50.49(23.5-97.73) | 58.93(35.47-91.85) | 16.72 | 2.22(4.29-1.04) | 1.03(1.64-0.61) | -2.622(-2.741--2.503) |
| Senegal | Female | 80.76(50.8-126.76) | 143.88(77.86-236.38) | 78.16 | 4.77(7.42-2.97) | 3.65(6.09-1.94) | -0.768(-1.09--0.445) |
| Serbia | Female | 171(120.8-244.79) | 164.86(117.23-232.95) | -3.59 | 2.82(3.96-2.05) | 2.05(2.91-1.46) | -1.052(-1.634--0.466) |
| Seychelles | Female | 0.76(0.52-1.06) | 1.96(1.18-3.22) | 157.89 | 2.43(3.35-1.64) | 3.45(5.67-2.08) | 1.225(1.011-1.44) |
| Sierra Leone | Female | 134.43(78.57-218.24) | 166.51(77.48-308.66) | 23.86 | 13.94(22.7-8.17) | 8.45(15.79-3.97) | -1.685(-1.849--1.521) |
| Singapore | Female | 9.5(6.45-13.62) | 16.59(10.44-24.66) | 74.63 | 0.76(1.1-0.51) | 0.41(0.61-0.26) | -2.091(-2.294--1.887) |
| Slovakia | Female | 214.26(166.46-281.92) | 235.19(164.85-324.77) | 9.77 | 6.8(8.87-5.24) | 5.12(7.18-3.58) | -1.015(-1.375--0.653) |
| Slovenia | Female | 160.96(96.84-241.79) | 66.33(45.43-98.34) | -58.79 | 11.7(17.62-7.07) | 3.22(4.7-2.22) | -4.345(-4.93--3.757) |
| Solomon Islands | Female | 2.41(1.4-3.94) | 5.02(3.22-7.5) | 108.30 | 3.21(5.21-1.85) | 2.71(3.97-1.73) | -0.604(-0.954--0.252) |
| Somalia | Female | 87.93(43.11-150.54) | 215.28(113.1-380.05) | 144.83 | 6.58(11.31-3.3) | 5.83(10.11-3.08) | -0.403(-0.519--0.287) |
| South Africa | Female | 809.39(537.93-1250.02) | 907.83(602.73-1311.81) | 12.16 | 6.05(9.57-4) | 3.48(5.06-2.29) | -1.906(-2.656--1.149) |
| South Sudan | Female | 52.45(31.81-81.51) | 80.37(46.35-133.6) | 53.23 | 5.08(7.94-3.04) | 4.63(7.66-2.69) | -0.301(-0.372--0.23) |
| Spain | Female | 2413.87(1725.74-3140.38) | 1492.37(948.84-2185.04) | -38.18 | 8.15(10.52-5.88) | 2.69(3.74-1.85) | -3.768(-4.128--3.406) |
| Sri Lanka | Female | 99.27(67-141.12) | 233.61(132.05-402.61) | 135.33 | 1.88(2.65-1.27) | 1.72(2.91-0.98) | -0.179(-0.737-0.382) |
| Sudan | Female | 66.24(28.82-138.17) | 70.66(36.96-122.53) | 6.67 | 1.44(3.04-0.63) | 0.88(1.58-0.44) | -1.636(-2.037--1.233) |
| Suriname | Female | 9.88(7.13-13.57) | 15.6(10.47-22.81) | 57.89 | 6.97(9.59-5.06) | 4.82(7.07-3.25) | -1.22(-1.708--0.729) |
| Sweden | Female | 211.53(164.56-258.18) | 275.7(206.71-350.41) | 30.34 | 2.79(3.37-2.2) | 2.6(3.23-2) | -0.161(-0.42-0.099) |
| Switzerland | Female | 228.6(181.86-271.04) | 296.9(223.36-391.84) | 29.88 | 4.27(5.01-3.42) | 3.22(4.03-2.51) | -0.967(-1.346--0.587) |
| Syrian Arab Republic | Female | 33.37(17.97-60.36) | 46.27(26.74-76.19) | 38.66 | 1.31(2.32-0.7) | 0.92(1.46-0.55) | -1.232(-1.561--0.902) |
| Taiwan (Province of China) | Female | 435.07(252.25-695.77) | 406.29(238.51-663.11) | -6.62 | 6.24(10-3.58) | 1.9(3.1-1.13) | -4.003(-4.285--3.719) |
| Tajikistan | Female | 123.49(93.96-159.1) | 292.63(210.07-389.81) | 136.97 | 7.52(9.69-5.75) | 10.82(14.35-7.78) | 1.301(1.008-1.595) |
| Thailand | Female | 1027.26(682.31-1506.31) | 1809.54(1072.95-2866.91) | 76.15 | 4.94(7.17-3.25) | 3.28(5.19-1.96) | -1.396(-1.59--1.202) |
| Timor-Leste | Female | 4.74(2.78-7.43) | 11.82(6.66-19.21) | 149.37 | 3.12(4.86-1.83) | 2.9(4.59-1.68) | -0.227(-0.438--0.016) |
| Togo | Female | 81.69(46.75-128.87) | 133.11(63.03-230.1) | 62.95 | 11.12(17.8-6.38) | 5.88(10.02-2.87) | -2.188(-2.379--1.996) |
| Tokelau | Female | 0.02(0.01-0.03) | 0.01(0.01-0.02) | -50.00 | 2.58(4.31-1.36) | 1.78(2.95-0.99) | -1.257(-1.322--1.192) |
| Tonga | Female | 0.73(0.47-1.08) | 0.86(0.53-1.31) | 17.81 | 2.52(3.7-1.64) | 2.01(3.08-1.23) | -0.773(-0.924--0.622) |
| Trinidad and Tobago | Female | 16.35(11.83-22.22) | 23.69(14.43-36.49) | 44.89 | 3.59(4.87-2.6) | 2.53(3.92-1.54) | -1.102(-2.077--0.117) |
| Tunisia | Female | 19.88(12.28-31.15) | 50.3(27.08-92.61) | 153.02 | 0.79(1.24-0.48) | 0.79(1.42-0.43) | 0.007(-0.121-0.136) |
| Turkey | Female | 262.48(140.9-461.19) | 392.1(214.95-642.14) | 49.38 | 1.33(2.31-0.73) | 0.85(1.38-0.46) | -1.578(-1.744--1.412) |
| Turkmenistan | Female | 110.14(86.2-135.64) | 287.8(206.93-382.47) | 161.30 | 9.7(11.85-7.59) | 12.36(16.44-9) | 0.843(0.294-1.396) |
| Tuvalu | Female | 0.11(0.05-0.18) | 0.1(0.05-0.18) | -9.09 | 2.7(4.53-1.44) | 1.89(3.25-0.99) | -1.211(-1.329--1.094) |
| Uganda | Female | 422.96(227.3-658.5) | 1017.47(527.03-1546.18) | 140.56 | 12.48(19.69-6.55) | 12.59(19.23-6.24) | 0.051(-0.121-0.223) |
| Ukraine | Female | 1440.45(1123.88-1834.56) | 3760.6(2542.91-5164.76) | 161.07 | 3.62(4.58-2.83) | 11.25(15.49-7.6) | 4.208(2.033-6.43) |
| United Arab Emirates | Female | 3.32(1.69-5.88) | 6.3(2.87-12.02) | 89.76 | 2.03(3.66-1.02) | 0.61(1.13-0.29) | -4.069(-4.749--3.384) |
| United Kingdom | Female | 1138.63(931.07-1346.14) | 2192.87(1739.91-2678) | 92.59 | 2.68(3.15-2.22) | 4.05(4.92-3.27) | 1.507(1.21-1.806) |
| United Republic of Tanzania | Female | 875.18(497.15-1350.38) | 1445.67(678.65-2516.9) | 65.19 | 15.44(24.04-8.64) | 11.16(19.73-5.09) | -1.085(-1.249--0.922) |
| United States of America | Female | 7440.36(5559.09-9552.25) | 15064.84(11083.21-19471.5) | 102.47 | 4.49(5.75-3.39) | 5.61(7.14-4.24) | 0.762(0.551-0.974) |
| United States Virgin Islands | Female | 2.79(1.52-4.76) | 3.96(2.14-6.97) | 41.94 | 6.06(10.47-3.29) | 3.96(6.95-2.16) | -1.45(-1.561--1.338) |
| Uruguay | Female | 89.75(63.67-120.07) | 79.25(50.45-111.72) | -11.70 | 4.21(5.57-3) | 2.46(3.45-1.66) | -1.967(-2.143--1.79) |
| Uzbekistan | Female | 548.39(425.4-681.11) | 1926.91(1456.61-2462.37) | 251.38 | 8.25(10.21-6.4) | 15.33(19.41-11.57) | 2.237(1.91-2.564) |
| Vanuatu | Female | 0.77(0.42-1.26) | 1.76(0.95-2.9) | 128.57 | 2.56(4.23-1.37) | 2.08(3.43-1.15) | -0.779(-1.133--0.423) |
| Venezuela (Bolivarian Republic of) | Female | 232.5(168.59-321.22) | 366.2(232.56-562.43) | 57.51 | 4.28(5.92-3.06) | 2.35(3.58-1.5) | -2.216(-2.497--1.934) |
| Viet Nam | Female | 708.93(414.26-1099.26) | 1069.35(671.85-1673.18) | 50.84 | 3.12(4.85-1.82) | 2.11(3.27-1.32) | -1.373(-1.513--1.233) |
| Yemen | Female | 71.79(25.85-155.4) | 64.06(34.91-108.49) | -10.77 | 2.79(6.06-1) | 0.96(1.64-0.51) | -3.608(-3.685--3.53) |
| Zambia | Female | 263.38(147.12-437.58) | 532.17(267.24-882.38) | 102.05 | 18.03(30.24-10.1) | 14.58(24.8-7.21) | -0.715(-0.943--0.486) |
| Zimbabwe | Female | 97.75(53.97-163.55) | 153.71(53.27-296.28) | 57.25 | 4.47(7.29-2.52) | 3.69(6.95-1.31) | -0.683(-1.144--0.22) |
| Afghanistan | Male | 107.65(53.69-179.07) | 145.71(77.19-241.32) | 35.36 | 2.94(4.8-1.5) | 2.41(3.9-1.3) | -0.622(-0.756--0.489) |
| Albania | Male | 83.19(63.73-105.49) | 144.27(93.23-209.89) | 73.42 | 8.4(10.54-6.44) | 7.47(10.84-4.83) | -0.423(-0.819--0.025) |
| Algeria | Male | 170.74(92.98-286.64) | 536.64(284.99-947.05) | 214.30 | 2.75(4.45-1.55) | 2.97(5.22-1.59) | 0.278(0.178-0.378) |
| American Samoa | Male | 0.97(0.54-1.73) | 1.02(0.61-1.73) | 5.15 | 6.51(11.21-3.81) | 4.09(6.74-2.52) | -1.616(-1.852--1.379) |
| Andorra | Male | 3.67(2.24-5.6) | 6.5(4.57-8.72) | 77.11 | 12.76(19.26-7.77) | 9.29(12.46-6.54) | -1.083(-1.137--1.029) |
| Angola | Male | 750.9(446.43-1156.43) | 2808.71(1944.82-3843.52) | 274.05 | 33.06(50.03-19.93) | 49.4(66.81-35.24) | 1.406(1.228-1.584) |
| Antigua and Barbuda | Male | 2.85(2.15-3.65) | 5.44(3.91-7.06) | 90.88 | 12.75(16.28-9.55) | 11.11(14.61-7.97) | -0.396(-1.087-0.3) |
| Argentina | Male | 3529.98(3017.61-3995.47) | 4802.87(3884.01-5694.09) | 36.06 | 24.21(27.38-20.65) | 20.43(24.24-16.48) | -0.496(-0.631--0.361) |
| Armenia | Male | 120.63(94.39-154.2) | 353.48(265.94-458.13) | 193.03 | 9.72(12.14-7.57) | 20.76(26.79-15.81) | 2.704(2.14-3.272) |
| Australia | Male | 788.85(678.26-875.92) | 1160.32(981.79-1338.71) | 47.09 | 8.93(9.91-7.67) | 6.5(7.47-5.5) | -1.088(-1.416--0.758) |
| Austria | Male | 1294.95(1096.89-1434.61) | 996.39(803.08-1133.05) | -23.06 | 28.67(31.85-24.28) | 13.63(15.48-11.07) | -2.646(-3.018--2.273) |
| Azerbaijan | Male | 611.5(463.3-746.42) | 1292.34(865.2-1804.47) | 111.34 | 28.46(34.99-21.47) | 31.47(44.39-20.06) | 0.33(0.216-0.445) |
| Bahamas | Male | 19.92(15.79-23.83) | 26.99(17.8-38.24) | 35.49 | 25.63(30.91-20.22) | 13.71(19.19-9.14) | -2.15(-2.507--1.792) |
| Bahrain | Male | 18.29(11.95-25.33) | 34.75(18.83-57.74) | 89.99 | 17.85(26.22-10.95) | 4.49(7.89-2.41) | -4.737(-5.355--4.116) |
| Bangladesh | Male | 3786.5(2517.76-5298.59) | 4886.4(3262.9-7049.13) | 29.05 | 13.78(19.24-9.07) | 7.43(10.63-4.92) | -1.982(-2.202--1.762) |
| Barbados | Male | 17.23(13.38-21.14) | 24.94(17.81-32.73) | 44.75 | 15.21(18.63-11.7) | 11.63(15.24-8.39) | -0.957(-1.408--0.505) |
| Belarus | Male | 391.42(308.64-466.19) | 1085.75(764.97-1493.75) | 177.39 | 7.79(9.24-6.17) | 17.63(24-12.55) | 2.853(1.627-4.094) |
| Belgium | Male | 829.89(697.29-931.62) | 1021.32(819.23-1208.43) | 23.07 | 13.51(15.18-11.38) | 10.89(12.87-8.74) | -0.786(-0.932--0.641) |
| Belize | Male | 7.37(5.44-9.25) | 32.38(24.09-41.37) | 339.35 | 15.05(19-11.03) | 20.64(26.65-15.36) | 1.16(0.798-1.523) |
| Benin | Male | 307.14(186.36-454.77) | 639.2(391.31-955.96) | 108.11 | 30.16(44.68-18.26) | 23.92(35.66-14.9) | -0.799(-0.972--0.624) |
| Bermuda | Male | 5.1(4.1-6.06) | 4.33(3.07-5.6) | -15.10 | 18.11(21.69-14.49) | 7.89(10.17-5.57) | -2.86(-3.025--2.694) |
| Bhutan | Male | 42.24(23.33-77.47) | 41.34(22.01-91.54) | -2.13 | 25.64(46.05-14.07) | 13.21(29.83-7.34) | -2.282(-2.46--2.104) |
| Bolivia (Plurinational State of) | Male | 492.67(305.03-736.3) | 1187.08(761.19-1726.96) | 140.95 | 29.67(44.62-18.27) | 27.06(39.45-17.45) | -0.296(-0.44--0.152) |
| Bosnia and Herzegovina | Male | 377.55(294.31-457.8) | 315.07(218.92-424.06) | -16.55 | 18.31(22.22-14.23) | 11.95(16.04-8.37) | -1.515(-1.904--1.125) |
| Botswana | Male | 111.93(64.93-185.85) | 207.46(129.95-295.29) | 85.35 | 38.76(62.66-23.1) | 28.6(39.66-18.31) | -1.031(-1.217--0.846) |
| Brazil | Male | 11834.94(9627.99-14247.75) | 19127.8(15812.15-23398.01) | 61.62 | 23.54(28.42-19.23) | 16.83(20.68-13.9) | -1.165(-1.38--0.95) |
| Brunei Darussalam | Male | 3.55(2.21-5.32) | 5.86(3.82-8.33) | 65.07 | 5.11(7.69-3.25) | 3.17(4.41-2.19) | -1.631(-2.041--1.221) |
| Bulgaria | Male | 1387.6(1171.65-1578.64) | 1739.61(1273.56-2266.11) | 25.37 | 24.12(27.5-20.45) | 31.23(40.66-22.98) | 1.053(0.689-1.419) |
| Burkina Faso | Male | 829.74(583.78-1111.11) | 1149.5(507.95-2108.58) | 38.54 | 39.84(53.34-28.1) | 26.24(47.77-11.71) | -1.385(-1.638--1.132) |
| Burundi | Male | 732.7(422.47-1164.53) | 952.64(493.02-1902.85) | 30.02 | 64.22(99.57-37.84) | 35.18(68.43-17.93) | -2.066(-2.246--1.885) |
| Cabo Verde | Male | 21.31(13.8-33.3) | 55.06(38.35-73.53) | 158.38 | 23.61(36.76-15.27) | 26.6(35.11-18.68) | 0.586(-0.01-1.186) |
| Cambodia | Male | 505.59(300.82-840.18) | 3559.02(2465.81-4710.63) | 603.93 | 21.4(34.13-12.89) | 61.38(80.31-42.2) | 3.681(3.497-3.864) |
| Cameroon | Male | 979.08(665.54-1369.78) | 1934.44(1134.93-2974.98) | 97.58 | 41.48(58.57-28.22) | 28.46(43.65-17.12) | -1.292(-1.386--1.199) |
| Canada | Male | 1500.12(1183.82-1786.4) | 2495.76(1945.19-3049.2) | 66.37 | 10.45(12.47-8.22) | 8.32(10.1-6.55) | -0.757(-0.849--0.665) |
| Central African Republic | Male | 294.67(155.65-503.46) | 323.45(134.29-613.9) | 9.77 | 47.32(79.44-25.45) | 26.95(49.24-12.06) | -1.943(-2.12--1.765) |
| Chad | Male | 276.67(135.54-496.42) | 972.06(471.64-1533.61) | 251.34 | 19.55(34.68-9.83) | 28.13(44.87-13.66) | 1.301(1.001-1.601) |
| Chile | Male | 2223.03(1848.39-2552.44) | 2809.06(2285.98-3298.66) | 26.36 | 45.23(52.13-37.47) | 25.59(30.07-20.86) | -1.987(-2.548--1.423) |
| China | Male | 76833.93(54994.44-99708.33) | 81310.65(57396.53-106467.54) | 5.83 | 16.69(21.61-11.97) | 8.58(11.16-6.16) | -2.299(-2.457--2.142) |
| Colombia | Male | 878.22(702.15-1050.67) | 1386.05(918.49-1982.88) | 57.82 | 9.55(11.55-7.53) | 5.73(8.19-3.81) | -2.357(-2.577--2.137) |
| Comoros | Male | 12.22(4.89-27.24) | 23.64(12.39-42.74) | 93.45 | 11.2(25.08-4.74) | 10.36(19.22-5.41) | -0.233(-0.697-0.234) |
| Congo | Male | 253.59(151.57-370.36) | 424.3(234.79-631.92) | 67.32 | 48(68.28-29.47) | 29.82(43.7-16.33) | -1.613(-1.861--1.364) |
| Cook Islands | Male | 0.5(0.25-0.93) | 1.24(0.8-1.67) | 148.00 | 6.84(12.54-3.48) | 10.73(14.44-7) | 1.598(1.379-1.818) |
| Costa Rica | Male | 147.7(119.05-174.68) | 386.74(260.29-548.11) | 161.84 | 15.95(19.03-12.77) | 15.86(22.45-10.67) | -0.015(-0.528-0.501) |
| Croatia | Male | 1074.12(900.72-1238.29) | 688.97(492.4-915.79) | -35.86 | 38.03(43.79-31.93) | 19.55(25.85-14.12) | -2.338(-2.834--1.84) |
| Cuba | Male | 394.9(304.13-497.83) | 981.19(681.73-1326.44) | 148.47 | 7.75(9.78-5.97) | 11.26(15.16-7.89) | 1.372(0.612-2.138) |
| Cyprus | Male | 48.17(32.86-64.9) | 72.61(54.56-89.74) | 50.74 | 13.41(18.68-9.13) | 8.48(10.57-6.33) | -1.606(-1.857--1.355) |
| Czechia | Male | 1378.69(1177.79-1543.95) | 1321.04(987.87-1717.7) | -4.18 | 23.63(26.57-20.18) | 15.56(20.21-11.71) | -1.416(-1.836--0.993) |
| C么te d'Ivoire | Male | 959.25(588.17-1415.97) | 1904.75(1195.17-2790.78) | 98.57 | 35.96(53.81-21.99) | 27.77(40.44-17.55) | -0.877(-1.059--0.695) |
| Democratic People's Republic of Korea | Male | 1606.96(960.86-2360.04) | 2269.36(1133.17-3361.27) | 41.22 | 21.59(31.24-13.74) | 15.69(22.5-7.92) | -1.095(-1.168--1.022) |
| Democratic Republic of the Congo | Male | 2728.25(1566.55-4094.78) | 3993.45(2013.52-7012.4) | 46.37 | 33.06(49.83-18.94) | 20.39(36.27-10.24) | -1.657(-1.947--1.367) |
| Denmark | Male | 429.9(357.72-485.62) | 485.21(381.92-577.86) | 12.87 | 13.43(15.16-11.21) | 10.26(12.23-8.04) | -0.891(-1.149--0.632) |
| Djibouti | Male | 12.92(7.02-26.26) | 40.1(22.56-74.22) | 210.37 | 14.88(29.27-8.56) | 11.61(22.33-6.39) | -0.81(-1.009--0.61) |
| Dominica | Male | 4.34(3.16-5.59) | 4.87(3.43-6.65) | 12.21 | 15.62(20.01-11.44) | 11.12(15.1-7.79) | -1.185(-1.293--1.077) |
| Dominican Republic | Male | 545.94(410.34-691.65) | 1181.36(707.99-1786.25) | 116.39 | 27.46(35.12-20.6) | 25.62(38.29-15.76) | -0.241(-0.574-0.092) |
| Ecuador | Male | 507.98(394.07-650.72) | 1354.49(940.17-1900.65) | 166.64 | 17.31(22.06-13.46) | 18.37(25.69-12.7) | 0.138(-0.175-0.451) |
| Egypt | Male | 1870.74(980.29-3283.45) | 3800.57(1730.17-7255.52) | 103.16 | 13.17(22.13-7.24) | 11.21(20.37-5.42) | -0.61(-1.075--0.143) |
| El Salvador | Male | 373.27(287.35-465) | 561.25(370.23-801.67) | 50.36 | 23.93(29.87-18.47) | 22.26(31.93-14.57) | -0.277(-1.541-1.002) |
| Equatorial Guinea | Male | 27.49(12.52-51.8) | 56.32(30.96-95.96) | 104.87 | 28.22(52.85-13.23) | 23.57(39.45-13.47) | -0.635(-0.95--0.318) |
| Eritrea | Male | 110.87(53.71-202.7) | 309.06(170.42-556.83) | 178.76 | 21.72(38.6-11.56) | 21.82(37.91-12.82) | 0.005(-0.15-0.16) |
| Estonia | Male | 66.21(52.38-79.5) | 154.07(106.99-207.75) | 132.70 | 8.18(9.85-6.49) | 17.27(23.2-12) | 2.747(1.215-4.302) |
| Eswatini | Male | 56.4(37.94-80.6) | 96.98(61.09-146.9) | 71.95 | 38.37(54.55-25.78) | 34.62(50.66-22.45) | -0.384(-0.561--0.206) |
| Ethiopia | Male | 3823.21(1659.79-7204.41) | 7259.43(4416.33-10689.81) | 89.88 | 32.7(61.37-14.86) | 31.29(46.32-18.96) | -0.127(-0.269-0.016) |
| Fiji | Male | 21.37(13.85-30.08) | 32.05(19.13-48) | 49.98 | 8.92(12.65-5.77) | 7.97(11.89-4.78) | -0.364(-0.768-0.041) |
| Finland | Male | 332(276.64-383.88) | 640.97(513.2-755.16) | 93.06 | 11.19(12.94-9.33) | 14.58(17.08-11.83) | 0.855(0.546-1.166) |
| France | Male | 7708.27(6588.5-8470.46) | 5838.3(4772.3-6746.94) | -24.26 | 23.1(25.5-19.75) | 10.98(12.65-9.04) | -2.523(-2.773--2.272) |
| Gabon | Male | 174.96(110.89-249.88) | 216.12(136.73-305.48) | 23.53 | 66.68(95.61-42.05) | 41.59(58.83-26.77) | -1.584(-1.777--1.391) |
| Gambia | Male | 45.27(24.77-75.77) | 130.66(78.76-197.62) | 188.62 | 21.16(34.81-11.66) | 24.84(37.8-14.94) | 0.681(0.268-1.096) |
| Georgia | Male | 737.21(539.13-950.4) | 878.3(636.78-1123.99) | 19.14 | 28(36.34-20.53) | 38.49(49.08-28.04) | 1.03(0.132-1.935) |
| Germany | Male | 11932.21(10227.92-13118.41) | 10919.84(8994.78-12575.08) | -8.48 | 24.03(26.45-20.67) | 14.39(16.57-11.92) | -1.768(-2.049--1.487) |
| Ghana | Male | 1541.8(967.24-2435.43) | 2741.55(1787.03-3883.83) | 77.81 | 47.14(75.03-28.99) | 34.42(48.64-22.38) | -1.111(-1.32--0.901) |
| Greece | Male | 922.07(770.83-1030.72) | 702.39(574.67-817.61) | -23.82 | 13.75(15.43-11.52) | 7.34(8.48-6) | -2.143(-2.362--1.923) |
| Greenland | Male | 1.97(1.16-2.9) | 3.35(2.08-4.73) | 70.05 | 8.01(11.82-4.93) | 7.89(11.14-4.93) | -0.038(-0.219-0.143) |
| Grenada | Male | 7.11(5.46-8.73) | 10.4(7.91-12.92) | 46.27 | 24.73(30.15-18.95) | 18.33(22.78-13.96) | -1.037(-1.547--0.525) |
| Guam | Male | 4.53(2.2-9.09) | 9.53(3.72-17.94) | 110.38 | 9.59(18.69-5.34) | 10.09(19-3.97) | 0.155(-0.278-0.589) |
| Guatemala | Male | 885.06(645.64-1172.73) | 2034.25(1392.4-2784.26) | 129.84 | 38.81(51.07-28.41) | 34.83(47.74-24.03) | -0.308(-0.965-0.354) |
| Guinea | Male | 284.29(169.66-461.62) | 579.05(337.3-922.22) | 103.68 | 16.53(26.64-9.75) | 18.98(30.37-11.16) | 0.476(0.338-0.615) |
| Guinea-Bissau | Male | 114.59(62.03-176.4) | 172.9(99.12-259.08) | 50.89 | 52.44(79.85-29.41) | 42.37(63.01-24.13) | -0.737(-0.802--0.673) |
| Guyana | Male | 129.92(100.68-157.96) | 146.04(103.49-192.19) | 12.41 | 60.8(74.1-46.95) | 43.87(57.85-30.93) | -1.051(-1.747--0.35) |
| Haiti | Male | 624.49(327.97-909.65) | 967.48(519.29-1466.36) | 54.92 | 35.44(51.06-18.83) | 26.33(40.12-14.15) | -0.973(-1.09--0.857) |
| Honduras | Male | 336.54(220.06-473.23) | 894.17(495.52-1382.44) | 165.70 | 29.62(41.56-19.57) | 30.33(47.04-16.72) | 0.091(-0.037-0.22) |
| Hungary | Male | 3532.49(3034.08-3925.7) | 2072(1582.59-2612.7) | -41.34 | 57.43(63.86-49.48) | 27.78(35.03-21.17) | -2.569(-3.219--1.914) |
| Iceland | Male | 5.1(3.91-6.4) | 9.16(7.07-11.1) | 79.61 | 4(5.01-3.06) | 3.68(4.47-2.85) | -0.205(-0.399--0.01) |
| India | Male | 42607.03(29147.14-62662.05) | 102756.41(64738.46-149198.24) | 141.17 | 14.31(20.85-9.91) | 16.34(23.99-10.3) | 0.435(-0.082-0.954) |
| Indonesia | Male | 5256.56(4007.86-6817.41) | 9158.76(6727.67-12106.4) | 74.23 | 10.09(12.87-7.84) | 8.69(11.24-6.54) | -0.523(-0.605--0.441) |
| Iran (Islamic Republic of) | Male | 210.53(143.95-332.48) | 982.99(524.94-1719.01) | 366.91 | 1.5(2.24-1.09) | 2.51(4.44-1.35) | 1.734(1.36-2.108) |
| Iraq | Male | 290.32(157.43-470.33) | 294.23(139.1-546.53) | 1.35 | 6.76(11.27-3.65) | 2.27(4.11-1.14) | -3.728(-3.847--3.608) |
| Ireland | Male | 88.22(70.95-103.5) | 189.98(147.33-227.79) | 115.35 | 4.95(5.8-3.98) | 5.7(6.81-4.44) | 0.42(0.105-0.735) |
| Israel | Male | 100.91(78.51-123.96) | 202.58(155.61-264.86) | 100.75 | 4.66(5.69-3.64) | 3.94(5.16-3.04) | -0.701(-0.904--0.498) |
| Italy | Male | 9243.99(7959.88-10072.22) | 5306.65(4330.64-6079.68) | -42.59 | 25(27.25-21.59) | 9.05(10.29-7.41) | -3.468(-3.615--3.321) |
| Jamaica | Male | 53.09(39.81-67.39) | 70.08(47.11-98.28) | 32.00 | 6.59(8.35-4.95) | 4.84(6.79-3.26) | -0.724(-2.237-0.813) |
| Japan | Male | 11546.16(9481.54-13567.26) | 9499.26(7410.96-11718.14) | -17.73 | 15.36(18.13-12.56) | 7.14(8.67-5.67) | -2.608(-2.897--2.318) |
| Jordan | Male | 19.99(9.77-37.97) | 74.88(35.97-133.05) | 274.59 | 2.55(4.74-1.31) | 1.91(3.33-0.99) | -0.977(-1.434--0.517) |
| Kazakhstan | Male | 808.76(609.69-1004.71) | 2728.79(1996-3658.27) | 237.40 | 15.32(19.47-11.35) | 33.94(45.3-25.13) | 2.853(2.105-3.607) |
| Kenya | Male | 2244.62(1296.92-4156.06) | 4881.08(3003.84-7468.33) | 117.46 | 52.56(97.81-30.38) | 42.54(65.37-26.28) | -0.73(-0.817--0.642) |
| Kiribati | Male | 5.59(1.86-12.62) | 6.03(1.83-15.37) | 7.87 | 24.99(53.93-9.67) | 14.56(35.45-5.13) | -1.823(-1.958--1.688) |
| Kuwait | Male | 3.26(2.04-4.91) | 13.68(8.1-21.01) | 319.63 | 0.77(1.15-0.49) | 0.85(1.34-0.51) | 0.42(-0.924-1.782) |
| Kyrgyzstan | Male | 360.18(272.94-447.74) | 1017.04(768.39-1289.29) | 182.37 | 25.75(32.15-19.75) | 40.05(51.13-30.19) | 1.55(0.9-2.204) |
| Lao People's Democratic Republic | Male | 318.56(179.34-474.74) | 592.16(363.93-876.77) | 85.89 | 26.67(40.13-15.46) | 23.05(34.71-14.22) | -0.513(-0.648--0.379) |
| Latvia | Male | 100.32(80.48-120.8) | 191.29(131.84-257.76) | 90.68 | 7.32(8.83-5.88) | 14.87(20.01-10.2) | 2.629(1.223-4.055) |
| Lebanon | Male | 102.25(52.84-157.12) | 113.15(36.92-207.7) | 10.66 | 9.24(14.48-4.77) | 4.84(8.9-1.57) | -2.22(-2.337--2.102) |
| Lesotho | Male | 128.33(71.45-216) | 174.67(96.44-269.07) | 36.11 | 26.38(44.71-14.52) | 27.87(42.46-15.96) | 0.2(0.059-0.341) |
| Liberia | Male | 281.02(191-386.2) | 383.48(218.29-587.17) | 36.46 | 45.93(63.61-30.97) | 30.34(46-17.5) | -1.445(-1.652--1.237) |
| Libya | Male | 18.23(9.09-32.15) | 72.5(26.51-146.39) | 297.70 | 1.87(3.36-0.91) | 2.51(5.09-1.02) | 0.988(0.401-1.578) |
| Lithuania | Male | 160.64(126.47-189.39) | 430.03(313.26-561.71) | 167.70 | 8.77(10.34-6.94) | 23.25(30.17-17.04) | 3.45(2.56-4.347) |
| Luxembourg | Male | 55.13(46.76-61.41) | 48.57(38.12-59.33) | -11.90 | 24.35(27.13-20.62) | 10.71(13.04-8.37) | -2.786(-3.062--2.508) |
| Madagascar | Male | 736(443.11-1142.96) | 1011.53(546.79-1703.87) | 37.44 | 25.45(39.68-15.42) | 16.29(26.5-9.18) | -1.484(-1.716--1.252) |
| Malawi | Male | 739.95(430.08-1123.6) | 1315.55(777.49-2034.77) | 77.79 | 35.82(53.5-21.34) | 34.2(53.14-20) | -0.145(-0.29-0.001) |
| Malaysia | Male | 556.32(336.64-828.43) | 1211.62(617.32-2087.49) | 117.79 | 10.64(16.11-6.33) | 8.24(14.18-4.32) | -0.884(-1.065--0.702) |
| Maldives | Male | 2.23(1.22-3.44) | 6(3.63-10.42) | 169.06 | 3.89(5.88-2.13) | 2.84(4.58-1.79) | -1.124(-1.59--0.656) |
| Mali | Male | 436.65(266.41-698.12) | 634.46(344.45-1122.79) | 45.30 | 20.17(32.15-12.34) | 13.27(23.02-7.26) | -1.367(-1.542--1.192) |
| Malta | Male | 17.46(13.69-20.81) | 20.53(15.46-25.31) | 17.58 | 9.11(10.88-7.1) | 5.43(6.66-4.09) | -1.763(-2.227--1.297) |
| Marshall Islands | Male | 2.18(0.98-3.88) | 3.43(1.42-6.74) | 57.34 | 20.32(35.53-9.82) | 14.62(28.73-6.38) | -1.131(-1.19--1.071) |
| Mauritania | Male | 82.69(50.2-125.32) | 91.88(49.74-157.41) | 11.11 | 16.94(25.81-10.3) | 8.56(14.4-4.72) | -2.327(-2.584--2.07) |
| Mauritius | Male | 146.78(114.67-179.14) | 130.64(87.2-177.65) | -11.00 | 36.38(44.62-28.31) | 15.57(21.14-10.41) | -2.952(-3.383--2.518) |
| Mexico | Male | 12518.91(10158.1-14420) | 23256.65(16820.66-30225.29) | 85.77 | 52.38(60.72-42.27) | 40.32(52.47-28.87) | -0.935(-1.154--0.717) |
| Micronesia (Federated States of) | Male | 8.63(4.26-14.67) | 7.05(2.68-13.53) | -18.31 | 29.43(49.35-15.21) | 16.32(30.87-6.55) | -2.03(-2.102--1.957) |
| Monaco | Male | 2.52(1.47-3.75) | 2.9(1.76-4.24) | 15.08 | 9.84(14.72-5.76) | 8.01(11.75-4.9) | -0.694(-0.776--0.612) |
| Mongolia | Male | 228.39(160.2-308.26) | 674.8(470.08-926.38) | 195.46 | 44.39(58.89-31.59) | 57.31(77.37-39.56) | 0.877(0.729-1.026) |
| Montenegro | Male | 22.43(17.72-27.98) | 30.69(22.14-41.09) | 36.83 | 7.55(9.41-5.98) | 6.99(9.32-5.06) | -0.232(-0.405--0.059) |
| Morocco | Male | 394.18(222.56-608.68) | 435.62(244.02-719.74) | 10.51 | 5.27(8.18-2.99) | 2.68(4.32-1.56) | -2.337(-2.644--2.029) |
| Mozambique | Male | 214.48(132.62-317.31) | 484.64(217.33-1044.46) | 125.96 | 7.71(11.43-4.9) | 8.8(17.86-4.16) | 0.473(0.311-0.634) |
| Myanmar | Male | 2185.99(1333.96-3344.52) | 9931.24(6379.6-13962.15) | 354.31 | 16.37(24.93-10.02) | 39.61(55.42-25.38) | 3.137(2.897-3.378) |
| Namibia | Male | 91.08(49.38-145.81) | 170.35(107.99-240.98) | 87.03 | 25.61(40.97-14.03) | 25.35(35.78-16.15) | 0.057(-0.269-0.385) |
| Nauru | Male | 0.87(0.34-1.5) | 0.78(0.32-1.36) | -10.34 | 28.13(47.94-11.47) | 24.85(42.42-11.3) | -0.422(-0.503--0.34) |
| Nepal | Male | 1129.66(748.68-1750.03) | 2543.68(1646.2-4101.65) | 125.17 | 20.82(32.58-13.62) | 23.4(37.67-15.42) | 0.464(0.304-0.623) |
| Netherlands | Male | 631.11(528.2-712.39) | 785.34(620.1-944.45) | 24.44 | 7.85(8.87-6.55) | 5.37(6.42-4.25) | -1.294(-1.507--1.081) |
| New Zealand | Male | 92.81(79.32-105.67) | 141.15(114.56-167.47) | 52.08 | 5.41(6.17-4.61) | 4.11(4.84-3.33) | -0.993(-1.161--0.826) |
| Nicaragua | Male | 215.84(161.15-276.96) | 747.46(516.29-1027.43) | 246.30 | 26.73(34.6-19.73) | 34.89(47.91-24.03) | 0.884(-0.033-1.809) |
| Niger | Male | 239.62(141.05-393.23) | 497.17(262.04-850.73) | 107.48 | 15.17(24.3-8.86) | 12.05(20.73-6.38) | -0.791(-0.973--0.609) |
| Nigeria | Male | 10522.89(6004.7-16742.3) | 17043.22(10301.53-28005.93) | 61.96 | 42.32(66.61-24.55) | 37.71(60.28-23.14) | -0.424(-0.611--0.237) |
| Niue | Male | 0.21(0.1-0.33) | 0.15(0.07-0.22) | -28.57 | 22.24(34.3-10.77) | 14.71(22.48-6.62) | -1.429(-1.505--1.353) |
| North Macedonia | Male | 120.3(98.45-142.24) | 183.11(129.25-247.68) | 52.21 | 12.62(14.96-10.33) | 11.75(15.75-8.41) | -0.279(-0.438--0.12) |
| Northern Mariana Islands | Male | 2.71(1.05-5.7) | 3.36(1.36-6.78) | 23.99 | 15.68(31.38-7.44) | 11.25(22.63-4.78) | -1.121(-1.252--0.989) |
| Norway | Male | 147.65(120.9-176.8) | 154.19(121.14-188.4) | 4.43 | 5.55(6.63-4.53) | 3.65(4.44-2.88) | -1.495(-1.794--1.195) |
| Oman | Male | 9.25(4.8-15.97) | 23.18(11.11-43.52) | 150.59 | 2.43(4.07-1.29) | 2.14(3.41-1.22) | -0.42(-0.882-0.044) |
| Pakistan | Male | 2016.08(909.23-3901.03) | 4946.44(2379.12-9145.17) | 145.35 | 5.91(11.52-2.67) | 6.8(12.89-3.24) | 0.47(0.373-0.568) |
| Palau | Male | 0.75(0.28-1.63) | 1.37(0.55-2.73) | 82.67 | 13.02(27.32-5.24) | 10.82(21.21-4.55) | -0.641(-0.686--0.596) |
| Palestine | Male | 30.16(13.21-59.97) | 73.66(39.74-119.12) | 144.23 | 7.58(15.11-3.38) | 6.42(10.8-3.26) | -0.637(-0.869--0.404) |
| Panama | Male | 86.74(66.53-106.17) | 214.6(144.86-295.66) | 147.41 | 11.08(13.68-8.42) | 10.56(14.57-7.13) | -0.128(-0.593-0.339) |
| Papua New Guinea | Male | 96.77(57.3-145.74) | 178.02(92.19-303.79) | 83.96 | 7.19(11.05-4.26) | 5.06(8.57-2.79) | -1.218(-1.39--1.045) |
| Paraguay | Male | 162.28(126.93-198.9) | 454.48(295.34-641.71) | 180.06 | 14.39(17.66-11.07) | 16.11(22.72-10.47) | 0.484(0.039-0.931) |
| Peru | Male | 1555.5(1126.55-2042.45) | 2699.78(1766.96-4001.93) | 73.56 | 24.11(31.57-17.79) | 17.11(25.32-11.23) | -1.142(-1.816--0.464) |
| Philippines | Male | 3935.18(2936.49-5213.39) | 7509.11(5247.19-10102.89) | 90.82 | 21.82(29.63-15.71) | 17.59(23.29-12.21) | -0.742(-0.881--0.604) |
| Poland | Male | 3257.48(2744.29-3689.96) | 4984.47(3755.97-6543.6) | 53.02 | 17.53(19.91-14.7) | 17.96(23.58-13.51) | -0.138(-0.803-0.532) |
| Portugal | Male | 2179.78(1860.22-2402.35) | 1236.77(1014.31-1411.61) | -43.26 | 37.31(41.09-31.9) | 13.79(15.74-11.37) | -3.384(-3.701--3.065) |
| Puerto Rico | Male | 571.28(436.14-691.48) | 449.03(298.99-628.76) | -21.40 | 34.44(41.66-26.29) | 16.33(22.81-10.83) | -2.477(-2.905--2.047) |
| Qatar | Male | 7.36(4.08-11.91) | 28.35(14.86-47.76) | 285.19 | 7.1(11.76-3.98) | 3.42(5.56-1.9) | -2.585(-3.585--1.574) |
| Republic of Korea | Male | 10093.35(8092.2-11623.91) | 5957.91(4659.33-7280.32) | -40.97 | 64.27(74.48-51.76) | 14.52(17.75-11.44) | -5.029(-5.177--4.881) |
| Republic of Moldova | Male | 1138.67(944.93-1279.17) | 1145.7(892.27-1385.98) | 0.62 | 58.92(66.27-48.81) | 47.28(57.13-36.94) | -0.612(-1.962-0.757) |
| Romania | Male | 4416.13(3685.31-4978.66) | 5405.56(4097.45-6889.63) | 22.40 | 33.89(38.21-28.35) | 36.13(45.91-27.26) | 0.103(-0.294-0.502) |
| Russian Federation | Male | 7062.77(5703-8240.23) | 21235.95(15747.07-26955.12) | 200.67 | 10.14(11.88-8.17) | 23.43(29.57-17.44) | 3.43(2.323-4.549) |
| Rwanda | Male | 1118.95(772.89-1469.32) | 1222.43(834.78-1805.87) | 9.25 | 79.36(104.69-55.58) | 42.72(61.68-29.61) | -2.13(-2.478--1.781) |
| Saint Kitts and Nevis | Male | 4.54(3.01-5.91) | 5.61(3.61-8.84) | 23.57 | 30.49(39.73-20.33) | 15.86(24.81-10.56) | -2.416(-3.06--1.767) |
| Saint Lucia | Male | 10.18(8.12-11.92) | 16.03(11.7-20.77) | 57.47 | 26.15(30.86-20.62) | 15.34(19.78-11.27) | -1.789(-2.056--1.52) |
| Saint Vincent and the Grenadines | Male | 5.47(4.11-6.91) | 11.05(8.24-14.04) | 102.01 | 16.86(21.48-12.54) | 15.77(20.03-11.73) | -0.279(-0.507--0.051) |
| Samoa | Male | 8.58(5.22-13.53) | 10.47(5.92-15.84) | 22.03 | 17.41(27.28-10.58) | 12.55(19.13-7.18) | -1.146(-1.232--1.061) |
| San Marino | Male | 2.04(1.24-2.72) | 3.38(1.76-5.04) | 65.69 | 14.44(19.18-8.61) | 11.79(17.68-6.25) | -0.689(-0.78--0.597) |
| Sao Tome and Principe | Male | 16.56(9.86-25.41) | 25.68(17.41-37.09) | 55.07 | 52.78(80.57-31.58) | 45.76(64.1-30.65) | -0.522(-0.906--0.136) |
| Saudi Arabia | Male | 267.47(78.87-604.7) | 258.59(126.31-548.92) | -3.32 | 7.48(16.8-2.43) | 2.63(4.66-1.44) | -3.58(-3.808--3.352) |
| Senegal | Male | 293.38(176.38-490.36) | 485.42(282.95-822.68) | 65.46 | 16.49(27.31-10.14) | 12.15(20.48-7.26) | -1.021(-1.514--0.525) |
| Serbia | Male | 918.82(715.4-1141.54) | 827.82(583.25-1112.7) | -9.90 | 16.23(19.97-12.67) | 12.21(16.41-8.58) | -0.977(-1.349--0.603) |
| Seychelles | Male | 8.26(5.59-11.15) | 18.85(13.53-24.3) | 128.21 | 31.06(42.41-20.71) | 30.6(39.16-21.84) | -0.031(-0.255-0.193) |
| Sierra Leone | Male | 452.67(301.62-662.62) | 470.03(290.83-716.29) | 3.84 | 44.57(65.9-29.69) | 22.64(34.2-14.24) | -2.302(-2.568--2.036) |
| Singapore | Male | 49.84(33.37-66.31) | 55.71(35.54-79.34) | 11.78 | 4.23(5.64-2.81) | 1.36(1.94-0.88) | -3.781(-4.217--3.343) |
| Slovakia | Male | 1011.34(824.06-1176.29) | 1000.14(724.33-1324.59) | -1.11 | 38.36(44.48-31.4) | 25.26(33.5-18.43) | -1.494(-1.93--1.057) |
| Slovenia | Male | 411.85(289.93-554.26) | 293.99(185.3-434.94) | -28.62 | 38.93(52.1-27.48) | 16.32(24.25-10.28) | -2.858(-3.36--2.353) |
| Solomon Islands | Male | 11.13(6.61-17.98) | 28.5(16.52-45.69) | 156.06 | 11.35(18.18-6.83) | 12.27(19.41-7.55) | 0.256(0.149-0.363) |
| Somalia | Male | 232.66(127.44-372.88) | 493.43(277.94-789.17) | 112.08 | 18.37(28.48-10.58) | 15.93(25.1-9.2) | -0.473(-0.598--0.348) |
| South Africa | Male | 2340.06(1711.61-3195.65) | 2901.96(2274.39-3583.1) | 24.01 | 22.27(31.04-16.03) | 13.95(17.23-10.81) | -1.623(-1.983--1.262) |
| South Sudan | Male | 191.24(110.14-357.79) | 251.94(140.81-445.61) | 31.74 | 13.95(25.85-8.09) | 12.25(21.07-7.11) | -0.463(-0.557--0.369) |
| Spain | Male | 6255.4(5156.62-7091.72) | 3997.68(3052.36-4820.41) | -36.09 | 27.64(31.35-22.84) | 10.13(12.14-7.83) | -3.425(-3.58--3.27) |
| Sri Lanka | Male | 1602.3(1060.55-2265.56) | 2442.74(1610.79-3506.71) | 52.45 | 24.88(35.46-16.17) | 21.18(30.15-14.08) | -0.452(-1.065-0.164) |
| Sudan | Male | 405.81(144.16-818.32) | 205.45(95.01-394.03) | -49.37 | 7.84(16.14-2.81) | 2.14(4.11-0.98) | -4.335(-4.555--4.115) |
| Suriname | Male | 31.45(23.68-38.83) | 57.28(39.91-76.37) | 82.13 | 23.41(29.19-17.7) | 19.61(26.31-13.67) | -0.548(-1.139-0.046) |
| Sweden | Male | 404.37(335.6-468.51) | 518.05(425-612.44) | 28.11 | 6.84(7.93-5.67) | 5.8(6.85-4.76) | -0.589(-0.796--0.381) |
| Switzerland | Male | 493.47(410-557.96) | 523.2(416.67-630.4) | 6.02 | 11.62(13.14-9.66) | 6.98(8.35-5.6) | -1.823(-1.946--1.7) |
| Syrian Arab Republic | Male | 239.64(124.93-420.58) | 270.38(102.21-520.76) | 12.83 | 8.16(14.34-4.28) | 4.25(8.12-1.67) | -2.259(-2.628--1.887) |
| Taiwan (Province of China) | Male | 3254.07(2700.43-3757.29) | 3483.63(2399.73-4814.78) | 7.05 | 38.28(44.5-31.39) | 19.72(27.34-13.63) | -2.2(-2.531--1.867) |
| Tajikistan | Male | 294.37(210.31-381.25) | 706.72(495.42-982.32) | 140.08 | 20.64(27.11-14.69) | 23.42(32.48-16.7) | 0.464(0.117-0.813) |
| Thailand | Male | 5232.19(3985.87-6645.62) | 10920.59(7431.25-15176.52) | 108.72 | 25.69(32.5-19.66) | 23.2(32-15.85) | -0.273(-0.467--0.079) |
| Timor-Leste | Male | 21.88(8.95-44.87) | 85.48(39.4-150.11) | 290.68 | 10.35(21.57-4.33) | 19.59(34.63-8.99) | 2.205(1.892-2.519) |
| Togo | Male | 246.75(162.7-359.55) | 447.7(250.98-720.94) | 81.44 | 36.88(54.21-24.03) | 22.95(36.9-13.21) | -1.611(-1.871--1.35) |
| Tokelau | Male | 0.08(0.04-0.13) | 0.07(0.04-0.11) | -12.50 | 13.94(22.36-7.19) | 10.3(15.97-5.54) | -1.035(-1.076--0.993) |
| Tonga | Male | 2.84(1.65-4.55) | 4.19(2.36-7.2) | 47.54 | 9.89(15.45-5.85) | 10.68(18.05-6.14) | 0.28(0.01-0.55) |
| Trinidad and Tobago | Male | 70.19(54.14-86.1) | 91.48(60.49-129.48) | 30.33 | 16.39(20.24-12.6) | 10.15(14.36-6.69) | -1.606(-2.36--0.847) |
| Tunisia | Male | 92.52(50.58-152.17) | 272.1(133.39-474.85) | 194.10 | 3.35(5.49-1.88) | 4.38(7.66-2.18) | 0.954(0.867-1.042) |
| Turkey | Male | 946.63(529.3-1555.2) | 1335.22(797.38-2068.84) | 41.05 | 4.84(7.9-2.67) | 3.07(4.8-1.82) | -1.619(-1.998--1.239) |
| Turkmenistan | Male | 210.63(149.49-283.03) | 1118.1(785.34-1501.81) | 430.84 | 22.01(28.99-16.15) | 49.12(66.12-34.72) | 2.917(1.801-4.046) |
| Tuvalu | Male | 0.58(0.29-0.97) | 0.61(0.31-1.07) | 5.17 | 17.2(28.73-8.96) | 11.25(19.71-5.85) | -1.442(-1.53--1.354) |
| Uganda | Male | 1518.81(1074.25-2152.82) | 2553.75(1624.17-3622.3) | 68.14 | 45.28(64.34-31.88) | 36.26(51.22-22.8) | -0.804(-0.931--0.677) |
| Ukraine | Male | 3364.88(2636.24-4104.47) | 10482.9(7560.96-13814.06) | 211.54 | 12.08(14.68-9.51) | 37.98(49.99-27.62) | 4.262(2.193-6.372) |
| United Arab Emirates | Male | 30.92(18.08-50.89) | 134.92(59.44-253.34) | 336.35 | 9.25(14.8-4.91) | 3.01(5.65-1.35) | -3.858(-4.265--3.45) |
| United Kingdom | Male | 2023.48(1724.2-2304.41) | 4594.88(3894.9-5324.42) | 127.08 | 5.73(6.52-4.89) | 9.32(10.78-7.92) | 1.752(1.512-1.993) |
| United Republic of Tanzania | Male | 2469.04(1794.03-3201.33) | 3927.84(2560.42-5686.14) | 59.08 | 43.94(57.03-31.69) | 30.62(45.04-19.84) | -1.228(-1.365--1.09) |
| United States of America | Male | 16213.25(12913.52-19390.94) | 30299.2(24702.64-35905.73) | 86.88 | 12.17(14.58-9.71) | 12.75(15.08-10.42) | 0.195(0.034-0.356) |
| United States Virgin Islands | Male | 8.11(4.91-11.88) | 14.76(9.2-21.65) | 82.00 | 18.65(27.42-11.36) | 18.86(27.69-11.75) | 0.068(-0.299-0.436) |
| Uruguay | Male | 279.22(223.91-330.11) | 250.4(196.68-304.84) | -10.32 | 16.55(19.54-13.37) | 11.43(13.87-8.97) | -1.354(-1.649--1.058) |
| Uzbekistan | Male | 1145.9(844.45-1493.62) | 4681.12(3423.13-6209.97) | 308.51 | 20.48(26.63-15.44) | 37.24(48.25-28.03) | 2.173(1.753-2.596) |
| Vanuatu | Male | 8.96(4.78-14.67) | 15.39(7.75-26.87) | 71.76 | 19.72(32.11-10.8) | 14.4(24.85-7.26) | -1.089(-1.32--0.857) |
| Venezuela (Bolivarian Republic of) | Male | 1148.4(924.89-1321.69) | 2542.27(1658.73-3540.61) | 121.37 | 22.58(26.21-18) | 17.62(24.53-11.6) | -0.903(-2.302-0.516) |
| Viet Nam | Male | 2147.62(1275.34-3311.6) | 14208.43(9850.24-18828.51) | 561.59 | 12.33(18.96-7.37) | 32.9(42.77-23.18) | 3.447(3.168-3.728) |
| Yemen | Male | 311.23(142.03-548.74) | 307.59(133.44-574.08) | -1.17 | 12.09(22.52-5.28) | 4.12(7.55-1.81) | -3.641(-3.758--3.525) |
| Zambia | Male | 869.33(558.59-1277.24) | 1976.88(1249.51-2840.86) | 127.40 | 51.93(76.87-32.83) | 49.49(71.01-29.97) | -0.155(-0.358-0.048) |
| Zimbabwe | Male | 668.12(459.74-903.09) | 835.02(466.76-1347.78) | 24.98 | 29.87(40.58-20.37) | 22.93(37-12.85) | -0.908(-1.285--0.53) |

**Table S6** The DALYs and age-standardized DALY rate of cirrhosis attributable to alcohol use in 1990 and 2019, and its temporal trends from 1990 to 2019.

| nation | sex | DLAY NO(95% UI) | | Change in absolute number (%) | age-standardized DALY rate per 100,000 No. (95% UI) | | 1990-2019 AAPC No.(95 CI) |
| --- | --- | --- | --- | --- | --- | --- | --- |
|  |  | 1990 | 2019 |  | 1990 | 2019 |  |
| Afghanistan | Both | 4388.41(2124.58-7649.48) | 7109.3(3257.38-12549.73) | 62.00 | 57.39(97.35-29.04) | 46.58(79.18-24.32) | -0.658(-0.784--0.531) |
| Albania | Both | 3312.04(2619.3-4126.8) | 4816.65(3235.47-6829.35) | 45.43 | 143.88(177.83-115.11) | 125.66(178.62-84.61) | -0.46(-0.902--0.017) |
| Algeria | Both | 6897.5(3874.02-11107.69) | 19886.73(11118.38-33229.41) | 188.32 | 48.26(76.93-27.45) | 51.11(85.39-28.37) | 0.21(0.098-0.323) |
| American Samoa | Both | 42.4(23.58-74.49) | 44.44(26.78-74.72) | 4.81 | 132.31(222.47-76.43) | 83.82(140.1-51.15) | -1.543(-1.795--1.291) |
| Andorra | Both | 147.9(90.25-225.9) | 248.1(168.53-341.44) | 67.75 | 248.26(378.52-150.53) | 184.32(253.58-125.83) | -1.026(-1.057--0.995) |
| Angola | Both | 31988.21(19091.59-50367.15) | 133582.46(90502.68-184852.07) | 317.60 | 592.03(922.91-360.49) | 848.74(1166.13-584.87) | 1.258(1.071-1.444) |
| Antigua and Barbuda | Both | 103.54(78.18-132.21) | 208.32(150.21-273.16) | 101.20 | 210.67(270.91-158.71) | 192.21(251.07-139.48) | -0.267(-0.808-0.276) |
| Argentina | Both | 137087.26(115080.95-156836.68) | 164454.97(131029.16-197322.19) | 19.96 | 423.79(484.67-356.42) | 322.39(386.55-256.87) | -0.861(-0.983--0.738) |
| Armenia | Both | 5334.78(4095.5-6851.59) | 13076.03(9778.17-17058.6) | 145.11 | 177.73(225.37-138.81) | 334.66(435.28-250.63) | 2.237(1.634-2.843) |
| Australia | Both | 31817.11(27028.03-35785.46) | 42651.47(35552.5-49465.25) | 34.05 | 170.54(192.09-144.7) | 123.69(142.99-102.5) | -1.059(-1.401--0.716) |
| Austria | Both | 52697.65(43813.78-59619.61) | 35507.51(28594.06-41311.04) | -32.62 | 535.46(603.83-446.06) | 247.91(288.36-200.43) | -2.648(-3.056--2.239) |
| Azerbaijan | Both | 28202.94(21152.99-36017.46) | 54361.82(37963.67-73620.19) | 92.75 | 495.59(635.45-372.88) | 515.69(701.87-359.68) | 0.139(-0.013-0.292) |
| Bahamas | Both | 909.26(710.67-1105.62) | 1129.27(728.92-1607.89) | 24.20 | 487.13(595.3-379.73) | 256.19(361.94-168.36) | -2.207(-2.574--1.84) |
| Bahrain | Both | 725.19(486.35-1006.98) | 1314.66(721.68-2141.25) | 81.28 | 283.97(407.31-179.84) | 82.3(137-46.34) | -4.287(-4.743--3.829) |
| Bangladesh | Both | 156244.35(101168.48-221362.74) | 189906.08(124644.04-271390.71) | 21.54 | 277.69(394.54-181.55) | 134.89(193.69-89.38) | -2.354(-2.518--2.19) |
| Barbados | Both | 595.13(477.66-714.67) | 806.76(580.84-1051.47) | 35.56 | 241.82(288.49-192.94) | 181.98(237.38-133.33) | -0.963(-1.256--0.669) |
| Belarus | Both | 17324.68(13489.06-21327.08) | 53624.57(37563.58-75076.55) | 209.53 | 137.32(169.1-107.89) | 398.49(556.91-278.14) | 3.717(2.49-4.958) |
| Belgium | Both | 36918.7(30635.22-41836.24) | 39298.08(31570.47-46324.72) | 6.44 | 280.13(317.03-233.63) | 217.21(255.22-176.08) | -0.924(-1.186--0.661) |
| Belize | Both | 302.75(232.09-376.76) | 1323.63(1002.81-1682.37) | 337.20 | 293.59(367.61-223.99) | 390.44(497.17-297.03) | 0.985(0.721-1.249) |
| Benin | Both | 12378.15(7755.25-18205.91) | 29130.62(17456.75-44487.19) | 135.34 | 526.87(771.01-331.58) | 444.52(667.93-274.3) | -0.579(-0.747--0.412) |
| Bermuda | Both | 194.5(154.72-232.03) | 133.15(93.51-172.89) | -31.54 | 298.05(356.15-236.58) | 120.59(156.92-84.95) | -3.085(-3.173--2.997) |
| Bhutan | Both | 1878.26(1061.23-3258.53) | 1630.03(863.02-3291.18) | -13.22 | 507.95(873.7-289.96) | 246.5(496.01-135.42) | -2.496(-2.662--2.33) |
| Bolivia (Plurinational State of) | Both | 21230.61(13199.61-31633.48) | 45809.77(30021.94-65601.61) | 115.77 | 559.84(833.04-347.49) | 474.91(682.01-313.81) | -0.555(-0.722--0.389) |
| Bosnia and Herzegovina | Both | 14926.42(11961.68-17884.8) | 10722.51(7656.06-14379.24) | -28.16 | 314.44(376.32-252.76) | 197.83(266.03-141.13) | -1.626(-1.965--1.287) |
| Botswana | Both | 4806.37(2681.12-8033.3) | 9225.94(5582.19-13834.16) | 91.95 | 655.34(1084.27-369.53) | 480.83(707.94-299.84) | -1.024(-1.197--0.851) |
| Brazil | Both | 525411.26(425159.25-636432.55) | 744200.82(609219.05-903045.8) | 41.64 | 456.21(553.21-369.6) | 299.87(363.64-245.85) | -1.429(-1.624--1.233) |
| Brunei Darussalam | Both | 184.24(116.54-275.6) | 284.95(191.68-400.36) | 54.66 | 118.2(176.24-76.09) | 67.17(92.06-46.37) | -1.92(-2.124--1.716) |
| Bulgaria | Both | 50950.93(42729.68-58290.32) | 61955.57(44742.25-80905.05) | 21.60 | 434.74(496.41-363.66) | 573.58(749.04-416.27) | 1.128(0.771-1.486) |
| Burkina Faso | Both | 35874.14(25029.68-48554.99) | 57833.59(30246.74-94039.09) | 61.21 | 715.88(957.13-501.51) | 495.52(788.98-260.76) | -1.256(-1.553--0.958) |
| Burundi | Both | 32477.82(17977.07-50082.3) | 39208.8(21184.37-73795.75) | 20.72 | 1154.1(1762.63-653.57) | 638.88(1186.24-342.9) | -2.029(-2.215--1.842) |
| Cabo Verde | Both | 994.46(646.76-1498.75) | 2210.51(1509.86-3035.46) | 122.28 | 456.68(687.36-301.51) | 450.96(613.55-311.78) | 0.042(-0.313-0.397) |
| Cambodia | Both | 28550.04(17764.6-44863.24) | 163015.07(111053.23-222278.52) | 470.98 | 489.54(751.01-310.64) | 1114.54(1519.94-758.38) | 2.847(2.724-2.969) |
| Cameroon | Both | 44207.85(30637.55-60194.29) | 92959.64(53888.23-144692.74) | 110.28 | 790.86(1076.76-547.12) | 560.5(873.41-330.63) | -1.175(-1.255--1.095) |
| Canada | Both | 58541.56(46289-70117.9) | 92451.4(72091.9-112916.82) | 57.92 | 187.57(224.71-148.61) | 159.14(192.66-125.52) | -0.53(-0.716--0.345) |
| Central African Republic | Both | 12271.2(6590.94-20177.54) | 14662.3(6408.99-27915.21) | 19.49 | 801.37(1300.72-443.07) | 479.7(875.38-221.7) | -1.769(-1.865--1.673) |
| Chad | Both | 12536.68(5890.08-22943.96) | 45851.13(21219.92-73877.49) | 265.74 | 393.92(704.47-189.38) | 611.93(976.94-284.47) | 1.574(1.404-1.743) |
| Chile | Both | 94096.29(77531.96-110299.71) | 102572.08(82092.46-122338.56) | 9.01 | 855.07(1004.01-702.4) | 437.77(522.28-349.41) | -2.326(-2.889--1.759) |
| China | Both | 3016698.59(2173658.42-3915714.86) | 2753844.72(1983749.57-3595344.6) | -8.71 | 298.77(388.64-215.43) | 136.08(177.26-98.24) | -2.7(-2.85--2.549) |
| Colombia | Both | 36625.99(30059.73-43340.83) | 50547.47(34785.91-71100.56) | 38.01 | 171.69(203.02-140.55) | 96.04(134.92-65.93) | -2.048(-2.756--1.336) |
| Comoros | Both | 491.06(201.08-963.57) | 1004.87(575.41-1710.43) | 104.63 | 206.58(398.07-87.93) | 188.4(317.04-109.14) | -0.272(-0.844-0.303) |
| Congo | Both | 10914.8(6405.91-16464.5) | 20318.08(10865.24-31508.51) | 86.15 | 812.13(1204.43-480.81) | 570.08(881.39-307.94) | -1.19(-1.527--0.851) |
| Cook Islands | Both | 18.12(8.67-34.72) | 39.19(26.26-52.93) | 116.28 | 123.36(230.98-60.78) | 170.98(231.05-115.38) | 1.159(1.043-1.275) |
| Costa Rica | Both | 6128.41(5024.05-7188.87) | 14681.03(10080.21-20468.16) | 139.56 | 303.25(357.15-248.55) | 278.29(388.91-191.08) | -0.302(-0.799-0.198) |
| Croatia | Both | 43588.05(35554.76-50971.32) | 21940.68(15691.79-29293.44) | -49.66 | 681.98(796.57-558.86) | 309.29(414.86-221.84) | -2.813(-3.349--2.274) |
| Cuba | Both | 15988.29(12503.26-19941.47) | 34937.86(24344.22-46873.86) | 118.52 | 152.9(190.91-119.76) | 199.69(266.71-138.96) | 1.02(0.203-1.843) |
| Cyprus | Both | 1635.24(1156.26-2189.56) | 2177.17(1701.03-2654.52) | 33.14 | 204.14(275.55-143.26) | 118.52(144.09-92.7) | -1.856(-1.989--1.723) |
| Czechia | Both | 57742.93(48686.66-65655.07) | 52942.16(39705.34-68808.66) | -8.31 | 456.17(518.92-384.41) | 321.97(417.75-241.84) | -1.202(-1.694--0.708) |
| C么te d'Ivoire | Both | 41137.78(25197.68-60916.61) | 84551.39(51482.77-125610.82) | 105.53 | 684.25(1009.85-421.13) | 541.83(806.25-334.08) | -0.812(-0.96--0.664) |
| Democratic People's Republic of Korea | Both | 65072.12(38009.03-98383.79) | 83418.83(42588.08-125242.75) | 28.19 | 330.88(490.58-197.16) | 249(374.05-129.43) | -1.561(-1.898--1.223) |
| Democratic Republic of the Congo | Both | 110791.2(63976.9-164928) | 170192.44(85375.94-296802.21) | 53.62 | 539.39(811.46-313.09) | 341.54(594.75-177.04) | -1.32(-1.577--1.062) |
| Denmark | Both | 20091.43(16726.99-22943.97) | 19069.37(14939.98-22769.25) | -5.09 | 313.12(358.05-260.23) | 211.5(252.46-163.77) | -0.871(-1.036--0.705) |
| Djibouti | Both | 560.77(317.01-1062.11) | 1553.1(873.31-2747.69) | 176.96 | 279.23(515.15-161.76) | 208.71(370.24-118.53) | -1.057(-1.14--0.973) |
| Dominica | Both | 181.13(131.58-234.34) | 183.49(129.35-253.2) | 1.30 | 293.38(381.02-212.72) | 216.55(298.17-151.98) | -0.462(-0.703--0.221) |
| Dominican Republic | Both | 24754.49(19003.23-31121.04) | 46958.6(28046.17-72110.41) | 89.70 | 536.22(672.42-408.12) | 466.33(712.63-278.23) | -0.228(-0.832-0.379) |
| Ecuador | Both | 21695.73(16712.63-27565.41) | 51993.78(36182.86-72090.17) | 139.65 | 337.34(428.8-262.14) | 325.38(449.61-225.53) | -0.464(-0.931-0.006) |
| Egypt | Both | 68767.22(38193.29-115022.99) | 132906.65(61998.9-249055.22) | 93.27 | 217.06(351.85-124.86) | 192.02(350.8-94.27) | -0.43(-1.071-0.215) |
| El Salvador | Both | 16625.95(13004.75-20423.76) | 23503.23(15667.42-33161.1) | 41.36 | 473.73(583.91-369.92) | 397.99(562.91-265.34) | -0.4(-0.691--0.108) |
| Equatorial Guinea | Both | 1125.59(532.57-2044.53) | 2869.45(1590.37-4866.48) | 154.93 | 461.98(840.22-220.82) | 404.52(664.7-230.03) | 0.127(-0.025-0.28) |
| Eritrea | Both | 5085.16(2571.41-8804.19) | 14529.13(8024.61-24721.86) | 185.72 | 375.49(628.53-200.61) | 393.37(657.05-232.37) | 2.964(1.284-4.673) |
| Estonia | Both | 3085.61(2486.33-3761.36) | 6769(4682.84-9101.96) | 119.37 | 162.9(197.75-130.97) | 375.29(502.78-259.3) | -0.472(-0.809--0.134) |
| Eswatini | Both | 2441(1628.39-3451.57) | 4292.04(2591.62-6754.47) | 75.83 | 638.46(903.94-427.7) | 560.92(856.02-347.13) | -0.518(-0.67--0.365) |
| Ethiopia | Both | 157460.36(71314.8-295870.5) | 287184.15(171357.65-422287.19) | 82.39 | 628.97(1175.56-295.29) | 540.64(795.82-334.1) | -0.554(-0.909--0.198) |
| Fiji | Both | 943.72(630.11-1306.61) | 1316.7(810.92-1921.85) | 39.52 | 172.8(238.23-114.84) | 146.35(213.81-91.37) | 0.688(-0.026-1.407) |
| Finland | Both | 15045.38(12513.46-17493.68) | 24338.27(19533.5-28863.04) | 61.77 | 239.79(278.97-199.78) | 297.59(351.21-240.22) | -2.636(-2.902--2.368) |
| France | Both | 308642.58(260125.66-345051.93) | 206061.26(167048.45-237455.21) | -33.24 | 436.84(488.76-367.85) | 201.24(231.28-163.81) | -1.571(-1.821--1.32) |
| Gabon | Both | 7031.37(4662.74-9724.86) | 9094.99(5743.21-13176.75) | 29.35 | 1105.66(1532.82-725.96) | 699.2(1008.23-439.55) | 0.576(0.068-1.086) |
| Gambia | Both | 1840.19(1018.07-3064.08) | 5613.46(3350.63-8683.67) | 205.05 | 404.31(667.29-228.65) | 461.62(707.04-275.01) | 1.106(0.8-1.413) |
| Georgia | Both | 29296.07(22349.31-36458.69) | 32185.57(23987.56-40750.38) | 9.86 | 469.69(584.38-359.67) | 668.23(843.03-495.54) | -1.945(-2.164--1.727) |
| Germany | Both | 542306.21(458265.43-599727.45) | 408465.85(335246.85-470337.55) | -24.68 | 500.83(554.28-426.18) | 284.87(327.01-232.34) | -1.392(-1.526--1.258) |
| Ghana | Both | 66114.15(41392.2-103639.89) | 114710.78(74165.48-166155.46) | 73.50 | 822.07(1298.8-511.37) | 550.05(784.4-357.17) | -1.772(-2.02--1.524) |
| Greece | Both | 27607.7(23121.74-31443) | 20196.79(16664.59-23413.9) | -26.84 | 192.82(219.15-162.57) | 114.76(132.58-95.1) | -0.324(-0.433--0.214) |
| Greenland | Both | 135.57(79.19-200.13) | 180.72(109.19-258.75) | 33.30 | 263.16(387.58-155.1) | 239.44(342.26-145.64) | -1.219(-1.558--0.879) |
| Grenada | Both | 278.73(214.79-341.23) | 377.7(284.42-466.35) | 35.51 | 446.74(548.76-344.98) | 315.52(390.8-236.67) | 0.345(0.006-0.684) |
| Guam | Both | 189.27(91.49-374.55) | 367.82(147.74-655.89) | 94.34 | 178.61(342.76-93.32) | 199.03(354.18-78.73) | -0.738(-1.246--0.228) |
| Guatemala | Both | 44284.63(32805.55-57253.96) | 96646.43(66471.36-131246.25) | 118.24 | 877.11(1136.08-654.85) | 698.67(949.19-481.34) | 0.383(0.251-0.515) |
| Guinea | Both | 11506.28(7503.67-17817.95) | 23451.9(13491.97-37941.13) | 103.82 | 313.54(482.35-203.89) | 350.26(563.7-206.84) | -0.679(-0.764--0.593) |
| Guinea-Bissau | Both | 4956.89(2720-7790.54) | 8276.9(4719.67-12904.53) | 66.98 | 968.33(1509.48-538.08) | 797.44(1224.2-452.56) | -1.111(-1.824--0.392) |
| Guyana | Both | 5218.43(4101.23-6349.45) | 5605.25(4045.85-7447.97) | 7.41 | 1069.64(1301.16-840.81) | 761.49(1005.6-552.14) | -1.206(-1.367--1.044) |
| Haiti | Both | 27064.43(13921.69-39899.82) | 41550.51(22099.5-63874.67) | 53.52 | 688.08(1003.73-366.92) | 478.41(731.56-257.19) | -0.173(-0.425-0.08) |
| Honduras | Both | 15606.98(10631.51-21426.7) | 38795.3(22806.28-59187.1) | 148.58 | 605.84(824.47-416.36) | 572.4(872.52-340.23) | -3.211(-3.78--2.639) |
| Hungary | Both | 156980.34(130546.99-179290.52) | 74800.94(56950.86-95419.71) | -52.35 | 1202.4(1375-1004.66) | 486.05(620.43-368.69) | -0.528(-0.701--0.354) |
| Iceland | Both | 208.7(163.36-261.23) | 328.23(249.76-402.94) | 57.27 | 81.06(101.49-63.15) | 69.51(85.06-53.04) | 0.213(-0.162-0.59) |
| India | Both | 1901529.35(1327728.83-2721771.02) | 4320300.47(2850777.7-6068078.69) | 127.20 | 302.45(431.58-215.62) | 322.39(453.95-213.35) | -0.967(-1.053--0.882) |
| Indonesia | Both | 272249.98(216496.09-347480.87) | 417786.7(322388.73-540152.72) | 53.46 | 225.43(283.21-179.74) | 170.22(215.79-133.25) | 1.554(1.061-2.05) |
| Iran (Islamic Republic of) | Both | 8337.7(5784.27-13186.17) | 36476.05(20084.48-62441.35) | 337.48 | 26.97(41.53-19.17) | 42.97(74.06-23.93) | -3.77(-3.886--3.655) |
| Iraq | Both | 11464.36(6327.51-18711.46) | 11574.53(5838.59-21229.73) | 0.96 | 119.85(196.36-66.63) | 39.5(70.43-20.16) | 0.345(-0.009-0.7) |
| Ireland | Both | 3954.36(3167.98-4662.1) | 7972.16(6221.95-9685.52) | 101.60 | 106.17(124.93-85.26) | 120.66(146.24-94.49) | -0.697(-0.964--0.429) |
| Israel | Both | 3722.49(2903.94-4566.9) | 6913.38(5343.72-8778.69) | 85.72 | 79.87(97.72-62.33) | 65.63(83.35-51.07) | -3.584(-3.871--3.297) |
| Italy | Both | 348977.4(294768.86-389686.31) | 171644.3(134803.71-200858.94) | -50.82 | 435.86(485.87-366.62) | 150.85(174.95-119.43) | -0.683(-1.994-0.645) |
| Jamaica | Both | 1958.97(1511.26-2453.98) | 2598.35(1754.25-3691.27) | 32.64 | 115.29(144.95-88.19) | 86.6(123.12-58.41) | -2.576(-2.83--2.321) |
| Japan | Both | 443236.26(363291.06-526512.02) | 293638.61(230082.34-362412.67) | -33.75 | 263.02(312.23-215.53) | 123.7(149.24-98.15) | -1.158(-1.428--0.887) |
| Jordan | Both | 834.04(438.14-1510.17) | 2912.27(1477.08-4955.02) | 249.18 | 48.12(85.32-25.92) | 34.42(58.33-18.07) | 2.969(2.128-3.818) |
| Kazakhstan | Both | 40714.98(30965.91-51506.79) | 132031.37(97981.44-174205.88) | 224.28 | 289.08(367.09-219.73) | 677.01(886.66-505.64) | -0.81(-0.911--0.708) |
| Kenya | Both | 92903.1(54523.98-160719.53) | 203962.81(123303.84-313413.79) | 119.54 | 916.62(1608.2-536.16) | 723.38(1103.74-442.12) | -1.801(-1.911--1.69) |
| Kiribati | Both | 256.15(88.18-567.05) | 285.95(92.23-693.73) | 11.63 | 501.7(1063.9-191.84) | 294.3(689.51-104.05) | -0.265(-1.402-0.886) |
| Kuwait | Both | 131(81.79-195.68) | 482(292.19-749.09) | 267.94 | 15.72(23.75-9.91) | 14.52(22.4-9.03) | 1.638(0.69-2.596) |
| Kyrgyzstan | Both | 17569.19(13678.63-21450.5) | 48081.79(36584.22-60785.98) | 173.67 | 531(650.27-416.7) | 824.9(1040.65-631.84) | -0.735(-0.904--0.566) |
| Lao People's Democratic Republic | Both | 15013.86(8606.73-22796.12) | 26574.46(16550.8-38428.8) | 77.00 | 564.35(853.06-325.56) | 456.68(663.37-286.71) | 3.055(1.63-4.5) |
| Latvia | Both | 4668.23(3808.01-5558.74) | 8815.85(6352.82-11595.7) | 88.85 | 142.01(168.35-115.97) | 334.11(441.46-240.03) | -2.603(-2.66--2.545) |
| Lebanon | Both | 3508.55(1920.23-5367.81) | 3443.93(1409.15-6206.75) | -1.84 | 140.84(218.68-76.22) | 65.95(118.6-26.99) | 0.23(0.059-0.4) |
| Lesotho | Both | 5245.42(2947.91-8818.55) | 7743.76(4118.97-12302.9) | 47.63 | 457.67(768.06-259.4) | 484.24(763.35-263.76) | -1.39(-1.669--1.11) |
| Liberia | Both | 11813.33(8031.87-16205.32) | 18979.19(11266.92-29634.85) | 60.66 | 951(1302.91-644.86) | 636.97(984.51-375.82) | 0.973(0.471-1.477) |
| Libya | Both | 689.73(364.83-1189.01) | 2846.01(1096.04-5702.84) | 312.63 | 33.58(58.63-17.15) | 44.72(88.8-18.41) | 3.841(2.88-4.811) |
| Lithuania | Both | 7801.44(6201.97-9362.59) | 21072.86(15324.31-27663.39) | 170.12 | 185.22(221.65-146.38) | 555.48(727.52-400.78) | -2.778(-2.979--2.577) |
| Luxembourg | Both | 2314.68(1931.2-2601.8) | 1856.52(1446.56-2261.47) | -19.79 | 467.22(525.5-391.28) | 206.33(250.97-161.41) | -1.498(-1.715--1.281) |
| Madagascar | Both | 30924.32(19461.4-46669.98) | 45608.7(25863-75630.27) | 47.48 | 479.91(729.08-301.06) | 306.91(502.58-177.89) | -0.318(-0.48--0.155) |
| Malawi | Both | 31046.55(18954.11-46118.64) | 55770.16(33213.98-85939.28) | 79.63 | 623.26(922.06-383.46) | 565.06(871.6-335.08) | -0.795(-0.989--0.601) |
| Malaysia | Both | 21432.28(13504.74-31113.39) | 44950.95(23999.08-73682.12) | 109.73 | 186.12(272.34-117.86) | 148.02(241.3-80.41) | -1.702(-1.901--1.503) |
| Maldives | Both | 107.12(56.76-168.69) | 254.88(151.06-436.28) | 137.94 | 95.31(146.87-52.52) | 58.6(95.89-36.96) | -1.183(-1.401--0.964) |
| Mali | Both | 20171.33(12687.81-31345.17) | 30825.04(17863.12-53414.62) | 52.82 | 415.96(644.29-263.24) | 289.71(495.54-168.98) | -1.441(-1.594--1.288) |
| Malta | Both | 663.56(527.29-797.92) | 703.43(540.45-873.92) | 6.01 | 155.74(187.37-123.91) | 101.66(126.19-77.36) | -1.004(-1.107--0.901) |
| Marshall Islands | Both | 96.59(43.47-169.06) | 152.35(64.16-296.84) | 57.73 | 405.24(697.23-196.86) | 302.94(584.57-133.16) | -2.284(-2.543--2.025) |
| Mauritania | Both | 3475.57(2183.14-5018.43) | 3793.57(2117.73-6194.81) | 9.15 | 320.2(461.68-202.83) | 162.8(259.66-92.86) | -2.828(-3.251--2.403) |
| Mauritius | Both | 5610.95(4424.68-6851.11) | 4672.29(3158.38-6292.05) | -16.73 | 619.83(757.12-487.34) | 271.12(363.94-183.26) | -1.117(-1.342--0.893) |
| Mexico | Both | 525503.28(432144-606355.84) | 893843.64(661967.09-1138463.52) | 70.09 | 974.65(1123.8-802.39) | 703.93(896.89-520.57) | -2.042(-2.116--1.969) |
| Micronesia (Federated States of) | Both | 376.28(188.97-634.8) | 306.87(117.57-585.1) | -18.45 | 593.66(990.15-306.98) | 328.54(622.51-127.6) | -0.414(-0.497--0.33) |
| Monaco | Both | 92.07(54.24-140.72) | 106.93(63.28-161.97) | 16.14 | 185.24(285.85-107.63) | 163.62(248.35-97.49) | 0.7(0.562-0.839) |
| Mongolia | Both | 10723.71(7796.76-14057.21) | 32836.66(23589.2-44370.8) | 206.21 | 877.04(1148.47-641.07) | 1076.76(1443.89-787) | -0.456(-0.682--0.23) |
| Montenegro | Both | 918.61(722.76-1129.52) | 1085.79(801.95-1457.9) | 18.20 | 139.58(170.97-109.95) | 121.85(163.61-90.55) | -2.362(-2.515--2.209) |
| Morocco | Both | 15041.97(9155.11-22497.03) | 16125.19(9348.05-26698.53) | 7.20 | 91.22(135.32-55.09) | 45.61(74.14-27.08) | 0.457(0.281-0.634) |
| Mozambique | Both | 9156.59(5898.42-13393.55) | 22105.69(10763.76-43342.23) | 141.42 | 134.69(194.7-87.13) | 152.6(284.52-77.66) | 2.904(2.692-3.115) |
| Myanmar | Both | 96845.16(59761.17-149342.04) | 426795.15(279611.22-597171.98) | 340.70 | 334.4(512.23-204.93) | 757.93(1060.99-497.17) | 0.001(-0.329-0.332) |
| Namibia | Both | 3626.99(1980.78-5776.94) | 7437.25(4490.17-11025.73) | 105.05 | 441.52(695.45-242.47) | 427.82(622.74-265.62) | -0.823(-0.928--0.718) |
| Nauru | Both | 38.94(15.24-67.09) | 35.49(15.02-61.83) | -8.86 | 574.7(973.7-236.1) | 451.55(776.35-201.24) | -0.149(-0.394-0.096) |
| Nepal | Both | 52549.98(36325.63-76492.82) | 105979.69(68018.18-159376.84) | 101.67 | 444.65(652.93-305.67) | 424.01(640.31-276.01) | -1.464(-1.619--1.308) |
| Netherlands | Both | 26479.53(21792.44-30502.68) | 27461.17(21807.99-32546.6) | 3.71 | 144.2(165.71-119.16) | 93.95(111.09-75.21) | -1.303(-1.355--1.251) |
| New Zealand | Both | 3891.89(3324.84-4421.76) | 4977.28(4049.7-5835.14) | 27.89 | 105.44(120.03-90.14) | 73.75(86.45-59.95) | 0.59(-0.088-1.272) |
| Nicaragua | Both | 8455.53(6484.21-10571.5) | 27346.11(19264.93-36769.86) | 223.41 | 440.48(549.76-335.54) | 531.87(716.33-376.16) | -0.828(-0.989--0.667) |
| Niger | Both | 9920.61(6126.36-15293.29) | 21743.45(12723.83-35584.49) | 119.17 | 290.99(447.35-181.92) | 228.64(373.87-134.82) | -0.727(-0.882--0.571) |
| Nigeria | Both | 392780.79(233014.76-611042.34) | 694124.46(418508.04-1129950.63) | 76.72 | 752.13(1169.28-450.45) | 608.16(973.49-370.38) | -1.543(-1.664--1.422) |
| Niue | Both | 8.14(4.23-12.52) | 5.23(2.5-8.01) | -35.75 | 404.41(623.94-209.01) | 258.44(402.19-119.73) | -0.379(-0.62--0.138) |
| North Macedonia | Both | 4443.78(3636.56-5277.87) | 6138.58(4439.95-8243.81) | 38.14 | 217.29(257.36-177.31) | 193.97(259.09-141.51) | -1.526(-1.771--1.281) |
| Northern Mariana Islands | Both | 123.5(47.05-255.59) | 129.6(54.14-249.81) | 4.94 | 347.55(681.29-156.68) | 218.92(422.14-92.15) | -1.576(-1.859--1.293) |
| Norway | Both | 5612.08(4606.9-6633.97) | 5420.23(4315.11-6591.81) | -3.42 | 106.17(125.36-87.31) | 67.43(81.7-53.72) | -0.775(-1.1--0.448) |
| Oman | Both | 386.56(210-655.7) | 951.6(455.81-1825.26) | 146.17 | 46.01(76.02-24.12) | 36.63(62.97-19.54) | 0.233(0.122-0.344) |
| Pakistan | Both | 110975.09(60013.79-194377.57) | 270376.09(155520.27-459103.17) | 143.64 | 169(295.43-90.44) | 181.07(303.53-106.93) | -0.396(-0.493--0.299) |
| Palau | Both | 31.32(11.79-66.86) | 54.6(22.24-108.04) | 74.33 | 248.44(522.9-97.4) | 222.17(431.04-92.52) | -0.358(-0.512--0.205) |
| Palestine | Both | 1080.81(491.8-2114.92) | 2873.73(1560.69-4730.25) | 165.89 | 109.8(217.46-50.2) | 100.1(167.36-53.03) | -0.21(-0.543-0.124) |
| Panama | Both | 3304.43(2627.03-3957.04) | 7677.74(5279.5-10638.62) | 132.35 | 198.6(238.06-156.45) | 183.47(254.46-125.79) | -1.301(-1.426--1.177) |
| Papua New Guinea | Both | 4226.44(2514.05-6307.98) | 7722.6(3892.61-13159.09) | 82.72 | 149.68(224.47-88.3) | 101.96(172.94-54.07) | 0.271(-0.356-0.902) |
| Paraguay | Both | 6416.28(5046.38-7860.57) | 15990.89(10604.48-22494.69) | 149.22 | 249.19(305.72-195.71) | 261.17(368.53-173.05) | -1.438(-2.038--0.835) |
| Peru | Both | 66657.35(48962.31-86444.41) | 104057.52(67967.86-151592.01) | 56.11 | 469.01(607.19-347.65) | 312.86(457.2-204.35) | -0.944(-1.056--0.832) |
| Philippines | Both | 174406.8(132104.12-225516.13) | 311576.51(222061.59-407317.16) | 78.65 | 427.67(566.69-319.15) | 323.73(423.94-229.72) | 0.474(-0.215-1.167) |
| Poland | Both | 125117.66(107672.85-139774.43) | 194835.92(151475.1-246884.16) | 55.72 | 291.15(325.54-250.27) | 351.63(446.64-271.9) | -3.605(-3.88--3.33) |
| Portugal | Both | 84321.26(70287.18-95239.25) | 41025.41(33259.64-47833.1) | -51.35 | 676.03(763.76-563.37) | 234.47(273.14-190.64) | -2.758(-3.302--2.211) |
| Puerto Rico | Both | 21458.09(16476-26100.86) | 14692.45(9842.45-20402.59) | -31.53 | 601.31(731.41-460.86) | 269.23(372.4-178.86) | -2.579(-3.052--2.103) |
| Qatar | Both | 293.63(170.82-466.21) | 1129.48(597.85-1853.44) | 284.66 | 137.98(224.28-78.67) | 66.31(105.04-37.76) | -5.041(-5.3--4.781) |
| Republic of Korea | Both | 440490.36(352443.63-509845.81) | 216908.49(171967.41-262062.9) | -50.76 | 1138.06(1328.14-914.07) | 252.23(304.53-201.61) | -1.165(-2.584-0.274) |
| Republic of Moldova | Both | 72185.12(57361.82-85283.66) | 57492.39(43932.64-71244.48) | -20.35 | 1541.24(1817.5-1235.13) | 1070.49(1329.88-819.99) | 0.236(-0.834-1.317) |
| Romania | Both | 183239.19(151558.14-212412.03) | 206824.55(154983.49-265514.76) | 12.87 | 656.2(758.54-545.09) | 681.97(875.55-512.82) | 4.371(3.263-5.492) |
| Russian Federation | Both | 338354.59(273399.06-400023.5) | 1140412.2(871338.43-1409403.69) | 237.05 | 190.16(224.91-152.91) | 585.66(726.76-446.88) | -2.476(-2.814--2.135) |
| Rwanda | Both | 53002.85(35590.94-71564.18) | 56090.63(37405.48-81821.43) | 5.83 | 1475.65(1972.59-1018.09) | 714.21(1022.87-480.81) | -2.496(-3.062--1.927) |
| Saint Kitts and Nevis | Both | 165.75(109.21-214.86) | 195.17(120.75-315.3) | 17.75 | 527.52(686.68-346.92) | 254.45(409.46-158.6) | -1.939(-2.16--1.716) |
| Saint Lucia | Both | 469.18(369.64-560.52) | 637.17(465.09-822.12) | 35.81 | 517.27(619.18-406.15) | 288.99(372.37-209.99) | -0.13(-0.411-0.152) |
| Saint Vincent and the Grenadines | Both | 212.91(163.35-267.78) | 393.63(292.31-503.5) | 84.88 | 296.58(374.38-226.37) | 287.65(367.11-213.86) | -1.018(-1.077--0.96) |
| Samoa | Both | 348.77(214.64-535.15) | 429.37(248.97-648.52) | 23.11 | 331.66(510.89-205.19) | 247.56(370.94-144.78) | -0.568(-0.678--0.458) |
| San Marino | Both | 77.65(46.35-104.03) | 116.01(60.91-184.79) | 49.40 | 257.98(346.17-152.08) | 216.77(347.56-113.78) | -0.517(-0.819--0.214) |
| Sao Tome and Principe | Both | 645.84(380.96-979.83) | 1067.13(707-1562.39) | 65.23 | 915.5(1385.62-551.15) | 786.89(1135.66-518.95) | -3.864(-4.026--3.702) |
| Saudi Arabia | Both | 9719.27(2815.53-21572.72) | 9780.27(4748.71-20177.92) | 0.63 | 135.6(296.65-42.91) | 43.89(81.65-23.27) | -1.128(-1.69--0.563) |
| Senegal | Both | 12453.78(7699.14-19798.96) | 20514.15(12086.78-33912.85) | 64.72 | 314.27(493.82-197.97) | 224.15(363.65-133.79) | -1.108(-1.499--0.716) |
| Serbia | Both | 33865.26(26904.25-41372.84) | 27281.9(19390.88-36735.25) | -19.44 | 283.59(344.59-225.3) | 204.99(275.72-144.86) | 0.033(-0.223-0.291) |
| Seychelles | Both | 322.09(226.17-425.41) | 723.73(529.75-925.77) | 124.70 | 574.23(759.33-399.96) | 575.61(733.37-422.03) | -1.955(-2.268--1.641) |
| Sierra Leone | Both | 18095.11(12015.6-26530.94) | 22184.56(13362.38-33917.17) | 22.60 | 830.33(1215.61-553.15) | 467.91(711.65-289.7) | -3.534(-3.857--3.21) |
| Singapore | Both | 1990.92(1362.17-2647.03) | 2124.4(1399.49-2988.24) | 6.70 | 72.83(97.44-49.4) | 25.71(36.04-17.05) | -1.499(-1.827--1.169) |
| Slovakia | Both | 40915.21(33468.7-48400.67) | 38365.63(27909.96-50974.77) | -6.23 | 714.91(847.87-582.47) | 475.28(634.66-342.23) | -3.427(-3.859--2.993) |
| Slovenia | Both | 18194.36(12484.08-24955.12) | 9584.56(6319.26-14171.73) | -47.32 | 759.11(1040.81-523.2) | 273.62(404.89-178.79) | 0.101(-0.017-0.219) |
| Solomon Islands | Both | 537.13(322.46-831.17) | 1399.62(829.11-2158.21) | 160.57 | 273.75(422.85-164.32) | 282.36(432.14-171.92) | -0.601(-0.746--0.455) |
| Somalia | Both | 10132.34(5696.94-16048.15) | 22387.27(12821.15-36920.9) | 120.95 | 325.52(508.65-186.28) | 272.05(443.11-156.56) | -2.107(-2.659--1.552) |
| South Africa | Both | 118148.35(88298.81-158619.5) | 127307(98641.01-158793.95) | 7.75 | 439(596.6-324.33) | 241.91(303.66-187.63) | -0.678(-0.771--0.585) |
| South Sudan | Both | 6988.42(4130.46-12155.89) | 9710.35(5485.84-16804.41) | 38.95 | 264.57(455.89-158.73) | 217.81(364.49-125.76) | -3.642(-3.779--3.506) |
| Spain | Both | 233705.03(191080.24-269666.53) | 129712.46(100019.48-158046.21) | -44.50 | 484.81(558.17-398.62) | 167.04(201.53-130.54) | -0.92(-1.6--0.235) |
| Sri Lanka | Both | 63178.43(42884.9-87506.75) | 84932.94(56128.36-120646.99) | 34.43 | 451.46(628.15-305.29) | 331.4(470.22-220.53) | -4.473(-4.729--4.217) |
| Sudan | Both | 15348.73(5771.3-30457.4) | 7403.04(3794.67-13597.84) | -51.77 | 138.03(273.23-53.07) | 36.32(67.28-18.52) | -0.931(-1.475--0.384) |
| Suriname | Both | 1349.67(1014.67-1686.35) | 2212.06(1572.13-2988.88) | 63.90 | 457.7(574.51-346.91) | 346.85(463.17-248.09) | -0.812(-1.079--0.545) |
| Sweden | Both | 16068.24(13294.38-18740.17) | 17919.96(14600.07-21262.89) | 11.52 | 136.67(159.3-112.86) | 107.98(127.71-88.15) | -1.806(-2.064--1.547) |
| Switzerland | Both | 19972.85(16698.45-22461.84) | 18970.5(15159.49-22664.21) | -5.02 | 222.7(251.54-187.32) | 131.23(155.05-105.24) | -2.389(-2.78--1.997) |
| Syrian Arab Republic | Both | 8842.75(4715.8-15413.37) | 9283.33(4067.42-17233.36) | 4.98 | 134.5(232.58-72) | 67.25(124.14-29.14) | -2.235(-2.564--1.905) |
| Taiwan (Province of China) | Both | 120048.56(98325.78-141616.96) | 119859.92(83838.36-164311.12) | -0.16 | 659.98(780.3-535.97) | 339.35(462.36-238.08) | 0.501(0.22-0.783) |
| Tajikistan | Both | 14850.77(11337.23-18730.82) | 36675.09(26673.52-49273.4) | 146.96 | 446.93(564.69-342.6) | 513.12(684.54-377.83) | -0.393(-0.572--0.213) |
| Thailand | Both | 235009.64(179068.96-298058.27) | 427861.13(294450.92-595395.42) | 82.06 | 494.51(623.94-379.57) | 438.6(609.21-301.79) | 1.509(1.213-1.807) |
| Timor-Leste | Both | 1024.27(472.65-1980.98) | 3169.04(1454.53-5311.36) | 209.39 | 226.36(435.83-109.1) | 348.69(586.59-163.79) | -1.695(-1.956--1.433) |
| Togo | Both | 11346.92(7604.48-16443.53) | 20668.41(11699.35-33115.32) | 82.15 | 675.81(983.9-451.43) | 410.26(655.34-238.88) | -0.802(-0.891--0.713) |
| Tokelau | Both | 3.3(1.79-5.12) | 2.74(1.57-4.16) | -16.97 | 262.7(414.15-140.53) | 206.81(315.48-115.79) | 0.181(-0.018-0.379) |
| Tonga | Both | 118.65(71.03-184.61) | 166.41(92.71-277.94) | 40.25 | 187.72(289.84-113.64) | 196.65(324.07-110.35) | -1.458(-2.118--0.794) |
| Trinidad and Tobago | Both | 2912.1(2252.2-3602.23) | 3536.21(2324.73-5083.45) | 21.43 | 309.18(383.87-238.62) | 195.25(279.24-129.55) | 0.63(0.549-0.712) |
| Tunisia | Both | 3937.16(2219.69-6203.79) | 10072.2(5224.83-17485.89) | 155.82 | 64.14(101.54-36.93) | 76.6(132.72-40.42) | -1.969(-2.287--1.65) |
| Turkey | Both | 40239.29(22871.04-65553.82) | 49987.57(30394.35-76688.97) | 24.23 | 93.87(153.97-53.31) | 53.75(82.41-32.77) | 3.072(2.131-4.021) |
| Turkmenistan | Both | 11039.41(8286.36-14181.48) | 55061.55(39412.36-72995.65) | 398.77 | 475.85(609.67-358.64) | 1100.71(1451.44-788.47) | -1.014(-1.062--0.966) |
| Tuvalu | Both | 24.93(12.29-40.82) | 26.03(13.45-45.09) | 4.41 | 313.89(516.67-157.13) | 233.05(401.45-120.92) | -0.732(-0.866--0.598) |
| Uganda | Both | 61251.1(42844.26-86011.03) | 120986.66(81063.84-169739.22) | 97.53 | 784.12(1096.69-551.41) | 638.4(872.91-429.85) | 5.186(2.697-7.735) |
| Ukraine | Both | 160321.35(125775.23-196091.92) | 555372.07(412128.14-706052.24) | 246.41 | 243.83(298.25-192.05) | 962.69(1221.43-716.59) | -3.296(-3.787--2.803) |
| United Arab Emirates | Both | 1257.5(744.17-2009.14) | 5324.91(2450.95-9838.65) | 323.45 | 173.32(273.21-100.52) | 66.4(121.52-31.32) | 1.758(1.511-2.005) |
| United Kingdom | Both | 94533.78(79717.79-108019.77) | 199843.44(168006.22-233794.97) | 111.40 | 131.95(151.05-111.17) | 215.71(252.2-180.82) | -1.211(-1.356--1.065) |
| United Republic of Tanzania | Both | 104173.01(73584.55-137239.71) | 173193.27(109961.86-248948.78) | 66.26 | 807.32(1061.93-571.35) | 563.62(815.65-356.92) | 0.219(0.043-0.396) |
| United States of America | Both | 734383.23(582293.14-889239.93) | 1278637.14(1024888.07-1532043.77) | 74.11 | 258.61(311.64-205.22) | 275.02(328.47-220.41) | -0.452(-0.786--0.117) |
| United States Virgin Islands | Both | 352.33(204.82-533.55) | 502.45(307.35-748.17) | 42.61 | 350.46(528.6-206.04) | 307.52(461.97-185.23) | -1.678(-1.985--1.37) |
| Uruguay | Both | 9850.63(7748.03-11796.68) | 7910.45(6088.61-9754.13) | -19.70 | 271.29(325.17-212.87) | 169.9(209.55-130.86) | 2.268(1.898-2.639) |
| Uzbekistan | Both | 58021.18(44688.9-72178.78) | 248478.08(184729.48-322492.32) | 328.25 | 440.64(546.39-338.11) | 826.93(1066.41-624.02) | -1.137(-1.309--0.965) |
| Vanuatu | Both | 387.55(205.51-628.52) | 667.56(340.91-1158.62) | 72.25 | 410.99(658.92-227.28) | 297.14(510.57-155.83) | 2.966(2.726-3.207) |
| Venezuela (Bolivarian Republic of) | Both | 45958.48(37043.3-53797.87) | 88293.13(57814.4-123087.83) | 92.11 | 391.95(458.72-314.9) | 284.6(397-187.52) | -3.539(-3.694--3.384) |
| Viet Nam | Both | 83997.86(52054.84-127781.19) | 492778.7(340246.42-660988.02) | 486.66 | 198.32(303.42-123.65) | 458.92(609.19-320.09) | -0.289(-0.437--0.141) |
| Yemen | Both | 12622.61(6037.97-22388.15) | 12521.59(5732.45-23323.51) | -0.80 | 205.41(359.31-97.65) | 72.12(132.6-34.3) | -0.91(-1.348--0.47) |
| Zambia | Both | 38907.63(24887.24-54523.8) | 91510.78(56071.57-135382.07) | 135.20 | 1033.19(1463.91-659.12) | 937.04(1382.96-571.1) | -0.482(-0.6--0.364) |
| Zimbabwe | Both | 26143.99(18453.25-35027.52) | 35996.96(19746-58910.04) | 37.69 | 496.46(661.26-347.23) | 378.11(606.83-210.46) | -1.782(-2.149--1.414) |
| Afghanistan | Female | 1459.94(592.96-2712.77) | 2479.49(975.61-4821.3) | 69.84 | 39.02(69.71-17.08) | 32.8(60.35-14.43) | -1.017(-1.193--0.84) |
| Albania | Female | 904.7(730.15-1091.89) | 984.26(673.49-1381.06) | 8.79 | 78.93(95.32-63.29) | 47.97(67.89-33) | -0.228(-0.435--0.022) |
| Algeria | Female | 1249.05(739.12-1945.95) | 2425.48(1481.21-3851.83) | 94.19 | 18.57(29.09-11.09) | 13.74(21.19-8.46) | -0.83(-0.905--0.755) |
| American Samoa | Female | 4.54(3-6.61) | 8.49(5.23-12.52) | 87.00 | 34.53(51.01-22.44) | 32.08(47.28-20.42) | 2.482(2.229-2.737) |
| Andorra | Female | 35.76(19.83-57.4) | 65.53(41.14-98.53) | 83.25 | 127.9(206.23-70.47) | 99.74(149.69-61.91) | 0.462(0.195-0.729) |
| Angola | Female | 5372.22(2720.83-9727.65) | 33957.84(20436.79-52237.03) | 532.10 | 200.23(349.15-103.91) | 402.42(613.9-247.33) | -1.657(-1.917--1.397) |
| Antigua and Barbuda | Female | 20.33(15.29-26.64) | 47.17(31.84-65.63) | 132.02 | 74.14(98.06-54.89) | 84.25(116.91-57.51) | 1.693(1.199-2.189) |
| Argentina | Female | 31711.86(24810.94-38754.24) | 31356.16(21966.91-41697.97) | -1.12 | 184.52(225.64-144.18) | 112.49(148.64-79.47) | -0.865(-1.222--0.507) |
| Armenia | Female | 1446.54(1068.97-1916.67) | 3267.31(2430.17-4332.32) | 125.87 | 89.37(115.96-66.82) | 141.94(187.9-105.34) | -2.626(-3.027--2.224) |
| Australia | Female | 7843.44(6211.02-9450.18) | 11270.96(8449.06-14276.22) | 43.70 | 81.01(97.46-64.23) | 63.22(79.53-47.19) | -0.014(-0.137-0.109) |
| Austria | Female | 13223.95(10238.19-16103.98) | 8707.84(6516.36-10950.25) | -34.15 | 242.96(294.93-190.98) | 114.98(144.28-86.97) | -2.843(-3.251--2.434) |
| Azerbaijan | Female | 8295.79(5456.7-12464.48) | 13836.63(8650.65-22445.77) | 66.79 | 263.61(392.95-175.2) | 259.01(414.85-162.74) | -6.077(-6.413--5.74) |
| Bahamas | Female | 211.12(145.13-291.08) | 210.74(136.54-322.28) | -0.18 | 210.41(290.68-143.09) | 92.42(141.47-59.7) | -1.826(-2.328--1.321) |
| Bahrain | Female | 101.63(55.04-168.24) | 69.9(36.58-122.42) | -31.22 | 101.42(170.35-54.44) | 16.53(27.75-9.09) | -0.667(-1.347-0.018) |
| Bangladesh | Female | 31321.47(18431.54-48428.12) | 47612.84(29540.69-74402.28) | 52.01 | 123.02(189.92-71.82) | 69.01(107.19-42.75) | 4.001(3.025-4.986) |
| Barbados | Female | 81.06(60.48-104.68) | 115.9(78.17-166.56) | 42.98 | 57.09(73.56-42.73) | 47.38(67.25-32.11) | -1.142(-1.461--0.823) |
| Belarus | Female | 5053.98(3575.85-6788.34) | 16443.3(10395.69-23651.42) | 225.35 | 70.79(94.24-49.49) | 219.23(314.23-141.56) | -0.174(-0.528-0.182) |
| Belgium | Female | 13431.87(10704.58-15806.09) | 12971.93(9982.98-16056.23) | -3.42 | 187.35(219.85-151.08) | 135.5(166.93-105.8) | -0.151(-0.253--0.049) |
| Belize | Female | 68.22(51.25-89.52) | 209.82(150.08-283.46) | 207.56 | 134.19(176.8-100.1) | 127.12(170.7-91.65) | -4.378(-4.594--4.162) |
| Benin | Female | 2461.27(1513.82-3913.15) | 6527.05(3523.71-11306.04) | 165.19 | 198.12(315.33-123.08) | 189(317.98-106.68) | -0.974(-1.351--0.596) |
| Bermuda | Female | 41.66(28.8-56.52) | 19.29(11.99-29.3) | -53.70 | 118.58(160.54-81.79) | 32.37(48.2-20.47) | -0.523(-0.684--0.362) |
| Bhutan | Female | 170.94(96.1-270) | 250.54(154.92-382.1) | 46.57 | 109.55(172.26-61.67) | 83.88(127.88-52.5) | -1.636(-2.248--1.02) |
| Bolivia (Plurinational State of) | Female | 4919.6(2692.26-7668.33) | 10648.81(6703.41-15594.89) | 116.46 | 256.84(402.97-142.6) | 219.31(318.48-139.21) | -0.953(-1.281--0.625) |
| Bosnia and Herzegovina | Female | 2372.9(1830.79-2925.9) | 1778.91(1248.85-2526.79) | -25.03 | 96.37(118.3-75.28) | 58.81(84.32-41.19) | -1.252(-1.452--1.051) |
| Botswana | Female | 857.93(387.97-1601.07) | 1639.61(774.15-2858.9) | 91.11 | 206.68(379.38-94.42) | 157.2(272.62-75.35) | -1.839(-1.919--1.758) |
| Brazil | Female | 69927.36(54661.12-90315.68) | 107192.6(82003.21-140898.85) | 53.29 | 118.71(152.5-94.2) | 82.75(108.47-63.31) | 0.882(0.596-1.169) |
| Brunei Darussalam | Female | 46.64(27.79-73.33) | 76.66(50.34-110.31) | 64.37 | 68.2(106.16-41.32) | 39.71(57.12-26.53) | -1.009(-1.254--0.763) |
| Bulgaria | Female | 7874.22(6066.61-10056.88) | 9155.84(6172.09-13010.48) | 16.28 | 128.12(162.15-98.83) | 160.46(230.16-106.95) | -3.16(-3.397--2.923) |
| Burkina Faso | Female | 11645.94(7168.89-16760.66) | 20229.6(7548.53-34444.39) | 73.71 | 429.64(615.14-265.24) | 314.82(541.72-113.35) | -1.033(-1.159--0.907) |
| Burundi | Female | 8499.74(4254.81-14861.88) | 6367.43(2731.48-12129.79) | -25.09 | 566.37(986.07-285.96) | 223.6(424.32-96.51) | 1.238(1.114-1.363) |
| Cabo Verde | Female | 277.02(159.31-449.12) | 399.52(231.6-605.81) | 44.22 | 212.17(339.5-123.29) | 157.22(239.69-90.98) | -1.34(-1.467--1.213) |
| Cambodia | Female | 9296.19(6019.01-13183.74) | 33709.54(18881.32-55779.01) | 262.62 | 303.18(431.85-199.05) | 431.92(714.59-241.72) | -0.138(-0.456-0.182) |
| Cameroon | Female | 11962.44(7407.7-17948.54) | 23418.58(11218.09-40607.58) | 95.77 | 414.08(621.43-254.18) | 279.45(487.49-133.14) | -1.141(-1.448--0.832) |
| Canada | Female | 16550.67(12517.89-21287.66) | 28684.68(21089.6-37111.53) | 73.31 | 99.97(128.32-75.98) | 96.07(121.69-71.18) | 1.348(1.246-1.451) |
| Central African Republic | Female | 2179.09(1157.27-3825.82) | 2980.73(1355.96-6131.89) | 36.79 | 260.95(458.1-135.23) | 188.35(376.78-87.25) | -2.492(-3.013--1.969) |
| Chad | Female | 3812.56(1725.57-7374.83) | 11877.94(4418.39-21214.67) | 211.55 | 226.5(428.98-105.96) | 327.32(578.83-129.92) | -3.173(-3.346--2.999) |
| Chile | Female | 21109.52(15821.3-27050.54) | 22506.73(16003.26-29772.64) | 6.62 | 365.72(470.24-273.15) | 178.75(234.8-127.26) | -1.865(-2.17--1.56) |
| China | Female | 275939.42(182773.74-407415.74) | 248599.94(152609.98-402636.13) | -9.91 | 59.87(88.17-39.82) | 23.81(38.38-14.63) | -0.242(-0.724-0.243) |
| Colombia | Female | 7982.56(6245.77-10164.79) | 12038.33(8201.2-16892.18) | 50.81 | 73.34(93.07-57.53) | 42.47(59.87-29.06) | 0.075(-0.312-0.464) |
| Comoros | Female | 144.18(59.77-249.37) | 304.27(144.35-505.96) | 111.03 | 119.29(204.69-50.94) | 109.73(182.45-51.91) | -0.21(-0.355--0.064) |
| Congo | Female | 2197.07(1156.53-3661.69) | 5526.98(2501.92-9927.6) | 151.56 | 304.55(503.49-159.94) | 308.98(560.49-141.97) | -0.046(-0.817-0.731) |
| Cook Islands | Female | 1.04(0.61-1.6) | 1.79(0.98-2.96) | 72.12 | 16.06(24.31-9.44) | 15.12(25.15-8.14) | -3.843(-4.47--3.211) |
| Costa Rica | Female | 1071.79(825.84-1380.66) | 2898.2(1999.61-4029.65) | 170.41 | 107.85(137.21-82.1) | 103.94(144.79-71.82) | -1.001(-1.305--0.695) |
| Croatia | Female | 9345.31(6852.19-12222.29) | 3342.82(2330.64-4760.95) | -64.23 | 267.58(351.23-194.74) | 84.44(123.53-57.89) | -2.578(-2.967--2.188) |
| Cuba | Female | 4157.91(3104.2-5467.52) | 5346.91(3578.74-7774.05) | 28.60 | 78.59(103.96-58.32) | 58.14(84.51-39.03) | -0.361(-0.741-0.022) |
| Cyprus | Female | 414.6(269.54-632.9) | 455.35(319.55-628.02) | 9.83 | 100.99(155.74-65.49) | 47.86(65.4-33.56) | -0.207(-0.312--0.102) |
| Czechia | Female | 12452.65(9743.97-15264.74) | 13852.68(10074.89-18548.03) | 11.24 | 179.18(218.14-141.74) | 163.54(219.93-119.07) | -1.331(-1.472--1.19) |
| C么te d'Ivoire | Female | 5677.26(3015.14-9784.98) | 13896.38(6655.48-24729.11) | 144.77 | 213.45(358.19-115.71) | 199.6(355.4-95.04) | -1.742(-2.077--1.407) |
| Democratic People's Republic of Korea | Female | 6506.63(3331.87-11062.65) | 7617.68(3991.1-13306.98) | 17.08 | 62(105.79-32.28) | 41.92(73.2-21.7) | -1.137(-1.458--0.815) |
| Democratic Republic of the Congo | Female | 18206.42(9648.42-31031.31) | 24664.39(13106.28-44517.8) | 35.47 | 161.08(274.06-85.9) | 96.95(178.78-52.28) | -0.814(-0.99--0.637) |
| Denmark | Female | 5660.66(4567.63-6708.98) | 5736.81(4305.04-7171.58) | 1.35 | 169.98(200.87-137.04) | 121.86(151-91.64) | -1.665(-1.781--1.55) |
| Djibouti | Female | 100.45(57.71-164.14) | 295.42(142.21-545.36) | 194.10 | 120.27(194.59-70.14) | 92.63(169.05-46.09) | -0.871(-1.402--0.337) |
| Dominica | Female | 53.33(35.2-76.36) | 39.47(25.03-59.43) | -25.99 | 152.72(218.07-101.17) | 95.43(143.75-60.12) | 0.217(-0.312-0.749) |
| Dominican Republic | Female | 6550.42(4813.16-8627.01) | 10961.84(6777.94-16643.33) | 67.35 | 272.25(356.28-197.19) | 214.23(323.13-133.34) | -0.213(-0.639-0.215) |
| Ecuador | Female | 3896.4(2910.19-5076.69) | 11438.18(7977.52-15924.48) | 193.56 | 128.44(170.06-95.68) | 141.82(196.82-98.6) | -0.285(-0.477--0.094) |
| Egypt | Female | 14427.3(9067.22-22263.68) | 25557.21(13673.51-44650.18) | 77.14 | 103.02(158.23-65.62) | 95.53(165.72-51.53) | 1.353(0.896-1.812) |
| El Salvador | Female | 2405.95(1802.22-3076.91) | 4102.48(2688.48-5875.17) | 70.51 | 141.66(181.27-105.88) | 122.62(176.06-80.43) | 0.372(0.223-0.521) |
| Equatorial Guinea | Female | 205.43(91.69-400.73) | 810.76(386.6-1419.68) | 294.66 | 149.54(289.69-65.13) | 217.59(377.97-103.74) | 3.054(1.433-4.702) |
| Eritrea | Female | 1133.91(521.62-2004.35) | 3263.8(1911.26-5146.73) | 187.84 | 166.7(297.54-79.57) | 185.36(289.93-110.72) | -0.934(-1.33--0.537) |
| Estonia | Female | 768.93(552.32-1046.19) | 1628.86(1068.34-2298.76) | 111.83 | 72.12(97.1-51.58) | 170.7(240.81-111.71) | -1.06(-1.236--0.883) |
| Eswatini | Female | 482.1(270.4-816.07) | 730.75(325.21-1380.48) | 51.58 | 229.3(379.75-127.19) | 177.42(327.35-80.79) | -0.314(-0.524--0.103) |
| Ethiopia | Female | 33032.92(16223.93-62572.18) | 49623.59(26310.52-85917.66) | 50.22 | 275.54(513.96-143.15) | 199.8(336.99-113.38) | 0.84(0.344-1.339) |
| Fiji | Female | 86.69(54.26-125.04) | 145.49(87.97-228.85) | 67.83 | 36.86(53.47-23.07) | 33.56(52.37-20.81) | -3.023(-3.245--2.8) |
| Finland | Female | 3151.71(2414.82-4007.93) | 5137.5(3683.38-6663.79) | 63.01 | 93.48(118.4-71.37) | 120.22(155.36-86.72) | -1.191(-1.46--0.922) |
| France | Female | 89145.16(70919.27-104392.95) | 55431.9(41922.02-68045.04) | -37.82 | 240.09(280.73-192.11) | 99.87(122.82-75.47) | 1.048(0.432-1.667) |
| Gabon | Female | 1352.84(883.91-2020.66) | 1924.67(1085.73-3103.65) | 42.27 | 406.43(618.93-262.95) | 289.02(466.43-161.74) | -1.064(-1.99--0.13) |
| Gambia | Female | 296.61(154.48-489.42) | 1115.65(563.61-2081.51) | 276.13 | 144.55(233.41-77.28) | 184.53(345.27-94.9) | -2.044(-2.333--1.753) |
| Georgia | Female | 4929.12(3834.52-6108.69) | 3142.84(2298.49-4134.9) | -36.24 | 134.49(165.9-105.43) | 99.21(129.75-72.82) | -2.037(-2.243--1.83) |
| Germany | Female | 173987.57(142437.7-197039.99) | 125225.13(99826.21-148726.33) | -28.03 | 294.91(334.61-243.26) | 163.33(192.6-130.15) | -2.75(-3.005--2.495) |
| Ghana | Female | 14850.35(7732.49-25664.26) | 21857.43(10842.2-37234.41) | 47.18 | 348.52(603.88-181.12) | 191.21(319.01-95.11) | -0.745(-0.879--0.611) |
| Greece | Female | 5765.9(4273.4-7456.18) | 3292.19(2478.79-4201.63) | -42.90 | 74.7(95.48-56.22) | 33.34(42.15-25.46) | -1.858(-2.203--1.512) |
| Greenland | Female | 62.94(33.81-98.01) | 71.75(37.82-109.57) | 14.00 | 269.45(420.88-145.04) | 217.58(331.96-117.45) | -1.068(-1.657--0.475) |
| Grenada | Female | 59.04(42.23-81.69) | 56.4(38.16-79.62) | -4.47 | 166.07(230-117.82) | 96.42(135.26-64.94) | -1.168(-1.786--0.547) |
| Guam | Female | 19.06(12.39-27.06) | 31.4(20.05-48.2) | 64.74 | 45.33(64.85-30.02) | 33.24(51.5-21.11) | -0.679(-0.906--0.451) |
| Guatemala | Female | 8278.75(5825.04-11321.59) | 16177.68(11132.04-22523.66) | 95.41 | 341.07(456.96-243.17) | 242.2(333.05-166.33) | -0.516(-0.622--0.41) |
| Guinea | Female | 2831.36(1636.06-4523.82) | 4087.43(2100.06-7079.62) | 44.36 | 157.84(252.18-92.86) | 129.11(226.59-66.55) | -1.173(-2.149--0.187) |
| Guinea-Bissau | Female | 1106.73(562.08-1964.82) | 1926.09(954.28-3395.16) | 74.03 | 397.34(703.11-204.02) | 344.16(596.16-180.21) | -1.372(-1.561--1.182) |
| Guyana | Female | 516.29(371.29-681.54) | 571.73(387.84-800.93) | 10.74 | 220.59(286.99-158.86) | 153.61(213.37-104.72) | 0.686(0.108-1.267) |
| Haiti | Female | 6222.52(2793.96-10804.21) | 9345.27(4051.7-17831.02) | 50.18 | 308.55(511.86-149.3) | 205.45(374.61-91.77) | -3.912(-4.488--3.333) |
| Honduras | Female | 3699.88(2631.02-5005.92) | 12024.03(6557.58-19109.68) | 224.98 | 293.24(394.24-207.22) | 342.25(537.06-188.51) | -1.136(-1.229--1.043) |
| Hungary | Female | 34352.69(25397.62-43724.22) | 13400.64(9340.14-18842.35) | -60.99 | 488.63(621.92-359.26) | 158.05(223.67-109.61) | -0.568(-1.336-0.206) |
| Iceland | Female | 59.34(44.4-78.06) | 76.46(52.5-103.23) | 28.85 | 45.83(60.26-34.21) | 33(44.45-22.93) | -1.137(-1.291--0.983) |
| India | Female | 220791.71(160722.17-312636.85) | 434372.73(307974.2-614031.62) | 96.73 | 83.81(118.19-61.42) | 68.77(96.73-49.01) | -0.324(-0.586--0.061) |
| Indonesia | Female | 96126.65(74420.92-120800.65) | 137491.05(102641.73-183614.61) | 43.03 | 160.86(203.06-123.92) | 114.23(149.43-85.07) | -2.946(-3.091--2.801) |
| Iran (Islamic Republic of) | Female | 1750(1294.54-2340.93) | 4542.44(2926.23-8148.76) | 159.57 | 12.96(17.89-9.33) | 11.69(21.16-7.6) | 0.115(-0.279-0.51) |
| Iraq | Female | 1361.09(729.4-2465.91) | 1543.73(899.93-2459.03) | 13.42 | 27.24(48.41-14.78) | 11.41(18.03-6.79) | -1.197(-1.472--0.922) |
| Ireland | Female | 1436.82(1088.46-1793.46) | 2557(1894.38-3270.28) | 77.96 | 74.4(92.34-56.34) | 75.92(96.43-57.1) | -3.809(-4.072--3.545) |
| Israel | Female | 1129.14(830.09-1457.23) | 1856.94(1392.15-2407.61) | 64.46 | 44.46(56.78-33.26) | 32.04(41.24-24.28) | -0.414(-1.353-0.534) |
| Italy | Female | 102628.23(81096-122911.6) | 49952.54(34743.47-66436.77) | -51.33 | 228.02(271.48-181.35) | 73.82(95.71-51.63) | -2.624(-3--2.246) |
| Jamaica | Female | 403.52(303.05-523.12) | 590.33(391.47-851.42) | 46.30 | 43.95(57.47-32.63) | 38.39(55.72-25.28) | -2.132(-2.451--1.812) |
| Japan | Female | 100890.79(75880.57-128103.33) | 73478.39(51437.15-100984.93) | -27.17 | 111.53(141.33-84.71) | 51.76(67.62-38.52) | 2.373(1.488-3.265) |
| Jordan | Female | 151.22(91.91-241.15) | 374.56(219.16-612.34) | 147.69 | 21.07(33.52-12.67) | 11.29(18.47-6.66) | -1.174(-1.304--1.044) |
| Kazakhstan | Female | 13772.98(9716.74-19114.2) | 35583.9(26552.02-48487.76) | 158.36 | 174.55(242.05-123.01) | 337.25(458.93-252.45) | -1.334(-1.481--1.188) |
| Kenya | Female | 21500.82(11179.13-38675.63) | 41481.2(20756.88-74505.57) | 92.93 | 433.94(768.49-230.12) | 307.33(543.39-157.67) | -1.143(-1.605--0.68) |
| Kiribati | Female | 28.95(15.94-45.07) | 37.82(18.09-65.23) | 30.64 | 124.94(193.38-71.8) | 84.33(139.45-43.81) | 0.829(0.05-1.613) |
| Kuwait | Female | 22.25(13.93-33.15) | 73.6(44.9-115.03) | 230.79 | 8.37(13.26-5.12) | 6(9.57-3.63) | -0.826(-1.003--0.648) |
| Kyrgyzstan | Female | 4406.21(3417.9-5535.27) | 9008.43(6773.47-12180.32) | 104.45 | 243.89(306.73-186.18) | 304.34(405.51-231.43) | 3.09(1.891-4.304) |
| Lao People's Democratic Republic | Female | 2942.35(1358.2-5454.14) | 4893.75(2798.91-7869.99) | 66.32 | 210.26(381.35-101.97) | 166.29(268.97-95.33) | -4.011(-4.089--3.933) |
| Latvia | Female | 1302.71(976.13-1681.43) | 2414.23(1540-3579.06) | 85.32 | 69.07(88.71-51.81) | 169.29(255.16-106.77) | 0.567(0.211-0.925) |
| Lebanon | Female | 532(251.87-996.23) | 375.5(178.35-682.38) | -29.42 | 43.46(82.18-20.36) | 13.27(24.12-6.35) | -1.448(-1.711--1.185) |
| Lesotho | Female | 861.37(347.47-1676.19) | 1458.58(568.75-2710.04) | 69.33 | 139.5(267.09-57.23) | 168.25(314.64-66.98) | -0.732(-1.084--0.378) |
| Liberia | Female | 3123.93(1854.9-4842.66) | 5058.98(2822.66-8295.73) | 61.94 | 521.43(812.97-308.37) | 343.85(562.42-193.25) | 3.87(3.156-4.588) |
| Libya | Female | 181.08(99.5-307.52) | 441.86(249.16-763.91) | 144.01 | 19.67(34.56-10.52) | 15.95(26.27-9.17) | -2.792(-3.323--2.258) |
| Lithuania | Female | 2151.22(1507.26-2879.2) | 6133.55(4241.39-8532.82) | 185.12 | 91.44(122.01-64.07) | 290.92(407.08-198.75) | -0.736(-0.832--0.641) |
| Luxembourg | Female | 659.72(517.59-794.1) | 504.04(363.65-651.72) | -23.60 | 249.16(299.34-195.98) | 110.76(143.67-80.63) | -0.861(-1.185--0.535) |
| Madagascar | Female | 5639.36(3365.57-9334.69) | 10066.47(5778.51-16425.28) | 78.50 | 180.61(292.52-110.15) | 143.6(226.46-85.32) | 0.113(-0.102-0.328) |
| Malawi | Female | 5347.42(3238.99-8398.25) | 8185.07(4344.18-15020.61) | 53.07 | 220.08(344.13-132.05) | 170.12(306.83-91.29) | -3.394(-3.74--3.047) |
| Malaysia | Female | 1944.6(1244.48-3133.45) | 5377.76(3258.34-8401.51) | 176.55 | 37.25(59.73-23.63) | 38.54(59.22-23.4) | -1.138(-1.331--0.945) |
| Maldives | Female | 32.61(14.85-56.08) | 39(26.77-55.04) | 19.60 | 69.36(117.27-33.73) | 26.27(37.59-17.93) | -1.515(-1.765--1.263) |
| Mali | Female | 6833.14(3826.88-11708.94) | 10465.32(4996.47-19450.58) | 53.16 | 272.73(467.8-154.35) | 194.69(354.75-94.59) | -0.637(-0.723--0.551) |
| Malta | Female | 161.41(121.77-210.2) | 161.28(113.24-224.56) | -0.08 | 70.92(92.79-53.37) | 45.8(63-32.84) | -2.258(-2.447--2.069) |
| Marshall Islands | Female | 7.68(4.44-12.18) | 14.06(5.82-26.72) | 83.07 | 78.97(121.91-46.16) | 65.61(123.08-28.6) | -1.558(-2.072--1.042) |
| Mauritania | Female | 1019.46(665.13-1527.41) | 1090.11(645.04-1655.68) | 6.93 | 184.23(277.22-118.07) | 95.01(145.74-55.22) | -0.906(-1.156--0.656) |
| Mauritius | Female | 229.12(161.03-308.35) | 309.15(204.68-445.58) | 34.93 | 52.05(70.36-36.89) | 33.81(48.45-22.81) | -1.335(-1.458--1.213) |
| Mexico | Female | 67257.19(54739.03-83567.49) | 130188.15(94413.61-181096.28) | 93.57 | 258.33(318.79-212.88) | 199.93(277.16-146.17) | 0.032(-0.02-0.084) |
| Micronesia (Federated States of) | Female | 23.34(12.98-37.68) | 25.47(10.95-47.62) | 9.13 | 88.29(142.09-50.3) | 59.73(108.23-26.93) | 0.367(0.231-0.503) |
| Monaco | Female | 24.1(13.27-42.27) | 30.94(16.52-53.21) | 28.38 | 86.41(149.12-48.64) | 86.67(147.54-46.5) | -0.957(-1.159--0.754) |
| Mongolia | Female | 3037.75(2168.68-4063.41) | 8239.46(5652.86-11608.74) | 171.24 | 496.91(665.57-351.98) | 554.2(756.32-385.16) | -0.821(-0.933--0.709) |
| Montenegro | Female | 162.75(99.84-230.54) | 163.06(113.63-226.92) | 0.19 | 47.51(67.13-29.3) | 35.7(49.86-25) | -0.527(-0.724--0.329) |
| Morocco | Female | 1408.18(804.56-2248.9) | 2513.32(1417.54-4181.56) | 78.48 | 19.27(30.9-10.99) | 15.08(24.29-8.85) | 0.664(0.099-1.232) |
| Mozambique | Female | 2752.59(1612.93-4429.09) | 4664.41(1520.17-8167.64) | 69.46 | 80.93(129.69-47.99) | 68.46(118.77-22.09) | 0.494(0.229-0.759) |
| Myanmar | Female | 12250.83(6999.5-19210.41) | 29715.74(18885.67-44469.85) | 142.56 | 84.25(131.51-48.78) | 100.48(149.1-64.39) | -0.396(-0.509--0.283) |
| Namibia | Female | 646.01(283.13-1208.19) | 1592.97(712.55-2975.3) | 146.59 | 143.22(265.44-63.36) | 163.77(303.11-75.25) | -0.675(-0.931--0.418) |
| Nauru | Female | 2(0.91-3.63) | 2.39(1.03-4.66) | 19.50 | 76.83(129.79-37.42) | 68.73(127.22-31.96) | -1.546(-1.867--1.225) |
| Nepal | Female | 13594.28(8864.72-19314.39) | 25869.08(16933.9-37653.44) | 90.29 | 241.12(340.68-158.46) | 198.36(290.04-130.61) | -1.513(-1.646--1.379) |
| Netherlands | Female | 9353.98(7282.17-11327.29) | 9347.63(6996.99-11760.42) | -0.07 | 92.49(111.65-72.56) | 58.8(73.25-44.84) | 0.938(0.433-1.446) |
| New Zealand | Female | 1279.03(1051.67-1498.92) | 1510.38(1161.01-1881.09) | 18.09 | 65.63(76.84-53.99) | 42.47(52.08-33.08) | -0.643(-0.781--0.505) |
| Nicaragua | Female | 1008.07(758-1294.74) | 3620.42(2597.25-4842.18) | 259.14 | 106.41(135.91-79.94) | 139.3(185.67-100.42) | -0.067(-0.284-0.151) |
| Niger | Female | 2410.7(1463.38-3707.68) | 5901.21(3206.94-9441.27) | 144.79 | 150.37(230.99-93.05) | 124.65(195.05-67.77) | -0.842(-1.11--0.574) |
| Nigeria | Female | 49500.71(28375.15-82291.26) | 118640.81(63955.03-202879.86) | 139.67 | 212.78(348.62-121.68) | 209.29(352.7-116.82) | -0.276(-0.7-0.15) |
| Niue | Female | 0.61(0.36-0.98) | 0.47(0.27-0.79) | -22.95 | 55.73(90.29-32.04) | 43.71(72.22-24.77) | -1.828(-1.951--1.704) |
| North Macedonia | Female | 609.34(453.45-841.8) | 855.55(600.87-1164.02) | 40.41 | 59.09(81.03-44.29) | 52.92(72.26-37.67) | -1.31(-1.664--0.955) |
| Northern Mariana Islands | Female | 9.42(5.83-14.17) | 15.19(9.51-22.82) | 61.25 | 89.83(132.46-56.95) | 52.37(75.21-34.35) | -1.463(-1.785--1.139) |
| Norway | Female | 1432.41(1170.15-1728.54) | 1462.92(1124.04-1889.68) | 2.13 | 50.48(60.89-41.35) | 35.04(44.53-27.33) | -0.584(-0.69--0.477) |
| Oman | Female | 84.16(47.02-139.38) | 140.36(78.87-230.84) | 66.78 | 26.02(43.63-14.51) | 16.86(27.16-9.66) | -0.574(-0.665--0.482) |
| Pakistan | Female | 39575.69(22722.95-65945.81) | 74455.66(46251.33-114069.84) | 88.13 | 142.33(248.22-79.16) | 120.43(185.64-74.68) | 0.525(0.373-0.678) |
| Palau | Female | 2.14(1-3.87) | 3.82(1.8-6.84) | 78.50 | 38.9(69.14-18.14) | 32.83(58.05-16.01) | -0.104(-0.84-0.637) |
| Palestine | Female | 197.44(86.75-431.84) | 586.86(277.23-1192.15) | 197.23 | 36.59(79.25-16.46) | 42.45(85.96-19.27) | -0.119(-0.199--0.038) |
| Panama | Female | 672.09(499.99-872.68) | 1654.88(1106.92-2458.28) | 146.23 | 81.55(105.31-61.16) | 78.56(116.49-52.54) | -1.762(-2.037--1.487) |
| Papua New Guinea | Female | 158.41(89.18-255.46) | 408.38(223.77-680.92) | 157.80 | 13.24(21.37-7.52) | 12.72(20.83-7.11) | -1.485(-2.058--0.909) |
| Paraguay | Female | 1221.32(851.62-1657.42) | 1621.81(965.66-2630.34) | 32.79 | 89.56(123.48-62.13) | 52.5(84.84-31.24) | -0.981(-1.181--0.781) |
| Peru | Female | 13881.58(10115.13-18555.94) | 21666.7(13893.22-32955.89) | 56.08 | 194.06(257.43-142.55) | 128.74(195.95-82.4) | 0.332(0.022-0.643) |
| Philippines | Female | 24045.24(15539.29-37017.5) | 43296.83(27600.1-66623.44) | 80.06 | 122.5(192.24-78.09) | 91.57(140.17-58.9) | -4.539(-4.893--4.183) |
| Poland | Female | 23932.84(21430.36-27057.76) | 33632.34(25467.05-43801.89) | 40.53 | 100.23(114.14-89.61) | 113.67(149.41-84.78) | -2.315(-2.864--1.763) |
| Portugal | Female | 20287.05(15393.3-25150.88) | 7771.87(5555.7-10553.19) | -61.69 | 306.87(378.89-233.21) | 80.71(107.92-58.04) | -0.232(-0.784-0.323) |
| Puerto Rico | Female | 2759.32(1894.98-3994.04) | 2470.96(1600.28-3655.77) | -10.45 | 144.38(207.51-98.9) | 77.61(114.58-49.79) | -4.714(-4.928--4.499) |
| Qatar | Female | 13.29(7.91-20.71) | 59.58(33.16-100.11) | 348.31 | 31.01(49.68-18.16) | 29.28(48.43-16.18) | -2.296(-3.64--0.933) |
| Republic of Korea | Female | 71332.4(53198.72-88129.3) | 38789.56(27335.5-50476.07) | -45.62 | 364.03(454.01-270.39) | 89.76(115.02-64.38) | -0.035(-0.693-0.627) |
| Republic of Moldova | Female | 36123.9(26781.69-45041.48) | 21086.7(14837.85-28355.66) | -41.63 | 1372.95(1704.81-1029.83) | 703.56(947.85-493.71) | 4.678(3.605-5.763) |
| Romania | Female | 44861.17(33301.87-58778.48) | 49521.71(34715.26-67796.98) | 10.39 | 301.28(395.23-225.14) | 292.03(403.42-201.68) | -3.07(-3.404--2.734) |
| Russian Federation | Female | 99261.6(77971.49-125546.18) | 357697.78(257251.49-489999.58) | 260.36 | 97.46(123.58-75.82) | 335.59(460.3-240.52) | -3.339(-3.518--3.16) |
| Rwanda | Female | 15762.88(8585.5-23929.63) | 13807.76(7677.87-21426.66) | -12.40 | 811.78(1219.77-442.37) | 331.04(513.03-183.58) | -2.878(-3.111--2.645) |
| Saint Kitts and Nevis | Female | 32.04(22.17-43.88) | 23.64(13.88-40.74) | -26.22 | 172.63(240.09-119.22) | 63.78(109.75-38.01) | -0.59(-0.869--0.311) |
| Saint Lucia | Female | 142.21(102.05-185.02) | 137.55(92.94-196.37) | -3.28 | 287.39(375.95-205.68) | 123.13(175.68-82.9) | -0.508(-0.586--0.429) |
| Saint Vincent and the Grenadines | Female | 33.03(23.67-45.57) | 47.31(30.86-68.85) | 43.23 | 84.53(117.71-59.67) | 72.26(105.1-47.52) | -0.169(-0.291--0.047) |
| Samoa | Female | 27.08(16.1-41.81) | 38.43(21.06-62.62) | 41.91 | 55.29(85.63-33.15) | 47.84(78.11-26.2) | -0.601(-1.064--0.135) |
| San Marino | Female | 25.61(13.88-37.34) | 42.86(20.33-73.22) | 67.36 | 159.84(230.37-86.86) | 151.67(260.5-73.21) | -2.92(-3.064--2.775) |
| Sao Tome and Principe | Female | 127.17(68.13-206.12) | 203.6(104.78-348.64) | 60.10 | 348.41(563.53-187.72) | 300.94(520.36-150.38) | -0.949(-1.326--0.571) |
| Saudi Arabia | Female | 1349.78(585.87-2831.83) | 1678.82(973.36-2668.58) | 24.38 | 49.93(100.16-22.58) | 21.16(33.79-12.66) | -1.274(-1.898--0.646) |
| Senegal | Female | 2634.14(1611.97-4416.11) | 4259.12(2365.87-6994.57) | 61.69 | 131.26(208.97-82.57) | 94.32(152.64-51.53) | 0.996(0.788-1.204) |
| Serbia | Female | 4950.26(3486.6-7047.62) | 4019.38(2810.64-5866.76) | -18.80 | 81.12(114.1-57.65) | 55.43(82.33-37.9) | -1.511(-1.717--1.305) |
| Seychelles | Female | 20.39(13.86-28.35) | 52.39(31.22-85.44) | 156.94 | 67.68(95.05-45.63) | 89.92(147.05-53.96) | -2.192(-2.402--1.981) |
| Sierra Leone | Female | 4102.3(2387.91-6597.58) | 5589.52(2563.53-10396.05) | 36.25 | 379.44(609.83-221.69) | 242.13(447.4-111.53) | -1.186(-1.566--0.805) |
| Singapore | Female | 293.18(196.16-424.36) | 446.44(277.09-678.02) | 52.28 | 21.25(30.37-14.3) | 11.26(17.02-7.03) | -4.736(-5.379--4.088) |
| Slovakia | Female | 6987.8(5320.02-9209.66) | 6928.58(4828.16-9745.53) | -0.85 | 230.47(304.88-173.31) | 164.89(234.12-113.07) | -0.672(-1.019--0.323) |
| Slovenia | Female | 4790.68(2879.31-7261.82) | 1656.58(1148.19-2438.43) | -65.42 | 365.8(553.96-218.33) | 92.06(136.35-63.93) | -0.495(-0.599--0.391) |
| Solomon Islands | Female | 87.72(50.85-142.23) | 184.03(117.18-277.22) | 109.79 | 103.68(169-61.32) | 85.85(127.56-55.6) | -2.546(-3.316--1.769) |
| Somalia | Female | 2708.82(1317.74-4701.26) | 6290.34(3238.95-11125.28) | 132.22 | 170.51(291.29-84.49) | 147.2(259.66-77.9) | -0.416(-0.533--0.298) |
| South Africa | Female | 30237.85(19917.98-44719.78) | 27772.45(18543.19-40476.05) | -8.15 | 201.97(297.87-133.92) | 98.99(144.41-66.8) | -3.974(-4.384--3.563) |
| South Sudan | Female | 1411.5(830.08-2185.29) | 2285.78(1265.68-3912.68) | 61.94 | 125.4(199.15-74.34) | 110.47(184.17-63.58) | -0.632(-1.032--0.23) |
| Spain | Female | 56892.99(42524.19-72394.13) | 28266.53(19997.5-38553.85) | -50.32 | 213(266.25-161.08) | 65.14(86.78-47.2) | -2.162(-2.564--1.757) |
| Sri Lanka | Female | 2905.11(1981.24-4123.64) | 5432.08(3189.99-9437.31) | 86.98 | 47.51(66.69-32.6) | 38.67(66.43-23.09) | -1.39(-1.927--0.849) |
| Sudan | Female | 2087.84(894.65-4706.01) | 1901.18(977.34-3255.1) | -8.94 | 38.45(82.25-16.69) | 20.1(35.04-10.32) | -0.519(-0.783--0.256) |
| Suriname | Female | 308.55(216.49-434.21) | 437.29(293.67-641.99) | 41.72 | 205.36(287.21-144.6) | 133.87(196.02-90.44) | -1.422(-1.709--1.133) |
| Sweden | Female | 4863.8(3871.55-5883.63) | 5631.85(4363.56-6950.24) | 15.79 | 76.11(91.91-60.7) | 64.56(79.02-50.18) | -1.847(-2.201--1.492) |
| Switzerland | Female | 5977.7(4866.71-6958.64) | 6236.03(4908.15-7702.1) | 4.32 | 125.93(145.77-103.06) | 82.33(99.43-65.53) | -4.169(-4.433--3.904) |
| Syrian Arab Republic | Female | 1059.37(552.01-2053.37) | 1221.16(689.34-2018.04) | 15.27 | 32.96(60.65-17.66) | 19.39(31.64-11.48) | 0.63(0.398-0.862) |
| Taiwan (Province of China) | Female | 11004.13(6563.09-17331.32) | 8421.04(5049.42-13756.84) | -23.47 | 140.99(223.63-84.83) | 41.08(66.3-24.78) | -1.689(-1.908--1.471) |
| Tajikistan | Female | 4198.85(3112.92-5639.36) | 9415.58(6721.54-12664.43) | 124.24 | 241.85(318.16-180.52) | 286.69(380.78-211.43) | -0.436(-0.68--0.192) |
| Thailand | Female | 34445.02(22418.75-50771.6) | 48910.27(29146.61-78351.62) | 42.00 | 148.46(215.71-97.89) | 90.85(145.18-54.51) | -2.151(-2.345--1.957) |
| Timor-Leste | Female | 156.2(89.62-245.96) | 330.85(178.71-526.31) | 111.81 | 85.94(133.88-50.03) | 75.3(119.96-42.03) | -1.331(-1.397--1.266) |
| Togo | Female | 2838.33(1616.76-4505.48) | 4481.2(2089.08-7943.02) | 57.88 | 313.64(494.6-178.34) | 167.08(291.04-79.49) | -0.909(-1.026--0.791) |
| Tokelau | Female | 0.52(0.26-0.9) | 0.34(0.17-0.57) | -34.62 | 73.73(128.43-37.07) | 49.82(83.06-26.06) | -1.111(-1.965--0.249) |
| Tonga | Female | 21.88(14.15-32.24) | 22.81(14.16-34.43) | 4.25 | 69.09(101.79-45.49) | 53.21(80.52-32.86) | 0.431(0.37-0.492) |
| Trinidad and Tobago | Female | 548.59(387.31-747.68) | 720.4(435.87-1143.89) | 31.32 | 114.05(157.01-80.35) | 80.16(126.92-48.55) | -2.054(-2.217--1.89) |
| Tunisia | Female | 681.4(430.65-1035.58) | 1632.26(887.62-2986.91) | 139.55 | 22.13(33.81-13.74) | 25.1(45.91-13.59) | 1.318(0.684-1.956) |
| Turkey | Female | 8214.13(4517.53-14078.77) | 9806.5(5403.84-16048.35) | 19.39 | 37.77(65.44-20.63) | 20.91(34.07-11.56) | -1.361(-1.412--1.31) |
| Turkmenistan | Female | 3281.37(2578.2-4041.59) | 9742.06(6920.63-13287.33) | 196.89 | 272.13(332.97-212.63) | 395.42(537.02-282.54) | -0.038(-0.255-0.179) |
| Tuvalu | Female | 3.27(1.59-5.53) | 2.87(1.43-5.05) | -12.23 | 77.03(130.09-38.3) | 51.33(89.79-25.81) | 4.894(2.479-7.366) |
| Uganda | Female | 12883.65(6933.38-20109.54) | 31458.28(16520.32-47543.06) | 144.17 | 325.15(503.26-174.03) | 319.88(483.75-167.22) | -4.445(-5.037--3.849) |
| Ukraine | Female | 46746.74(36154.84-59799.32) | 144237.01(97188.22-198601.45) | 208.55 | 127.95(162.06-99) | 469.96(652.78-314.84) | 1.488(1.191-1.787) |
| United Arab Emirates | Female | 119.18(61-212.66) | 223.48(99.91-426.66) | 87.51 | 55.45(98.4-28.28) | 15.05(27.97-7.41) | -1.18(-1.354--1.005) |
| United Kingdom | Female | 32812.96(27292.3-38298.24) | 62608.05(50575.9-75763.44) | 90.80 | 88.05(103.29-72.88) | 132.55(160.41-107.18) | 0.622(0.404-0.841) |
| United Republic of Tanzania | Female | 26037.52(14749.35-40355.48) | 42420.31(20241-72490.56) | 62.92 | 402.18(622.98-228.82) | 281.65(480.73-134.23) | -1.552(-1.67--1.434) |
| United States of America | Female | 215103.24(165344.6-272145.19) | 406113.73(308814.45-510960.13) | 88.80 | 141.4(178.58-108.74) | 170.37(213.29-129.52) | -2.312(-2.482--2.141) |
| United States Virgin Islands | Female | 79.72(42.66-135.21) | 92.9(51.65-159.66) | 16.53 | 158.62(268.96-85.03) | 99.64(171.19-54.62) | 2.094(1.722-2.466) |
| Uruguay | Female | 2182.95(1573.22-2856.7) | 1591.25(1069.65-2226.33) | -27.11 | 110.88(145.42-80.35) | 59.87(82.89-40.37) | -0.689(-1.078--0.299) |
| Uzbekistan | Female | 16712.17(12922.68-20605.38) | 63892.71(48394.93-82632.48) | 282.31 | 243.52(299.73-188.05) | 435.96(553.08-331.18) | -2.232(-2.522--1.94) |
| Vanuatu | Female | 24.31(13.5-39.12) | 54.64(28.77-89.69) | 124.76 | 68.82(111.7-38.27) | 57.34(94.5-30.67) | -1.35(-1.448--1.252) |
| Venezuela (Bolivarian Republic of) | Female | 7448.61(5337.22-10264.81) | 10255.55(6431.71-15648.46) | 37.68 | 122.52(168.84-88.19) | 65.12(98.99-40.89) | -3.8(-3.892--3.708) |
| Viet Nam | Female | 16653.9(9985.04-25091.81) | 25696.05(16338.11-40455.92) | 54.29 | 71.16(108.32-42.72) | 48.19(75.45-30.69) | -0.86(-1.065--0.656) |
| Yemen | Female | 2210.57(786.04-4500.07) | 1913.96(1050.74-3494.84) | -13.42 | 73.09(154.78-26.21) | 23.81(41.33-13.05) | -0.672(-1.189--0.153) |
| Zambia | Female | 9048.64(5093.94-15087.87) | 17541.23(8898.86-29067.4) | 93.85 | 487.78(809.15-274.11) | 378.19(620.48-193.14) | -0.631(-0.765--0.498) |
| Zimbabwe | Female | 2982.84(1621.49-5146.6) | 4685.64(1556.1-9394.03) | 57.09 | 119.64(202.89-65.83) | 99.11(192.39-34.11) | -0.056(-0.476-0.366) |
| Afghanistan | Male | 2928.47(1394.86-5105.22) | 4629.81(2234.87-8216.69) | 58.10 | 74.94(127.18-36.2) | 61.5(100.92-32.19) | 0.38(0.297-0.464) |
| Albania | Male | 2407.35(1852.79-3089.83) | 3832.4(2482.32-5549.67) | 59.20 | 209.15(265.15-161.67) | 207.93(298.08-137.02) | -1.65(-1.862--1.436) |
| Algeria | Male | 5648.45(3012.27-9467.47) | 17461.25(9327.78-30063.45) | 209.13 | 78.69(133.88-42.24) | 87.6(151.88-46.56) | -1.032(-1.082--0.983) |
| American Samoa | Male | 37.86(19.57-70.13) | 35.95(20.64-63.1) | -5.04 | 221.74(396.63-123.34) | 137.02(239.19-80.16) | 1.195(0.98-1.411) |
| Andorra | Male | 112.14(68.42-171.91) | 182.57(125.04-246.47) | 62.81 | 356.83(541.1-220.33) | 264.06(357.03-179.39) | -0.557(-1.158-0.048) |
| Angola | Male | 26615.98(15702.02-41345.72) | 99624.62(68162.7-136634.82) | 274.30 | 977.71(1501.44-587.6) | 1379.21(1882.25-958.04) | -0.669(-0.797--0.54) |
| Antigua and Barbuda | Male | 83.21(62.48-108.95) | 161.14(116.94-206.99) | 93.65 | 368.81(483.81-275.56) | 310.9(400.48-227.08) | 2.389(1.965-2.815) |
| Argentina | Male | 105375.4(89642.94-119067.05) | 133098.81(108600.3-157401.06) | 26.31 | 694.92(785.81-591.81) | 560.56(662.91-457.22) | -1.148(-1.502--0.793) |
| Armenia | Male | 3888.24(2980.92-4983.78) | 9808.72(7229.43-12899.48) | 152.27 | 282.94(356.47-221.85) | 566.84(744.78-418.4) | -2.798(-3.307--2.287) |
| Australia | Male | 23973.68(20578.72-26531.87) | 31380.51(26658.91-35697.11) | 30.90 | 263.8(292.24-226.44) | 187.7(213.12-159.11) | 0.101(-0.055-0.258) |
| Austria | Male | 39473.7(33260.05-43726.48) | 26799.68(21972.01-30468.5) | -32.11 | 875.57(970.77-737.41) | 388.43(441.4-319.08) | -2.055(-2.525--1.582) |
| Azerbaijan | Male | 19907.15(15092.97-24544.59) | 40525.19(27678.73-56934.55) | 103.57 | 793.22(971.88-605.49) | 812.55(1124.43-549.54) | -4.373(-4.838--3.906) |
| Bahamas | Male | 698.15(553.59-835.2) | 918.54(595.09-1285.49) | 31.57 | 803.64(961.25-639.38) | 439.71(616.31-287.36) | -2.345(-2.547--2.143) |
| Bahrain | Male | 623.56(421.65-838.92) | 1244.77(680.09-2034.23) | 99.62 | 428.28(607.19-273.51) | 120.94(205.51-65.95) | -1.101(-1.473--0.727) |
| Bangladesh | Male | 124922.88(82126.93-175540.35) | 142293.24(92368.42-208625.32) | 13.90 | 407.54(573.59-270.52) | 200.28(292.87-131.28) | 3.392(2.097-4.703) |
| Barbados | Male | 514.07(401.06-618.38) | 690.86(496.13-900.87) | 34.39 | 464.66(564.67-363.89) | 335.14(436.34-244.6) | -0.819(-0.922--0.715) |
| Belarus | Male | 12270.7(9694.81-14770.84) | 37181.27(26234.2-50957.27) | 203.01 | 230.83(276.84-183.3) | 612.89(834.7-434.5) | 1.343(0.975-1.712) |
| Belgium | Male | 23486.84(19914.95-26339.97) | 26326.15(21228.73-30863.3) | 12.09 | 382.25(429.27-324.05) | 302.05(352.93-245.14) | -0.737(-0.908--0.566) |
| Belize | Male | 234.53(174.75-292.98) | 1113.81(842.23-1414.93) | 374.91 | 443.71(557.24-331.13) | 654.19(834.52-491.78) | -2.869(-3.033--2.705) |
| Benin | Male | 9916.88(5980.29-14674.49) | 22603.58(13723.03-33875.65) | 127.93 | 894.86(1311.44-542.21) | 722.02(1084.19-437.85) | -2.739(-2.966--2.512) |
| Bermuda | Male | 152.84(122.8-180.17) | 113.86(80.79-146.32) | -25.50 | 505.68(596.37-406) | 217.42(279.05-154.37) | -0.592(-0.74--0.444) |
| Bhutan | Male | 1707.32(944.21-3067.53) | 1379.49(685.24-3015.74) | -19.20 | 877.78(1593.04-486.41) | 395.19(878.25-202.8) | -1.697(-2.114--1.278) |
| Bolivia (Plurinational State of) | Male | 16311(9904.42-24524.9) | 35160.95(22299.4-50874.15) | 115.57 | 889.71(1338.11-546.64) | 744.69(1083.1-476.28) | -1.025(-1.225--0.824) |
| Bosnia and Herzegovina | Male | 12553.51(9789.29-15198.59) | 8943.59(6348.56-12009.38) | -28.76 | 562.45(681.58-439.66) | 349.45(471.86-247.12) | -1.401(-1.59--1.211) |
| Botswana | Male | 3948.45(2214.38-6663.37) | 7586.33(4664.2-11182.47) | 92.13 | 1189.98(1981.08-682.53) | 866.78(1228.68-545.9) | -1.818(-1.974--1.661) |
| Brazil | Male | 455483.9(369123.39-547610.46) | 637008.22(524417.56-772606.45) | 39.85 | 816.84(981.1-662.85) | 541.49(658.62-446.29) | 1.147(0.751-1.545) |
| Brunei Darussalam | Male | 137.59(83.73-204.16) | 208.29(139.04-300.42) | 51.38 | 161.9(245.28-101.94) | 94.02(132.31-63.34) | -1.275(-1.557--0.992) |
| Bulgaria | Male | 43076.72(36343.39-48887.94) | 52799.72(38653.15-68731.07) | 22.57 | 763.88(866.73-642.06) | 1009.43(1314.01-738.48) | -2.053(-2.232--1.873) |
| Burkina Faso | Male | 24228.2(16831.6-32540.27) | 37604(16177.06-69656.18) | 55.21 | 1031.1(1383.82-720.32) | 710.04(1300.97-318.85) | -0.003(-0.359-0.353) |
| Burundi | Male | 23978.08(13274.53-39030.65) | 32841.37(17200.51-66236.22) | 36.96 | 1849.46(2949.25-1058.2) | 1017.47(2031.96-526.26) | 3.41(3.179-3.64) |
| Cabo Verde | Male | 717.43(467.45-1097.41) | 1810.99(1251.26-2469.28) | 152.43 | 807.78(1232.86-532.54) | 779.22(1043.05-547.71) | -1.144(-1.237--1.05) |
| Cambodia | Male | 19253.85(11157.89-32691.62) | 129305.53(91080.78-172821.07) | 571.58 | 731.76(1227.95-433.92) | 1941.09(2579.26-1354.58) | -0.723(-0.814--0.633) |
| Cameroon | Male | 32245.41(22017.56-45216.11) | 69541.06(41051.71-107845.58) | 115.66 | 1184.97(1652.66-811.2) | 850.08(1311.98-501.5) | -1.926(-2.133--1.718) |
| Canada | Male | 41990.89(33602.23-49354.77) | 63766.71(50427.28-76646.7) | 51.86 | 284.13(334.61-226.78) | 226.41(270.36-180.07) | 1.465(1.123-1.808) |
| Central African Republic | Male | 10092.12(4982.64-17371.84) | 11681.58(4591.8-22881.56) | 15.75 | 1414.6(2403.91-745.43) | 810.28(1517.56-349.69) | -2.287(-2.888--1.683) |
| Chad | Male | 8724.12(4019.83-16463.16) | 33973.19(16423.49-54045.88) | 289.42 | 579.85(1080.19-273.78) | 872.01(1380.4-423.59) | -2.562(-2.711--2.412) |
| Chile | Male | 72986.76(61142.33-83781.37) | 80065.35(65370.91-93575.42) | 9.70 | 1403.22(1612.49-1173.99) | 725.52(848.02-592.78) | -2.509(-2.727--2.29) |
| China | Male | 2740759.16(1964588.4-3567170.85) | 2505244.78(1777987.35-3280270.26) | -8.59 | 528.42(685.21-381.32) | 250.56(328.23-178.92) | -0.209(-0.795-0.381) |
| Colombia | Male | 28643.42(23115.21-33895.29) | 38509.15(25183.12-55059.53) | 34.44 | 274.22(326.06-220.68) | 157.52(224.97-103.33) | -1.782(-2.147--1.416) |
| Comoros | Male | 346.88(130.2-790.39) | 700.6(355.25-1264.33) | 101.97 | 297.93(667.79-115.31) | 276.76(492.83-143.04) | 1.545(1.313-1.778) |
| Congo | Male | 8717.74(5053.05-13212.86) | 14791.1(8219.65-22562.1) | 69.67 | 1422.31(2087.92-842.64) | 841.7(1246.58-466.56) | -0.236(-0.773-0.304) |
| Cook Islands | Male | 17.08(7.97-33.46) | 37.4(24.81-50.58) | 118.97 | 218.79(422.2-104.11) | 335.9(455.64-226.39) | -2.585(-3.158--2.009) |
| Costa Rica | Male | 5056.62(4096.38-5904.16) | 11782.83(7915.8-16409.37) | 133.02 | 504.6(592.39-409.98) | 474.87(663.43-319.97) | 1.567(0.676-2.466) |
| Croatia | Male | 34242.75(28658.05-39455.87) | 18597.86(13282.55-24711.19) | -45.69 | 1167.4(1345.45-977.7) | 556.37(736.4-399.66) | -1.645(-1.856--1.432) |
| Cuba | Male | 11830.38(9237.45-14593.47) | 29590.95(20591.19-39735.73) | 150.13 | 228.5(283.27-178.19) | 348.36(467.15-243.09) | -1.57(-2.019--1.119) |
| Cyprus | Male | 1220.64(850.18-1620.79) | 1721.82(1325.1-2096.99) | 41.06 | 317.55(428.41-220.77) | 196.66(238.72-152.55) | -0.886(-1.048--0.724) |
| Czechia | Male | 45290.28(38760.59-50615.29) | 39089.48(29413.36-50639.59) | -13.69 | 766.54(856.88-657.1) | 485.34(626.99-366.85) | -1.198(-1.259--1.137) |
| C么te d'Ivoire | Male | 35460.52(21515.97-52788.82) | 70655.01(43963.72-103258.47) | 99.25 | 1090.75(1617.16-674.82) | 845.69(1236.29-536.29) | -1.602(-1.935--1.268) |
| Democratic People's Republic of Korea | Male | 58565.49(33809.17-87817.92) | 75801.15(37734.59-113938.79) | 29.43 | 676.69(985.13-407.45) | 477.45(713.96-239.76) | -1.419(-1.683--1.154) |
| Democratic Republic of the Congo | Male | 92584.77(53589.94-138246.34) | 145528.05(71117.88-255516.29) | 57.18 | 975.36(1465-562.5) | 611.98(1072.95-311.05) | -1.073(-1.27--0.876) |
| Denmark | Male | 14430.77(12057.66-16335.45) | 13332.55(10521.64-15830.91) | -7.61 | 460.41(520.91-385.7) | 302.53(359.74-236.52) | -1.208(-1.298--1.117) |
| Djibouti | Male | 460.32(245.71-940.96) | 1257.68(704.42-2277.52) | 173.22 | 423.48(856.32-235.11) | 304.61(561.34-173.69) | -0.33(-0.712-0.053) |
| Dominica | Male | 127.8(94.46-163.32) | 144.01(101.3-196.1) | 12.68 | 469.8(602.44-342.59) | 330.75(447.62-233.52) | -0.316(-0.614--0.018) |
| Dominican Republic | Male | 18204.07(13816.9-22994.84) | 35996.76(20481.59-55469.74) | 97.74 | 809.36(1026.75-611.48) | 726.1(1107.28-419.99) | -0.58(-0.947--0.211) |
| Ecuador | Male | 17799.33(13447.27-23093.04) | 40555.61(27622.25-58199.85) | 127.85 | 550.72(713.79-417.35) | 519.59(744.93-355.29) | -0.405(-1.47-0.671) |
| Egypt | Male | 54339.92(26823.7-97782.13) | 107349.44(45747.4-215722.57) | 97.55 | 329.2(582.51-170.81) | 278.4(545.28-124.09) | -0.908(-1.297--0.518) |
| El Salvador | Male | 14220(10859.34-17770.8) | 19400.75(12644.61-27610.54) | 36.43 | 846(1058.94-644.72) | 760.96(1085.94-496.48) | -0.019(-0.214-0.178) |
| Equatorial Guinea | Male | 920.16(413.7-1765.59) | 2058.7(1086.08-3584.89) | 123.73 | 849.25(1595.74-389.54) | 644.5(1081.33-366.71) | 2.77(1.156-4.409) |
| Eritrea | Male | 3951.25(1821.31-7406.25) | 11265.33(5894.6-20584.47) | 185.11 | 638.42(1145.97-322) | 638.53(1150.06-359.45) | -0.302(-0.577--0.027) |
| Estonia | Male | 2316.68(1845.66-2787.09) | 5140.14(3592.88-6846.72) | 121.88 | 278.18(334.69-221.92) | 602.01(803.24-421.08) | -0.302(-0.45--0.155) |
| Eswatini | Male | 1958.91(1324.35-2822.75) | 3561.29(2190.9-5548.28) | 81.80 | 1124.39(1607.15-760.29) | 1052.25(1586.91-667.05) | -0.54(-0.949--0.13) |
| Ethiopia | Male | 124427.44(52977.29-236077.67) | 237560.55(142401.71-347350.02) | 90.92 | 957.18(1815.71-412.5) | 870.75(1272.6-531.58) | 0.665(0.171-1.161) |
| Fiji | Male | 857.03(567.77-1204.42) | 1171.21(705.81-1715.17) | 36.66 | 308.41(433.07-200.28) | 260.42(382.72-157.15) | -2.549(-2.803--2.294) |
| Finland | Male | 11893.67(9967.79-13677.4) | 19200.77(15565.84-22514.88) | 61.44 | 393.75(452.07-330.43) | 476.48(556.73-389.95) | -1.689(-2.039--1.338) |
| France | Male | 219497.42(186881.36-241180.57) | 150629.36(123496.12-173544.35) | -31.38 | 659.94(725.81-564.33) | 311.29(357.97-255.81) | 0.727(0.242-1.214) |
| Gabon | Male | 5678.53(3626.33-8020.25) | 7170.32(4524.11-10357.56) | 26.27 | 1904.85(2725.67-1207.88) | 1149.49(1626.72-735.24) | 1.391(0.698-2.088) |
| Gambia | Male | 1543.57(842.84-2629.37) | 4497.82(2732.37-6845.25) | 191.39 | 623.28(1049.22-342.82) | 741.88(1116.78-443.7) | -2.007(-2.237--1.775) |
| Georgia | Male | 24366.95(17858.82-31132.11) | 29042.73(21033.67-36838.95) | 19.19 | 885.25(1131.86-653.48) | 1313.03(1664.63-963.7) | -1.02(-1.156--0.884) |
| Germany | Male | 368318.63(315745.07-404868.56) | 283240.73(234108.41-325385.11) | -23.10 | 734.29(807.47-631.71) | 410.03(469.68-338.32) | -1.615(-1.848--1.381) |
| Ghana | Male | 51263.8(32087.86-81010.77) | 92853.34(59715.23-133134.42) | 81.13 | 1324.02(2096.09-832.22) | 983.9(1396.04-643.69) | -0.08(-0.298-0.139) |
| Greece | Male | 21841.79(18607.63-24298.73) | 16904.6(13820.73-19479.35) | -22.60 | 325.25(361.74-276.5) | 202.9(233.47-167.22) | -1.27(-1.552--0.986) |
| Greenland | Male | 72.64(41.97-107.73) | 108.98(68.18-155.14) | 50.03 | 264.5(388.8-158.25) | 255.73(365.87-160.24) | 0.624(0.293-0.957) |
| Grenada | Male | 219.69(169.63-263.97) | 321.3(245.35-394.07) | 46.25 | 773.35(932.65-597.39) | 532.45(650.79-409.92) | -0.437(-1.061-0.192) |
| Guam | Male | 170.21(76.88-353.25) | 336.42(121.81-614.57) | 97.65 | 296.84(594.47-145.49) | 355.67(646.42-128.55) | 0.678(0.532-0.825) |
| Guatemala | Male | 36005.88(26523.13-47662.84) | 80468.75(54640.73-110999.89) | 123.49 | 1435.93(1903.89-1059.53) | 1250.46(1738.1-846.43) | -0.658(-0.723--0.592) |
| Guinea | Male | 8674.92(5213.93-14321.06) | 19364.47(10701.9-31290.19) | 123.22 | 475.46(776.16-284.86) | 578.86(928.49-326.66) | -1.055(-1.79--0.314) |
| Guinea-Bissau | Male | 3850.15(2033.64-6002.36) | 6350.81(3643.34-9678.2) | 64.95 | 1593.27(2458.48-853.64) | 1314.85(1962.89-756.41) | -1.037(-1.171--0.903) |
| Guyana | Male | 4702.15(3633.77-5705.32) | 5033.52(3607.53-6680.84) | 7.05 | 1951.23(2377.83-1508.48) | 1411.89(1864.9-1015.91) | -0.395(-0.486--0.305) |
| Haiti | Male | 20841.91(10679.95-30614.68) | 32205.25(17159.51-48988.81) | 54.52 | 1079.75(1582.5-565.5) | 785.58(1187.66-423.56) | -3.028(-3.62--2.433) |
| Honduras | Male | 11907.1(7653.87-16848.58) | 26771.27(15255.05-41829.29) | 124.83 | 931.48(1314.9-602.59) | 831.04(1299.58-470.59) | -0.35(-0.525--0.174) |
| Hungary | Male | 122627.65(105397.94-136382.9) | 61400.3(46800.07-77385.49) | -49.93 | 2014.52(2241.16-1728.8) | 856.64(1081.81-655.09) | 0.432(-0.028-0.894) |
| Iceland | Male | 149.36(113.97-186.71) | 251.76(194.78-304.87) | 68.56 | 117.98(147.29-89.88) | 106.14(128.47-82.83) | -0.868(-0.96--0.776) |
| India | Male | 1680737.63(1123754.36-2477536.57) | 3885927.73(2496812.88-5587252.92) | 131.20 | 501.33(739.04-341.89) | 571.41(823.89-363.21) | 2.106(1.643-2.571) |
| Indonesia | Male | 176123.33(134467.81-230571.48) | 280295.66(201512.13-377695.38) | 59.15 | 293.01(376.41-223.53) | 227.71(300.04-167.16) | -3.902(-4.021--3.783) |
| Iran (Islamic Republic of) | Male | 6587.7(4294.69-11125.44) | 31933.61(16991.26-55128.88) | 384.75 | 39.83(63.84-27.02) | 74.05(128.93-39.45) | 0.549(0.186-0.914) |
| Iraq | Male | 10103.27(5512.3-16476.32) | 10030.8(4707.1-19193.99) | -0.72 | 211.85(345.16-114.51) | 67.28(127.71-31.77) | -0.674(-0.872--0.476) |
| Ireland | Male | 2517.54(2053.23-2929.01) | 5415.15(4268.68-6430.88) | 115.10 | 140.14(163.06-114.29) | 167.28(199.24-132.6) | -3.598(-3.835--3.361) |
| Israel | Male | 2593.36(2045.81-3143.46) | 5056.44(3898.02-6543.34) | 94.98 | 120.25(145.5-94.36) | 101.86(131.65-78.31) | -0.875(-2.295-0.565) |
| Italy | Male | 246349.17(212612.53-268016.87) | 121691.76(99971.77-137508.5) | -50.60 | 674.78(733.71-581.6) | 234.17(263.97-192.77) | -2.641(-2.957--2.323) |
| Jamaica | Male | 1555.45(1165.8-1980.31) | 2008.03(1348.09-2830.77) | 29.10 | 192.23(244.7-143.25) | 136.3(191.25-91.57) | -0.964(-1.393--0.532) |
| Japan | Male | 342345.46(284436.54-398834.18) | 220160.23(176606.31-264195.63) | -35.69 | 429.97(501.53-356.46) | 198.07(235.1-160) | 3.138(2.275-4.009) |
| Jordan | Male | 682.81(313.63-1310.19) | 2537.71(1173.94-4499.71) | 271.66 | 72.91(139.06-35.83) | 54.31(96.99-26.07) | -0.676(-0.775--0.576) |
| Kazakhstan | Male | 26942(20502.17-33270.33) | 96447.48(69821.52-131284.16) | 257.98 | 447.07(554.71-339.58) | 1085.5(1455.51-795.68) | -1.813(-1.946--1.679) |
| Kenya | Male | 71402.28(41055.14-129443.45) | 162481.61(100474.82-246479.48) | 127.56 | 1417.35(2641.94-815.3) | 1164.72(1776.98-719.15) | 0.165(-1.254-1.604) |
| Kiribati | Male | 227.2(65.33-533.92) | 248.14(66.51-638.15) | 9.22 | 909.45(2053.8-300.91) | 532.04(1343.38-161.96) | 1.694(0.932-2.461) |
| Kuwait | Male | 108.75(67.52-162.99) | 408.4(242.03-636.07) | 275.54 | 19.91(30.21-12.43) | 20.52(32.51-12.2) | -0.794(-0.947--0.641) |
| Kyrgyzstan | Male | 13162.98(9846.15-16196.5) | 39073.37(29119.12-49191.55) | 196.84 | 865.08(1068.65-659.97) | 1405.13(1766.82-1056.77) | 2.866(1.39-4.363) |
| Lao People's Democratic Republic | Male | 12071.52(6726.89-17746.79) | 21680.72(13353.72-31932.51) | 79.60 | 948.02(1409.06-529.78) | 754.25(1112.41-464.95) | -2.151(-2.243--2.059) |
| Latvia | Male | 3365.52(2730.25-4028.08) | 6401.62(4397.93-8596.03) | 90.21 | 239.08(286.97-194.77) | 525.4(706.44-362.91) | 0.24(0.096-0.384) |
| Lebanon | Male | 2976.56(1585.2-4485.98) | 3068.43(1098.16-5626.69) | 3.09 | 240.74(365.77-125.64) | 128.52(234.95-45.83) | -1.354(-1.63--1.077) |
| Lesotho | Male | 4384.06(2450.88-7480.26) | 6285.18(3288.04-9855.78) | 43.36 | 816.94(1394.91-457.53) | 875.01(1343.4-484.47) | 1.569(0.92-2.221) |
| Liberia | Male | 8689.4(5928.82-11891.85) | 13920.21(7849.25-21646.34) | 60.20 | 1343.24(1838.83-920.84) | 910.93(1399.52-518.43) | 3.663(2.73-4.605) |
| Libya | Male | 508.65(259.75-888.58) | 2404.15(796.61-4942.61) | 372.65 | 45.37(80.22-22.54) | 71.94(146.66-25.55) | -2.917(-3.198--2.635) |
| Lithuania | Male | 5650.22(4512.5-6647.64) | 14939.31(10938.21-19316.52) | 164.40 | 301.91(353.96-242.18) | 856.71(1103.4-626.47) | -1.685(-1.823--1.547) |
| Luxembourg | Male | 1654.96(1411.44-1843.9) | 1352.48(1056.76-1629.97) | -18.28 | 710.61(791.75-605.74) | 301.75(363.18-237.44) | -0.148(-0.299-0.004) |
| Madagascar | Male | 25284.96(15415.71-38632.29) | 35542.23(18710.26-60231.11) | 40.57 | 780.15(1207.99-468.6) | 476.09(798.43-260.48) | -0.981(-1.191--0.77) |
| Malawi | Male | 25699.13(15072.62-39079.71) | 47585.09(28212.49-73413.08) | 85.16 | 1065.72(1621.69-620.1) | 1015.92(1568.99-597.57) | -1.31(-1.656--0.963) |
| Malaysia | Male | 19487.68(11924.22-28582.61) | 39573.19(19647.69-67265.55) | 103.07 | 337.14(499.09-204.13) | 253.15(432.22-127.25) | -1.254(-1.45--1.058) |
| Maldives | Male | 74.51(40.02-114.11) | 215.88(120.45-395.38) | 189.73 | 117.3(178.05-64.54) | 81.02(140.77-48.06) | -1.61(-1.806--1.414) |
| Mali | Male | 13338.19(7973.95-21319.45) | 20359.72(11048.43-36632.12) | 52.64 | 559.18(886.85-341.06) | 380.05(674.4-208.99) | -1.014(-1.096--0.932) |
| Malta | Male | 502.15(395.3-597.9) | 542.15(415.56-663.16) | 7.97 | 254.31(302.87-200.25) | 158.49(192.96-120.85) | -2.339(-2.493--2.185) |
| Marshall Islands | Male | 88.91(37.5-159.9) | 138.29(56.52-270.48) | 55.54 | 711.65(1266.05-322.55) | 530.48(1044.13-220.45) | -2.938(-3.383--2.491) |
| Mauritania | Male | 2456.11(1524.77-3720.33) | 2703.46(1405.65-4808.61) | 10.07 | 462.99(700.22-286.26) | 232.08(410.67-123.65) | -1.108(-1.336--0.88) |
| Mauritius | Male | 5381.83(4246.93-6577.14) | 4363.14(2922.93-5881.07) | -18.93 | 1224.21(1498.62-956.22) | 518.51(696.83-349.31) | -2.012(-2.075--1.949) |
| Mexico | Male | 458246.08(372875.31-526097.45) | 763655.49(563184.14-989705.03) | 66.65 | 1736.82(1990.14-1410.05) | 1264.5(1640-925.43) | -0.64(-0.707--0.573) |
| Micronesia (Federated States of) | Male | 352.94(171.7-604.86) | 281.4(104.59-542.51) | -20.27 | 1071.37(1820.79-530.83) | 595.98(1141.4-225.08) | 0.92(0.773-1.067) |
| Monaco | Male | 67.97(39.66-101.49) | 75.99(46.71-110.39) | 11.80 | 295.08(441.19-171.97) | 244.63(360.39-148.59) | -0.41(-0.677--0.142) |
| Mongolia | Male | 7685.96(5347.38-10467.12) | 24597.19(17155-33712.85) | 220.03 | 1287.28(1750.12-893.17) | 1677.98(2295.34-1175.44) | -2.638(-2.813--2.463) |
| Montenegro | Male | 755.86(600.42-930.35) | 922.73(671.92-1235.44) | 22.08 | 242.23(299.2-193.28) | 214.49(287.06-155.92) | 0.924(0.747-1.102) |
| Morocco | Male | 13633.79(7992.49-21080.04) | 13611.87(7550.28-23022.64) | -0.16 | 165.33(255.28-95.09) | 76.38(127.37-43.44) | 3.296(3.089-3.504) |
| Mozambique | Male | 6404(3988.17-9845.92) | 17441.28(7663.81-38730.23) | 172.35 | 195.04(290.56-121.91) | 252.84(544.01-113.69) | -0.017(-0.33-0.296) |
| Myanmar | Male | 84594.33(52108.95-130036.11) | 397079.41(259045.86-558394.61) | 369.39 | 600.07(930.75-361.86) | 1518.53(2133.01-988.31) | -0.54(-0.623--0.458) |
| Namibia | Male | 2980.98(1652.7-4732.68) | 5844.27(3618.22-8376.8) | 96.05 | 775.25(1231.65-424.04) | 754.79(1069.05-479.13) | 0.276(0.116-0.436) |
| Nauru | Male | 36.95(14.13-64.27) | 33.1(13.61-58.19) | -10.42 | 1017.71(1742.54-403.27) | 868.48(1507.94-370.42) | -1.497(-1.75--1.244) |
| Nepal | Male | 38955.7(26457.52-58681.22) | 80110.61(50597.02-125052.28) | 105.65 | 639.99(977.29-434.89) | 681.74(1064-433.27) | -1.159(-1.277--1.04) |
| Netherlands | Male | 17125.55(14422.97-19356.99) | 18113.54(14517.68-21437.2) | 5.77 | 201.77(227.39-169.68) | 130.62(153.93-104.4) | 0.69(-0.366-1.757) |
| New Zealand | Male | 2612.86(2248.9-2971.55) | 3466.9(2840.42-4071.3) | 32.69 | 148(168.07-127.2) | 108.31(126.03-88.67) | -0.711(-0.915--0.506) |
| Nicaragua | Male | 7447.46(5554.08-9413.08) | 23725.69(16349.7-32196.07) | 218.57 | 804.64(1028.22-601.68) | 989.58(1357.89-681.16) | -0.43(-0.651--0.209) |
| Niger | Male | 7509.91(4539.87-12328.01) | 15842.24(8243.56-27055.73) | 110.95 | 416.37(680.66-246.35) | 338.53(575.6-179.28) | -1.666(-1.813--1.518) |
| Nigeria | Male | 343280.08(195632.05-549398.71) | 575483.65(338761.51-957161.97) | 67.64 | 1197.51(1896.09-686.54) | 1066.46(1769.47-639.26) | -0.57(-0.754--0.386) |
| Niue | Male | 7.53(3.55-11.79) | 4.76(2.04-7.32) | -36.79 | 772.36(1210.53-365.26) | 477.39(744.79-203.6) | -1.14(-1.362--0.917) |
| North Macedonia | Male | 3834.44(3114.4-4522.67) | 5283.03(3755.66-7108.57) | 37.78 | 384(453.7-312.59) | 336.03(449.05-241.03) | -1.736(-2.216--1.254) |
| Northern Mariana Islands | Male | 114.08(40.21-244.47) | 114.42(43.99-230.68) | 0.30 | 520.11(1073.11-219.7) | 368.39(744.8-141.3) | -0.633(-1.111--0.154) |
| Norway | Male | 4179.67(3417.04-4955.11) | 3957.31(3139.19-4746.31) | -5.32 | 164.96(194.86-134.38) | 99.67(119.52-79.26) | 0.701(0.588-0.813) |
| Oman | Male | 302.4(161.77-530.48) | 811.23(363.92-1626.21) | 168.26 | 61.47(104.78-31.9) | 50.49(89.1-26.48) | -0.621(-0.73--0.511) |
| Pakistan | Male | 71399.4(32728.95-138493.37) | 195920.43(95323.37-359128.51) | 174.40 | 196.06(383.84-88.84) | 239.91(445.96-115.95) | -0.796(-1.068--0.524) |
| Palau | Male | 29.18(10.28-63.67) | 50.78(19.8-102.41) | 74.02 | 452.12(975.28-166.97) | 377.65(755.08-149.82) | -0.201(-0.61-0.21) |
| Palestine | Male | 883.38(386.81-1732.18) | 2286.87(1274.76-3602.05) | 158.88 | 198.71(388.7-87.99) | 159.72(257.14-85.97) | -1.36(-1.477--1.243) |
| Panama | Male | 2632.34(2062.5-3149.26) | 6022.85(4104.99-8306.62) | 128.80 | 311.56(373.09-240.52) | 290.56(400.6-197.43) | 0.587(-0.071-1.249) |
| Papua New Guinea | Male | 4068.03(2410.27-6075.62) | 7314.23(3574.01-12582.69) | 79.80 | 275.11(412.6-162.53) | 185.15(313.31-96.45) | -1.359(-2.031--0.682) |
| Paraguay | Male | 5194.96(4122.24-6336.13) | 14369.07(9520.28-20213.62) | 176.60 | 414.54(506.09-326.51) | 474.33(668.47-312.6) | -0.926(-1.041--0.81) |
| Peru | Male | 52775.77(37768.56-69625.01) | 82390.82(53070.28-122854.4) | 56.11 | 750.18(988.55-540.54) | 507.75(755.8-326.79) | 0.31(-0.431-1.056) |
| Philippines | Male | 150361.56(113394.86-192801.52) | 268279.68(189030.07-360522.83) | 78.42 | 740.15(977.54-554.94) | 564.06(758.29-396.06) | -3.396(-3.719--3.073) |
| Poland | Male | 101184.82(85469.09-114210.51) | 161203.58(121940.53-211721.74) | 59.32 | 517.65(585.11-437.17) | 604.74(793.46-456.19) | -2.868(-3.301--2.433) |
| Portugal | Male | 64034.21(54912.66-70310.35) | 33253.54(27403.98-37910.4) | -48.07 | 1106.66(1216.19-944.34) | 408.82(467.07-337.55) | -3.122(-3.671--2.571) |
| Puerto Rico | Male | 18698.78(14509.84-22367.04) | 12221.49(8055.82-16942.83) | -34.64 | 1122.51(1342.55-870.66) | 490.53(678.9-327.1) | -5.26(-5.528--4.992) |
| Qatar | Male | 280.34(161.06-448.38) | 1069.9(556.59-1788.75) | 281.64 | 187.04(311.95-104.5) | 77.11(125.88-42.97) | -0.381(-1.86-1.121) |
| Republic of Korea | Male | 369157.95(297640.48-424306.64) | 178118.93(142131.67-213513.59) | -51.75 | 2007.91(2314.85-1614.28) | 416.77(501.79-333.98) | 0.282(-0.906-1.485) |
| Republic of Moldova | Male | 36061.22(30044.35-40331.61) | 36405.69(28520.18-43832.26) | 0.96 | 1772.55(1984.13-1474.47) | 1506.33(1811.27-1178.38) | 4.055(2.921-5.202) |
| Romania | Male | 138378.02(115700.87-155336.9) | 157302.84(117062.85-200441.38) | 13.68 | 1048.84(1177.32-879.97) | 1099.98(1400.81-821.73) | -2.205(-2.611--1.797) |
| Russian Federation | Male | 239092.98(193849.9-278429.02) | 782714.42(585462.38-989391.61) | 227.37 | 318.5(372.57-258.11) | 877.07(1107.37-656.46) | -2.572(-3.117--2.024) |
| Rwanda | Male | 37239.97(25136.13-49375.89) | 42282.88(28303.49-63910.88) | 13.54 | 2280.87(3022.89-1567.81) | 1200.32(1782.63-821.56) | -1.738(-1.998--1.478) |
| Saint Kitts and Nevis | Male | 133.71(86.03-173.02) | 171.53(104.61-273.65) | 28.29 | 933.59(1216.73-595.14) | 446.02(713.83-278.31) | -0.292(-0.62-0.038) |
| Saint Lucia | Male | 326.97(264.52-378.75) | 499.62(368.57-643.88) | 52.80 | 784.03(911.86-630.41) | 461.16(593.1-339.43) | -1.119(-1.187--1.051) |
| Saint Vincent and the Grenadines | Male | 179.87(136.84-224.59) | 346.32(261.94-439.64) | 92.54 | 530.15(663.42-401.9) | 489.33(622.1-368.17) | -0.762(-0.83--0.694) |
| Samoa | Male | 321.69(195.47-504.24) | 390.94(218.35-597.49) | 21.53 | 600.83(947.19-367.37) | 435.95(664.89-244.66) | -0.648(-1.195--0.099) |
| San Marino | Male | 52.04(32.14-68.57) | 73.15(38.78-111.15) | 40.56 | 363.13(477.55-221.46) | 288.56(448.9-152.91) | -4.067(-4.268--3.866) |
| Sao Tome and Principe | Male | 518.67(307.08-791.35) | 863.53(578.87-1293.94) | 66.49 | 1532.76(2335.07-913.6) | 1287.05(1872.84-872.95) | -1.065(-1.626--0.501) |
| Saudi Arabia | Male | 8369.49(2142.59-19094) | 8101.45(3643.31-18218.2) | -3.20 | 192.52(433.75-57.04) | 58.85(117.64-29.93) | -1.097(-1.492--0.701) |
| Senegal | Male | 9819.63(5764.59-16310.98) | 16255.03(9148.61-27597.17) | 65.54 | 499.06(829.03-295.16) | 362.74(619.9-208.73) | -0.212(-0.463-0.04) |
| Serbia | Male | 28915(22733.65-35969.63) | 23262.52(16338.94-31344.72) | -19.55 | 502.55(619.32-396.81) | 363.93(487.43-255.84) | -2.108(-2.426--1.789) |
| Seychelles | Male | 301.7(206.51-400.68) | 671.34(490.86-861.17) | 122.52 | 1087.85(1457.47-739.47) | 1015.1(1295.13-734.58) | -3.98(-4.376--3.583) |
| Sierra Leone | Male | 13992.8(9190.21-20437.02) | 16595.05(10119.34-25343.1) | 18.60 | 1272.09(1864.33-842.19) | 686.83(1052.45-424.05) | -1.612(-2.067--1.154) |
| Singapore | Male | 1697.74(1146.86-2256.26) | 1677.96(1074.64-2347.72) | -1.17 | 129.01(172.11-86.67) | 39.25(55.33-25.32) | -3.251(-3.751--2.748) |
| Slovakia | Male | 33927.42(27502.53-39676.77) | 31437.05(22864.51-42308.96) | -7.34 | 1270.29(1486.65-1026.82) | 808.45(1089.19-586.46) | 0.378(0.28-0.476) |
| Slovenia | Male | 13403.68(9408.53-17942.12) | 7927.98(5050.42-11693.96) | -40.85 | 1221.02(1629.87-857.35) | 460.56(682.29-293.65) | -0.485(-0.624--0.345) |
| Solomon Islands | Male | 449.41(261.26-710.9) | 1215.6(687.24-1956.24) | 170.49 | 425.77(677.68-250.56) | 476.42(761.89-280.61) | -1.968(-2.412--1.521) |
| Somalia | Male | 7423.51(3957.55-12122.27) | 16096.94(9059.22-26348.34) | 116.84 | 496.9(788.71-276.81) | 429.19(687.36-242.89) | -0.545(-0.641--0.449) |
| South Africa | Male | 87910.5(65599.42-117658.46) | 99534.55(78334.58-122403.29) | 13.22 | 720.45(974.18-529.68) | 414.11(511.13-325.93) | -3.595(-3.728--3.461) |
| South Sudan | Male | 5576.92(3236.98-10534.7) | 7424.58(4096.47-13064.75) | 33.13 | 368.6(692.32-215.98) | 316.36(556.98-177.58) | -0.729(-1.439--0.015) |
| Spain | Male | 176812.04(148199.42-199332.27) | 101445.93(79449.96-120474.09) | -42.62 | 788.05(888.39-660.54) | 275.49(326.34-216.28) | -5.1(-5.236--4.963) |
| Sri Lanka | Male | 60273.33(39584.34-84091.46) | 79500.86(52517.65-112894.57) | 31.90 | 853.03(1196.66-562.78) | 663.45(937.81-441.77) | -0.746(-1.287--0.202) |
| Sudan | Male | 13260.88(4664.46-26530.85) | 5501.86(2639.75-10588.27) | -58.51 | 231.75(462.06-83.73) | 50.29(97.1-23.64) | -0.968(-1.224--0.712) |
| Suriname | Male | 1041.12(785.26-1282.37) | 1774.77(1234.45-2372.55) | 70.47 | 721.6(889.77-542.81) | 577.55(772.31-404.48) | -2.082(-2.463--1.699) |
| Sweden | Male | 11204.44(9376.21-13006.87) | 12288.11(10060.83-14421.45) | 9.67 | 199.96(232.15-167.54) | 151.74(177.67-124.49) | -2.357(-2.757--1.954) |
| Switzerland | Male | 13995.16(11762.39-15793.95) | 12734.46(10153.32-14999.94) | -9.01 | 331.22(373.7-278.64) | 181.82(213.54-144.87) | -1.853(-2.183--1.522) |
| Syrian Arab Republic | Male | 7783.38(4033.07-13337.67) | 8062.17(3191.73-15505.52) | 3.58 | 228.66(392.45-119.77) | 115.43(220.37-46.19) | 0.413(0.108-0.719) |
| Taiwan (Province of China) | Male | 109044.43(91076.23-125095.96) | 111438.89(78636.36-154047.62) | 2.20 | 1143.2(1314.44-951.46) | 652.43(894.32-458.96) | -0.188(-0.383-0.006) |
| Tajikistan | Male | 10651.93(7746.07-13703.82) | 27259.51(18942.56-38295.44) | 155.91 | 671.09(863.24-479.27) | 745.74(1028.08-526) | 1.855(1.569-2.142) |
| Thailand | Male | 200564.62(151713.01-256772.48) | 378950.86(260939.72-525667.81) | 88.94 | 867.19(1101.51-661.47) | 811.28(1115.06-560.7) | -1.453(-1.751--1.154) |
| Timor-Leste | Male | 868.07(355.24-1801.79) | 2838.18(1238.26-4901.54) | 226.95 | 359.08(737.25-149.07) | 614.16(1069.3-275.07) | -1.085(-1.128--1.042) |
| Togo | Male | 8508.58(5625.77-12430.81) | 16187.21(8892.04-26108.86) | 90.25 | 1080.67(1577.93-706.17) | 702.65(1128.57-393.88) | 0.357(0.138-0.576) |
| Tokelau | Male | 2.78(1.42-4.41) | 2.4(1.27-3.78) | -13.67 | 490(781.02-249.39) | 356.68(562.82-188.77) | -1.655(-2.391--0.914) |
| Tonga | Male | 96.77(54.78-158.46) | 143.6(76.86-247.96) | 48.39 | 317.53(516.75-183.13) | 350.09(603.73-188.24) | 0.74(0.669-0.811) |
| Trinidad and Tobago | Male | 2363.51(1839.9-2878.28) | 2815.8(1877.35-3989.67) | 19.14 | 511.75(624.21-395.54) | 311.6(439.88-207.73) | -1.956(-2.316--1.595) |
| Tunisia | Male | 3255.76(1770.99-5298.48) | 8439.94(4285.62-14677.19) | 159.23 | 105.48(171.62-57.72) | 130.12(225.69-67.28) | 3.37(2.142-4.613) |
| Turkey | Male | 32025.16(17833.91-52040.63) | 40181.08(24649.73-61673.12) | 25.47 | 151.29(246.9-83.88) | 87.89(135.09-53.29) | -1.425(-1.485--1.366) |
| Turkmenistan | Male | 7758.03(5491.58-10498.18) | 45319.5(31963.93-60586.51) | 484.16 | 707.72(954.57-504.57) | 1812.3(2422.71-1277.81) | -0.767(-0.918--0.615) |
| Tuvalu | Male | 21.66(10.27-36.36) | 23.16(11.54-41.1) | 6.93 | 616.66(1031.71-295.85) | 405.92(720.96-204.01) | 4.907(2.76-7.099) |
| Uganda | Male | 48367.45(33678.01-69396.71) | 89528.38(57696.4-128245.62) | 85.10 | 1260.2(1791.41-885.34) | 1019.09(1445.95-653.14) | -3.624(-3.973--3.275) |
| Ukraine | Male | 113574.61(88883.82-138092.84) | 411135.06(300329.91-539198.63) | 262.00 | 399.09(485.69-313.33) | 1531.24(1998.44-1120.73) | 1.837(1.589-2.087) |
| United Arab Emirates | Male | 1138.32(669.27-1864.72) | 5101.43(2295.57-9512.27) | 348.15 | 239.42(384.63-134.62) | 83.5(155.49-37.28) | -1.214(-1.368--1.058) |
| United Kingdom | Male | 61720.82(52476-70283.78) | 137235.39(116277.8-158598.34) | 122.35 | 180.17(205.17-152.92) | 301.6(348.74-255.83) | 0.025(-0.215-0.265) |
| United Republic of Tanzania | Male | 78135.49(56739.84-101849.16) | 130772.96(85376.74-192209.79) | 67.37 | 1238.34(1600.01-900.24) | 867.53(1258.62-573.15) | -0.089(-0.438-0.261) |
| United States of America | Male | 519279.98(418932.95-614072.58) | 872523.41(714602.59-1027319.34) | 68.03 | 388.42(459.22-312.48) | 386.53(454.64-317.09) | -1.452(-1.768--1.135) |
| United States Virgin Islands | Male | 272.61(158.61-403.56) | 409.55(253.79-599.41) | 50.23 | 565.36(833.13-335.3) | 549.15(805.75-335.07) | 2.321(1.888-2.756) |
| Uruguay | Male | 7667.68(6131.51-9071.2) | 6319.19(4996.36-7680.73) | -17.59 | 455.83(538.92-365.5) | 299.88(364.52-236.87) | -1.071(-1.238--0.904) |
| Uzbekistan | Male | 41309.01(30234.59-53700.45) | 184585.37(133957.62-244083.06) | 346.84 | 662.7(861.65-488.76) | 1253.1(1651.35-935.51) | -0.959(-2.372-0.475) |
| Vanuatu | Male | 363.24(185.76-595.43) | 612.91(300.88-1089.95) | 68.73 | 716.35(1173.69-375.47) | 528.4(929.32-261.84) | 3.389(3.239-3.539) |
| Venezuela (Bolivarian Republic of) | Male | 38509.87(31400.43-44219.95) | 78037.58(50963.36-109264.61) | 102.64 | 678.2(778.44-547.17) | 520.67(726.28-343.33) | -3.474(-3.636--3.312) |
| Viet Nam | Male | 67343.96(40933.68-104988.92) | 467082.65(321247.48-625693.1) | 593.58 | 357.05(552.8-217.75) | 935.29(1238.93-648.27) | -0.083(-0.242-0.077) |
| Yemen | Male | 10412.04(4753.24-18062.66) | 10607.63(4354.26-20282.96) | 1.88 | 338.8(596.93-153.11) | 121.33(227.84-51.58) | -0.633(-1.163--0.101) |
| Zambia | Male | 29858.99(19653.23-43415.76) | 73969.55(47070.39-107190.03) | 147.73 | 1560.77(2295.28-1006.71) | 1507.23(2165.57-952.61) | |
| Zimbabwe | Male | 23161.14(16308.06-31093.28) | 31311.32(17078.52-51254.66) | 35.19 | 888.9(1192.58-620.41) | 725.89(1177.51-405.7) | |

**Table S7** The death cases and age-standardized death rate of liver cancer attributable to alcohol use in 1990 and 2019, and its temporal trends from 1990 to 2019.

| nation | sex | Death Case NO(95% UI) | | Change in absolute number (%) | age-standardized death rate per 100,000 No. (95% UI) | | 1990-2019 AAPC No.(95 CI) |
| --- | --- | --- | --- | --- | --- | --- | --- |
|  |  | 1990 | 2019 |  | 1990 | 2019 |  |
| Afghanistan | Both | 71.08(41.74-111.77) | 103.13(62.53-159.39) | 45.09 | 1.01(1.56-0.6) | 0.88(1.33-0.53) | -0.486(-0.541--0.431) |
| Albania | Both | 68.46(49.73-88.66) | 111.51(70.09-162.91) | 62.88 | 3.42(4.47-2.48) | 2.53(3.68-1.6) | -1.029(-1.352--0.706) |
| Algeria | Both | 19.44(11.59-29.87) | 72.21(42.78-110.9) | 271.45 | 0.17(0.25-0.1) | 0.23(0.35-0.13) | 1.027(0.74-1.316) |
| American Samoa | Both | 0.14(0.08-0.22) | 0.38(0.24-0.56) | 171.43 | 0.62(0.95-0.38) | 0.78(1.17-0.49) | 0.823(0.686-0.96) |
| Andorra | Both | 2.18(1.47-3.22) | 5.17(3.4-7.15) | 137.16 | 3.91(5.72-2.66) | 3.73(5.15-2.46) | -0.142(-0.203--0.082) |
| Angola | Both | 13.78(8.18-21.58) | 46.93(30.14-68.94) | 240.57 | 0.38(0.57-0.23) | 0.45(0.65-0.29) | 0.613(0.526-0.7) |
| Antigua and Barbuda | Both | 1.39(1.01-1.83) | 0.99(0.71-1.34) | -28.78 | 2.6(3.39-1.88) | 1(1.35-0.72) | -3.233(-3.914--2.547) |
| Argentina | Both | 166.49(115.67-220.04) | 385.48(275.21-497.43) | 131.53 | 0.51(0.68-0.36) | 0.71(0.91-0.5) | 1.129(0.812-1.447) |
| Armenia | Both | 6.91(4.83-9.31) | 85.47(59.48-116.86) | 1136.90 | 0.27(0.36-0.19) | 2.06(2.8-1.44) | 7.139(5.314-8.996) |
| Australia | Both | 163.5(123.84-199.78) | 659.29(497.27-823.05) | 303.24 | 0.82(1.01-0.63) | 1.62(2.01-1.23) | 2.37(2.194-2.546) |
| Austria | Both | 202.94(166.92-236.29) | 419.59(336.04-506.13) | 106.76 | 1.74(2.02-1.44) | 2.4(2.9-1.94) | 1.096(0.752-1.441) |
| Azerbaijan | Both | 11.13(7.69-15.05) | 100.4(62.08-151.86) | 802.07 | 0.24(0.32-0.16) | 1.16(1.76-0.72) | 5.705(5.083-6.331) |
| Bahamas | Both | 4.83(3.56-6.26) | 4.19(2.9-5.82) | -13.25 | 3.16(4.07-2.33) | 1.09(1.49-0.76) | -3.642(-4.069--3.213) |
| Bahrain | Both | 1.05(0.65-1.58) | 4.14(2.45-6.53) | 294.29 | 0.71(1.06-0.44) | 0.52(0.81-0.31) | -1.11(-1.689--0.527) |
| Bangladesh | Both | 330.25(220.94-471.2) | 742.07(457.23-1088.67) | 124.70 | 0.7(1-0.46) | 0.58(0.84-0.35) | -0.592(-0.764--0.42) |
| Barbados | Both | 2.87(1.81-4.37) | 5(3.51-6.67) | 74.22 | 0.96(1.45-0.6) | 1(1.33-0.7) | 0.049(-0.36-0.459) |
| Belarus | Both | 60.73(45.14-75.45) | 161.89(105-235.23) | 166.57 | 0.46(0.58-0.35) | 1.01(1.48-0.66) | 2.709(2.031-3.391) |
| Belgium | Both | 164.91(124.22-206.65) | 327.25(246.81-416.14) | 98.44 | 1.05(1.31-0.8) | 1.47(1.87-1.11) | 1.192(0.789-1.597) |
| Belize | Both | 1.79(1.29-2.33) | 2.94(2.14-3.83) | 64.25 | 1.96(2.55-1.41) | 1.13(1.48-0.82) | -1.838(-2.164--1.51) |
| Benin | Both | 24.31(15.89-37.35) | 46.16(28.79-72.03) | 89.88 | 1.25(1.93-0.82) | 1(1.55-0.63) | -0.762(-0.874--0.649) |
| Bermuda | Both | 1.8(1.35-2.3) | 1.05(0.76-1.4) | -41.67 | 2.89(3.67-2.16) | 0.79(1.06-0.57) | -4.376(-5.045--3.703) |
| Bhutan | Both | 1.64(0.95-2.77) | 5.12(2.98-8.13) | 212.20 | 0.66(1.09-0.4) | 0.94(1.48-0.54) | 1.169(0.998-1.341) |
| Bolivia (Plurinational State of) | Both | 44.62(28.16-66.88) | 131.94(86.36-192.01) | 195.70 | 1.47(2.18-0.94) | 1.57(2.27-1.02) | 0.237(0.097-0.376) |
| Bosnia and Herzegovina | Both | 76.98(55.46-100.35) | 167.74(113.76-232.31) | 117.90 | 1.89(2.47-1.39) | 2.73(3.77-1.86) | 1.261(0.906-1.618) |
| Botswana | Both | 1.07(0.4-3.06) | 4.63(2.8-7.3) | 332.71 | 0.19(0.54-0.07) | 0.33(0.5-0.2) | 2.034(1.745-2.324) |
| Brazil | Both | 499.54(432.74-569.83) | 1782.44(1533.76-2023.48) | 256.82 | 0.57(0.65-0.49) | 0.76(0.86-0.65) | 0.968(0.77-1.167) |
| Brunei Darussalam | Both | 0.9(0.56-1.35) | 3.16(1.93-4.88) | 251.11 | 0.96(1.45-0.6) | 1.06(1.57-0.65) | 0.361(-0.031-0.754) |
| Bulgaria | Both | 334.41(253.75-413.59) | 295.29(212.66-397.53) | -11.70 | 2.59(3.19-2) | 2.08(2.81-1.49) | -0.82(-1.371--0.266) |
| Burkina Faso | Both | 20.3(12.84-29.42) | 34.73(22.01-50.11) | 71.08 | 0.49(0.7-0.32) | 0.41(0.58-0.26) | -0.655(-0.771--0.538) |
| Burundi | Both | 29.58(18.95-43.71) | 38.59(21.43-69.54) | 30.46 | 1.29(1.91-0.83) | 0.89(1.58-0.51) | -1.256(-1.444--1.068) |
| Cabo Verde | Both | 0.46(0.3-0.66) | 9.96(6.83-13.74) | 2065.22 | 0.2(0.28-0.13) | 2.41(3.33-1.63) | 9.2(8.143-10.266) |
| Cambodia | Both | 54.96(35.36-82.94) | 190.27(121.26-274.51) | 246.20 | 1.25(1.92-0.8) | 1.63(2.34-1.04) | 0.967(0.834-1.1) |
| Cameroon | Both | 5.53(3.14-9.58) | 16.89(10.46-26.08) | 205.42 | 0.13(0.23-0.07) | 0.15(0.23-0.09) | 0.434(0.261-0.606) |
| Canada | Both | 349.57(299.48-397.77) | 1477.25(1184.13-1747.94) | 322.59 | 1.07(1.22-0.92) | 2.17(2.56-1.75) | 2.467(2.224-2.71) |
| Central African Republic | Both | 7.13(4.07-11.32) | 11.25(6.15-19.53) | 57.78 | 0.62(0.94-0.36) | 0.54(0.92-0.3) | -0.445(-0.656--0.234) |
| Chad | Both | 27.43(16.2-42.93) | 48.76(29.57-74.75) | 77.76 | 0.98(1.52-0.58) | 0.93(1.42-0.56) | -0.181(-0.283--0.079) |
| Chile | Both | 68.61(49.88-88.87) | 228.42(164.06-296.11) | 232.93 | 0.69(0.9-0.5) | 0.94(1.22-0.68) | 1.137(0.845-1.431) |
| China | Both | 23153.92(15271.45-33747.91) | 22032.63(15322.2-30742.6) | -4.84 | 2.54(3.63-1.72) | 1.08(1.49-0.75) | -2.895(-3.387--2.4) |
| Colombia | Both | 148.48(108.34-190.33) | 440.24(292.35-632.45) | 196.50 | 0.87(1.13-0.64) | 0.84(1.21-0.56) | -0.18(-0.972-0.618) |
| Comoros | Both | 1.49(0.7-3.09) | 2.86(1.59-5.53) | 91.95 | 0.68(1.42-0.34) | 0.61(1.16-0.34) | -0.419(-0.945-0.11) |
| Congo | Both | 6.9(4.04-10.94) | 12.98(7.7-20.4) | 88.12 | 0.65(1.01-0.39) | 0.52(0.8-0.32) | -0.735(-0.854--0.616) |
| Cook Islands | Both | 0.21(0.13-0.3) | 0.47(0.31-0.68) | 123.81 | 1.62(2.35-1) | 1.9(2.73-1.24) | 0.568(0.416-0.721) |
| Costa Rica | Both | 33.08(24.27-42.05) | 92.61(62.46-131) | 179.96 | 1.93(2.47-1.41) | 1.82(2.58-1.24) | -0.253(-0.94-0.438) |
| Croatia | Both | 89.12(69.03-108.45) | 146.51(103.3-197.15) | 64.40 | 1.38(1.69-1.08) | 1.66(2.22-1.17) | 0.65(0.111-1.191) |
| Cuba | Both | 226.97(168.79-289.93) | 173.41(118.66-236.51) | -23.60 | 2.18(2.77-1.63) | 0.9(1.23-0.62) | -2.884(-3.721--2.04) |
| Cyprus | Both | 9.8(6.72-13.34) | 24.43(17.6-31.88) | 149.29 | 1.18(1.59-0.84) | 1.24(1.61-0.9) | 0.116(-0.202-0.436) |
| Czechia | Both | 288.79(231.27-343.77) | 309.41(229.26-402.63) | 7.14 | 2.06(2.45-1.65) | 1.44(1.88-1.07) | -1.196(-1.886--0.501) |
| C么te d'Ivoire | Both | 59.07(35.37-89.96) | 106.95(64.88-165.64) | 81.06 | 1.55(2.36-0.96) | 1.09(1.67-0.67) | -1.247(-1.493--1) |
| Democratic People's Republic of Korea | Both | 236.55(136.56-380.37) | 321.66(190.36-503.82) | 35.98 | 1.34(2.12-0.8) | 0.98(1.52-0.58) | -1.085(-1.172--0.998) |
| Democratic Republic of the Congo | Both | 53.14(33.98-78.46) | 104.27(62.35-158.87) | 96.22 | 0.35(0.51-0.23) | 0.29(0.44-0.18) | -0.619(-0.785--0.451) |
| Denmark | Both | 62.88(48.05-78.05) | 147.09(109.39-187.2) | 133.92 | 0.77(0.95-0.6) | 1.28(1.63-0.95) | 1.802(1.657-1.948) |
| Djibouti | Both | 1.06(0.51-2.34) | 4.7(2.43-9.07) | 343.40 | 0.79(1.68-0.4) | 0.84(1.62-0.46) | 0.238(-0.016-0.492) |
| Dominica | Both | 2.2(1.59-2.89) | 1.11(0.77-1.54) | -49.55 | 3(3.9-2.16) | 1.22(1.7-0.84) | -3.033(-3.363--2.701) |
| Dominican Republic | Both | 49.45(35.94-64.75) | 158.4(94.75-265.75) | 220.32 | 1.35(1.77-0.98) | 1.73(2.87-1.04) | 0.837(0.57-1.105) |
| Ecuador | Both | 39.54(28-53.77) | 161.91(108.35-232.41) | 309.48 | 0.78(1.07-0.55) | 1.11(1.59-0.75) | 1.117(0.69-1.546) |
| Egypt | Both | 383.54(225.7-597) | 1199.79(655.54-2084.32) | 212.82 | 1.27(1.97-0.75) | 1.8(3.09-1) | 1.259(1.045-1.473) |
| El Salvador | Both | 26.52(19.15-34.54) | 39.32(25.47-56.2) | 48.27 | 0.91(1.19-0.65) | 0.67(0.96-0.43) | -1.14(-1.508--0.77) |
| Equatorial Guinea | Both | 0.86(0.51-1.35) | 2.82(1.44-4.7) | 227.91 | 0.44(0.67-0.26) | 0.63(1.03-0.32) | 1.288(1.088-1.487) |
| Eritrea | Both | 7.34(3.93-13.2) | 18.79(10.5-32.05) | 155.99 | 0.76(1.36-0.42) | 0.74(1.24-0.42) | -0.093(-0.387-0.203) |
| Estonia | Both | 15.49(11.49-19.42) | 41.64(29.5-54.89) | 168.82 | 0.75(0.94-0.56) | 1.6(2.13-1.13) | 2.76(1.785-3.745) |
| Eswatini | Both | 3.24(1.65-7.79) | 27.58(6.72-57.17) | 751.23 | 1.1(2.59-0.56) | 4.6(9.29-1.17) | 5.113(4.745-5.482) |
| Ethiopia | Both | 134.18(88.79-192.25) | 263.72(194.51-357.66) | 96.54 | 0.7(0.96-0.47) | 0.69(0.93-0.51) | -0.019(-0.164-0.125) |
| Fiji | Both | 2.58(1.62-3.95) | 6.39(3.83-10) | 147.67 | 0.73(1.12-0.46) | 0.85(1.31-0.52) | 0.538(0.166-0.912) |
| Finland | Both | 74.72(55.82-94.03) | 192.78(141.06-245.46) | 158.00 | 1.03(1.29-0.77) | 1.54(1.94-1.14) | 1.377(1.183-1.571) |
| France | Both | 1511.83(1110.2-1914.45) | 2569.3(1841.13-3420.71) | 69.95 | 1.87(2.37-1.38) | 1.98(2.61-1.41) | 0.127(-0.117-0.372) |
| Gabon | Both | 3.43(1.99-5.16) | 6.55(3.47-11.42) | 90.96 | 0.61(0.92-0.36) | 0.65(1.11-0.35) | 0.205(0.03-0.381) |
| Gambia | Both | 19.62(12.41-29.81) | 74.92(43.65-112.14) | 281.86 | 5.4(8.02-3.46) | 7.71(11.55-4.5) | 1.251(0.132-2.384) |
| Georgia | Both | 18.66(12.81-24.94) | 70.52(49.52-94.8) | 277.92 | 0.3(0.41-0.21) | 1.22(1.63-0.85) | 4.949(3.601-6.316) |
| Germany | Both | 1332.85(1082.89-1600.4) | 3557.39(2851.11-4233.87) | 166.90 | 1.05(1.25-0.85) | 1.89(2.25-1.53) | 2.068(1.867-2.269) |
| Ghana | Both | 73.95(46.33-115.75) | 212.17(137.09-310.21) | 186.91 | 1.23(1.91-0.78) | 1.37(1.98-0.89) | 0.385(0.219-0.551) |
| Greece | Both | 120.05(88.7-154.48) | 266.49(196.52-342.55) | 121.98 | 0.77(0.98-0.57) | 1.1(1.41-0.82) | 1.257(0.995-1.52) |
| Greenland | Both | 0.7(0.52-0.91) | 1.92(1.31-2.66) | 174.29 | 1.88(2.41-1.41) | 2.68(3.67-1.86) | 1.222(0.98-1.465) |
| Grenada | Both | 2.28(1.67-2.96) | 1.32(0.97-1.72) | -42.11 | 3.17(4.1-2.3) | 1.19(1.54-0.88) | -3.35(-3.958--2.738) |
| Guam | Both | 0.35(0.22-0.52) | 1.45(0.92-2.16) | 314.29 | 0.47(0.68-0.29) | 0.75(1.12-0.48) | 1.64(1.203-2.079) |
| Guatemala | Both | 85.17(58.63-114.2) | 130.05(85.58-188.22) | 52.69 | 2.44(3.26-1.68) | 1.2(1.73-0.78) | -2.4(-2.989--1.808) |
| Guinea | Both | 168.1(109.24-252.07) | 326.96(189.05-492.86) | 94.50 | 5.16(7.57-3.36) | 6.14(9.17-3.54) | 0.625(0.456-0.794) |
| Guinea-Bissau | Both | 5.76(3.4-9.26) | 7.91(4.74-12.15) | 37.33 | 1.45(2.3-0.87) | 1.15(1.75-0.71) | -0.844(-1.008--0.68) |
| Guyana | Both | 9.03(6.5-11.84) | 6.73(4.59-9.36) | -25.47 | 2.42(3.19-1.76) | 1.11(1.54-0.76) | -2.56(-3.04--2.077) |
| Haiti | Both | 70.33(40.67-111.38) | 96.08(51.41-162.54) | 36.61 | 2.25(3.66-1.28) | 1.47(2.5-0.8) | -1.477(-1.611--1.343) |
| Honduras | Both | 63.12(20.01-104.67) | 267.13(110.74-433.36) | 323.21 | 3.18(5.36-0.96) | 4.55(7.31-1.87) | 1.315(1.047-1.585) |
| Hungary | Both | 448.06(357.25-533.76) | 218.8(158.08-288.54) | -51.17 | 2.98(3.55-2.39) | 1.12(1.48-0.8) | -3.466(-4.162--2.766) |
| Iceland | Both | 1.72(1.22-2.24) | 5.7(4.18-7.4) | 231.40 | 0.6(0.78-0.43) | 1.04(1.35-0.77) | 1.95(1.526-2.375) |
| India | Both | 2907.88(2213.06-3672.34) | 8577.85(6764.53-10585.64) | 194.99 | 0.69(0.87-0.52) | 0.76(0.94-0.61) | 0.443(0.221-0.665) |
| Indonesia | Both | 417.09(330.23-520.8) | 877.81(678.08-1097.73) | 110.46 | 0.44(0.55-0.35) | 0.43(0.53-0.34) | -0.07(-0.226-0.085) |
| Iran (Islamic Republic of) | Both | 118.19(84.58-159.07) | 271.22(205.28-354.82) | 129.48 | 0.5(0.65-0.36) | 0.4(0.52-0.3) | -0.793(-1.208--0.377) |
| Iraq | Both | 32.43(19.75-50.07) | 128.68(77.67-195.13) | 296.79 | 0.43(0.66-0.26) | 0.6(0.91-0.37) | 1.139(0.523-1.759) |
| Ireland | Both | 21.68(15.98-27.81) | 95.14(69.9-122.6) | 338.84 | 0.51(0.66-0.38) | 1.26(1.61-0.93) | 3.085(2.645-3.526) |
| Israel | Both | 30.33(20.56-41.31) | 84.29(58.91-112.22) | 177.91 | 0.61(0.83-0.42) | 0.73(0.97-0.51) | 0.608(0.359-0.858) |
| Italy | Both | 1612.01(1399.6-1819.74) | 1733.73(1456.55-2015.92) | 7.55 | 1.79(2.03-1.55) | 1.26(1.46-1.06) | -1.292(-1.71--0.873) |
| Jamaica | Both | 23.8(17.76-30.97) | 27.26(18.72-37.54) | 14.54 | 1.32(1.72-0.99) | 0.93(1.28-0.64) | -1.235(-2.363--0.095) |
| Japan | Both | 2894.51(2334.17-3725.09) | 3930.08(3099.35-5113.42) | 35.78 | 1.67(2.16-1.35) | 1.09(1.4-0.88) | -1.471(-1.648--1.294) |
| Jordan | Both | 3.77(2.3-5.68) | 15.8(9.42-24.79) | 319.10 | 0.32(0.48-0.19) | 0.27(0.42-0.16) | -0.57(-0.876--0.262) |
| Kazakhstan | Both | 139.83(101.42-177.86) | 359.66(253.6-469.91) | 157.21 | 1.1(1.4-0.8) | 2.07(2.72-1.49) | 2.067(1.639-2.497) |
| Kenya | Both | 56.15(34.09-103.44) | 182.66(112.4-284.66) | 225.31 | 0.71(1.3-0.43) | 0.86(1.33-0.54) | 0.592(0.131-1.055) |
| Kiribati | Both | 0.56(0.33-0.86) | 0.89(0.52-1.33) | 58.93 | 1.43(2.21-0.89) | 1.22(1.79-0.73) | -0.555(-0.669--0.441) |
| Kuwait | Both | 1.61(1.04-2.37) | 5.51(3.31-8.57) | 242.24 | 0.29(0.43-0.18) | 0.26(0.4-0.15) | -0.081(-0.833-0.677) |
| Kyrgyzstan | Both | 10.09(7.06-13.44) | 35.26(24.61-46.61) | 249.45 | 0.34(0.45-0.24) | 0.81(1.08-0.57) | 3.128(2.67-3.588) |
| Lao People's Democratic Republic | Both | 44.84(26.33-70.77) | 72.88(45.93-109.12) | 62.53 | 2.13(3.3-1.28) | 1.7(2.51-1.11) | -0.773(-0.837--0.71) |
| Latvia | Both | 21.47(16.19-27.09) | 45.43(32.89-59.43) | 111.60 | 0.59(0.75-0.45) | 1.16(1.52-0.84) | 2.392(1.839-2.948) |
| Lebanon | Both | 10.13(6.31-15.23) | 20.06(11.47-33.34) | 98.03 | 0.46(0.68-0.29) | 0.38(0.63-0.22) | -0.647(-0.794--0.499) |
| Lesotho | Both | 9.95(4.75-24.44) | 46.18(15.4-78.36) | 364.12 | 1(2.45-0.49) | 3.58(6.02-1.23) | 4.584(4.371-4.798) |
| Liberia | Both | 14.99(9.27-23.57) | 19.36(11.64-31.36) | 29.15 | 1.36(2.13-0.85) | 1.01(1.65-0.61) | -1.037(-1.259--0.815) |
| Libya | Both | 8.89(5.36-13.9) | 24.13(14.03-39.61) | 171.43 | 0.5(0.79-0.3) | 0.5(0.8-0.28) | 0.033(-0.43-0.499) |
| Lithuania | Both | 26.44(19.49-32.87) | 75.74(53.19-98.67) | 186.46 | 0.58(0.73-0.43) | 1.36(1.78-0.96) | 2.952(2.234-3.674) |
| Luxembourg | Both | 6.64(4.98-8.22) | 13.93(9.92-18.78) | 109.79 | 1.2(1.48-0.91) | 1.41(1.91-1) | 0.525(0.356-0.695) |
| Madagascar | Both | 37.1(18.98-78.95) | 64.27(34.83-117.17) | 73.23 | 0.75(1.59-0.38) | 0.63(1.15-0.34) | -0.595(-0.948--0.24) |
| Malawi | Both | 29.23(16.71-51.04) | 51.67(33.89-73.95) | 76.77 | 0.77(1.33-0.45) | 0.76(1.08-0.5) | -0.032(-0.38-0.317) |
| Malaysia | Both | 67.12(43.75-99.17) | 223.17(137.09-348.15) | 232.49 | 0.76(1.14-0.49) | 0.85(1.31-0.52) | 0.484(0.253-0.716) |
| Maldives | Both | 1.23(0.68-2.17) | 3.54(2.34-5.08) | 187.80 | 1.53(2.62-0.88) | 1.26(1.83-0.82) | -0.674(-0.988--0.36) |
| Mali | Both | 106.3(65.36-157.95) | 227.81(139.07-351.15) | 114.31 | 2.46(3.65-1.53) | 2.59(3.9-1.59) | 0.182(-0.014-0.379) |
| Malta | Both | 2.3(1.66-2.96) | 7.33(5.16-9.64) | 218.70 | 0.53(0.68-0.38) | 0.78(1.02-0.56) | 1.348(0.9-1.797) |
| Marshall Islands | Both | 0.24(0.14-0.38) | 0.48(0.28-0.78) | 100.00 | 1.46(2.35-0.84) | 1.38(2.22-0.83) | -0.189(-0.336--0.043) |
| Mauritania | Both | 10.4(6.48-16.08) | 13.21(7.83-20.21) | 27.02 | 1.04(1.59-0.66) | 0.67(1.03-0.4) | -1.552(-1.661--1.442) |
| Mauritius | Both | 2.92(2.01-3.98) | 8.15(5.14-12.27) | 179.11 | 0.4(0.54-0.28) | 0.47(0.7-0.3) | 0.548(-0.054-1.155) |
| Mexico | Both | 262.06(228.2-297.64) | 1277.38(1023.66-1562.75) | 387.44 | 0.64(0.73-0.56) | 1.11(1.36-0.89) | 1.985(1.525-2.448) |
| Micronesia (Federated States of) | Both | 0.68(0.41-1.06) | 0.97(0.52-1.58) | 42.65 | 1.43(2.26-0.84) | 1.36(2.13-0.77) | -0.181(-0.304--0.058) |
| Monaco | Both | 0.77(0.51-1.07) | 2.49(1.71-3.41) | 223.38 | 1.11(1.53-0.74) | 2.68(3.71-1.84) | 3.101(2.759-3.446) |
| Mongolia | Both | 182.98(115.53-258.27) | 729.23(477.38-1054) | 298.53 | 17.55(24.44-11.24) | 34.21(47.83-23.12) | 2.286(2.052-2.52) |
| Montenegro | Both | 16.41(12.09-21.14) | 25.42(18.01-34.51) | 54.91 | 2.65(3.41-1.96) | 2.53(3.42-1.8) | -0.117(-0.326-0.093) |
| Morocco | Both | 28.19(16.62-44.12) | 75.79(45.46-117.02) | 168.85 | 0.22(0.35-0.13) | 0.27(0.41-0.16) | 0.506(-0.008-1.024) |
| Mozambique | Both | 26.26(14.5-51.74) | 99.57(57.61-146.43) | 279.17 | 0.48(0.95-0.27) | 0.99(1.45-0.57) | 2.551(2.367-2.735) |
| Myanmar | Both | 117.23(71.35-197.66) | 394.51(254.66-571.87) | 236.53 | 0.54(0.89-0.33) | 0.89(1.27-0.59) | 1.821(1.177-2.469) |
| Namibia | Both | 2.76(1.32-6.29) | 11.92(7.52-17.09) | 331.88 | 0.38(0.85-0.18) | 0.85(1.21-0.54) | 2.859(2.546-3.172) |
| Nauru | Both | 0.05(0.03-0.08) | 0.06(0.03-0.09) | 20.00 | 1.36(2.04-0.81) | 1.3(1.97-0.77) | -0.144(-0.26--0.027) |
| Nepal | Both | 43.28(29.09-61.69) | 134.32(78.83-220.06) | 210.35 | 0.51(0.73-0.34) | 0.64(1.04-0.38) | 0.815(0.671-0.959) |
| Netherlands | Both | 111.49(88.09-135.35) | 379.23(288.99-470.19) | 240.15 | 0.56(0.67-0.44) | 1.11(1.36-0.86) | 2.384(1.961-2.81) |
| New Zealand | Both | 33.64(29.1-38.04) | 106.19(92.26-120.52) | 215.67 | 0.87(0.98-0.75) | 1.41(1.6-1.23) | 1.705(1.475-1.935) |
| Nicaragua | Both | 13.72(9.4-18.32) | 53.35(36.28-74.95) | 288.85 | 0.92(1.24-0.62) | 1.26(1.75-0.85) | 1.084(0.356-1.816) |
| Niger | Both | 2.95(1.78-4.45) | 7.69(4.54-11.91) | 160.68 | 0.11(0.17-0.07) | 0.11(0.16-0.06) | -0.182(-0.369-0.005) |
| Nigeria | Both | 295.33(207.15-405.7) | 614.98(451.8-813.88) | 108.23 | 0.7(0.96-0.5) | 0.81(1.07-0.61) | 0.508(0.411-0.606) |
| Niue | Both | 0.02(0.01-0.03) | 0.02(0.01-0.03) | 0.00 | 1.06(1.57-0.67) | 1.06(1.57-0.68) | -0.004(-0.042-0.035) |
| North Macedonia | Both | 61.48(45.3-78.94) | 105.26(70.26-148.01) | 71.21 | 3.3(4.21-2.44) | 3.2(4.47-2.15) | -0.06(-0.293-0.174) |
| Northern Mariana Islands | Both | 0.14(0.09-0.22) | 0.54(0.34-0.83) | 285.71 | 0.78(1.16-0.48) | 0.99(1.44-0.65) | 0.814(0.515-1.113) |
| Norway | Both | 31.89(27.93-36.19) | 76.89(64.23-90.8) | 141.11 | 0.48(0.54-0.42) | 0.82(0.97-0.69) | 1.901(1.668-2.134) |
| Oman | Both | 2.78(1.55-4.41) | 8.51(5.24-13.44) | 206.12 | 0.43(0.67-0.24) | 0.52(0.81-0.33) | 0.67(0.355-0.987) |
| Pakistan | Both | 309.91(195.22-435.29) | 617.75(461.84-826.25) | 99.33 | 0.54(0.76-0.34) | 0.54(0.72-0.41) | 0.037(-0.13-0.204) |
| Palau | Both | 0.13(0.08-0.22) | 0.32(0.19-0.48) | 146.15 | 1.32(2.17-0.76) | 1.39(2.08-0.87) | 0.237(0.093-0.381) |
| Palestine | Both | 6.37(3.67-9.87) | 13.18(8.16-19.73) | 106.91 | 0.78(1.2-0.44) | 0.62(0.93-0.38) | -0.804(-1.187--0.419) |
| Panama | Both | 15.84(11.73-20.33) | 43.67(29.25-63.21) | 175.69 | 1.07(1.38-0.78) | 1.06(1.54-0.71) | 0.065(-0.328-0.46) |
| Papua New Guinea | Both | 3.12(1.85-4.96) | 9(5.42-14.59) | 188.46 | 0.19(0.3-0.12) | 0.23(0.37-0.15) | 0.648(0.585-0.712) |
| Paraguay | Both | 18.37(12.84-23.77) | 39.84(25.3-57.22) | 116.88 | 0.85(1.11-0.59) | 0.74(1.07-0.47) | -0.441(-0.713--0.168) |
| Peru | Both | 230.54(163.53-308.71) | 300.33(194.66-424.47) | 30.27 | 2.03(2.73-1.44) | 0.95(1.35-0.61) | -2.542(-3.336--1.74) |
| Philippines | Both | 671.4(461.55-884.82) | 1258.62(940.77-1644.3) | 87.46 | 2.21(2.91-1.51) | 1.58(2.05-1.19) | -1.118(-1.271--0.966) |
| Poland | Both | 1167.1(1032.65-1305.42) | 650.18(528.78-799.94) | -44.29 | 2.67(2.99-2.37) | 0.91(1.13-0.74) | -3.555(-4.206--2.898) |
| Portugal | Both | 101(76.48-125.42) | 409.25(302.85-513.97) | 305.20 | 0.71(0.88-0.55) | 1.83(2.3-1.37) | 3.253(2.981-3.527) |
| Puerto Rico | Both | 89.54(66.04-113.61) | 67.21(44.33-95.93) | -24.94 | 2.42(3.07-1.8) | 0.95(1.36-0.62) | -3.159(-3.82--2.494) |
| Qatar | Both | 1.52(0.88-2.37) | 10.49(5.69-17.06) | 590.13 | 1.87(2.96-1.08) | 1.8(2.9-1.02) | -0.142(-1.464-1.198) |
| Republic of Korea | Both | 531.98(335.03-785.15) | 2750.2(1939.04-3745.58) | 416.97 | 1.7(2.46-1.1) | 3.05(4.16-2.16) | 2.039(1.639-2.441) |
| Republic of Moldova | Both | 37.67(31.29-43.81) | 67.48(53.05-84.15) | 79.13 | 0.85(0.99-0.71) | 1.16(1.45-0.92) | 0.946(-0.882-2.807) |
| Romania | Both | 205.99(153.24-258.06) | 498.61(359.78-649.93) | 142.06 | 0.74(0.91-0.56) | 1.37(1.79-0.98) | 2.205(1.677-2.735) |
| Russian Federation | Both | 1041.74(905.97-1185.23) | 2550.75(2035.15-3203.28) | 144.85 | 0.57(0.65-0.5) | 1.09(1.37-0.87) | 2.245(1.648-2.845) |
| Rwanda | Both | 48.54(30.95-73.41) | 85.74(57.28-124.61) | 76.64 | 1.7(2.51-1.12) | 1.46(2.08-1.01) | -0.529(-0.746--0.312) |
| Saint Kitts and Nevis | Both | 1.63(1.17-2.13) | 0.85(0.59-1.18) | -47.85 | 4.15(5.4-3.01) | 1.32(1.81-0.93) | -4.114(-4.7--3.524) |
| Saint Lucia | Both | 2.07(1.56-2.63) | 1.91(1.4-2.5) | -7.73 | 2.34(2.97-1.77) | 0.9(1.17-0.66) | -3.18(-3.744--2.613) |
| Saint Vincent and the Grenadines | Both | 1.78(1.28-2.31) | 1.61(1.18-2.07) | -9.55 | 2.43(3.16-1.78) | 1.19(1.52-0.88) | -2.393(-2.99--1.794) |
| Samoa | Both | 0.74(0.43-1.14) | 1.01(0.63-1.5) | 36.49 | 0.83(1.27-0.5) | 0.69(1.02-0.43) | -0.669(-0.765--0.573) |
| San Marino | Both | 0.26(0.18-0.35) | 0.66(0.38-1.01) | 153.85 | 0.76(1.01-0.54) | 1.05(1.63-0.6) | 1.159(1.066-1.252) |
| Sao Tome and Principe | Both | 0.28(0.18-0.41) | 0.62(0.36-0.97) | 121.43 | 0.45(0.64-0.29) | 0.61(0.93-0.36) | 1.045(0.772-1.319) |
| Saudi Arabia | Both | 25.45(14.72-41.49) | 59.2(34.48-95.49) | 132.61 | 0.47(0.76-0.27) | 0.42(0.67-0.24) | -0.428(-0.569--0.286) |
| Senegal | Both | 10.05(6.48-14.71) | 21.29(12.88-31.67) | 111.84 | 0.33(0.47-0.21) | 0.3(0.45-0.18) | -0.235(-0.562-0.092) |
| Serbia | Both | 250.54(179.87-326.29) | 333.7(225.16-462.35) | 33.19 | 2.19(2.85-1.57) | 2.03(2.79-1.37) | -0.2(-0.426-0.026) |
| Seychelles | Both | 0.94(0.62-1.35) | 1.61(1.07-2.25) | 71.28 | 1.66(2.38-1.1) | 1.46(2.03-0.97) | -0.455(-0.701--0.209) |
| Sierra Leone | Both | 22.11(14.18-33.97) | 31.79(19.49-48.31) | 43.78 | 1.17(1.8-0.76) | 0.93(1.41-0.58) | -0.775(-1.036--0.514) |
| Singapore | Both | 13.69(9.07-19.54) | 53.91(34.15-80.77) | 293.79 | 0.63(0.91-0.41) | 0.7(1.06-0.45) | 0.362(-0.037-0.762) |
| Slovakia | Both | 123.41(96.03-148.63) | 142.32(98.87-192.83) | 15.32 | 2.04(2.46-1.61) | 1.52(2.05-1.05) | -1.107(-1.369--0.844) |
| Slovenia | Both | 28.66(19.75-39.53) | 87.94(59.66-124.38) | 206.84 | 1.17(1.6-0.8) | 2.06(2.91-1.39) | 1.983(1.369-2.601) |
| Solomon Islands | Both | 1.02(0.6-1.66) | 1.93(1.17-2.91) | 89.22 | 0.76(1.23-0.46) | 0.66(0.98-0.41) | -0.509(-0.786--0.232) |
| Somalia | Both | 19.35(10.03-41.88) | 46.27(24.61-88.26) | 139.12 | 0.82(1.75-0.44) | 0.74(1.43-0.4) | -0.335(-0.506--0.164) |
| South Africa | Both | 263.89(156.46-505.76) | 557.08(451.14-668.65) | 111.10 | 1.24(2.37-0.73) | 1.24(1.48-1) | 0.026(-0.604-0.661) |
| South Sudan | Both | 17.65(8.53-39.51) | 23.85(11.32-49.07) | 35.13 | 0.76(1.65-0.37) | 0.68(1.41-0.33) | -0.376(-0.494--0.258) |
| Spain | Both | 673.89(475.31-874.14) | 1325.14(918.21-1791.53) | 96.64 | 1.21(1.56-0.86) | 1.45(1.93-1) | 0.609(0.483-0.736) |
| Sri Lanka | Both | 45.63(30.1-64.64) | 180.98(110.41-275.94) | 296.63 | 0.45(0.64-0.3) | 0.7(1.05-0.43) | 1.62(1.153-2.089) |
| Sudan | Both | 44.55(22.16-85.52) | 95(49.29-179.34) | 113.24 | 0.5(0.95-0.25) | 0.57(1.07-0.3) | 0.43(0.291-0.57) |
| Suriname | Both | 5.75(4.15-7.48) | 5.67(4.01-7.97) | -1.39 | 2.28(2.97-1.65) | 0.96(1.34-0.68) | -2.979(-3.441--2.514) |
| Sweden | Both | 150.15(130.12-170.33) | 272.27(234.83-309.18) | 81.33 | 1(1.13-0.87) | 1.35(1.53-1.16) | 1.037(0.779-1.296) |
| Switzerland | Both | 106.82(82.05-129.83) | 306.88(227.23-388.9) | 187.29 | 1.03(1.26-0.79) | 1.8(2.3-1.35) | 1.898(1.617-2.179) |
| Syrian Arab Republic | Both | 23.47(14.07-35.62) | 47.87(27.03-77.2) | 103.96 | 0.48(0.75-0.29) | 0.41(0.65-0.24) | -0.569(-0.792--0.346) |
| Taiwan (Province of China) | Both | 252.97(168.77-355.8) | 285.79(174.3-448.91) | 12.97 | 1.47(2.08-0.99) | 0.72(1.13-0.44) | -2.597(-3.337--1.851) |
| Tajikistan | Both | 5.95(3.96-8.15) | 38.66(25.02-56.09) | 549.75 | 0.22(0.31-0.15) | 0.89(1.3-0.57) | 4.923(4.644-5.204) |
| Thailand | Both | 1937.24(1336.41-2659.23) | 7409.16(4775.8-10917.41) | 282.46 | 5.45(7.45-3.77) | 7.2(10.47-4.66) | 0.877(0.608-1.146) |
| Timor-Leste | Both | 3.81(2.1-6.24) | 10.79(5.63-17.79) | 183.20 | 1.4(2.27-0.8) | 1.33(2.16-0.72) | -0.165(-0.347-0.017) |
| Togo | Both | 13.94(8.6-21.51) | 32.83(20.4-49.58) | 135.51 | 1.17(1.79-0.73) | 0.94(1.39-0.6) | -0.763(-0.858--0.668) |
| Tokelau | Both | 0.01(0.01-0.02) | 0.01(0.01-0.02) | 0.00 | 0.97(1.5-0.57) | 0.91(1.39-0.53) | -0.213(-0.261--0.164) |
| Tonga | Both | 1.65(0.92-2.6) | 2.63(1.56-4.1) | 59.39 | 2.93(4.54-1.68) | 3.34(5.2-1.97) | 0.486(0.333-0.638) |
| Trinidad and Tobago | Both | 19.15(14.11-24.48) | 17.07(11.12-24.47) | -10.86 | 2.28(2.9-1.67) | 0.92(1.32-0.6) | -3.125(-3.542--2.707) |
| Tunisia | Both | 10.42(5.68-17.37) | 26.36(13.58-47.68) | 152.98 | 0.21(0.35-0.12) | 0.21(0.38-0.11) | -0.024(-0.203-0.154) |
| Turkey | Both | 163.6(105.62-240.8) | 337.68(210.24-515.27) | 106.41 | 0.49(0.73-0.31) | 0.39(0.6-0.24) | -0.739(-0.939--0.54) |
| Turkmenistan | Both | 4.8(3.29-6.45) | 69.09(45.36-98.2) | 1339.38 | 0.27(0.36-0.18) | 1.71(2.43-1.14) | 6.596(5.665-7.535) |
| Tuvalu | Both | 0.09(0.05-0.13) | 0.11(0.06-0.18) | 22.22 | 1.21(1.89-0.71) | 1.07(1.69-0.64) | -0.405(-0.454--0.356) |
| Uganda | Both | 103.25(68.68-140.98) | 292.49(199.14-414.31) | 183.28 | 1.59(2.15-1.07) | 2.07(2.85-1.42) | 0.896(0.692-1.1) |
| Ukraine | Both | 212.8(183.38-241.27) | 699.74(562.54-856.44) | 228.83 | 0.29(0.33-0.25) | 0.92(1.13-0.74) | 3.946(3.581-4.313) |
| United Arab Emirates | Both | 2.2(0.92-4.61) | 21.84(6.92-58.82) | 892.73 | 0.58(1.31-0.23) | 0.61(1.63-0.2) | 0.129(-0.366-0.627) |
| United Kingdom | Both | 602.49(534.89-671.35) | 1823.05(1598.35-2052.59) | 202.59 | 0.66(0.74-0.59) | 1.45(1.63-1.28) | 2.723(2.46-2.986) |
| United Republic of Tanzania | Both | 60.1(39.77-87.71) | 155.11(101.49-223.88) | 158.09 | 0.58(0.83-0.39) | 0.68(0.98-0.44) | 0.572(0.171-0.974) |
| United States of America | Both | 1877.29(1659.48-2090.72) | 7395.44(6010.49-8782.18) | 293.94 | 0.59(0.65-0.52) | 1.35(1.6-1.1) | 2.908(2.793-3.023) |
| United States Virgin Islands | Both | 1.11(0.79-1.52) | 1.74(1.23-2.41) | 56.76 | 1.33(1.81-0.94) | 0.9(1.22-0.64) | -1.261(-1.625--0.897) |
| Uruguay | Both | 16.3(11.46-21.96) | 38.29(26.81-50.16) | 134.91 | 0.41(0.55-0.29) | 0.72(0.94-0.51) | 2.004(1.528-2.483) |
| Uzbekistan | Both | 23.7(16.8-31.27) | 375.92(252.66-523.14) | 1486.16 | 0.22(0.3-0.15) | 1.87(2.55-1.28) | 7.735(7.33-8.143) |
| Vanuatu | Both | 0.76(0.39-1.32) | 2.11(1.13-3.48) | 177.63 | 1.18(2.06-0.6) | 1.22(2.03-0.66) | 0.094(-0.07-0.258) |
| Venezuela (Bolivarian Republic of) | Both | 213.29(156.67-268.13) | 243.94(161.78-347.33) | 14.37 | 2.27(2.88-1.66) | 0.86(1.21-0.57) | -3.507(-4.473--2.531) |
| Viet Nam | Both | 286.8(173.86-444.44) | 577.37(354.45-846.64) | 101.31 | 0.72(1.12-0.44) | 0.66(0.97-0.41) | -0.312(-0.471--0.152) |
| Yemen | Both | 9.74(5.15-17.62) | 28.81(16.38-48.46) | 195.79 | 0.21(0.38-0.11) | 0.24(0.4-0.13) | 0.4(0.343-0.457) |
| Zambia | Both | 17.78(10.59-31.72) | 50.22(32.95-70.52) | 182.45 | 0.69(1.23-0.41) | 0.83(1.16-0.55) | 0.642(0.457-0.829) |
| Zimbabwe | Both | 86.82(50.14-168.41) | 154.86(93.32-257.57) | 78.37 | 2.07(3.94-1.22) | 2.16(3.51-1.32) | 0.269(-0.015-0.554) |
| Afghanistan | Female | 18.43(10.34-29.43) | 33.37(18.94-54.89) | 81.06 | 0.52(0.82-0.3) | 0.49(0.8-0.28) | -0.113(-0.182--0.045) |
| Albania | Female | 13.19(8.85-18.93) | 14.98(8.63-23.2) | 13.57 | 1.26(1.81-0.84) | 0.65(1.01-0.38) | -2.304(-2.609--1.999) |
| Algeria | Female | 3.76(2.17-6.08) | 11.74(6.67-18.55) | 212.23 | 0.06(0.1-0.04) | 0.07(0.12-0.04) | 0.54(0.249-0.832) |
| American Samoa | Female | 0.04(0.02-0.05) | 0.08(0.05-0.13) | 100.00 | 0.33(0.5-0.21) | 0.34(0.53-0.2) | -0.088(-0.371-0.196) |
| Andorra | Female | 0.33(0.2-0.55) | 0.88(0.5-1.4) | 166.67 | 1.25(2.05-0.75) | 1.26(2.02-0.71) | 0.029(-0.14-0.199) |
| Angola | Female | 3.04(1.66-4.95) | 11.2(6.47-17.87) | 268.42 | 0.15(0.25-0.08) | 0.18(0.28-0.11) | 0.569(0.345-0.794) |
| Antigua and Barbuda | Female | 0.31(0.21-0.44) | 0.26(0.17-0.37) | -16.13 | 1.01(1.42-0.67) | 0.5(0.71-0.33) | -2.405(-2.804--2.003) |
| Argentina | Female | 39.08(25.77-55.92) | 66.74(44.75-94.7) | 70.78 | 0.21(0.31-0.14) | 0.21(0.3-0.14) | -0.002(-0.188-0.185) |
| Armenia | Female | 1.93(1.23-2.76) | 22.6(14.49-32.42) | 1070.98 | 0.13(0.19-0.08) | 0.93(1.33-0.6) | 6.99(5.087-8.927) |
| Australia | Female | 25.39(17.13-35.16) | 115.97(76.57-160.29) | 356.75 | 0.23(0.32-0.16) | 0.53(0.72-0.35) | 2.831(2.568-3.094) |
| Austria | Female | 36.37(23.73-50.33) | 61.97(41.34-85.98) | 70.39 | 0.47(0.64-0.32) | 0.63(0.87-0.43) | 0.971(0.688-1.255) |
| Azerbaijan | Female | 3.15(2.05-4.53) | 24.3(14.1-37.8) | 671.43 | 0.11(0.16-0.07) | 0.53(0.81-0.3) | 5.636(5.031-6.245) |
| Bahamas | Female | 1.18(0.81-1.59) | 1.03(0.68-1.46) | -12.71 | 1.38(1.88-0.95) | 0.49(0.7-0.33) | -3.452(-3.952--2.949) |
| Bahrain | Female | 0.19(0.11-0.29) | 0.64(0.36-1) | 236.84 | 0.27(0.43-0.16) | 0.22(0.34-0.12) | -0.752(-1.254--0.247) |
| Bangladesh | Female | 32.02(18.71-51.16) | 97.72(57.64-152.01) | 205.18 | 0.15(0.24-0.09) | 0.16(0.24-0.09) | 0.236(0.023-0.449) |
| Barbados | Female | 0.87(0.56-1.27) | 1.58(1.07-2.22) | 81.61 | 0.49(0.7-0.32) | 0.57(0.81-0.39) | 0.473(0.078-0.869) |
| Belarus | Female | 20.58(14.24-27.67) | 46.78(29.6-67.04) | 127.31 | 0.24(0.33-0.17) | 0.46(0.66-0.29) | 2.244(1.412-3.082) |
| Belgium | Female | 41.9(27.67-58.33) | 57.94(37.49-83.87) | 38.28 | 0.45(0.61-0.3) | 0.45(0.64-0.3) | 0.027(-0.408-0.463) |
| Belize | Female | 0.65(0.44-0.9) | 0.73(0.49-1.01) | 12.31 | 1.42(1.96-0.95) | 0.56(0.77-0.37) | -3.222(-3.651--2.791) |
| Benin | Female | 4.98(2.89-7.77) | 10.87(6.58-17.15) | 118.27 | 0.49(0.77-0.29) | 0.43(0.67-0.26) | -0.455(-0.549--0.361) |
| Bermuda | Female | 0.54(0.37-0.74) | 0.23(0.15-0.34) | -57.41 | 1.52(2.08-1.04) | 0.31(0.45-0.2) | -5.32(-5.945--4.692) |
| Bhutan | Female | 0.21(0.11-0.34) | 0.61(0.33-1.11) | 190.48 | 0.17(0.29-0.09) | 0.23(0.42-0.13) | 1.014(0.94-1.089) |
| Bolivia (Plurinational State of) | Female | 21.15(11.67-36.88) | 58.79(34.32-93.88) | 177.97 | 1.31(2.26-0.73) | 1.33(2.07-0.79) | 0.044(-0.075-0.163) |
| Bosnia and Herzegovina | Female | 14.56(9.53-20.8) | 46(28.89-68.63) | 215.93 | 0.65(0.92-0.42) | 1.34(1.97-0.85) | 2.496(2.102-2.89) |
| Botswana | Female | 0.32(0.15-0.59) | 0.93(0.51-1.5) | 190.63 | 0.1(0.19-0.05) | 0.12(0.19-0.07) | 0.55(0.335-0.765) |
| Brazil | Female | 153.83(129.62-177.78) | 429.7(362.67-497.14) | 179.33 | 0.33(0.38-0.28) | 0.33(0.38-0.28) | 0.05(-0.204-0.306) |
| Brunei Darussalam | Female | 0.17(0.1-0.28) | 0.63(0.37-1) | 270.59 | 0.4(0.63-0.23) | 0.43(0.67-0.26) | 0.281(-0.109-0.674) |
| Bulgaria | Female | 89.02(60.66-120.93) | 68.34(44.79-97.77) | -23.23 | 1.28(1.72-0.88) | 0.82(1.16-0.54) | -1.614(-2.162--1.062) |
| Burkina Faso | Female | 5.66(3.36-8.8) | 11.48(6.78-17.12) | 102.83 | 0.25(0.39-0.15) | 0.23(0.35-0.14) | -0.25(-0.542-0.043) |
| Burundi | Female | 8.58(4.98-13.85) | 9.98(6.03-15.77) | 16.32 | 0.68(1.09-0.4) | 0.47(0.75-0.28) | -1.252(-1.507--0.996) |
| Cabo Verde | Female | 0.13(0.08-0.19) | 2.32(1.39-3.62) | 1684.62 | 0.09(0.15-0.06) | 1.02(1.62-0.59) | 8.613(7.933-9.298) |
| Cambodia | Female | 16.8(9.84-26.79) | 53.95(31.7-83.92) | 221.13 | 0.63(1.01-0.38) | 0.77(1.2-0.46) | 0.69(0.523-0.858) |
| Cameroon | Female | 1.22(0.71-1.92) | 3.2(1.83-5.14) | 162.30 | 0.06(0.09-0.03) | 0.05(0.09-0.03) | -0.124(-0.252-0.005) |
| Canada | Female | 67.66(49.94-87.65) | 270.9(183.44-365.66) | 300.38 | 0.37(0.48-0.27) | 0.73(0.97-0.5) | 2.451(2.148-2.756) |
| Central African Republic | Female | 1.5(0.79-2.76) | 2.52(1.2-4.85) | 68.00 | 0.23(0.41-0.13) | 0.21(0.4-0.1) | -0.375(-0.586--0.163) |
| Chad | Female | 6.07(3.64-9.43) | 10.24(6.07-16.4) | 68.70 | 0.42(0.66-0.25) | 0.41(0.68-0.24) | -0.044(-0.21-0.121) |
| Chile | Female | 15.8(10.52-22.17) | 53.86(36.16-77.31) | 240.89 | 0.29(0.41-0.19) | 0.4(0.58-0.27) | 1.151(0.789-1.514) |
| China | Female | 5611.65(4010.52-7666.45) | 4939.15(3495.84-6652.15) | -11.98 | 1.27(1.71-0.91) | 0.46(0.62-0.33) | -3.417(-3.766--3.067) |
| Colombia | Female | 53.87(36.01-74.17) | 111.74(68.83-165.7) | 107.43 | 0.62(0.86-0.41) | 0.39(0.58-0.24) | -1.657(-2.14--1.171) |
| Comoros | Female | 0.39(0.21-0.64) | 0.85(0.5-1.34) | 117.95 | 0.35(0.56-0.19) | 0.32(0.51-0.19) | -0.243(-0.71-0.226) |
| Congo | Female | 1.6(0.94-2.61) | 3.2(1.71-5.21) | 100.00 | 0.27(0.43-0.16) | 0.23(0.37-0.13) | -0.482(-0.665--0.298) |
| Cook Islands | Female | 0.05(0.03-0.08) | 0.08(0.05-0.12) | 60.00 | 0.8(1.29-0.48) | 0.61(0.96-0.37) | -0.961(-1.415--0.506) |
| Costa Rica | Female | 8.63(5.84-11.97) | 21.05(12.85-31.86) | 143.92 | 0.98(1.37-0.65) | 0.77(1.17-0.47) | -0.91(-1.359--0.46) |
| Croatia | Female | 29.18(20.58-38.09) | 31.84(20.56-45.72) | 9.12 | 0.75(0.98-0.53) | 0.6(0.85-0.4) | -0.872(-1.395--0.346) |
| Cuba | Female | 70.7(48.69-96.27) | 34.74(22.98-49.8) | -50.86 | 1.34(1.81-0.92) | 0.34(0.49-0.22) | -4.697(-5.12--4.271) |
| Cyprus | Female | 1.63(0.98-2.48) | 4.34(2.64-6.47) | 166.26 | 0.4(0.59-0.25) | 0.42(0.62-0.26) | 0.134(-0.26-0.53) |
| Czechia | Female | 76.16(53.52-99.97) | 70.49(47.62-98.34) | -7.44 | 0.91(1.17-0.64) | 0.58(0.81-0.4) | -1.588(-2.08--1.093) |
| C么te d'Ivoire | Female | 7.87(4.78-12.11) | 19.04(11.08-29.7) | 141.93 | 0.45(0.69-0.28) | 0.4(0.62-0.24) | -0.394(-0.603--0.184) |
| Democratic People's Republic of Korea | Female | 55.54(31.08-92.71) | 68.78(37.91-109.58) | 23.84 | 0.56(0.92-0.32) | 0.37(0.58-0.2) | -1.487(-1.625--1.35) |
| Democratic Republic of the Congo | Female | 14.29(8.6-22.36) | 31.16(17.75-50.91) | 118.05 | 0.16(0.25-0.1) | 0.16(0.25-0.09) | -0.16(-0.217--0.102) |
| Denmark | Female | 12.94(8.64-17.98) | 25(16.12-35.56) | 93.20 | 0.28(0.38-0.19) | 0.41(0.57-0.27) | 1.266(0.858-1.676) |
| Djibouti | Female | 0.28(0.17-0.45) | 1.04(0.6-1.74) | 271.43 | 0.43(0.68-0.26) | 0.4(0.65-0.23) | -0.249(-0.467--0.032) |
| Dominica | Female | 0.73(0.49-1.02) | 0.27(0.18-0.4) | -63.01 | 1.66(2.3-1.13) | 0.58(0.85-0.38) | -3.678(-4.166--3.187) |
| Dominican Republic | Female | 14.5(9.79-20.18) | 38.96(23.24-60.16) | 168.69 | 0.8(1.11-0.54) | 0.82(1.27-0.49) | 0.106(-0.193-0.406) |
| Ecuador | Female | 17.81(12.11-25.29) | 68.15(44.79-98.76) | 282.65 | 0.71(1-0.48) | 0.89(1.29-0.59) | 0.809(0.492-1.128) |
| Egypt | Female | 44.76(24.88-72.19) | 134.95(72.69-240.33) | 201.50 | 0.31(0.5-0.17) | 0.48(0.81-0.26) | 1.499(1.035-1.965) |
| El Salvador | Female | 8.55(5.6-12.19) | 8(4.8-12.25) | -6.43 | 0.55(0.78-0.35) | 0.23(0.36-0.14) | -2.861(-3.511--2.206) |
| Equatorial Guinea | Female | 0.22(0.11-0.36) | 0.56(0.28-1.02) | 154.55 | 0.19(0.32-0.1) | 0.2(0.37-0.1) | 0.228(-0.082-0.539) |
| Eritrea | Female | 2.46(1.11-4.61) | 6.95(4.19-11.2) | 182.52 | 0.45(0.82-0.2) | 0.48(0.76-0.29) | 0.286(0.174-0.399) |
| Estonia | Female | 4.98(3.34-6.8) | 12.07(7.53-17.38) | 142.37 | 0.37(0.5-0.25) | 0.71(1-0.45) | 2.38(1.957-2.804) |
| Eswatini | Female | 0.82(0.46-1.32) | 1.82(0.9-3.48) | 121.95 | 0.5(0.81-0.28) | 0.53(1-0.27) | 0.2(0.056-0.344) |
| Ethiopia | Female | 27.09(15.66-44.55) | 43.55(31.41-59.91) | 60.76 | 0.28(0.45-0.16) | 0.22(0.3-0.16) | -0.794(-0.985--0.603) |
| Fiji | Female | 0.71(0.42-1.11) | 1.46(0.83-2.42) | 105.63 | 0.39(0.6-0.24) | 0.37(0.6-0.22) | -0.183(-0.444-0.079) |
| Finland | Female | 17.71(10.96-25.11) | 34.29(21.63-49.32) | 93.62 | 0.39(0.54-0.25) | 0.49(0.68-0.32) | 0.742(0.576-0.908) |
| France | Female | 150.28(100.29-208.19) | 342.78(222.72-493.78) | 128.09 | 0.3(0.42-0.21) | 0.44(0.63-0.29) | 1.327(1.114-1.541) |
| Gabon | Female | 0.91(0.5-1.46) | 1.24(0.63-2) | 36.26 | 0.3(0.47-0.16) | 0.22(0.35-0.11) | -0.948(-1.082--0.814) |
| Gambia | Female | 2.46(1.52-3.8) | 10.31(5.88-16.09) | 319.11 | 1.53(2.37-0.92) | 2.13(3.34-1.23) | 1.223(0.185-2.271) |
| Georgia | Female | 5.64(3.66-7.98) | 14.4(9.28-20.2) | 155.32 | 0.15(0.21-0.1) | 0.4(0.57-0.26) | 3.518(2.245-4.806) |
| Germany | Female | 268.8(189.65-365.7) | 487.34(331.7-682.83) | 81.30 | 0.33(0.45-0.24) | 0.46(0.64-0.32) | 1.17(0.839-1.501) |
| Ghana | Female | 8.77(5.26-13.78) | 19.55(11.74-30.25) | 122.92 | 0.28(0.43-0.16) | 0.22(0.35-0.14) | -0.717(-0.916--0.518) |
| Greece | Female | 34.17(23.09-46.91) | 56.09(36.88-79.7) | 64.15 | 0.39(0.54-0.27) | 0.39(0.54-0.27) | 0.022(-0.261-0.306) |
| Greenland | Female | 0.09(0.06-0.13) | 0.24(0.15-0.36) | 166.67 | 0.56(0.8-0.37) | 0.75(1.13-0.46) | 1.213(0.643-1.786) |
| Grenada | Female | 0.77(0.52-1.07) | 0.35(0.24-0.49) | -54.55 | 1.83(2.48-1.27) | 0.61(0.84-0.42) | -3.722(-4.121--3.322) |
| Guam | Female | 0.06(0.04-0.09) | 0.11(0.07-0.18) | 83.33 | 0.17(0.26-0.11) | 0.12(0.18-0.07) | -1.369(-2.465--0.262) |
| Guatemala | Female | 25.49(16.56-36) | 40(24.5-59.98) | 56.92 | 1.45(2.07-0.93) | 0.67(1.01-0.41) | -2.62(-3.444--1.789) |
| Guinea | Female | 24.24(14.78-37.67) | 38.18(22.3-59.95) | 57.51 | 1.47(2.3-0.89) | 1.44(2.28-0.84) | 0.011(-0.045-0.068) |
| Guinea-Bissau | Female | 1.1(0.63-1.82) | 1.95(1.1-3.25) | 77.27 | 0.53(0.87-0.31) | 0.51(0.86-0.28) | -0.188(-0.333--0.043) |
| Guyana | Female | 3.07(2.11-4.22) | 1.95(1.25-2.88) | -36.48 | 1.6(2.21-1.09) | 0.6(0.87-0.39) | -3.317(-3.901--2.729) |
| Haiti | Female | 23.06(11.6-41.37) | 28.32(13.66-51.31) | 22.81 | 1.43(2.57-0.7) | 0.81(1.44-0.39) | -1.972(-2.212--1.731) |
| Honduras | Female | 11.17(3.34-20.59) | 49.99(15.95-97.22) | 347.54 | 1.08(2.02-0.31) | 1.61(3.11-0.51) | 1.404(0.884-1.926) |
| Hungary | Female | 129.05(92.39-168.31) | 55.29(36.42-79.37) | -57.16 | 1.45(1.87-1.05) | 0.47(0.66-0.31) | -3.919(-4.672--3.16) |
| Iceland | Female | 0.27(0.17-0.4) | 0.71(0.44-1.04) | 162.96 | 0.17(0.25-0.11) | 0.24(0.35-0.15) | 1.157(0.929-1.386) |
| India | Female | 581.8(430.77-769.51) | 1887.77(1326.08-2581.81) | 224.47 | 0.29(0.39-0.22) | 0.33(0.45-0.23) | 0.454(0.178-0.732) |
| Indonesia | Female | 84.59(63.33-110.67) | 125.77(91.51-169.76) | 48.68 | 0.17(0.22-0.13) | 0.12(0.16-0.09) | -1.155(-1.32--0.989) |
| Iran (Islamic Republic of) | Female | 26.06(16.95-39.94) | 54.52(40.37-70.97) | 109.21 | 0.24(0.37-0.15) | 0.16(0.21-0.12) | -1.54(-2.242--0.832) |
| Iraq | Female | 7.5(4.31-11.75) | 26.58(15.35-43.37) | 254.40 | 0.19(0.3-0.11) | 0.23(0.37-0.13) | 0.57(0.208-0.934) |
| Ireland | Female | 4.98(3.25-7.16) | 18.66(11.96-27.03) | 274.70 | 0.21(0.3-0.14) | 0.46(0.66-0.3) | 2.697(2.111-3.287) |
| Israel | Female | 6.41(3.96-9.85) | 13.55(8.37-20.11) | 111.39 | 0.23(0.35-0.15) | 0.21(0.31-0.13) | -0.384(-0.684--0.083) |
| Italy | Female | 266.26(220.05-317.85) | 254.67(203.51-307.72) | -4.35 | 0.5(0.6-0.42) | 0.31(0.37-0.25) | -1.757(-2.034--1.48) |
| Jamaica | Female | 6.19(4.22-8.45) | 6.57(4.34-9.64) | 6.14 | 0.64(0.87-0.44) | 0.43(0.62-0.28) | -1.363(-2.101--0.619) |
| Japan | Female | 444.21(345.33-598.52) | 792.93(566.16-1185.57) | 78.50 | 0.46(0.62-0.35) | 0.34(0.49-0.25) | -0.999(-1.154--0.844) |
| Jordan | Female | 0.8(0.45-1.29) | 2.18(1.22-3.54) | 172.50 | 0.13(0.22-0.07) | 0.08(0.13-0.04) | -1.934(-2.331--1.535) |
| Kazakhstan | Female | 35.19(23.67-49.21) | 87.87(56-125.18) | 149.70 | 0.45(0.63-0.31) | 0.87(1.24-0.55) | 2.16(1.494-2.83) |
| Kenya | Female | 17.61(12.9-29.47) | 52.81(34.65-78.05) | 199.89 | 0.43(0.7-0.32) | 0.47(0.67-0.31) | 0.314(0.145-0.484) |
| Kiribati | Female | 0.17(0.1-0.26) | 0.3(0.16-0.48) | 76.47 | 0.81(1.25-0.5) | 0.75(1.15-0.41) | -0.278(-0.388--0.168) |
| Kuwait | Female | 0.18(0.11-0.27) | 0.58(0.33-0.89) | 222.22 | 0.09(0.14-0.05) | 0.07(0.1-0.04) | -1.102(-1.836--0.362) |
| Kyrgyzstan | Female | 2.91(1.86-4.13) | 8.86(5.77-12.83) | 204.47 | 0.16(0.23-0.1) | 0.36(0.53-0.23) | 2.915(2.462-3.371) |
| Lao People's Democratic Republic | Female | 8.92(4.29-16.85) | 12.64(7.48-19.34) | 41.70 | 0.82(1.5-0.4) | 0.58(0.88-0.35) | -1.174(-1.318--1.03) |
| Latvia | Female | 6.87(4.71-9.47) | 13.35(8.56-19.63) | 94.32 | 0.29(0.4-0.2) | 0.52(0.77-0.34) | 2.056(1.484-2.632) |
| Lebanon | Female | 2.59(1.53-4.15) | 4.47(2.37-7.63) | 72.59 | 0.23(0.38-0.14) | 0.16(0.26-0.08) | -1.415(-1.603--1.226) |
| Lesotho | Female | 2.52(1.39-4.35) | 4.97(2.46-9.05) | 97.22 | 0.46(0.79-0.25) | 0.67(1.2-0.34) | 1.336(1.106-1.566) |
| Liberia | Female | 2.69(1.63-4.18) | 4.11(2.38-6.47) | 52.79 | 0.52(0.81-0.32) | 0.43(0.7-0.25) | -0.699(-0.845--0.553) |
| Libya | Female | 2.23(1.28-3.59) | 5.33(2.98-8.75) | 139.01 | 0.27(0.43-0.15) | 0.23(0.38-0.12) | -0.51(-0.937--0.081) |
| Lithuania | Female | 8.14(5.58-10.95) | 19.46(12.8-27.26) | 139.07 | 0.29(0.38-0.2) | 0.53(0.75-0.35) | 2.121(1.574-2.671) |
| Luxembourg | Female | 1.53(1.01-2.09) | 2.98(1.86-4.31) | 94.77 | 0.46(0.63-0.31) | 0.55(0.79-0.34) | 0.574(0.382-0.767) |
| Madagascar | Female | 10.25(6.43-15.74) | 20.05(11.87-32.25) | 95.61 | 0.41(0.63-0.26) | 0.37(0.58-0.22) | -0.392(-0.595--0.188) |
| Malawi | Female | 7.96(4.87-12) | 11.39(6.75-17.11) | 43.09 | 0.39(0.6-0.24) | 0.3(0.44-0.18) | -0.956(-1.294--0.616) |
| Malaysia | Female | 14.15(8.9-21.35) | 39.89(22.8-65.07) | 181.91 | 0.32(0.48-0.19) | 0.31(0.49-0.18) | -0.1(-0.264-0.065) |
| Maldives | Female | 0.1(0.05-0.21) | 0.26(0.16-0.4) | 160.00 | 0.31(0.63-0.15) | 0.21(0.33-0.13) | -1.29(-1.889--0.688) |
| Mali | Female | 9.2(5.5-14.09) | 19.29(10.96-31.05) | 109.67 | 0.43(0.67-0.26) | 0.46(0.74-0.26) | 0.218(0.096-0.339) |
| Malta | Female | 0.44(0.28-0.65) | 1.21(0.76-1.81) | 175.00 | 0.18(0.27-0.12) | 0.24(0.35-0.15) | 0.919(0.622-1.218) |
| Marshall Islands | Female | 0.05(0.03-0.09) | 0.09(0.05-0.18) | 80.00 | 0.63(1.09-0.35) | 0.55(1-0.29) | -0.44(-0.515--0.365) |
| Mauritania | Female | 2.31(1.39-3.71) | 3.14(1.84-5.03) | 35.93 | 0.44(0.71-0.27) | 0.31(0.51-0.18) | -1.191(-1.316--1.065) |
| Mauritius | Female | 0.72(0.47-1.02) | 1.55(0.97-2.43) | 115.28 | 0.18(0.26-0.12) | 0.16(0.25-0.1) | -0.218(-0.591-0.156) |
| Mexico | Female | 89.98(75.88-105.47) | 390.23(298.56-493.21) | 333.69 | 0.42(0.5-0.36) | 0.63(0.8-0.48) | 1.376(1.006-1.747) |
| Micronesia (Federated States of) | Female | 0.16(0.08-0.27) | 0.19(0.09-0.36) | 18.75 | 0.68(1.17-0.37) | 0.53(0.95-0.27) | -0.839(-1.033--0.645) |
| Monaco | Female | 0.12(0.07-0.18) | 0.35(0.21-0.56) | 191.67 | 0.27(0.42-0.16) | 0.67(1.04-0.4) | 3.135(2.901-3.369) |
| Mongolia | Female | 36.04(21.24-57.13) | 206.3(123.56-314.51) | 472.42 | 6.42(10.09-3.79) | 18.44(27.71-11.04) | 3.73(3.284-4.177) |
| Montenegro | Female | 4.58(3-6.53) | 5.93(3.77-8.83) | 29.48 | 1.32(1.9-0.86) | 1.08(1.59-0.7) | -0.698(-1.008--0.388) |
| Morocco | Female | 4.61(2.57-7.57) | 10.16(5.53-17.02) | 120.39 | 0.07(0.12-0.04) | 0.07(0.11-0.04) | -0.167(-0.308--0.026) |
| Mozambique | Female | 8.47(5.27-12.67) | 19.81(11.55-31.6) | 133.88 | 0.28(0.42-0.18) | 0.34(0.54-0.2) | 0.649(0.44-0.859) |
| Myanmar | Female | 23.78(12.76-42.21) | 89.9(54.01-138.27) | 278.05 | 0.2(0.36-0.11) | 0.35(0.54-0.21) | 2.126(1.416-2.84) |
| Namibia | Female | 0.79(0.45-1.26) | 2.49(1.44-3.89) | 215.19 | 0.2(0.32-0.12) | 0.31(0.49-0.18) | 1.598(1.402-1.794) |
| Nauru | Female | 0.01(0.01-0.02) | 0.01(0.01-0.02) | 0.00 | 0.64(1.02-0.38) | 0.5(0.91-0.27) | -0.857(-1.043--0.67) |
| Nepal | Female | 8.85(4.97-15.29) | 25.79(14.93-39.52) | 191.41 | 0.2(0.35-0.12) | 0.23(0.35-0.13) | 0.439(0.388-0.489) |
| Netherlands | Female | 26.63(17.71-36.74) | 79.25(51.43-110.35) | 197.60 | 0.22(0.31-0.15) | 0.43(0.59-0.28) | 2.211(1.953-2.47) |
| New Zealand | Female | 6.09(5.04-7.29) | 17.81(14.39-21.27) | 192.45 | 0.28(0.34-0.23) | 0.44(0.52-0.35) | 1.409(0.809-2.013) |
| Nicaragua | Female | 3.27(2.17-4.57) | 14(8.81-20.64) | 328.13 | 0.41(0.58-0.27) | 0.6(0.88-0.38) | 1.485(0.954-2.019) |
| Niger | Female | 0.46(0.27-0.72) | 1.43(0.81-2.31) | 210.87 | 0.04(0.06-0.02) | 0.04(0.06-0.02) | 0.042(-0.077-0.162) |
| Nigeria | Female | 88.13(60.06-123.2) | 191.97(129.07-267.1) | 117.83 | 0.42(0.59-0.29) | 0.45(0.62-0.31) | 0.2(0.091-0.308) |
| Niue | Female | 0.01(0-0.01) | 0(0-0.01) | -100.00 | 0.5(0.78-0.29) | 0.36(0.59-0.2) | -1.141(-1.252--1.029) |
| North Macedonia | Female | 15.46(10.46-21.11) | 23.37(14.37-35.03) | 51.16 | 1.62(2.22-1.1) | 1.36(2.01-0.85) | -0.659(-0.881--0.437) |
| Northern Mariana Islands | Female | 0.03(0.02-0.05) | 0.06(0.04-0.1) | 100.00 | 0.47(0.73-0.28) | 0.24(0.35-0.14) | -2.48(-2.898--2.061) |
| Norway | Female | 6.63(5.55-7.85) | 14.86(12.02-17.67) | 124.13 | 0.17(0.2-0.14) | 0.3(0.35-0.24) | 1.973(1.835-2.111) |
| Oman | Female | 0.35(0.19-0.61) | 0.95(0.54-1.48) | 171.43 | 0.12(0.21-0.07) | 0.14(0.23-0.08) | 0.437(0.135-0.74) |
| Pakistan | Female | 118.04(71.66-178.63) | 218.16(150-303.35) | 84.82 | 0.47(0.71-0.28) | 0.41(0.58-0.28) | -0.377(-0.486--0.267) |
| Palau | Female | 0.01(0-0.01) | 0.01(0.01-0.02) | 0.00 | 0.14(0.22-0.08) | 0.12(0.2-0.07) | -0.48(-0.554--0.406) |
| Palestine | Female | 1.69(0.92-2.89) | 2.68(1.61-4.2) | 58.58 | 0.37(0.62-0.2) | 0.24(0.38-0.14) | -1.59(-2.019--1.16) |
| Panama | Female | 4.05(2.65-5.78) | 10.41(6.43-15.12) | 157.04 | 0.55(0.79-0.36) | 0.49(0.72-0.3) | -0.372(-0.906-0.166) |
| Papua New Guinea | Female | 0.53(0.3-0.86) | 1.26(0.72-2.04) | 137.74 | 0.07(0.11-0.04) | 0.07(0.11-0.04) | -0.004(-0.12-0.111) |
| Paraguay | Female | 5.17(3.37-7.34) | 7.24(4.41-11.11) | 40.04 | 0.45(0.64-0.29) | 0.26(0.39-0.15) | -1.897(-2.277--1.515) |
| Peru | Female | 96.31(65.25-136.01) | 123.72(77.16-187.07) | 28.46 | 1.66(2.34-1.13) | 0.75(1.14-0.46) | -2.695(-3.187--2.202) |
| Philippines | Female | 97.43(73.75-125.27) | 199.46(145.24-267.48) | 104.72 | 0.67(0.86-0.52) | 0.49(0.65-0.36) | -1.072(-1.264--0.879) |
| Poland | Female | 452.35(388-522.44) | 155.02(117.26-201.55) | -65.73 | 1.72(1.98-1.48) | 0.37(0.48-0.27) | -5.125(-6.215--4.022) |
| Portugal | Female | 19.56(13.19-27.54) | 49.79(32.23-71) | 154.55 | 0.24(0.33-0.16) | 0.36(0.5-0.24) | 1.414(1.085-1.744) |
| Puerto Rico | Female | 24.65(16.61-33.92) | 16.16(10.21-23.65) | -34.44 | 1.23(1.67-0.83) | 0.39(0.57-0.25) | -3.919(-4.711--3.12) |
| Qatar | Female | 0.2(0.11-0.32) | 0.93(0.5-1.58) | 365.00 | 0.71(1.21-0.38) | 0.68(1.11-0.37) | -0.068(-0.389-0.254) |
| Republic of Korea | Female | 106.15(64.33-166.08) | 521.56(345.1-754.76) | 391.34 | 0.63(0.99-0.39) | 1.05(1.51-0.69) | 1.78(1.072-2.492) |
| Republic of Moldova | Female | 13.2(10.01-16.53) | 18.85(13.17-25.03) | 42.80 | 0.5(0.62-0.38) | 0.54(0.71-0.38) | 0.072(-1.449-1.618) |
| Romania | Female | 50.61(34.42-69.32) | 114.5(75.84-162.46) | 126.24 | 0.33(0.44-0.22) | 0.53(0.74-0.36) | 1.722(1.457-1.988) |
| Russian Federation | Female | 334.22(279.84-389.13) | 726.93(565.81-922.91) | 117.50 | 0.28(0.33-0.24) | 0.49(0.62-0.38) | 1.905(1.444-2.367) |
| Rwanda | Female | 14.68(8.45-24.84) | 24.9(15.97-37.76) | 69.62 | 0.93(1.53-0.54) | 0.74(1.13-0.46) | -0.78(-1.097--0.463) |
| Saint Kitts and Nevis | Female | 0.51(0.33-0.7) | 0.17(0.11-0.25) | -66.67 | 2.3(3.1-1.54) | 0.54(0.78-0.36) | -4.82(-5.448--4.187) |
| Saint Lucia | Female | 0.66(0.46-0.89) | 0.41(0.28-0.57) | -37.88 | 1.34(1.8-0.95) | 0.36(0.5-0.25) | -4.424(-5.448--3.389) |
| Saint Vincent and the Grenadines | Female | 0.48(0.32-0.66) | 0.32(0.21-0.45) | -33.33 | 1.17(1.6-0.78) | 0.48(0.68-0.33) | -2.974(-3.289--2.659) |
| Samoa | Female | 0.11(0.06-0.17) | 0.17(0.1-0.27) | 54.55 | 0.24(0.38-0.15) | 0.23(0.37-0.13) | -0.182(-0.267--0.097) |
| San Marino | Female | 0.04(0.02-0.06) | 0.1(0.05-0.17) | 150.00 | 0.2(0.31-0.13) | 0.28(0.49-0.15) | 1.144(0.928-1.361) |
| Sao Tome and Principe | Female | 0.07(0.04-0.1) | 0.12(0.07-0.2) | 71.43 | 0.2(0.29-0.11) | 0.23(0.39-0.13) | 0.734(0.571-0.896) |
| Saudi Arabia | Female | 2.44(1.38-4) | 6.01(3.27-10.05) | 146.31 | 0.11(0.18-0.06) | 0.1(0.16-0.05) | -0.32(-0.566--0.073) |
| Senegal | Female | 4.2(2.61-6.4) | 9.18(5.27-14.18) | 118.57 | 0.28(0.43-0.17) | 0.25(0.39-0.14) | -0.308(-0.91-0.297) |
| Serbia | Female | 65.18(41.87-94.8) | 78.21(47.04-115.16) | 19.99 | 1.1(1.57-0.72) | 0.87(1.25-0.54) | -0.807(-1.182--0.431) |
| Seychelles | Female | 0.17(0.11-0.26) | 0.22(0.14-0.33) | 29.41 | 0.54(0.8-0.34) | 0.4(0.59-0.25) | -1.057(-1.402--0.71) |
| Sierra Leone | Female | 4.16(2.44-6.52) | 7.42(4.3-11.9) | 78.37 | 0.45(0.71-0.27) | 0.43(0.68-0.25) | -0.195(-0.466-0.077) |
| Singapore | Female | 2.12(1.34-3.15) | 9.43(5.58-14.61) | 344.81 | 0.19(0.28-0.12) | 0.24(0.37-0.14) | 0.884(0.345-1.426) |
| Slovakia | Female | 32.5(22.86-43.28) | 34.83(21.7-51.67) | 7.17 | 0.93(1.22-0.66) | 0.64(0.95-0.4) | -1.368(-1.682--1.054) |
| Slovenia | Female | 8.08(5.05-11.92) | 15.12(9.15-22.96) | 87.13 | 0.53(0.79-0.34) | 0.61(0.91-0.37) | 0.446(-0.068-0.964) |
| Solomon Islands | Female | 0.34(0.19-0.57) | 0.65(0.37-1) | 91.18 | 0.49(0.81-0.28) | 0.38(0.59-0.23) | -0.918(-1.189--0.647) |
| Somalia | Female | 5.46(2.61-9.79) | 14.76(8.04-26.44) | 170.33 | 0.42(0.76-0.21) | 0.41(0.73-0.22) | -0.119(-0.215--0.023) |
| South Africa | Female | 73.91(50.46-100.7) | 106.59(81.49-144.05) | 44.22 | 0.62(0.85-0.42) | 0.42(0.56-0.32) | -1.263(-1.685--0.839) |
| South Sudan | Female | 3.87(2.33-5.88) | 5.68(3.08-9.29) | 46.77 | 0.37(0.57-0.23) | 0.33(0.54-0.18) | -0.43(-0.588--0.273) |
| Spain | Female | 126.62(81.63-182.16) | 211.23(130.8-316.53) | 66.82 | 0.39(0.55-0.26) | 0.38(0.56-0.24) | -0.084(-0.289-0.121) |
| Sri Lanka | Female | 8.33(5.1-12.87) | 31.8(18.4-51.37) | 281.75 | 0.16(0.25-0.1) | 0.22(0.36-0.13) | 1.15(0.509-1.796) |
| Sudan | Female | 7.47(3.6-12.76) | 12.8(6.93-20.55) | 71.35 | 0.17(0.3-0.08) | 0.16(0.26-0.09) | -0.237(-0.312--0.161) |
| Suriname | Female | 1.65(1.11-2.28) | 1.41(0.9-2.02) | -14.55 | 1.26(1.75-0.84) | 0.44(0.64-0.28) | -3.561(-3.994--3.127) |
| Sweden | Female | 34.52(27.93-42.31) | 39.55(31.72-48.61) | 14.57 | 0.41(0.5-0.33) | 0.36(0.44-0.29) | -0.47(-0.747--0.192) |
| Switzerland | Female | 15.35(10.13-21.25) | 44.86(29.41-63.81) | 192.25 | 0.25(0.34-0.17) | 0.48(0.68-0.32) | 2.182(1.783-2.583) |
| Syrian Arab Republic | Female | 5.25(3.06-8.33) | 10.85(6.03-17.54) | 106.67 | 0.23(0.38-0.13) | 0.2(0.32-0.11) | -0.453(-0.845--0.059) |
| Taiwan (Province of China) | Female | 26.05(16.88-38.1) | 55.53(32.46-87.3) | 113.17 | 0.34(0.49-0.22) | 0.26(0.41-0.15) | -1.186(-2.12--0.243) |
| Tajikistan | Female | 1.37(0.87-2) | 8.7(5.3-13.13) | 535.04 | 0.09(0.13-0.06) | 0.38(0.58-0.23) | 5.188(4.757-5.622) |
| Thailand | Female | 352.12(222.57-527.58) | 1164.94(706.49-1771.6) | 230.84 | 1.92(2.89-1.24) | 2.09(3.19-1.24) | 0.256(-0.051-0.565) |
| Timor-Leste | Female | 0.75(0.4-1.3) | 1.87(1.1-3) | 149.33 | 0.57(0.98-0.3) | 0.46(0.73-0.27) | -0.736(-0.826--0.646) |
| Togo | Female | 3.18(1.96-4.85) | 8.42(4.96-12.81) | 164.78 | 0.5(0.78-0.31) | 0.42(0.65-0.25) | -0.641(-0.812--0.47) |
| Tokelau | Female | 0(0-0.01) | 0(0-0.01) | #DIV/0! | 0.61(1.01-0.35) | 0.47(0.75-0.27) | -0.896(-0.933--0.859) |
| Tonga | Female | 0.25(0.14-0.39) | 0.33(0.2-0.52) | 32.00 | 0.85(1.32-0.51) | 0.79(1.23-0.47) | -0.272(-0.395--0.149) |
| Trinidad and Tobago | Female | 6.14(4.27-8.4) | 4.97(3.04-7.32) | -19.06 | 1.4(1.9-0.97) | 0.51(0.75-0.32) | -3.356(-3.877--2.831) |
| Tunisia | Female | 1.58(0.78-2.78) | 3.83(1.79-7.04) | 142.41 | 0.07(0.12-0.03) | 0.06(0.11-0.03) | -0.399(-0.65--0.148) |
| Turkey | Female | 38.01(21.45-64.21) | 65.87(38.77-99.34) | 73.30 | 0.21(0.36-0.12) | 0.14(0.22-0.08) | -1.374(-1.76--0.987) |
| Turkmenistan | Female | 1.33(0.86-1.96) | 15.37(9.61-23.27) | 1055.64 | 0.12(0.18-0.08) | 0.7(1.04-0.44) | 6.089(5.407-6.774) |
| Tuvalu | Female | 0.02(0.01-0.04) | 0.02(0.01-0.04) | 0.00 | 0.61(1.05-0.34) | 0.45(0.74-0.25) | -1.096(-1.233--0.959) |
| Uganda | Female | 17.45(10.67-25.79) | 77.92(47.73-115.34) | 346.53 | 0.54(0.8-0.33) | 1.02(1.52-0.62) | 2.228(2.053-2.404) |
| Ukraine | Female | 77.02(64.18-90.72) | 174.98(133.09-227.13) | 127.19 | 0.16(0.19-0.14) | 0.36(0.47-0.27) | 2.684(2.168-3.204) |
| United Arab Emirates | Female | 0.24(0.11-0.42) | 1.06(0.41-1.9) | 341.67 | 0.18(0.35-0.09) | 0.11(0.21-0.04) | -1.746(-3.176--0.296) |
| United Kingdom | Female | 134.89(113.9-157.74) | 382.2(319.41-450.5) | 183.34 | 0.25(0.29-0.22) | 0.55(0.65-0.47) | 2.681(2.433-2.929) |
| United Republic of Tanzania | Female | 16.54(10.66-24.14) | 39.98(25.37-57.15) | 141.72 | 0.3(0.44-0.2) | 0.32(0.46-0.2) | 0.205(0.03-0.38) |
| United States of America | Female | 333.31(279.95-388.23) | 981.93(806.56-1171.21) | 194.60 | 0.18(0.21-0.15) | 0.32(0.39-0.27) | 2.08(1.913-2.248) |
| United States Virgin Islands | Female | 0.35(0.24-0.5) | 0.39(0.25-0.57) | 11.43 | 0.8(1.12-0.53) | 0.36(0.51-0.23) | -2.724(-3.042--2.406) |
| Uruguay | Female | 3.1(1.96-4.4) | 6.46(4.16-9.52) | 108.39 | 0.14(0.2-0.09) | 0.2(0.29-0.13) | 1.344(1.252-1.436) |
| Uzbekistan | Female | 6.91(4.45-9.91) | 100.1(62.74-147.59) | 1348.63 | 0.11(0.16-0.07) | 0.92(1.33-0.59) | 7.706(7.444-7.969) |
| Vanuatu | Female | 0.14(0.07-0.25) | 0.36(0.19-0.63) | 157.14 | 0.49(0.88-0.26) | 0.44(0.78-0.23) | -0.423(-0.747--0.098) |
| Venezuela (Bolivarian Republic of) | Female | 65.38(43.18-90.27) | 46.62(28.2-73.08) | -28.69 | 1.31(1.82-0.86) | 0.3(0.47-0.18) | -5.037(-5.759--4.309) |
| Viet Nam | Female | 65.65(37.92-104.45) | 123.21(71.57-193.38) | 87.68 | 0.29(0.45-0.16) | 0.25(0.39-0.14) | -0.482(-0.66--0.303) |
| Yemen | Female | 1.77(0.81-3.31) | 4.77(2.54-8.18) | 169.49 | 0.07(0.13-0.03) | 0.07(0.13-0.04) | 0.087(0.014-0.161) |
| Zambia | Female | 4.33(2.6-6.78) | 10.54(6.37-16.43) | 143.42 | 0.33(0.5-0.2) | 0.31(0.48-0.19) | -0.161(-0.307--0.015) |
| Zimbabwe | Female | 18.5(10.83-28.15) | 47.64(25.95-76.67) | 157.51 | 0.89(1.37-0.52) | 1.18(1.91-0.65) | 1.031(0.505-1.56) |
| Afghanistan | Male | 52.65(29.9-84.63) | 69.77(42.05-106.34) | 32.52 | 1.43(2.26-0.84) | 1.31(1.99-0.77) | -0.315(-0.425--0.206) |
| Albania | Male | 55.27(40.06-71.19) | 96.53(60.28-141.73) | 74.65 | 5.97(7.64-4.3) | 4.62(6.74-2.94) | -0.941(-1.386--0.494) |
| Algeria | Male | 15.68(9.34-24.45) | 60.47(35.28-94.79) | 285.65 | 0.27(0.42-0.16) | 0.38(0.58-0.22) | 1.054(0.757-1.353) |
| American Samoa | Male | 0.11(0.06-0.17) | 0.29(0.18-0.45) | 163.64 | 0.92(1.44-0.52) | 1.27(1.92-0.8) | 1.097(0.733-1.461) |
| Andorra | Male | 1.85(1.25-2.73) | 4.29(2.88-5.87) | 131.89 | 6.5(9.34-4.44) | 6.16(8.51-4.15) | -0.138(-0.164--0.111) |
| Angola | Male | 10.74(6.1-17.06) | 35.73(22.95-52.67) | 232.68 | 0.62(0.98-0.36) | 0.79(1.15-0.5) | 0.896(0.745-1.047) |
| Antigua and Barbuda | Male | 1.08(0.78-1.41) | 0.73(0.52-0.99) | -32.41 | 4.65(6.07-3.36) | 1.58(2.14-1.13) | -3.737(-4.63--2.836) |
| Argentina | Male | 127.41(89.16-167.26) | 318.73(229.76-409.26) | 150.16 | 0.9(1.18-0.63) | 1.35(1.73-0.98) | 1.409(1.063-1.755) |
| Armenia | Male | 4.98(3.52-6.66) | 62.87(44.21-84.93) | 1162.45 | 0.48(0.64-0.34) | 3.64(4.93-2.59) | 7.244(6.063-8.438) |
| Australia | Male | 138.11(107.13-165.56) | 543.32(418.19-675.3) | 293.40 | 1.54(1.84-1.2) | 2.82(3.49-2.16) | 2.134(1.973-2.295) |
| Austria | Male | 166.57(141.7-190.1) | 357.62(290.58-429.38) | 114.70 | 3.66(4.17-3.11) | 4.51(5.41-3.68) | 0.717(0.395-1.041) |
| Azerbaijan | Male | 7.98(5.49-10.84) | 76.1(45.22-119.83) | 853.63 | 0.45(0.6-0.31) | 1.99(3.19-1.17) | 5.306(4.802-5.813) |
| Bahamas | Male | 3.66(2.69-4.79) | 3.16(2.18-4.34) | -13.66 | 5.51(7.15-4.07) | 1.83(2.49-1.27) | -3.827(-4.19--3.463) |
| Bahrain | Male | 0.86(0.53-1.31) | 3.5(2.05-5.65) | 306.98 | 1.12(1.7-0.7) | 0.77(1.22-0.46) | -1.4(-2.089--0.707) |
| Bangladesh | Male | 298.23(197.85-422.57) | 644.35(403.67-951.82) | 116.06 | 1.15(1.63-0.76) | 0.96(1.41-0.6) | -0.545(-0.684--0.405) |
| Barbados | Male | 2(1.14-3.17) | 3.43(2.4-4.56) | 71.50 | 1.59(2.51-0.9) | 1.52(2.02-1.07) | -0.216(-0.716-0.287) |
| Belarus | Male | 40.15(30.68-48.87) | 115.11(68.12-178.55) | 186.70 | 0.87(1.06-0.68) | 1.91(2.92-1.15) | 2.766(1.979-3.558) |
| Belgium | Male | 123.01(94.64-148.55) | 269.31(204.41-336.28) | 118.93 | 1.9(2.3-1.48) | 2.66(3.31-2.02) | 1.2(0.7-1.703) |
| Belize | Male | 1.13(0.83-1.49) | 2.22(1.6-2.94) | 96.46 | 2.49(3.26-1.79) | 1.67(2.21-1.19) | -1.333(-1.939--0.724) |
| Benin | Male | 19.34(12.37-30.4) | 35.29(21.53-57.24) | 82.47 | 2.06(3.25-1.31) | 1.69(2.74-1.03) | -0.676(-0.782--0.571) |
| Bermuda | Male | 1.26(0.96-1.61) | 0.82(0.59-1.08) | -34.92 | 4.73(5.99-3.61) | 1.4(1.84-1.01) | -4.218(-4.766--3.667) |
| Bhutan | Male | 1.43(0.8-2.46) | 4.51(2.56-7.2) | 215.38 | 1.21(2.04-0.7) | 1.61(2.61-0.91) | 0.973(0.797-1.15) |
| Bolivia (Plurinational State of) | Male | 23.47(14.53-34.82) | 73.15(45.03-111.26) | 211.67 | 1.66(2.45-1.04) | 1.84(2.79-1.16) | 0.361(0.245-0.477) |
| Bosnia and Herzegovina | Male | 62.42(45.13-80.7) | 121.74(84.09-166.66) | 95.03 | 3.64(4.72-2.69) | 4.52(6.2-3.14) | 0.737(0.34-1.135) |
| Botswana | Male | 0.74(0.22-2.67) | 3.7(2.24-5.78) | 400.00 | 0.29(1.07-0.09) | 0.61(0.93-0.39) | 2.674(2.306-3.045) |
| Brazil | Male | 345.7(300.94-391.88) | 1352.74(1167.82-1533.55) | 291.30 | 0.85(0.96-0.74) | 1.29(1.46-1.11) | 1.442(1.152-1.733) |
| Brunei Darussalam | Male | 0.72(0.44-1.12) | 2.53(1.53-4.04) | 251.39 | 1.61(2.51-0.99) | 1.92(2.87-1.19) | 0.723(-0.068-1.52) |
| Bulgaria | Male | 245.4(190.16-300.34) | 226.94(166.35-302.3) | -7.52 | 4.14(5.07-3.26) | 3.7(4.92-2.7) | -0.333(-0.901-0.238) |
| Burkina Faso | Male | 14.65(8.86-21.64) | 23.25(14.22-34.54) | 58.70 | 0.76(1.11-0.47) | 0.63(0.94-0.38) | -0.649(-0.791--0.507) |
| Burundi | Male | 21(13.06-32.56) | 28.61(14.79-57.34) | 36.24 | 2.03(3.14-1.28) | 1.3(2.51-0.69) | -1.538(-1.702--1.374) |
| Cabo Verde | Male | 0.33(0.22-0.48) | 7.64(5.26-10.46) | 2215.15 | 0.33(0.47-0.22) | 4.34(5.91-2.97) | 9.523(8.017-11.049) |
| Cambodia | Male | 38.17(23.98-59.4) | 136.32(86.87-194.85) | 257.14 | 2.1(3.27-1.31) | 2.93(4.1-1.89) | 1.168(1.008-1.328) |
| Cameroon | Male | 4.31(2.32-7.88) | 13.69(8.32-21.43) | 217.63 | 0.21(0.39-0.11) | 0.25(0.39-0.16) | 0.642(0.451-0.833) |
| Canada | Male | 281.91(247.82-312.92) | 1206.35(981.76-1428.1) | 327.92 | 1.95(2.17-1.71) | 3.8(4.49-3.09) | 2.316(2.055-2.577) |
| Central African Republic | Male | 5.62(3.02-8.9) | 8.73(4.55-15.7) | 55.34 | 1.09(1.71-0.61) | 1.01(1.78-0.55) | -0.27(-0.503--0.036) |
| Chad | Male | 21.37(11.51-34.81) | 38.52(22.3-60.05) | 80.25 | 1.57(2.56-0.86) | 1.36(2.13-0.79) | -0.49(-0.6--0.38) |
| Chile | Male | 52.81(39.06-67.62) | 174.56(127.15-223.49) | 230.54 | 1.19(1.52-0.87) | 1.61(2.06-1.17) | 1.05(0.518-1.585) |
| China | Male | 17542.28(10505.91-27677.13) | 17093.49(11152.92-25641.76) | -2.56 | 3.85(5.91-2.42) | 1.74(2.58-1.14) | -2.638(-3.076--2.199) |
| Colombia | Male | 94.61(71.21-117.52) | 328.5(218.09-478.82) | 247.21 | 1.15(1.43-0.87) | 1.39(2.03-0.92) | 0.633(-0.709-1.993) |
| Comoros | Male | 1.1(0.46-2.61) | 2.02(1-4.42) | 83.64 | 1.04(2.44-0.46) | 0.96(2.11-0.48) | -0.302(-0.853-0.253) |
| Congo | Male | 5.3(2.85-8.64) | 9.79(5.65-15.42) | 84.72 | 1.17(1.88-0.66) | 0.86(1.35-0.52) | -1.051(-1.156--0.945) |
| Cook Islands | Male | 0.16(0.09-0.24) | 0.39(0.25-0.57) | 143.75 | 2.36(3.6-1.38) | 3.26(4.64-2.1) | 1.13(0.928-1.333) |
| Costa Rica | Male | 24.46(18.52-30.61) | 71.55(48.93-101.59) | 192.52 | 2.94(3.69-2.22) | 3.06(4.3-2.08) | 0.089(-0.751-0.937) |
| Croatia | Male | 59.94(46.73-72.64) | 114.67(80.64-154.96) | 91.31 | 2.35(2.83-1.87) | 3.02(4.04-2.14) | 0.915(0.311-1.523) |
| Cuba | Male | 156.27(116.75-196.26) | 138.67(94.76-189.06) | -11.26 | 3.06(3.84-2.29) | 1.55(2.11-1.06) | -2.211(-3.181--1.231) |
| Cyprus | Male | 8.17(5.62-11.21) | 20.09(14.72-26.29) | 145.90 | 2.08(2.82-1.47) | 2.16(2.79-1.6) | 0.125(-0.259-0.51) |
| Czechia | Male | 212.63(173.79-248.57) | 238.91(176.8-309.17) | 12.36 | 3.73(4.35-3.05) | 2.54(3.28-1.89) | -1.245(-2.095--0.388) |
| C么te d'Ivoire | Male | 51.2(30.05-79.25) | 87.91(53.36-138.74) | 71.70 | 2.54(3.91-1.51) | 1.76(2.74-1.09) | -1.272(-1.562--0.981) |
| Democratic People's Republic of Korea | Male | 181.01(96.91-313.08) | 252.88(145.33-412.12) | 39.70 | 2.51(4.16-1.39) | 1.78(2.89-1.05) | -1.182(-1.299--1.063) |
| Democratic Republic of the Congo | Male | 38.86(24.53-58.53) | 73.11(42.48-114.26) | 88.14 | 0.56(0.81-0.36) | 0.48(0.72-0.29) | -0.519(-0.746--0.292) |
| Denmark | Male | 49.94(39.32-60.55) | 122.09(91.59-153.35) | 144.47 | 1.41(1.7-1.11) | 2.25(2.81-1.68) | 1.685(1.41-1.96) |
| Djibouti | Male | 0.77(0.32-1.96) | 3.66(1.78-7.6) | 375.32 | 1.16(2.86-0.51) | 1.23(2.57-0.63) | 0.231(-0.002-0.465) |
| Dominica | Male | 1.47(1.06-1.94) | 0.84(0.57-1.17) | -42.86 | 4.87(6.4-3.52) | 1.9(2.64-1.3) | -3.17(-3.606--2.732) |
| Dominican Republic | Male | 34.95(25.05-46.3) | 119.44(68.51-214.88) | 241.75 | 1.92(2.55-1.38) | 2.72(4.82-1.57) | 1.201(0.872-1.532) |
| Ecuador | Male | 21.73(15.3-29.83) | 93.75(61.89-137) | 331.43 | 0.86(1.19-0.6) | 1.36(1.97-0.9) | 1.539(1.244-1.835) |
| Egypt | Male | 338.78(197.92-534.88) | 1064.84(579.13-1877.32) | 214.32 | 2.24(3.48-1.3) | 2.92(5.03-1.6) | 0.982(0.756-1.208) |
| El Salvador | Male | 17.96(13.02-22.91) | 31.32(20.23-44.78) | 74.39 | 1.32(1.7-0.96) | 1.25(1.79-0.81) | -0.286(-0.793-0.224) |
| Equatorial Guinea | Male | 0.64(0.36-1.03) | 2.25(1.09-3.86) | 251.56 | 0.77(1.22-0.44) | 1.24(2.08-0.6) | 1.729(1.361-2.1) |
| Eritrea | Male | 4.88(2.04-10.59) | 11.84(5.41-23.84) | 142.62 | 1.27(2.76-0.52) | 1.15(2.24-0.54) | -0.289(-0.659-0.082) |
| Estonia | Male | 10.52(7.97-13.04) | 29.56(21.01-39.56) | 180.99 | 1.41(1.73-1.08) | 3(4.02-2.14) | 2.725(1.644-3.818) |
| Eswatini | Male | 2.42(1.09-6.75) | 25.76(5.41-54) | 964.46 | 1.94(5.38-0.89) | 11.33(22.94-2.44) | 6.381(5.859-6.906) |
| Ethiopia | Male | 107.09(69.78-154.71) | 220.17(159.47-307.04) | 105.59 | 1.08(1.51-0.71) | 1.12(1.56-0.82) | 0.171(-0.017-0.359) |
| Fiji | Male | 1.87(1.15-2.94) | 4.93(2.9-7.63) | 163.64 | 1.1(1.69-0.67) | 1.43(2.21-0.87) | 0.954(0.692-1.217) |
| Finland | Male | 57.02(43.91-69.54) | 158.49(117.57-199.34) | 177.96 | 2.02(2.46-1.56) | 2.81(3.51-2.11) | 1.136(0.937-1.336) |
| France | Male | 1361.55(1004.89-1726.08) | 2226.52(1593.04-2973.45) | 63.53 | 3.93(4.95-2.91) | 3.79(5.05-2.72) | -0.176(-0.411-0.06) |
| Gabon | Male | 2.52(1.27-4) | 5.3(2.72-9.82) | 110.32 | 1.04(1.61-0.52) | 1.18(2.17-0.62) | 0.45(0.261-0.64) |
| Gambia | Male | 17.16(10.72-26.53) | 64.6(37.1-98.77) | 276.46 | 8.95(13.35-5.75) | 13.69(20.5-8.14) | 1.495(0.421-2.579) |
| Georgia | Male | 13.02(8.78-17.4) | 56.12(39.25-75.8) | 331.03 | 0.58(0.77-0.4) | 2.36(3.18-1.65) | 5.015(4.13-5.909) |
| Germany | Male | 1064.05(871.25-1266.52) | 3070.04(2517.65-3616.86) | 188.52 | 2.21(2.62-1.81) | 3.56(4.2-2.93) | 1.677(1.467-1.887) |
| Ghana | Male | 65.19(40.16-103.43) | 192.62(121.31-282.18) | 195.47 | 2.31(3.6-1.46) | 2.9(4.21-1.89) | 0.824(0.629-1.02) |
| Greece | Male | 85.88(63.84-109.23) | 210.4(156.05-270.41) | 144.99 | 1.22(1.54-0.91) | 1.93(2.47-1.45) | 1.602(1.282-1.923) |
| Greenland | Male | 0.61(0.45-0.79) | 1.69(1.15-2.35) | 177.05 | 3.24(4.12-2.45) | 4.38(5.99-3.04) | 1.022(0.806-1.239) |
| Grenada | Male | 1.51(1.1-1.93) | 0.97(0.72-1.25) | -35.76 | 4.97(6.4-3.61) | 1.9(2.43-1.43) | -3.22(-3.941--2.492) |
| Guam | Male | 0.29(0.18-0.44) | 1.34(0.84-2.01) | 362.07 | 0.77(1.14-0.49) | 1.41(2.1-0.91) | 2.127(1.706-2.55) |
| Guatemala | Male | 59.68(42.15-80.37) | 90.05(60.11-128.98) | 50.89 | 3.45(4.61-2.4) | 1.86(2.66-1.25) | -2.138(-2.914--1.355) |
| Guinea | Male | 143.86(92.14-212.75) | 288.78(164.08-437.24) | 100.74 | 8.84(12.99-5.71) | 10.77(16.13-6.08) | 0.695(0.533-0.857) |
| Guinea-Bissau | Male | 4.67(2.68-7.67) | 5.96(3.55-9.44) | 27.62 | 2.43(3.92-1.4) | 1.98(3.01-1.21) | -0.773(-0.984--0.561) |
| Guyana | Male | 5.96(4.35-7.92) | 4.79(3.23-6.71) | -19.63 | 3.31(4.33-2.44) | 1.73(2.38-1.18) | -2.072(-2.619--1.523) |
| Haiti | Male | 47.27(23.57-88.31) | 67.77(30.4-133.33) | 43.37 | 3.19(5.97-1.61) | 2.22(4.29-1) | -1.276(-1.633--0.918) |
| Honduras | Male | 51.95(16.37-87.14) | 217.13(92.62-347.83) | 317.96 | 5.37(9.04-1.64) | 7.88(12.53-3.4) | 1.316(0.898-1.735) |
| Hungary | Male | 319.01(259.69-374.73) | 163.51(120.97-215.23) | -48.74 | 5.19(6.07-4.24) | 2.09(2.74-1.55) | -3.194(-3.858--2.525) |
| Iceland | Male | 1.44(1.04-1.87) | 4.98(3.68-6.36) | 245.83 | 1.1(1.42-0.79) | 1.9(2.43-1.41) | 1.943(1.448-2.44) |
| India | Male | 2326.08(1728.76-2965.5) | 6690.07(5170.48-8431.67) | 187.61 | 1.07(1.36-0.79) | 1.23(1.55-0.96) | 0.539(0.128-0.953) |
| Indonesia | Male | 332.5(257.51-424.63) | 752.03(576.8-965) | 126.17 | 0.75(0.96-0.59) | 0.8(1-0.62) | 0.223(0.076-0.371) |
| Iran (Islamic Republic of) | Male | 92.13(66.28-126.63) | 216.69(163.03-286.6) | 135.20 | 0.75(1.02-0.56) | 0.65(0.86-0.48) | -0.598(-1.235-0.044) |
| Iraq | Male | 24.92(14.72-39.21) | 102.09(60.21-157.18) | 309.67 | 0.69(1.09-0.41) | 1(1.53-0.59) | 1.292(0.716-1.87) |
| Ireland | Male | 16.7(12.63-20.96) | 76.48(57.04-96.6) | 357.96 | 0.89(1.1-0.68) | 2.13(2.69-1.6) | 3.017(2.52-3.517) |
| Israel | Male | 23.93(16.3-32.58) | 70.74(50-93.58) | 195.61 | 1.05(1.43-0.72) | 1.34(1.77-0.96) | 0.77(0.629-0.911) |
| Italy | Male | 1345.75(1168.27-1513.13) | 1479.06(1239.58-1724.76) | 9.91 | 3.45(3.87-3) | 2.38(2.76-2) | -1.364(-1.794--0.932) |
| Jamaica | Male | 17.6(13.17-22.64) | 20.7(14.11-28.63) | 17.61 | 2.11(2.72-1.58) | 1.48(2.05-1.02) | -1.408(-2.96-0.168) |
| Japan | Male | 2450.3(1980.7-3139.12) | 3137.15(2527.84-4041.51) | 28.03 | 3.16(4.02-2.57) | 1.98(2.53-1.6) | -1.577(-1.84--1.314) |
| Jordan | Male | 2.98(1.77-4.59) | 13.61(7.85-21.68) | 356.71 | 0.5(0.77-0.3) | 0.45(0.7-0.26) | -0.425(-0.713--0.135) |
| Kazakhstan | Male | 104.64(75.44-132.05) | 271.79(194.92-355.62) | 159.74 | 2.24(2.81-1.66) | 3.96(5.14-2.86) | 1.843(1.304-2.385) |
| Kenya | Male | 38.54(20.1-79.81) | 129.85(73.7-216.95) | 236.92 | 1.01(2.05-0.53) | 1.34(2.21-0.76) | 0.955(0.443-1.468) |
| Kiribati | Male | 0.39(0.22-0.64) | 0.59(0.34-0.92) | 51.28 | 2.22(3.55-1.27) | 1.86(2.83-1.11) | -0.6(-0.769--0.43) |
| Kuwait | Male | 1.43(0.91-2.11) | 4.94(2.94-7.72) | 245.45 | 0.42(0.62-0.26) | 0.38(0.6-0.23) | 0.004(-0.76-0.773) |
| Kyrgyzstan | Male | 7.18(5.11-9.51) | 26.41(18.55-34.98) | 267.83 | 0.64(0.84-0.45) | 1.44(1.92-1.01) | 2.928(2.432-3.425) |
| Lao People's Democratic Republic | Male | 35.92(20.74-58.32) | 60.24(36.98-91.62) | 67.71 | 3.62(5.87-2.16) | 2.94(4.44-1.87) | -0.73(-0.791--0.669) |
| Latvia | Male | 14.6(11.02-17.9) | 32.08(22.57-43.27) | 119.73 | 1.14(1.4-0.87) | 2.22(2.98-1.57) | 2.263(1.703-2.825) |
| Lebanon | Male | 7.54(4.56-11.5) | 15.59(8.88-26.11) | 106.76 | 0.69(1.03-0.42) | 0.66(1.1-0.38) | -0.161(-0.392-0.07) |
| Lesotho | Male | 7.43(3.18-21.64) | 41.21(12.33-71.99) | 454.64 | 1.77(5.05-0.78) | 8.22(14.47-2.51) | 5.592(5.234-5.951) |
| Liberia | Male | 12.29(7.38-19.54) | 15.25(8.99-26.09) | 24.08 | 2.08(3.33-1.28) | 1.59(2.75-0.92) | -0.954(-1.317--0.59) |
| Libya | Male | 6.66(3.99-10.69) | 18.8(10.87-30.86) | 182.28 | 0.7(1.13-0.42) | 0.75(1.24-0.42) | 0.25(-0.151-0.652) |
| Lithuania | Male | 18.29(13.68-22.42) | 56.28(39.78-73.82) | 207.71 | 1.06(1.31-0.8) | 2.67(3.52-1.89) | 3.181(2.348-4.022) |
| Luxembourg | Male | 5.11(3.91-6.22) | 10.95(7.54-14.94) | 114.29 | 2.26(2.75-1.73) | 2.38(3.26-1.64) | 0.116(-0.285-0.517) |
| Madagascar | Male | 26.85(11.06-66.84) | 44.22(18.97-95.03) | 64.69 | 1.06(2.61-0.44) | 0.92(1.9-0.42) | -0.449(-0.769--0.129) |
| Malawi | Male | 21.27(11.23-41.14) | 40.28(25.98-57.97) | 89.37 | 1.2(2.31-0.65) | 1.39(1.98-0.92) | 0.466(0.133-0.799) |
| Malaysia | Male | 52.97(33.95-79.91) | 183.28(111.46-289.04) | 246.01 | 1.24(1.89-0.78) | 1.41(2.18-0.87) | 0.536(0.297-0.776) |
| Maldives | Male | 1.13(0.61-1.99) | 3.27(2.15-4.71) | 189.38 | 2.31(3.97-1.32) | 2.17(3.18-1.41) | -0.234(-0.52-0.053) |
| Mali | Male | 97.1(59.1-145.81) | 208.52(124.05-321.58) | 114.75 | 4.51(6.72-2.81) | 4.58(6.98-2.75) | 0.055(-0.152-0.262) |
| Malta | Male | 1.86(1.36-2.37) | 6.11(4.38-8.01) | 228.49 | 0.97(1.23-0.71) | 1.4(1.81-1.01) | 1.27(0.845-1.697) |
| Marshall Islands | Male | 0.19(0.1-0.32) | 0.38(0.21-0.67) | 100.00 | 2.38(4.11-1.2) | 2.14(3.69-1.18) | -0.334(-0.503--0.165) |
| Mauritania | Male | 8.09(5.01-12.47) | 10.07(5.79-16.03) | 24.47 | 1.74(2.64-1.08) | 1.02(1.62-0.59) | -1.811(-2.191--1.429) |
| Mauritius | Male | 2.21(1.52-3.02) | 6.6(4.07-10.04) | 198.64 | 0.7(0.94-0.49) | 0.86(1.31-0.54) | 0.724(0.022-1.431) |
| Mexico | Male | 172.08(151.51-192.06) | 887.15(692.71-1114.29) | 415.55 | 0.88(0.98-0.77) | 1.68(2.1-1.31) | 2.427(2.185-2.67) |
| Micronesia (Federated States of) | Male | 0.52(0.3-0.86) | 0.78(0.41-1.32) | 50.00 | 2.22(3.67-1.27) | 2.33(3.8-1.28) | 0.167(0.05-0.284) |
| Monaco | Male | 0.65(0.43-0.9) | 2.13(1.47-2.93) | 227.69 | 2.18(3-1.46) | 4.93(6.74-3.42) | 2.869(2.515-3.224) |
| Mongolia | Male | 146.94(93.23-209.25) | 522.93(345.79-747.45) | 255.88 | 31.28(44.1-20.33) | 56.79(77.94-39.45) | 2.061(1.909-2.214) |
| Montenegro | Male | 11.83(8.8-15.06) | 19.49(13.75-26.86) | 64.75 | 4.39(5.58-3.31) | 4.34(5.98-3.09) | -0.016(-0.303-0.271) |
| Morocco | Male | 23.57(13.57-37.63) | 65.62(38.45-101.73) | 178.40 | 0.38(0.59-0.22) | 0.47(0.73-0.28) | 0.672(0.085-1.263) |
| Mozambique | Male | 17.8(8.53-41.45) | 79.76(43.34-119.92) | 348.09 | 0.71(1.63-0.35) | 1.92(2.83-1.05) | 3.508(3.347-3.669) |
| Myanmar | Male | 93.45(55.5-153.53) | 304.6(194.2-443.89) | 225.95 | 0.95(1.54-0.58) | 1.65(2.32-1.09) | 1.939(1.358-2.524) |
| Namibia | Male | 1.96(0.79-5.24) | 9.43(6.01-13.75) | 381.12 | 0.6(1.56-0.24) | 1.59(2.28-1.03) | 3.509(3.161-3.857) |
| Nauru | Male | 0.04(0.02-0.07) | 0.04(0.02-0.08) | 0.00 | 2.03(3.19-1.17) | 2.27(3.5-1.3) | 0.408(0.333-0.483) |
| Nepal | Male | 34.43(22.56-49.5) | 108.53(61.47-187.47) | 215.22 | 0.8(1.15-0.52) | 1.09(1.87-0.63) | 1.077(0.893-1.262) |
| Netherlands | Male | 84.86(69.56-100.56) | 299.98(236.33-363.04) | 253.50 | 1(1.18-0.82) | 1.88(2.26-1.49) | 2.191(1.7-2.683) |
| New Zealand | Male | 27.55(23.68-31.15) | 88.38(76.85-100.67) | 220.80 | 1.55(1.75-1.34) | 2.5(2.85-2.18) | 1.686(1.315-2.058) |
| Nicaragua | Male | 10.45(7-14.31) | 39.35(27.02-54.75) | 276.56 | 1.52(2.07-1.02) | 2.09(2.88-1.44) | 1.04(0.579-1.503) |
| Niger | Male | 2.49(1.47-3.84) | 6.26(3.6-9.82) | 151.41 | 0.18(0.27-0.11) | 0.18(0.27-0.11) | -0.096(-0.256-0.064) |
| Nigeria | Male | 207.19(134.2-304.15) | 423.01(296.99-594.95) | 104.17 | 1.03(1.48-0.68) | 1.2(1.67-0.85) | 0.503(0.364-0.642) |
| Niue | Male | 0.02(0.01-0.02) | 0.02(0.01-0.03) | 0.00 | 1.76(2.65-1.08) | 1.91(2.77-1.21) | 0.282(0.203-0.362) |
| North Macedonia | Male | 46.02(34.17-58.78) | 81.9(55.4-113.03) | 77.97 | 5.16(6.58-3.85) | 5.27(7.23-3.61) | 0.154(-0.254-0.563) |
| Northern Mariana Islands | Male | 0.11(0.06-0.17) | 0.48(0.3-0.75) | 336.36 | 1(1.52-0.6) | 1.74(2.52-1.12) | 1.884(1.539-2.231) |
| Norway | Male | 25.26(22.3-28.47) | 62.02(51.11-74.5) | 145.53 | 0.86(0.96-0.76) | 1.39(1.67-1.15) | 1.673(1.433-1.915) |
| Oman | Male | 2.43(1.34-3.84) | 7.57(4.55-12.1) | 211.52 | 0.71(1.11-0.4) | 0.83(1.31-0.52) | 0.523(0.307-0.74) |
| Pakistan | Male | 191.87(116.99-277.48) | 399.59(265.39-570) | 108.26 | 0.6(0.87-0.36) | 0.66(0.96-0.44) | 0.311(0.104-0.52) |
| Palau | Male | 0.13(0.07-0.21) | 0.3(0.18-0.47) | 130.77 | 2.53(4.22-1.44) | 2.67(3.98-1.68) | 0.194(-0.007-0.396) |
| Palestine | Male | 4.68(2.64-7.34) | 10.5(6.41-15.78) | 124.36 | 1.29(2.01-0.73) | 1.08(1.62-0.66) | -0.548(-1.019--0.076) |
| Panama | Male | 11.79(8.95-14.93) | 33.27(22.56-48.14) | 182.19 | 1.57(2-1.19) | 1.67(2.42-1.13) | 0.291(-0.126-0.71) |
| Papua New Guinea | Male | 2.59(1.5-4.2) | 7.75(4.63-12.55) | 199.23 | 0.32(0.5-0.19) | 0.39(0.62-0.24) | 0.681(0.615-0.748) |
| Paraguay | Male | 13.2(9.27-17.11) | 32.6(20.76-46.12) | 146.97 | 1.29(1.68-0.9) | 1.29(1.84-0.82) | 0.008(-0.291-0.308) |
| Peru | Male | 134.22(95.41-181.14) | 176.6(110.02-263.78) | 31.58 | 2.43(3.26-1.73) | 1.17(1.74-0.72) | -2.4(-3.13--1.664) |
| Philippines | Male | 573.97(381.28-773.7) | 1059.15(755.01-1424.82) | 84.53 | 3.86(5.2-2.5) | 2.88(3.84-2.09) | -0.975(-1.124--0.825) |
| Poland | Male | 714.75(641.26-787.59) | 495.16(388.7-632.17) | -30.72 | 4.08(4.48-3.69) | 1.69(2.15-1.33) | -2.852(-3.528--2.172) |
| Portugal | Male | 81.45(62.95-99.8) | 359.45(268.31-447.69) | 341.31 | 1.33(1.61-1.03) | 3.62(4.52-2.72) | 3.477(3.191-3.763) |
| Puerto Rico | Male | 64.89(48.78-82.15) | 51.06(32.98-74.13) | -21.31 | 3.8(4.8-2.85) | 1.63(2.36-1.05) | -2.851(-3.47--2.229) |
| Qatar | Male | 1.32(0.75-2.09) | 9.56(5.16-15.49) | 624.24 | 2.76(4.44-1.57) | 2.19(3.55-1.23) | -0.848(-2.282-0.607) |
| Republic of Korea | Male | 425.83(261.92-642.05) | 2228.64(1553.9-3052.06) | 423.36 | 3.28(4.83-2.08) | 5.5(7.6-3.92) | 1.756(1.357-2.158) |
| Republic of Moldova | Male | 24.48(20.43-28.36) | 48.63(38.35-60.22) | 98.65 | 1.39(1.6-1.17) | 2.1(2.59-1.66) | 1.383(-0.359-3.155) |
| Romania | Male | 155.38(117.11-195.76) | 384.11(278.57-498.5) | 147.21 | 1.26(1.58-0.96) | 2.44(3.16-1.78) | 2.351(1.8-2.905) |
| Russian Federation | Male | 707.52(607.58-810.63) | 1823.82(1354.56-2415.08) | 157.78 | 1.14(1.29-1) | 2.07(2.71-1.55) | 2.185(1.41-2.966) |
| Rwanda | Male | 33.86(21.33-51.1) | 60.84(40.18-91.76) | 79.68 | 2.68(3.97-1.72) | 2.53(3.66-1.74) | -0.202(-0.489-0.085) |
| Saint Kitts and Nevis | Male | 1.12(0.8-1.47) | 0.68(0.47-0.94) | -39.29 | 6.51(8.48-4.76) | 2.18(2.97-1.57) | -4.071(-4.749--3.389) |
| Saint Lucia | Male | 1.4(1.06-1.78) | 1.5(1.1-1.97) | 7.14 | 3.66(4.59-2.8) | 1.52(2-1.11) | -3.001(-3.382--2.618) |
| Saint Vincent and the Grenadines | Male | 1.3(0.95-1.68) | 1.29(0.95-1.66) | -0.77 | 4.14(5.31-3.04) | 1.87(2.39-1.38) | -2.671(-3.351--1.987) |
| Samoa | Male | 0.63(0.36-0.99) | 0.84(0.51-1.26) | 33.33 | 1.45(2.22-0.86) | 1.16(1.73-0.72) | -0.771(-0.886--0.657) |
| San Marino | Male | 0.22(0.16-0.3) | 0.56(0.33-0.86) | 154.55 | 1.44(1.92-1.03) | 1.89(2.91-1.09) | 0.985(0.899-1.071) |
| Sao Tome and Principe | Male | 0.22(0.14-0.32) | 0.5(0.27-0.77) | 127.27 | 0.76(1.09-0.49) | 1.03(1.57-0.57) | 1.037(0.69-1.385) |
| Saudi Arabia | Male | 23.01(13.24-37.88) | 53.19(30.78-87.27) | 131.16 | 0.75(1.21-0.43) | 0.63(1.02-0.37) | -0.624(-0.822--0.425) |
| Senegal | Male | 5.85(3.65-8.49) | 12.1(6.94-19.14) | 106.84 | 0.37(0.54-0.24) | 0.36(0.55-0.21) | -0.072(-0.752-0.611) |
| Serbia | Male | 185.37(133.87-240.66) | 255.5(174.31-356.58) | 37.83 | 3.49(4.52-2.52) | 3.41(4.73-2.36) | -0.057(-0.284-0.171) |
| Seychelles | Male | 0.77(0.5-1.12) | 1.39(0.9-1.99) | 80.52 | 3.09(4.45-2.04) | 2.64(3.71-1.74) | -0.531(-0.683--0.378) |
| Sierra Leone | Male | 17.95(11.33-28.32) | 24.38(14.42-38.07) | 35.82 | 1.85(2.91-1.17) | 1.46(2.25-0.88) | -0.807(-1.096--0.517) |
| Singapore | Male | 11.57(7.74-16.62) | 44.48(28.21-66.99) | 284.44 | 1.17(1.68-0.78) | 1.22(1.84-0.78) | 0.151(-0.252-0.555) |
| Slovakia | Male | 90.91(71.35-109.16) | 107.49(72.09-147.89) | 18.24 | 3.53(4.23-2.82) | 2.71(3.68-1.82) | -0.958(-1.163--0.754) |
| Slovenia | Male | 20.58(14.46-28.06) | 72.82(49.24-102.27) | 253.84 | 2.13(2.88-1.51) | 3.8(5.34-2.57) | 2.037(1.315-2.764) |
| Solomon Islands | Male | 0.68(0.39-1.13) | 1.29(0.77-1.96) | 89.71 | 0.95(1.59-0.54) | 0.92(1.4-0.57) | -0.102(-0.276-0.072) |
| Somalia | Male | 13.89(6.7-36.1) | 31.51(15.41-70.59) | 126.85 | 1.28(3.25-0.61) | 1.24(2.64-0.61) | -0.098(-0.267-0.07) |
| South Africa | Male | 189.98(101.95-416.56) | 450.49(356.69-553.37) | 137.12 | 2.1(4.64-1.12) | 2.42(2.94-1.92) | 0.578(-0.163-1.325) |
| South Sudan | Male | 13.78(5.65-33.89) | 18.17(7.66-41.75) | 31.86 | 1.07(2.61-0.45) | 0.98(2.21-0.43) | -0.298(-0.434--0.162) |
| Spain | Male | 547.27(388.54-699.11) | 1113.91(774.5-1478.96) | 103.54 | 2.26(2.89-1.63) | 2.71(3.59-1.89) | 0.593(0.425-0.761) |
| Sri Lanka | Male | 37.29(24.83-53.02) | 149.18(89.75-228.49) | 300.05 | 0.74(1.05-0.49) | 1.32(1.99-0.82) | 2.125(1.653-2.598) |
| Sudan | Male | 37.08(18.3-76.22) | 82.2(40.98-163.92) | 121.68 | 0.79(1.59-0.4) | 0.9(1.77-0.45) | 0.418(0.267-0.568) |
| Suriname | Male | 4.1(2.92-5.35) | 4.26(3.01-6.04) | 3.90 | 3.4(4.41-2.44) | 1.58(2.22-1.13) | -2.635(-3.083--2.184) |
| Sweden | Male | 115.63(100.92-129.45) | 232.71(200.72-263.71) | 101.25 | 1.73(1.94-1.52) | 2.41(2.73-2.08) | 1.144(0.821-1.467) |
| Switzerland | Male | 91.47(71.3-109.83) | 262.02(195.17-329.67) | 186.45 | 2.08(2.5-1.62) | 3.31(4.14-2.48) | 1.574(1.272-1.878) |
| Syrian Arab Republic | Male | 18.22(10.65-28.43) | 37.02(20.67-59.74) | 103.18 | 0.71(1.09-0.42) | 0.61(0.98-0.34) | -0.599(-0.887--0.311) |
| Taiwan (Province of China) | Male | 226.92(148.67-323.18) | 230.26(138.43-359.33) | 1.47 | 2.49(3.51-1.63) | 1.24(1.95-0.75) | -2.647(-3.436--1.851) |
| Tajikistan | Male | 4.57(3.09-6.39) | 29.96(19.23-43.9) | 555.58 | 0.4(0.56-0.27) | 1.46(2.13-0.93) | 4.56(4.309-4.811) |
| Thailand | Male | 1585.12(1081.79-2196.22) | 6244.22(4001.38-9166.53) | 293.93 | 9.61(13.17-6.73) | 13.26(19.26-8.46) | 1.039(0.705-1.374) |
| Timor-Leste | Male | 3.06(1.55-5.25) | 8.92(4.26-15.14) | 191.50 | 2.21(3.66-1.17) | 2.22(3.78-1.1) | 0.028(-0.168-0.225) |
| Togo | Male | 10.76(6.49-16.95) | 24.41(14.78-37.49) | 126.86 | 1.92(3.04-1.16) | 1.74(2.61-1.08) | -0.351(-0.447--0.255) |
| Tokelau | Male | 0.01(0-0.01) | 0.01(0-0.01) | 0.00 | 1.41(2.32-0.69) | 1.34(2.1-0.71) | -0.171(-0.209--0.133) |
| Tonga | Male | 1.4(0.75-2.25) | 2.3(1.33-3.57) | 64.29 | 5.17(8.15-2.82) | 6.27(9.7-3.65) | 0.697(0.515-0.879) |
| Trinidad and Tobago | Male | 13.01(9.64-16.57) | 12.1(7.93-17.38) | -6.99 | 3.29(4.18-2.44) | 1.38(1.96-0.91) | -2.976(-3.395--2.557) |
| Tunisia | Male | 8.83(4.79-14.87) | 22.53(11.28-40.56) | 155.15 | 0.34(0.57-0.19) | 0.37(0.67-0.19) | 0.281(0.056-0.507) |
| Turkey | Male | 125.58(77.38-192.34) | 271.81(169.32-418.02) | 116.44 | 0.8(1.21-0.49) | 0.68(1.03-0.42) | -0.506(-0.816--0.194) |
| Turkmenistan | Male | 3.47(2.41-4.62) | 53.72(35.13-75.85) | 1448.13 | 0.5(0.67-0.34) | 3.02(4.25-2.03) | 6.333(5.27-7.407) |
| Tuvalu | Male | 0.06(0.03-0.1) | 0.09(0.05-0.14) | 50.00 | 2.02(3.45-1.07) | 1.78(2.97-0.99) | -0.437(-0.55--0.324) |
| Uganda | Male | 85.8(56.49-117.75) | 214.57(145.47-303.11) | 150.08 | 2.7(3.67-1.82) | 3.44(4.77-2.4) | 0.808(0.549-1.068) |
| Ukraine | Male | 135.78(117.48-152.56) | 524.75(410.13-662.56) | 286.47 | 0.56(0.63-0.49) | 1.86(2.32-1.46) | 4.11(3.679-4.542) |
| United Arab Emirates | Male | 1.97(0.76-4.25) | 20.78(6.36-57.18) | 954.82 | 0.86(2.02-0.31) | 0.82(2.25-0.26) | -0.123(-0.559-0.316) |
| United Kingdom | Male | 467.6(417.43-516.59) | 1440.85(1267.34-1616.66) | 208.14 | 1.21(1.34-1.09) | 2.47(2.76-2.17) | 2.481(2.213-2.75) |
| United Republic of Tanzania | Male | 43.56(27.72-66.7) | 115.13(73.2-174.91) | 164.30 | 0.88(1.31-0.58) | 1.09(1.64-0.7) | 0.743(0.298-1.189) |
| United States of America | Male | 1543.99(1375.66-1704.72) | 6413.51(5147.12-7656.24) | 315.39 | 1.13(1.25-1.01) | 2.52(3-2.03) | 2.807(2.701-2.913) |
| United States Virgin Islands | Male | 0.76(0.53-1.03) | 1.35(0.95-1.89) | 77.63 | 1.98(2.65-1.37) | 1.57(2.15-1.12) | -0.689(-1.055--0.32) |
| Uruguay | Male | 13.21(9.15-17.78) | 31.83(22.7-41.09) | 140.95 | 0.77(1.02-0.53) | 1.41(1.82-1.01) | 2.169(1.642-2.699) |
| Uzbekistan | Male | 16.79(11.97-21.78) | 275.81(184.06-379.49) | 1542.70 | 0.4(0.52-0.28) | 3.15(4.32-2.17) | 7.483(6.867-8.103) |
| Vanuatu | Male | 0.62(0.29-1.15) | 1.74(0.88-3) | 180.65 | 1.72(3.19-0.82) | 1.93(3.32-0.99) | 0.414(0.274-0.555) |
| Venezuela (Bolivarian Republic of) | Male | 147.91(112.21-181.48) | 197.33(131.32-285.02) | 33.41 | 3.36(4.14-2.54) | 1.5(2.15-1) | -3.118(-4.346--1.874) |
| Viet Nam | Male | 221.15(132.75-347.54) | 454.15(279.23-673.78) | 105.36 | 1.35(2.13-0.81) | 1.27(1.86-0.8) | -0.264(-0.409--0.12) |
| Yemen | Male | 7.98(3.98-15.36) | 24.05(13.44-41.61) | 201.38 | 0.38(0.73-0.2) | 0.4(0.69-0.22) | 0.207(0.164-0.25) |
| Zambia | Male | 13.46(7.21-26.32) | 39.68(26.28-55.81) | 194.80 | 1(1.96-0.54) | 1.44(2.02-0.99) | 1.268(1.062-1.474) |
| Zimbabwe | Male | 68.33(37.12-145.27) | 107.22(61.54-193.91) | 56.91 | 3.35(7.09-1.84) | 3.55(6.34-2.05) | 0.289(-0.267-0.848) |

**Table S8** The DALYs and age-standardized DALY rate of liver cancer attributable to alcohol use in 1990 and 2019, and its temporal trends from 1990 to 2019.

| nation | sex | DLAY NO(95% UI) | | Change in absolute number (%) | age-standardized DALY rate per 100,000 No. (95% UI) | | 1990-2019 AAPC No.(95 CI) |
| --- | --- | --- | --- | --- | --- | --- | --- |
|  |  | 1990 | 2019 |  | 1990 | 2019 |  |
| Afghanistan | Both | 1852.01(1073.24-2883.16) | 3012.61(1828.22-4773.05) | 62.67 | 24.53(37.66-14.49) | 21.25(32.72-12.96) | -0.504(-0.565--0.444) |
| Albania | Both | 1692.47(1213.92-2204.19) | 2474.18(1552.39-3716.52) | 46.19 | 78.6(102.21-56.9) | 57.78(85.11-36.42) | -1.072(-1.578--0.564) |
| Algeria | Both | 473.21(284.85-728.48) | 1698.3(987.17-2624.14) | 258.89 | 3.68(5.58-2.25) | 4.94(7.68-2.9) | 0.964(0.683-1.246) |
| American Samoa | Both | 4.06(2.37-6.3) | 10.27(6.35-15.72) | 152.96 | 15.76(24.21-9.45) | 19.94(29.64-12.59) | 0.85(0.711-0.988) |
| Andorra | Both | 51.16(34.2-76.04) | 114.71(75.98-161.36) | 124.22 | 87.48(130.57-58.76) | 83.81(117.97-55.64) | -0.13(-0.222--0.038) |
| Angola | Both | 392.91(230.09-616.07) | 1348.65(853.36-1985.8) | 243.25 | 9.09(14.25-5.42) | 10.64(15.64-6.83) | 0.558(0.453-0.664) |
| Antigua and Barbuda | Both | 31.13(22.37-40.77) | 23.12(15.99-31.33) | -25.73 | 60.87(80.94-42.93) | 22.13(29.84-15.48) | -3.447(-4.067--2.823) |
| Argentina | Both | 3836.03(2730.89-5072.71) | 8355.44(6075.9-10710.5) | 117.81 | 11.65(15.32-8.33) | 15.69(20.16-11.52) | 1.062(0.734-1.391) |
| Armenia | Both | 172.09(119.71-230.79) | 1935.22(1321.29-2623.15) | 1024.54 | 6.2(8.26-4.39) | 46.58(62.53-32.39) | 7.15(5.397-8.932) |
| Australia | Both | 3975.29(3086.6-4821.44) | 14774(10983.69-18446.64) | 271.65 | 20.47(24.91-15.89) | 38.87(48.59-29) | 2.248(2.077-2.419) |
| Austria | Both | 4761.08(3953.14-5555.21) | 8775.03(7084.19-10583.74) | 84.31 | 43.79(51.09-36.31) | 54.27(65.37-43.93) | 0.717(0.394-1.041) |
| Azerbaijan | Both | 281.82(193.08-384.41) | 2632.1(1644.71-3999.72) | 833.96 | 5.53(7.45-3.82) | 26.42(39.61-16.7) | 5.569(4.955-6.187) |
| Bahamas | Both | 128.36(94.09-168.11) | 109.16(73.8-152.93) | -14.96 | 78.59(102.04-56.7) | 26.46(36.73-18.23) | -3.742(-4.214--3.268) |
| Bahrain | Both | 26.85(16.7-39.79) | 110.84(63.85-174.62) | 312.81 | 14.9(22.46-9.28) | 10.66(16.6-6.4) | -1.236(-1.819--0.649) |
| Bangladesh | Both | 9444(6250.43-13692.29) | 18162.85(11564.53-26252.07) | 92.32 | 18.02(26.02-12) | 13.38(19.35-8.5) | -0.967(-1.162--0.771) |
| Barbados | Both | 61.47(38.66-93.06) | 108.83(74.41-145.86) | 77.05 | 22.29(34.18-13.9) | 22.32(29.71-15.43) | -0.094(-0.445-0.258) |
| Belarus | Both | 1468.77(1099.85-1833.29) | 3898.48(2434.16-5820.23) | 165.42 | 11.18(13.94-8.44) | 25.08(37.48-15.47) | 2.824(1.918-3.738) |
| Belgium | Both | 3530.52(2679.26-4358.66) | 6810.57(5118.03-8609.72) | 92.91 | 23.71(29.27-18.12) | 33.6(41.96-25.29) | 1.256(0.839-1.674) |
| Belize | Both | 42.54(30.79-55.89) | 73.84(53.2-98.27) | 73.58 | 45.26(59.34-32.51) | 25.79(34.07-18.58) | -1.906(-2.202--1.609) |
| Benin | Both | 617.02(395.91-947.51) | 1221.34(734.77-1940.08) | 97.94 | 29.75(45.89-18.84) | 23.24(36.56-14.23) | -0.841(-0.974--0.707) |
| Bermuda | Both | 42.08(31.52-53.1) | 21.47(15.11-29.08) | -48.98 | 65.96(83.11-49.28) | 17.01(23.06-11.95) | -4.598(-5.194--3.998) |
| Bhutan | Both | 47.56(26.72-81.4) | 124.98(71.1-197.14) | 162.78 | 17.08(28.7-9.81) | 21.4(33.76-12.23) | 0.769(0.604-0.933) |
| Bolivia (Plurinational State of) | Both | 1092.62(668.8-1611.99) | 2933.71(1906.43-4302.82) | 168.50 | 33.06(49.61-20.58) | 32.75(47.95-21.52) | -0.038(-0.151-0.075) |
| Bosnia and Herzegovina | Both | 2011.71(1412.29-2634.95) | 3680.45(2505.56-5198.03) | 82.95 | 45.61(58.97-33.07) | 60.95(85.72-41.3) | 0.977(0.617-1.337) |
| Botswana | Both | 30.11(10.89-87.87) | 140(82.41-226.16) | 364.96 | 4.72(13.7-1.74) | 8.54(13.47-5.1) | 2.154(1.902-2.406) |
| Brazil | Both | 13243.44(11488.55-15208.86) | 42771.29(36713.56-48796.08) | 222.96 | 13.83(15.84-12) | 17.67(20.13-15.21) | 0.834(0.621-1.048) |
| Brunei Darussalam | Both | 25.01(15.42-37.38) | 92.27(56.32-146.61) | 268.93 | 22.65(34.09-13.97) | 25.47(38.88-15.68) | 0.417(0.071-0.764) |
| Bulgaria | Both | 8331.26(6311.24-10398.72) | 6796.84(4893.39-9246.23) | -18.42 | 64.71(80.81-49.26) | 52.22(70.96-37.02) | -0.8(-1.381--0.216) |
| Burkina Faso | Both | 517.15(322.95-746.73) | 932(578.36-1341.71) | 80.22 | 11.23(16.2-7.12) | 9.36(13.43-5.88) | -0.574(-0.705--0.443) |
| Burundi | Both | 784.02(489.73-1163.23) | 1068.05(595.4-1915.53) | 36.23 | 31.64(46.47-19.95) | 21.33(38.22-11.79) | -1.361(-1.557--1.164) |
| Cabo Verde | Both | 9.87(6.61-14) | 249.03(169.12-353.64) | 2423.10 | 4.38(6.22-2.92) | 56.44(79.23-38.36) | 9.346(8.625-10.072) |
| Cambodia | Both | 1562.03(994.23-2324.33) | 5202.47(3295.6-7639.83) | 233.06 | 31.34(46.78-20.05) | 40.1(57.95-25.75) | 0.881(0.677-1.086) |
| Cameroon | Both | 147.98(83.73-258.23) | 469.25(284.15-738.92) | 217.10 | 3.09(5.38-1.74) | 3.53(5.47-2.18) | 0.479(0.301-0.657) |
| Canada | Both | 8450.01(7278.04-9599.43) | 32452(26073.76-38593.65) | 284.05 | 26.41(29.97-22.74) | 50.96(60.34-41.11) | 2.354(2.104-2.604) |
| Central African Republic | Both | 205.71(115.08-335.26) | 329.84(175.63-588.63) | 60.34 | 15.63(24.91-8.95) | 13.51(23.3-7.45) | -0.513(-0.717--0.31) |
| Chad | Both | 689.91(405.65-1071.27) | 1273.67(762.03-1973.49) | 84.61 | 23.44(36.38-13.84) | 21.34(32.62-12.95) | -0.316(-0.446--0.186) |
| Chile | Both | 1695.25(1256.02-2190.8) | 5050.76(3649.57-6574.2) | 197.94 | 16.4(21.25-12.03) | 20.94(27.15-15.15) | 0.847(0.434-1.261) |
| China | Both | 738587.13(458340.37-1115685.82) | 630023.72(416839.65-917031.4) | -14.70 | 75.52(112.5-48.19) | 30.48(44.31-20.08) | -3.065(-3.434--2.694) |
| Colombia | Both | 3861.09(2819.55-4947.65) | 9632.59(6271.35-13751.66) | 149.48 | 20.67(26.47-14.89) | 18.39(26.22-11.97) | -0.426(-1.438-0.595) |
| Comoros | Both | 37.5(15.71-78.35) | 71.45(37.81-142.94) | 90.53 | 16.26(34.04-7.02) | 14.16(27.81-7.55) | -0.497(-1.203-0.214) |
| Congo | Both | 193.47(112.43-315.22) | 367.46(207.73-573.81) | 89.93 | 16.38(26.28-9.59) | 12.51(19.63-7.43) | -0.899(-1.025--0.772) |
| Cook Islands | Both | 5.56(3.36-8.24) | 12.08(7.72-17.55) | 117.27 | 41.11(60.3-25.07) | 49.6(72.69-31.85) | 0.681(0.482-0.881) |
| Costa Rica | Both | 804.13(590.3-1021.99) | 2155.21(1438.99-3091.84) | 168.02 | 44.72(56.95-32.71) | 41.65(59.9-27.79) | -0.155(-0.857-0.552) |
| Croatia | Both | 2102.74(1602.28-2605.11) | 3137.22(2176.91-4266.9) | 49.20 | 31.98(39.3-24.46) | 38.07(52.22-26.33) | 0.621(0.08-1.166) |
| Cuba | Both | 5107(3773.37-6511.42) | 3618.6(2490.85-4999.51) | -29.14 | 49.35(63.14-36.44) | 19.32(26.74-13.26) | -3.076(-3.887--2.259) |
| Cyprus | Both | 224.75(156.6-301.4) | 513.03(374.71-671.26) | 128.27 | 26.49(35.39-18.66) | 26.35(34.6-19.3) | -0.073(-0.293-0.147) |
| Czechia | Both | 6791.87(5413.86-8082.11) | 6553.59(4726.01-8632.83) | -3.51 | 49.51(58.84-39.19) | 32.66(43.13-23.6) | -1.404(-2.164--0.638) |
| C么te d'Ivoire | Both | 1712.36(1005.23-2535.83) | 2994.37(1788.64-4644.42) | 74.87 | 37.42(56.94-22.51) | 25.17(39.29-15.25) | -1.372(-1.631--1.113) |
| Democratic People's Republic of Korea | Both | 7707.98(4197.06-12711.52) | 9636.62(5436.77-15673.97) | 25.02 | 40.32(66.19-22.96) | 28.8(46.24-16.22) | -1.157(-1.252--1.062) |
| Democratic Republic of the Congo | Both | 1500.64(957.65-2210.88) | 3079.06(1812.53-4811.63) | 105.18 | 8.5(12.37-5.46) | 7.44(11.38-4.42) | -0.464(-0.678--0.25) |
| Denmark | Both | 1384.1(1073.79-1704.16) | 3149.51(2325.18-4016.9) | 127.55 | 18.25(22.49-14.15) | 29.8(37.98-21.94) | 1.746(1.59-1.903) |
| Djibouti | Both | 30.88(14.5-68.22) | 130.4(64.58-257.28) | 322.28 | 19.17(42.16-9.31) | 19.72(38.07-10.26) | 0.125(-0.101-0.351) |
| Dominica | Both | 48.47(34.77-63.74) | 25.04(17-35) | -48.34 | 70.65(94.38-50.08) | 28.21(39.36-19.14) | -3.113(-3.456--2.77) |
| Dominican Republic | Both | 1303.39(938.67-1731.91) | 3929.01(2286.12-6897.23) | 201.45 | 32.61(43.27-23.49) | 41.14(71.41-24.27) | 0.785(0.516-1.055) |
| Ecuador | Both | 934.2(656.7-1259.78) | 3403.27(2257.93-4906.42) | 264.30 | 17.12(23.14-12.03) | 22.44(32.27-14.84) | 0.915(0.564-1.267) |
| Egypt | Both | 10983.78(6429.22-17349.32) | 34334.29(18728.3-59913.48) | 212.59 | 33.66(52.63-19.89) | 47.16(81.71-25.93) | 1.227(1.035-1.419) |
| El Salvador | Both | 682.92(483.58-906.48) | 861.49(554.72-1262) | 26.15 | 22.35(29.58-15.9) | 14.84(21.67-9.5) | -1.489(-1.852--1.125) |
| Equatorial Guinea | Both | 24.28(14.1-38.6) | 76.6(37.91-127.91) | 215.49 | 11.11(17.35-6.62) | 14.32(24.19-7.23) | 0.957(0.792-1.121) |
| Eritrea | Both | 219.51(117.24-399.25) | 545.71(297.96-955.62) | 148.60 | 19.37(34.95-10.45) | 18.05(30.75-10.12) | -0.185(-0.505-0.136) |
| Estonia | Both | 384.33(283.35-480.46) | 888.95(630.71-1194.54) | 131.30 | 18.78(23.51-13.92) | 37.69(51.77-26.45) | 2.748(1.985-3.517) |
| Eswatini | Both | 93.89(46.62-227.5) | 822.73(190.4-1705.17) | 776.27 | 28.31(68.02-14.4) | 122.56(253.97-29.39) | 5.235(4.89-5.582) |
| Ethiopia | Both | 3608.69(2383.89-5328.65) | 6476.07(4713.81-8865.89) | 79.46 | 16.75(24.19-11.14) | 15.27(20.9-11.21) | -0.296(-0.441--0.15) |
| Fiji | Both | 75.27(47-114.58) | 179.61(107.33-284.22) | 138.62 | 18.37(28.08-11.48) | 21.55(33.61-13.13) | 0.609(0.39-0.828) |
| Finland | Both | 1719.2(1301.92-2135.65) | 3779.78(2809.93-4788.75) | 119.86 | 24.79(30.87-18.69) | 33.23(42.06-25.1) | 1.017(0.854-1.18) |
| France | Both | 34974.69(25731.94-44297.73) | 54146.65(37982.38-71890.72) | 54.82 | 45.41(57.42-33.53) | 45.87(61.01-32.34) | 0.015(-0.219-0.249) |
| Gabon | Both | 93.13(53.62-139.55) | 178.12(93.19-307.3) | 91.26 | 15.47(23.19-9) | 15.6(27.17-8.15) | 0.062(-0.145-0.268) |
| Gambia | Both | 577.84(349.64-895.54) | 2155.72(1226.06-3368.95) | 273.07 | 143.01(221.36-89.21) | 202.03(311.98-114.57) | 1.205(0.65-1.763) |
| Georgia | Both | 447.02(303.45-601.59) | 1740.61(1213.37-2418.98) | 289.38 | 7.04(9.46-4.84) | 31.86(44.11-21.98) | 5.427(3.962-6.913) |
| Germany | Both | 29813.03(23957.41-35641.44) | 71741.81(58255.32-85689.87) | 140.64 | 24.73(29.54-19.89) | 42.32(50.88-34.42) | 1.898(1.732-2.065) |
| Ghana | Both | 2022.91(1283.27-3184.57) | 5780.15(3660.23-8574.3) | 185.73 | 29.53(45.82-18.35) | 32.67(47.75-20.77) | 0.35(0.165-0.535) |
| Greece | Both | 2402(1778.28-3033.19) | 4909.51(3654.59-6336.71) | 104.39 | 15.57(19.63-11.64) | 23.19(30.08-16.84) | 1.403(1.145-1.662) |
| Greenland | Both | 22.01(15.82-28.85) | 51.57(34.04-73.2) | 134.30 | 51.86(67.5-38.14) | 67.47(93.14-45.49) | 0.905(0.666-1.144) |
| Grenada | Both | 51.11(37.25-65.97) | 31.88(23.16-41.51) | -37.62 | 75.89(99.28-55.15) | 27.36(35.47-20) | -3.546(-3.876--3.215) |
| Guam | Both | 10.01(6.29-14.65) | 40.32(25.2-60.89) | 302.80 | 11.48(16.75-7.24) | 20.7(30.83-13.04) | 2.035(1.617-2.456) |
| Guatemala | Both | 2261.08(1557.1-3071.67) | 3198.72(2072.37-4717.21) | 41.47 | 57.27(76.98-39.5) | 28.03(41.54-18.01) | -2.461(-3.035--1.884) |
| Guinea | Both | 4310.09(2773.77-6305.63) | 8585.6(4926.03-13187.98) | 99.20 | 124.1(182-80.46) | 147.06(224.75-84.72) | 0.609(0.416-0.803) |
| Guinea-Bissau | Both | 158.04(92.61-263.34) | 223.52(133.02-353.51) | 41.43 | 35.92(58.58-20.96) | 27.82(43.22-16.62) | -0.89(-1.081--0.699) |
| Guyana | Both | 242.15(169.94-322.27) | 175.96(115.38-251.36) | -27.33 | 59.33(77.96-41.73) | 26.45(37.23-17.94) | -2.693(-3.147--2.237) |
| Haiti | Both | 1827.42(1054.56-2847.84) | 2459.13(1340.54-4216.36) | 34.57 | 53.1(84-30.93) | 33.68(57.8-18.04) | -1.58(-1.694--1.466) |
| Honduras | Both | 1592.56(527.89-2621.36) | 6394.22(2622.67-10721.14) | 301.51 | 74.24(122.73-23.8) | 102.7(170.34-42.11) | 1.156(0.9-1.412) |
| Hungary | Both | 10423.82(8371.62-12454.34) | 4647.6(3332.4-6247.02) | -55.41 | 70.74(84.14-56.89) | 25.55(34.36-18.15) | -3.491(-4.239--2.737) |
| Iceland | Both | 38.85(28.59-50.69) | 123.17(90.14-158.67) | 217.04 | 14.14(18.4-10.37) | 23.75(30.73-17.57) | 1.794(1.365-2.224) |
| India | Both | 79042.92(60434.42-99263.76) | 217886.04(170796.22-270744.54) | 175.66 | 16.24(20.41-12.31) | 18.19(22.58-14.28) | 0.465(0.1-0.832) |
| Indonesia | Both | 11511.61(9127.66-14216.45) | 22203.35(16868.16-28018.71) | 92.88 | 10.72(13.43-8.47) | 9.78(12.26-7.53) | -0.332(-0.497--0.167) |
| Iran (Islamic Republic of) | Both | 2915.88(2104.46-3923.87) | 6185.11(4618.41-8122.08) | 112.12 | 10.77(14.16-7.81) | 8.52(11.17-6.36) | -0.85(-1.257--0.441) |
| Iraq | Both | 827.37(488.25-1269.3) | 3293.79(1960.18-5077.63) | 298.10 | 10.29(15.8-6.1) | 13.74(20.97-8.28) | 0.992(0.412-1.575) |
| Ireland | Both | 477.3(356.7-603.18) | 1993.02(1489.2-2523.63) | 317.56 | 11.72(14.75-8.71) | 27.35(34.64-20.35) | 2.917(2.543-3.292) |
| Israel | Both | 662.05(456.77-902.24) | 1829.62(1279.06-2436.21) | 176.36 | 13.6(18.48-9.4) | 16.59(22.09-11.6) | 0.654(0.439-0.87) |
| Italy | Both | 37400.24(32241.98-42370.13) | 35435.95(29633.43-41085.13) | -5.25 | 43.13(48.82-37.25) | 28.96(33.72-24.16) | -1.465(-2.129--0.797) |
| Jamaica | Both | 539.26(400.96-693.56) | 618.34(418.43-867.04) | 14.66 | 30.94(39.99-22.8) | 21.06(29.53-14.22) | -1.284(-2.258--0.3) |
| Japan | Both | 76551.78(61078.71-100079.07) | 71112.91(56864.98-90973.67) | -7.10 | 44.05(57.62-35.18) | 23.76(30.53-18.96) | -2.046(-2.443--1.647) |
| Jordan | Both | 97.03(58.18-146.69) | 383.72(221.31-608.8) | 295.47 | 7.06(10.72-4.25) | 5.76(9.11-3.4) | -0.702(-0.882--0.521) |
| Kazakhstan | Both | 3792.85(2734.28-4912.28) | 9430.46(6563.65-12508.18) | 148.64 | 28.51(36.59-20.56) | 51.11(67.24-36.05) | 1.878(1.364-2.394) |
| Kenya | Both | 1479.68(895.44-2739.71) | 5013.65(3050.86-7900.69) | 238.83 | 16.81(31.13-10.15) | 20.63(32.24-12.67) | 0.62(0.145-1.098) |
| Kiribati | Both | 17.41(10.55-27.52) | 28.6(16.41-43.88) | 64.27 | 40.17(62.55-24.24) | 34.07(51.24-20.14) | -0.561(-0.656--0.466) |
| Kuwait | Both | 45.28(28.74-67.33) | 138.63(81.17-216.86) | 206.16 | 6.81(10.05-4.33) | 5.47(8.58-3.26) | -0.578(-1.269-0.117) |
| Kyrgyzstan | Both | 250.71(177.27-334.67) | 915.87(630.94-1227.93) | 265.31 | 8.17(10.78-5.73) | 19.08(25.47-13.37) | 3.057(2.513-3.604) |
| Lao People's Democratic Republic | Both | 1249(727.35-2050.72) | 2011.05(1238.32-3063.67) | 61.01 | 54.71(88.19-32.15) | 41.87(63.43-25.97) | -0.929(-0.977--0.88) |
| Latvia | Both | 524.37(388.32-662.05) | 985.24(722.96-1300.37) | 87.89 | 14.71(18.56-10.96) | 27.96(37.22-20.32) | 2.309(1.674-2.949) |
| Lebanon | Both | 254.29(154.7-380.65) | 453.22(261.85-753.58) | 78.23 | 10.63(15.79-6.62) | 8.7(14.5-4.98) | -0.734(-0.928--0.539) |
| Lesotho | Both | 273.25(127.57-676.32) | 1326.05(430.45-2250.83) | 385.29 | 25.78(63.29-12.13) | 93.59(156.79-30.71) | 4.64(4.415-4.865) |
| Liberia | Both | 377.41(235.5-581.79) | 527.92(316.26-855.71) | 39.88 | 32.28(49.51-20.36) | 23.25(37.72-13.83) | -1.162(-1.424--0.9) |
| Libya | Both | 232.71(138.93-367.36) | 644.59(375.39-1056.94) | 176.99 | 11.95(18.68-7.16) | 11.9(19.43-6.89) | -0.016(-0.351-0.319) |
| Lithuania | Both | 647.32(471.1-806.94) | 1707.69(1209.37-2251.44) | 163.81 | 14.42(17.92-10.56) | 33.77(44.6-23.95) | 2.953(2.167-3.745) |
| Luxembourg | Both | 150.19(113.17-185.36) | 297.47(207.79-402.48) | 98.06 | 28.08(34.61-21.38) | 31.47(42.5-21.88) | 0.339(0.165-0.513) |
| Madagascar | Both | 990.28(513.13-2051.18) | 1816.75(971.1-3315.18) | 83.46 | 18.05(37.3-9.28) | 15(27.3-8.14) | -0.625(-0.908--0.341) |
| Malawi | Both | 796.77(449.43-1432.79) | 1320.77(848.12-1913.06) | 65.77 | 18.87(33.3-10.73) | 17.36(24.96-11.29) | -0.266(-0.69-0.161) |
| Malaysia | Both | 1748.6(1147.35-2540.71) | 5539.69(3390.93-8690.31) | 216.81 | 18.09(26.61-11.75) | 19.69(30.5-12.13) | 0.357(0.173-0.541) |
| Maldives | Both | 32.63(17.68-59.77) | 89.31(57.35-131.73) | 173.71 | 34.4(60.34-19.25) | 27.7(40.41-17.74) | -0.781(-1.083--0.479) |
| Mali | Both | 3058.06(1860.77-4613.01) | 6562.07(3932.38-10214.77) | 114.58 | 66.05(98.92-40.51) | 68.05(106.52-41.23) | 0.099(-0.097-0.295) |
| Malta | Both | 51.92(37.35-66.54) | 153.32(110.8-201.76) | 195.30 | 11.92(15.26-8.58) | 17.7(22.86-12.85) | 1.336(0.955-1.719) |
| Marshall Islands | Both | 6.69(3.99-10.34) | 14.49(8.44-23.94) | 116.59 | 37.33(58.96-21.66) | 35.5(57.31-21.2) | -0.159(-0.297--0.021) |
| Mauritania | Both | 267.2(160.9-416.17) | 318.09(186.43-494.66) | 19.05 | 25.29(39.09-15.48) | 14.81(23.05-8.57) | -1.887(-2.012--1.762) |
| Mauritius | Both | 74.05(51.6-101.42) | 187.56(116.02-282.06) | 153.29 | 9.56(13.05-6.64) | 10.36(15.44-6.49) | 0.321(-0.191-0.836) |
| Mexico | Both | 6515.19(5666.23-7417.9) | 29913.86(23795.26-36912.61) | 359.14 | 14.66(16.67-12.72) | 25.13(30.94-20.1) | 1.923(1.473-2.376) |
| Micronesia (Federated States of) | Both | 19.52(11.56-30.56) | 29(15.08-48.54) | 48.57 | 37.58(59.2-22.31) | 35.51(57.72-19.09) | -0.194(-0.308--0.08) |
| Monaco | Both | 16.07(10.71-22.43) | 51.03(34.78-71.29) | 217.55 | 26.1(36.62-17.7) | 61.7(85.8-42.17) | 3.029(2.698-3.361) |
| Mongolia | Both | 4918.96(3028.93-7089.71) | 19966.19(12859.05-29861.97) | 305.90 | 451.12(646.64-280.55) | 787.04(1130.39-516.21) | 1.944(1.801-2.088) |
| Montenegro | Both | 413.14(298.95-534.35) | 591.86(417.04-793.71) | 43.26 | 64.48(82.77-46.92) | 60.16(80.01-42.77) | -0.206(-0.421-0.009) |
| Morocco | Both | 649.16(384.62-1014.67) | 1720.91(996.86-2690.65) | 165.10 | 4.74(7.36-2.82) | 5.53(8.55-3.31) | 0.526(0.275-0.778) |
| Mozambique | Both | 664.96(362.76-1288.72) | 2580.28(1487.95-3838.16) | 288.04 | 10.7(20.77-5.87) | 22.52(33.2-12.87) | 2.629(2.458-2.799) |
| Myanmar | Both | 2910.99(1779.92-4895.89) | 9739.59(6217.02-14337.99) | 234.58 | 12.19(20.3-7.52) | 20.29(29.63-13.05) | 1.848(1.152-2.549) |
| Namibia | Both | 72.49(33.61-169.97) | 327.85(205.58-492.42) | 352.27 | 9.44(22.12-4.39) | 21.42(31.74-13.61) | 2.902(2.582-3.222) |
| Nauru | Both | 1.69(0.94-2.73) | 1.93(1.02-3.3) | 14.20 | 35.73(55.64-20.74) | 34.1(54.78-19.56) | -0.161(-0.275--0.047) |
| Nepal | Both | 1122.72(747.04-1640.46) | 3108.85(1804.73-5272.27) | 176.90 | 11.47(16.32-7.74) | 13.63(23.06-8.02) | 0.614(0.502-0.725) |
| Netherlands | Both | 2519.97(1997.24-3034.28) | 7833.82(6068.21-9649.24) | 210.87 | 13.07(15.75-10.35) | 24.84(30.44-19.29) | 2.214(1.777-2.654) |
| New Zealand | Both | 870.86(750.37-988.53) | 2513.47(2179.73-2863.93) | 188.62 | 23.16(26.44-19.94) | 35.88(40.92-31.21) | 1.526(1.338-1.713) |
| Nicaragua | Both | 361.02(247.52-477.89) | 1293.21(873.41-1823.72) | 258.21 | 22.2(29.75-15.3) | 28.22(39.8-19.1) | 0.789(0.15-1.432) |
| Niger | Both | 81.45(47.81-124.55) | 206.53(119.57-321.04) | 153.57 | 2.66(4.02-1.61) | 2.45(3.76-1.43) | -0.29(-0.391--0.189) |
| Nigeria | Both | 7398.97(5221.55-10197.32) | 14803.22(10700.14-20103.54) | 100.07 | 16.08(22.2-11.35) | 17.12(22.72-12.51) | 0.209(0.072-0.347) |
| Niue | Both | 0.58(0.37-0.89) | 0.6(0.36-0.91) | 3.45 | 28.25(43.74-17.57) | 27.54(41.37-16.98) | -0.083(-0.144--0.022) |
| North Macedonia | Both | 1539.9(1118.66-1989.45) | 2461.03(1642.71-3469.75) | 59.82 | 78.42(100.87-57.79) | 73.53(103.56-49.55) | -0.193(-0.416-0.03) |
| Northern Mariana Islands | Both | 4.74(2.84-7.37) | 15.54(9.37-24.5) | 227.85 | 20.09(30.52-12.16) | 25.28(37.78-15.97) | 0.778(0.474-1.083) |
| Norway | Both | 710.88(623.23-801.66) | 1667.07(1385.03-1977.65) | 134.51 | 11.71(13.25-10.28) | 19.2(22.8-15.97) | 1.714(1.531-1.898) |
| Oman | Both | 78.39(43.61-124.66) | 247.4(148.91-392.7) | 215.60 | 10.52(16.8-5.91) | 11.98(18.73-7.4) | 0.428(0.202-0.655) |
| Pakistan | Both | 8729.21(5531.64-12276.34) | 18564.01(13697.97-25144.69) | 112.67 | 14.2(19.97-8.94) | 14.37(19.28-10.7) | 0.025(-0.096-0.148) |
| Palau | Both | 3.84(2.11-6.32) | 9.53(5.68-14.83) | 148.18 | 35.29(58.6-19.31) | 38.35(58.04-23.12) | 0.328(0.084-0.574) |
| Palestine | Both | 147.73(85.44-236.72) | 329.63(205.17-496.81) | 123.13 | 16.93(26.49-9.79) | 13.52(20.24-8.32) | -0.747(-1.102--0.391) |
| Panama | Both | 401.47(298.43-517.34) | 1006.4(670.23-1456.48) | 150.68 | 25.9(33.39-19.04) | 24.38(35.28-16.24) | -0.145(-0.584-0.297) |
| Papua New Guinea | Both | 78.39(45.74-122.79) | 221.94(131.53-359.63) | 183.12 | 4.21(6.57-2.52) | 4.93(8.02-2.97) | 0.55(0.468-0.632) |
| Paraguay | Both | 452.3(316.88-591.83) | 930.04(591.66-1344.43) | 105.62 | 19.85(25.92-13.89) | 16.58(24-10.57) | -0.599(-0.86--0.337) |
| Peru | Both | 5552.44(3880.8-7534.01) | 6502.09(4195.05-9378.68) | 17.10 | 45.68(62.01-32.02) | 20.42(29.36-13.12) | -2.632(-3.348--1.91) |
| Philippines | Both | 19990.62(14021.72-26451.06) | 36182.02(26606.12-47479.94) | 80.99 | 57.91(76.5-39.92) | 41.4(54.03-30.6) | -1.139(-1.281--0.998) |
| Poland | Both | 26806.97(23620.1-30085.16) | 13769.06(11081.79-17062.18) | -48.64 | 60.71(68.11-53.62) | 20.28(25.15-16.32) | -3.655(-4.301--3.005) |
| Portugal | Both | 2420.65(1840.26-3000) | 9062.15(6750.12-11472.97) | 274.37 | 17.65(21.7-13.46) | 45.21(57.71-33.38) | 3.323(2.984-3.663) |
| Puerto Rico | Both | 1989.32(1469.66-2534.18) | 1422.93(911.8-2063.15) | -28.47 | 54.32(69.24-40.08) | 21.94(31.82-14.22) | -3.064(-3.657--2.468) |
| Qatar | Both | 41.69(23.49-65.63) | 288.12(156.8-470.15) | 591.10 | 40.07(64.74-22.99) | 33.78(54.43-18.73) | -0.587(-1.331-0.162) |
| Republic of Korea | Both | 16125.61(9917.59-23961.3) | 65166.94(45693.4-88918.12) | 304.12 | 45.8(67.78-28.64) | 72.49(99.34-51.41) | 1.609(1.243-1.977) |
| Republic of Moldova | Both | 968.8(797.62-1130.33) | 1549.39(1214.5-1951.88) | 59.93 | 21.02(24.42-17.43) | 27.05(33.9-21.12) | 0.738(-0.95-2.454) |
| Romania | Both | 5053.67(3739.01-6385.73) | 11503.17(8112.62-15137.55) | 127.62 | 17.63(22.21-13.26) | 33.96(44.85-23.85) | 2.353(1.614-3.097) |
| Russian Federation | Both | 27258.81(23494.92-31342.04) | 62716.47(49284.81-80078.81) | 130.08 | 14.74(16.93-12.7) | 27.6(35.28-21.58) | 2.171(1.504-2.842) |
| Rwanda | Both | 1325.65(820.06-2051.38) | 2369.26(1551.38-3523.47) | 78.72 | 42.19(64.49-26.38) | 35.47(51.96-23.59) | -0.596(-0.842--0.35) |
| Saint Kitts and Nevis | Both | 36.09(26.19-47.39) | 21.49(14.83-30.26) | -40.45 | 98.2(129.25-70.71) | 30.33(42.14-21.17) | -4.051(-4.828--3.269) |
| Saint Lucia | Both | 49.25(37.05-62.9) | 43.88(31.43-58.07) | -10.90 | 55.11(70.59-41.41) | 20.2(26.6-14.63) | -3.359(-3.876--2.839) |
| Saint Vincent and the Grenadines | Both | 41.59(30.17-54.01) | 38.84(28.19-50.69) | -6.61 | 57.33(74.07-41.76) | 28.14(36.77-20.49) | -2.373(-3.002--1.74) |
| Samoa | Both | 20.29(11.78-32.37) | 28.38(17.31-43.24) | 39.87 | 21.66(34.04-12.6) | 18.06(27.2-11.21) | -0.635(-0.714--0.556) |
| San Marino | Both | 5.61(3.99-7.64) | 13.46(7.63-21.41) | 139.93 | 17.04(23.07-12.14) | 23.68(38.4-13.29) | 1.168(1.079-1.257) |
| Sao Tome and Principe | Both | 7.26(4.59-10.58) | 17.12(10.09-27.07) | 135.81 | 10.85(15.85-6.89) | 14.43(22.75-8.51) | 1.024(0.724-1.325) |
| Saudi Arabia | Both | 650.83(369.08-1034.96) | 1569.18(879.65-2565.9) | 141.10 | 10.81(17.68-6.16) | 9.03(14.78-5.29) | -0.619(-0.85--0.388) |
| Senegal | Both | 253.95(157.78-375.04) | 535.08(321.09-809.48) | 110.70 | 7.51(11.13-4.79) | 6.83(10.3-4.13) | -0.295(-0.689-0.101) |
| Serbia | Both | 6326.66(4459.44-8235.97) | 7348.47(4896.89-10257.35) | 16.15 | 52.7(67.86-37.91) | 46.93(65.85-31.56) | -0.36(-0.55--0.171) |
| Seychelles | Both | 23.77(15.53-34.32) | 42.69(27.27-60.74) | 79.60 | 42.47(61.32-27.72) | 36.24(51.23-23.79) | -0.536(-0.787--0.284) |
| Sierra Leone | Both | 550.92(346.38-860.39) | 832.83(505.91-1270.25) | 51.17 | 27.69(42.94-17.64) | 21.63(32.93-12.89) | -0.834(-1.073--0.594) |
| Singapore | Both | 352.06(232.88-506.46) | 1168.77(754.22-1732.67) | 231.98 | 14.86(21.37-9.84) | 14.62(21.77-9.44) | -0.059(-0.268-0.15) |
| Slovakia | Both | 3074.58(2404.77-3716.1) | 3294.2(2242.75-4567.98) | 7.14 | 51.44(62.25-39.84) | 36.12(49.78-24.76) | -1.306(-1.572--1.04) |
| Slovenia | Both | 693.36(468.63-959.05) | 1886.97(1249.52-2689.68) | 172.15 | 28.28(39.32-19.05) | 47.3(67.35-31.04) | 1.828(1.095-2.567) |
| Solomon Islands | Both | 30.96(18.22-49.89) | 58.81(35.55-89.81) | 89.95 | 19.61(31.64-11.61) | 16.5(24.79-10.01) | -0.623(-1.031--0.214) |
| Somalia | Both | 560.53(284.49-1215.18) | 1306.79(695.5-2600.16) | 133.13 | 20.07(43.66-10.36) | 17.85(33.94-9.46) | -0.375(-0.504--0.245) |
| South Africa | Both | 7726.78(4615.23-14648.61) | 15692.1(12694.36-19136.9) | 103.09 | 32.93(63.31-19.54) | 31.93(38.6-25.9) | -0.059(-0.623-0.509) |
| South Sudan | Both | 460.22(222.28-1030.59) | 624.95(288.06-1315.93) | 35.79 | 18.32(41.27-8.9) | 15.47(31.94-7.25) | -0.585(-0.73--0.44) |
| Spain | Both | 15460.62(10964.1-20102.84) | 28227.52(19660.44-37630.97) | 82.58 | 28.8(37.39-20.62) | 34.05(45.49-24.01) | 0.562(0.414-0.71) |
| Sri Lanka | Both | 1128.3(754.65-1600.38) | 4309.87(2648.66-6722.77) | 281.98 | 10.06(14.12-6.69) | 16.09(25.03-9.9) | 1.73(1.201-2.261) |
| Sudan | Both | 1050.12(543.21-2009.61) | 2234.51(1172.59-4202.73) | 112.79 | 11.04(20.84-5.62) | 12.22(22.95-6.34) | 0.314(0.17-0.459) |
| Suriname | Both | 146.71(104.65-192.11) | 139.71(97.21-198.54) | -4.77 | 54.68(71.69-38.86) | 22.6(31.79-15.83) | -2.985(-3.578--2.389) |
| Sweden | Both | 3197.17(2778.27-3623.76) | 5665.61(4887.61-6459.72) | 77.21 | 23.53(26.67-20.58) | 31.46(35.84-27.12) | 1.003(0.753-1.254) |
| Switzerland | Both | 2360.21(1807.8-2839.29) | 6374.54(4714.79-8150.99) | 170.08 | 24.3(29.4-18.67) | 40.48(51.96-30.12) | 1.718(1.437-2) |
| Syrian Arab Republic | Both | 593.08(355.98-891.91) | 1183.81(682.83-1920.23) | 99.60 | 10.96(16.75-6.62) | 9.09(14.52-5.25) | -0.723(-0.921--0.525) |
| Taiwan (Province of China) | Both | 7735.47(4985.05-10915.82) | 7027.18(4286.57-10952.94) | -9.16 | 42.77(60.3-27.7) | 18.1(27.83-10.97) | -3.112(-3.866--2.353) |
| Tajikistan | Both | 149.34(99.89-208.61) | 1083.13(704.6-1603.33) | 625.28 | 5.29(7.38-3.52) | 20.1(28.63-13.16) | 4.706(4.463-4.95) |
| Thailand | Both | 54654.66(37669.58-76196.04) | 186006.53(118916.66-282591.84) | 240.33 | 138.22(189.96-95.67) | 177.93(266.96-114.55) | 0.765(0.504-1.027) |
| Timor-Leste | Both | 113.46(61.97-187.55) | 271.35(139.53-443.21) | 139.16 | 34.95(56.86-19.36) | 31.53(50.89-16.48) | -0.325(-0.514--0.136) |
| Togo | Both | 382.87(235.74-598.62) | 918.21(553.95-1414.91) | 139.82 | 28.09(43.64-17.09) | 22.45(33.74-13.88) | -0.794(-0.881--0.706) |
| Tokelau | Both | 0.33(0.19-0.51) | 0.32(0.17-0.51) | -3.03 | 24.85(38.89-14.45) | 23.27(37.1-12.92) | -0.231(-0.3--0.161) |
| Tonga | Both | 45.23(25.3-71.79) | 70.72(41.15-110.62) | 56.36 | 75.52(119.33-42.15) | 87.29(136.03-50.95) | 0.536(0.4-0.672) |
| Trinidad and Tobago | Both | 457.12(338.89-593.97) | 393.64(253.91-569.18) | -13.89 | 52.7(68.71-38.5) | 20.81(30.05-13.43) | -3.19(-3.62--2.759) |
| Tunisia | Both | 251.68(138.67-431.18) | 627.69(315.45-1146.71) | 149.40 | 4.77(8.18-2.65) | 4.84(8.87-2.44) | 0.06(-0.111-0.231) |
| Turkey | Both | 4041.67(2541.59-6056.77) | 7579.38(4730.33-11488.26) | 87.53 | 11.07(16.4-7.1) | 8.49(12.92-5.26) | -0.929(-1.142--0.715) |
| Turkmenistan | Both | 120.91(84.94-162.6) | 2035.97(1330.17-2899.26) | 1583.87 | 6.16(8.31-4.32) | 46.07(65.17-30.59) | 7.182(6.234-8.138) |
| Tuvalu | Both | 2.36(1.4-3.67) | 3.02(1.74-4.85) | 27.97 | 31.41(48.46-19.01) | 27.8(44.52-16.11) | -0.416(-0.477--0.355) |
| Uganda | Both | 2823.93(1846.75-3947.31) | 8486.98(5605.19-12353.23) | 200.54 | 40.19(55.6-26.41) | 52.58(74.9-35.48) | 0.92(0.643-1.197) |
| Ukraine | Both | 4887.31(4188.58-5583.57) | 16516.46(13130.26-20527.07) | 237.95 | 6.73(7.66-5.78) | 22.83(28.43-18.11) | 4.177(3.729-4.626) |
| United Arab Emirates | Both | 71.09(30.08-144.7) | 750.23(236.76-2006.72) | 955.32 | 13.21(29.41-5.25) | 13.92(37.6-4.5) | 0.15(-0.241-0.543) |
| United Kingdom | Both | 13316.26(11722.93-14873.8) | 36849.73(32310.5-41415.8) | 176.73 | 15.71(17.51-13.8) | 32.09(36.19-28.2) | 2.473(2.294-2.653) |
| United Republic of Tanzania | Both | 1533.28(985.75-2222.77) | 3921.16(2531.21-5795.65) | 155.74 | 13.29(19.34-8.64) | 15.34(22.52-9.94) | 0.515(0.107-0.924) |
| United States of America | Both | 44370.63(39322.77-49324.34) | 177320.58(144413.59-211284.47) | 299.64 | 14.7(16.39-13.01) | 33.87(40.46-27.61) | 2.954(2.866-3.042) |
| United States Virgin Islands | Both | 28.69(20.25-39.2) | 39.39(27.78-54.49) | 37.30 | 31.56(42.63-22.29) | 20.96(29.01-14.64) | -1.307(-1.66--0.953) |
| Uruguay | Both | 368.09(256.47-504.38) | 824.99(584.36-1079.48) | 124.13 | 9.52(13.05-6.67) | 16.66(21.83-11.97) | 2.015(1.533-2.5) |
| Uzbekistan | Both | 580.86(398.89-767.68) | 10853.97(7187.48-15146.63) | 1768.60 | 5.07(6.73-3.48) | 45.24(62.14-30.72) | 7.908(7.48-8.338) |
| Vanuatu | Both | 21.4(10.97-36.98) | 59.26(31.91-97.12) | 176.92 | 29.53(51.12-14.96) | 31.35(51.44-16.85) | 0.216(-0.011-0.444) |
| Venezuela (Bolivarian Republic of) | Both | 5451.59(4032.77-6883.2) | 5660.56(3719.36-8158.41) | 3.83 | 54.26(68.27-40.05) | 19.06(27.37-12.57) | -3.729(-4.682--2.767) |
| Viet Nam | Both | 6942.65(4269.43-10860.59) | 13941.32(8545.66-21084.44) | 100.81 | 16.83(26.24-10.4) | 14.74(21.83-9.04) | -0.485(-0.655--0.314) |
| Yemen | Both | 250.69(127.53-458.75) | 712.94(405.62-1206.38) | 184.39 | 4.88(8.91-2.59) | 5.27(8.98-3) | 0.249(0.159-0.339) |
| Zambia | Both | 456.34(271.32-806.78) | 1325.62(839.55-1891.23) | 190.49 | 15.4(27.49-9.04) | 18.77(26.31-12.12) | 0.657(0.477-0.838) |
| Zimbabwe | Both | 2444.42(1404.43-4723.32) | 4586.91(2697.94-7746.11) | 87.65 | 53.31(102.76-30.57) | 56.52(94.37-33.58) | 0.309(0.03-0.589) |
| Afghanistan | Female | 548.51(298.37-895.51) | 1058.08(579.6-1733.96) | 92.90 | 14.31(23.07-7.95) | 13.3(22.27-7.54) | -0.206(-0.277--0.136) |
| Albania | Female | 313.73(212.22-446.69) | 317.86(184.48-496.49) | 1.32 | 28.68(40.76-19.35) | 14.5(22.7-8.48) | -2.394(-2.721--2.065) |
| Algeria | Female | 98.95(56.79-154.34) | 298.7(173.2-493.85) | 201.87 | 1.49(2.32-0.87) | 1.7(2.73-0.99) | 0.494(0.226-0.762) |
| American Samoa | Female | 0.99(0.59-1.54) | 2.21(1.3-3.5) | 123.23 | 8.09(12.54-4.88) | 8.39(13.12-5.03) | 0.012(-0.272-0.297) |
| Andorra | Female | 7.66(4.65-12.45) | 18.23(10.39-28.79) | 137.99 | 27.49(44.24-16.72) | 27.35(42.45-15.35) | -0.021(-0.174-0.132) |
| Angola | Female | 95.35(52.54-157.72) | 350.27(198.43-575.16) | 267.35 | 4.04(6.62-2.19) | 4.65(7.45-2.72) | 0.45(0.286-0.614) |
| Antigua and Barbuda | Female | 6.61(4.45-9.17) | 5.87(3.78-8.34) | -11.20 | 23.23(32.24-15.62) | 10.77(15.34-7) | -2.587(-3.17--2.001) |
| Argentina | Female | 869.42(578.85-1231.02) | 1380.74(933.09-1945.12) | 58.81 | 4.84(6.87-3.23) | 4.73(6.67-3.19) | -0.067(-0.252-0.119) |
| Armenia | Female | 46.19(30.44-66.36) | 481.17(312.68-692.65) | 941.72 | 2.98(4.24-1.95) | 20.3(29.1-13.25) | 6.757(4.69-8.866) |
| Australia | Female | 583.02(405.79-786.99) | 2416.78(1631.83-3298.08) | 314.53 | 5.7(7.63-3.97) | 12.09(16.41-8.19) | 2.627(2.434-2.821) |
| Austria | Female | 723.27(489.35-976.64) | 1223.63(837.58-1660.85) | 69.18 | 10.52(13.97-7.39) | 14.26(19.27-9.86) | 1.047(0.685-1.411) |
| Azerbaijan | Female | 75.02(49.63-107.14) | 608.46(354.73-957.09) | 711.06 | 2.57(3.7-1.68) | 11.46(17.89-6.68) | 5.34(4.687-5.997) |
| Bahamas | Female | 29.77(20.84-40.11) | 25.74(16.92-37.11) | -13.54 | 33.28(44.94-22.92) | 11.67(16.74-7.74) | -3.531(-3.997--3.062) |
| Bahrain | Female | 4.73(2.88-7.28) | 16.13(9.29-25.13) | 241.01 | 5.83(9.12-3.53) | 4.34(6.84-2.44) | -0.988(-1.468--0.505) |
| Bangladesh | Female | 979.14(555.67-1608.12) | 2579.85(1501.31-4167.38) | 163.48 | 3.91(6.26-2.23) | 3.82(6.11-2.25) | -0.055(-0.301-0.192) |
| Barbados | Female | 17.69(11.4-25.06) | 33.54(22.19-47.76) | 89.60 | 11.17(15.65-7.23) | 12.7(18.29-8.39) | 0.383(-0.007-0.774) |
| Belarus | Female | 448.64(310.96-599.84) | 995.67(620.84-1438.36) | 121.93 | 5.49(7.27-3.87) | 10.76(15.49-6.84) | 2.325(1.382-3.277) |
| Belgium | Female | 808.58(551.65-1101.96) | 1078.23(724.65-1524.03) | 33.35 | 9.57(12.89-6.7) | 9.82(13.66-6.66) | 0.085(-0.32-0.492) |
| Belize | Female | 15.08(10.28-20.73) | 18.95(12.67-26.41) | 25.66 | 32.56(44.77-22) | 13.15(18.3-8.86) | -3.143(-3.616--2.668) |
| Benin | Female | 128.84(74.77-195.85) | 289.47(172.33-460.91) | 124.67 | 11.92(18.43-6.81) | 10.41(16.47-6.18) | -0.485(-0.575--0.395) |
| Bermuda | Female | 11.58(7.93-15.87) | 4.49(2.95-6.59) | -61.23 | 32.61(44.75-22.31) | 6.54(9.52-4.26) | -5.408(-5.998--4.815) |
| Bhutan | Female | 5.76(2.95-9.52) | 14.55(8-26.39) | 152.60 | 4.21(7.04-2.22) | 5.12(9.22-2.85) | 0.647(0.551-0.743) |
| Bolivia (Plurinational State of) | Female | 506.39(278.99-894.18) | 1282.43(741.27-2133.07) | 153.25 | 29.19(51.39-15.95) | 27.48(45.25-16.02) | -0.226(-0.336--0.117) |
| Bosnia and Herzegovina | Female | 366.16(237.32-522.26) | 950.99(592.19-1430.54) | 159.72 | 15.37(21.76-10.07) | 29.14(43.08-18.43) | 2.155(1.711-2.601) |
| Botswana | Female | 8.76(4.24-15.89) | 26.3(13.85-43.51) | 200.23 | 2.54(4.57-1.23) | 3.02(4.97-1.63) | 0.593(0.318-0.869) |
| Brazil | Female | 4065.67(3439.16-4753.12) | 10248.72(8640.4-11986.04) | 152.08 | 8.04(9.32-6.8) | 7.83(9.14-6.61) | -0.078(-0.206-0.051) |
| Brunei Darussalam | Female | 4.49(2.59-7.3) | 17.16(10.07-27.32) | 282.18 | 8.78(14.04-5.11) | 9.86(15.61-5.86) | 0.442(0.167-0.718) |
| Bulgaria | Female | 2107.2(1439.78-2849.55) | 1446.94(941.21-2074.31) | -31.33 | 30.68(41.36-21.22) | 19.72(28.59-12.95) | -1.631(-2.229--1.03) |
| Burkina Faso | Female | 157.11(91.42-244.1) | 333.15(195.91-512.71) | 112.05 | 6.2(9.53-3.68) | 5.78(8.67-3.38) | -0.18(-0.471-0.111) |
| Burundi | Female | 233.58(133.45-389.07) | 285.77(165.88-456.99) | 22.34 | 17.05(28.01-9.8) | 11.59(18.42-6.92) | -1.33(-1.577--1.082) |
| Cabo Verde | Female | 2.73(1.69-4.18) | 50.91(31.4-77.06) | 1764.84 | 2.13(3.29-1.3) | 22.02(33.66-13.34) | 8.476(7.828-9.128) |
| Cambodia | Female | 516.53(301.38-822.53) | 1526.77(896.53-2447.88) | 195.58 | 17.39(27.82-10.17) | 20.42(32.73-12.09) | 0.55(0.381-0.72) |
| Cameroon | Female | 33(19.1-51.79) | 87.64(50.5-144.95) | 165.58 | 1.34(2.09-0.78) | 1.3(2.11-0.73) | -0.118(-0.221--0.014) |
| Canada | Female | 1476.58(1107.71-1906.73) | 5488.68(3732.52-7309.52) | 271.72 | 8.46(10.94-6.31) | 16.24(21.45-11.5) | 2.341(1.854-2.83) |
| Central African Republic | Female | 46.13(24.32-84.74) | 77.53(36.21-150.09) | 68.07 | 6.29(11.52-3.33) | 5.58(10.77-2.65) | -0.414(-0.638--0.19) |
| Chad | Female | 156.74(95.83-240.22) | 282.61(163.84-457.16) | 80.30 | 10.3(15.83-6.21) | 9.99(16.22-5.89) | -0.081(-0.288-0.126) |
| Chile | Female | 391.36(262.77-546.74) | 1162.46(772.18-1651.7) | 197.03 | 6.97(9.75-4.66) | 8.96(12.69-6.02) | 0.863(0.547-1.18) |
| China | Female | 155800.93(109892.87-212856.43) | 120749.29(83567.33-163136.87) | -22.50 | 33.67(46-23.8) | 11.22(15.08-7.86) | -3.762(-4.196--3.326) |
| Colombia | Female | 1380.05(918.11-1919.51) | 2475.82(1551-3709.09) | 79.40 | 14.59(20.23-9.66) | 8.71(13.08-5.43) | -1.817(-2.252--1.379) |
| Comoros | Female | 10.28(4.61-17.16) | 21.76(12.43-34.93) | 111.67 | 8.55(14.38-4.06) | 7.91(12.74-4.54) | -0.272(-0.736-0.194) |
| Congo | Female | 46.77(26.95-76.1) | 97.38(50.81-159.27) | 108.21 | 7.06(11.39-4.17) | 6.03(9.91-3.2) | -0.543(-0.758--0.328) |
| Cook Islands | Female | 1.29(0.75-2.04) | 1.85(1.12-2.87) | 43.41 | 20.28(32.36-11.83) | 14.75(23.05-8.97) | -1.083(-1.553--0.611) |
| Costa Rica | Female | 205.69(138.4-284.01) | 473.35(283.1-715.95) | 130.13 | 22.43(31.45-15.03) | 17.18(25.83-10.41) | -0.995(-1.463--0.524) |
| Croatia | Female | 626.39(437.24-819.23) | 604.63(395.2-860.87) | -3.47 | 16.3(21.23-11.57) | 12.91(18.44-8.34) | -0.89(-1.432--0.344) |
| Cuba | Female | 1591.26(1123.68-2145.11) | 722.16(466.52-1046.88) | -54.62 | 30.37(40.92-21.3) | 7.38(10.81-4.78) | -4.847(-5.264--4.428) |
| Cyprus | Female | 35.3(22.21-52.51) | 86.58(55.02-124.81) | 145.27 | 8.08(11.66-5.22) | 8.58(12.29-5.45) | 0.164(-0.16-0.49) |
| Czechia | Female | 1639.52(1159.78-2123.74) | 1408.62(950.09-1967.96) | -14.08 | 20.65(26.74-14.77) | 12.93(18.23-8.72) | -1.659(-1.987--1.33) |
| C么te d'Ivoire | Female | 224.72(136.18-344.57) | 525.42(299.87-839.81) | 133.81 | 10.8(16.66-6.55) | 9.42(14.81-5.45) | -0.442(-0.626--0.257) |
| Democratic People's Republic of Korea | Female | 1533.6(855.84-2565.88) | 1733.17(942.67-2807.74) | 13.01 | 14.68(24.25-8.29) | 9.51(15.53-5.16) | -1.503(-1.655--1.35) |
| Democratic Republic of the Congo | Female | 441.4(261.45-700.14) | 944.56(520.89-1580.37) | 113.99 | 4.37(6.79-2.64) | 4.16(6.81-2.34) | -0.195(-0.262--0.128) |
| Denmark | Female | 270.4(184.3-369.99) | 501.86(334.71-704.74) | 85.60 | 6.51(8.84-4.49) | 9.16(12.71-6.25) | 1.2(0.765-1.637) |
| Djibouti | Female | 8.33(4.83-13.24) | 30.07(16.31-51.79) | 260.98 | 10.51(16.55-6.34) | 9.55(15.93-5.36) | -0.313(-0.672-0.047) |
| Dominica | Female | 15.08(10.26-20.94) | 5.66(3.75-8.18) | -62.47 | 38.29(53.69-25.83) | 12.77(18.26-8.46) | -3.851(-4.339--3.361) |
| Dominican Republic | Female | 375.53(252.09-516.25) | 922.45(545-1441.57) | 145.64 | 18.64(25.73-12.54) | 18.82(29.59-11.23) | 0.029(-0.2-0.258) |
| Ecuador | Female | 407.41(276.12-578.21) | 1410.34(916.51-2088.37) | 246.17 | 14.92(21.19-10.04) | 17.89(26.39-11.67) | 0.568(0.075-1.064) |
| Egypt | Female | 1222.33(691.63-1915.28) | 3675.15(1961.24-6510.42) | 200.67 | 7.72(12.29-4.31) | 11.38(20.04-6.17) | 1.373(0.922-1.826) |
| El Salvador | Female | 220.23(143.94-310.99) | 179.57(108.69-274.98) | -18.46 | 13.6(19.16-8.74) | 5.38(8.24-3.23) | -3.19(-3.687--2.692) |
| Equatorial Guinea | Female | 6.52(3.39-10.89) | 16.47(7.85-30.02) | 152.61 | 5.21(8.69-2.76) | 5.06(9.25-2.47) | -0.045(-0.39-0.3) |
| Eritrea | Female | 72.62(31.89-139.31) | 196.24(115.1-327.5) | 170.23 | 11.26(21.09-5.09) | 11.66(18.9-7.01) | 0.155(0.05-0.26) |
| Estonia | Female | 108.68(73.62-146.88) | 227.72(143.38-322.95) | 109.53 | 8.52(11.49-5.79) | 15.65(22.13-10) | 2.261(1.517-3.01) |
| Eswatini | Female | 22.64(12.62-36.16) | 49.21(24.14-94.41) | 117.36 | 12.39(19.66-6.92) | 13.11(24.74-6.5) | 0.143(-0.008-0.293) |
| Ethiopia | Female | 824.26(461.26-1379.74) | 1187.09(847.43-1678.67) | 44.02 | 7.39(12.14-4.26) | 5.3(7.36-3.81) | -1.156(-1.337--0.975) |
| Fiji | Female | 21.3(12.41-33.91) | 40.69(22.85-67.16) | 91.03 | 10.1(15.78-5.87) | 9.45(15.41-5.34) | -0.187(-0.435-0.063) |
| Finland | Female | 360.69(232.64-504.04) | 622.37(408.31-877.8) | 72.55 | 8.7(12.12-5.79) | 10.39(14.57-7.03) | 0.576(0.334-0.818) |
| France | Female | 3008.2(2068.06-4098.23) | 6355.51(4225.32-9024.78) | 111.27 | 6.86(9.34-4.75) | 9.78(13.89-6.48) | 1.231(1.02-1.442) |
| Gabon | Female | 25.02(13.68-40.7) | 35.34(17.32-59.49) | 41.25 | 7.76(12.59-4.26) | 5.64(9.42-2.83) | -1.057(-1.192--0.921) |
| Gambia | Female | 64.36(38.54-99.35) | 274.39(154.86-428.19) | 326.34 | 36.64(56.83-21.63) | 52.85(82.26-29.88) | 1.363(0.164-2.575) |
| Georgia | Female | 126.63(83.43-180.19) | 310.7(200.62-440.21) | 145.36 | 3.37(4.76-2.21) | 9.54(13.46-6.28) | 3.716(2.788-4.651) |
| Germany | Female | 5383.25(3877.65-7101.25) | 9193.93(6386.89-12543.04) | 70.79 | 7.53(9.86-5.5) | 10.25(13.82-7.21) | 1.157(0.967-1.348) |
| Ghana | Female | 239.42(145.39-369.85) | 522.53(314.43-807.08) | 118.25 | 6.71(10.45-4) | 5.36(8.31-3.21) | -0.764(-0.98--0.546) |
| Greece | Female | 638(442.68-863.53) | 934.63(642.49-1285.26) | 46.49 | 7.63(10.22-5.39) | 7.89(10.77-5.41) | 0.092(-0.111-0.294) |
| Greenland | Female | 2.59(1.71-3.7) | 6.01(3.7-9.49) | 132.05 | 14(19.91-9.27) | 17.95(27.66-11.22) | 0.962(0.562-1.364) |
| Grenada | Female | 16.6(11.45-22.43) | 8.16(5.7-11.22) | -50.84 | 43.44(59.11-29.88) | 13.85(19-9.64) | -3.891(-4.301--3.48) |
| Guam | Female | 1.57(0.97-2.38) | 2.84(1.69-4.47) | 80.89 | 3.92(5.98-2.47) | 2.95(4.57-1.78) | -0.969(-1.969-0.04) |
| Guatemala | Female | 696.75(450.88-995.86) | 987.96(596.06-1505.28) | 41.80 | 34.54(49-22.73) | 15.87(23.95-9.37) | -2.653(-3.48--1.819) |
| Guinea | Female | 625.93(389.34-967.94) | 1028.29(592.23-1627.18) | 64.28 | 35.68(55.55-21.97) | 35.18(55.82-20.06) | 0.016(-0.039-0.071) |
| Guinea-Bissau | Female | 30.9(17.12-53.08) | 54.99(30.15-92.66) | 77.96 | 13.36(22.61-7.54) | 12.58(21.11-6.96) | -0.267(-0.423--0.11) |
| Guyana | Female | 79.89(54.47-109.24) | 50.91(31.81-75.82) | -36.27 | 38.46(52.62-26.02) | 14.48(21.21-9.22) | -3.26(-3.845--2.672) |
| Haiti | Female | 600.38(297.65-1099.06) | 749.07(353.55-1415.99) | 24.77 | 33.86(61.48-16.89) | 18.95(35.02-9.09) | -1.997(-2.189--1.804) |
| Honduras | Female | 296.14(94.39-531.17) | 1216.33(411.27-2417.94) | 310.73 | 26.48(48.43-8.15) | 36.59(72.85-12.24) | 1.146(0.62-1.675) |
| Hungary | Female | 2801.39(2000.95-3634.4) | 1097.48(719.85-1572.18) | -60.82 | 33.06(42.71-24.11) | 10.54(15.01-6.87) | -3.953(-4.705--3.195) |
| Iceland | Female | 5.81(3.69-8.41) | 14.15(8.99-20.37) | 143.55 | 3.99(5.74-2.54) | 5.33(7.68-3.43) | 1.006(0.723-1.289) |
| India | Female | 15657.12(11639.97-20397.24) | 46637.41(32863.01-64816.06) | 197.87 | 6.73(8.86-5) | 7.69(10.63-5.44) | 0.454(0.287-0.621) |
| Indonesia | Female | 2440.59(1796.92-3196.87) | 3151.7(2249.43-4336.02) | 29.14 | 4.26(5.56-3.16) | 2.67(3.62-1.95) | -1.597(-1.783--1.41) |
| Iran (Islamic Republic of) | Female | 643.68(421.87-957.58) | 1281.82(941.23-1655.12) | 99.14 | 5.1(7.75-3.35) | 3.45(4.47-2.53) | -1.477(-2.076--0.874) |
| Iraq | Female | 200.23(114.46-314.1) | 732.09(412.44-1179.73) | 265.62 | 4.81(7.59-2.75) | 5.71(9.29-3.28) | 0.551(0.2-0.903) |
| Ireland | Female | 102.92(69.11-148.01) | 368.25(238.84-518.12) | 257.80 | 4.69(6.58-3.17) | 9.72(13.62-6.36) | 2.447(2.101-2.795) |
| Israel | Female | 133.26(82.93-200.46) | 270.63(169.08-397.39) | 103.08 | 4.99(7.48-3.19) | 4.52(6.58-2.85) | -0.381(-0.642--0.119) |
| Italy | Female | 5474.55(4504.17-6514.99) | 4532.51(3694.54-5447.4) | -17.21 | 11.17(13.33-9.25) | 6.53(7.87-5.37) | -1.94(-2.214--1.665) |
| Jamaica | Female | 137.45(95.54-186.18) | 155.97(99.37-236) | 13.47 | 14.95(20.25-10.36) | 10.33(15.52-6.57) | -1.374(-2.657--0.074) |
| Japan | Female | 9903.95(7553.76-13432.74) | 12068(8928.77-17147.44) | 21.85 | 10.39(14.28-7.9) | 6.66(9.46-4.92) | -1.533(-1.795--1.269) |
| Jordan | Female | 20.39(11.74-32.18) | 53.81(29.86-88.27) | 163.90 | 3.02(4.85-1.7) | 1.67(2.73-0.93) | -2.002(-2.277--1.725) |
| Kazakhstan | Female | 875.94(593.13-1221.35) | 2199.88(1420.28-3121.57) | 151.15 | 11.18(15.58-7.61) | 21.04(29.51-13.64) | 2.094(1.608-2.582) |
| Kenya | Female | 485.26(344.71-837.21) | 1477.05(940.49-2235.32) | 204.38 | 10.54(17.86-7.65) | 11.39(16.92-7.41) | 0.277(0.108-0.446) |
| Kiribati | Female | 5.07(3.03-8) | 8.95(4.95-14.58) | 76.53 | 22.18(34.72-13.38) | 20.04(31.94-11.09) | -0.352(-0.497--0.207) |
| Kuwait | Female | 4.92(3.13-7.35) | 15.33(8.58-24.16) | 211.59 | 2.01(3.14-1.24) | 1.4(2.23-0.79) | -1.343(-2.211--0.467) |
| Kyrgyzstan | Female | 65.71(43.11-93.57) | 216.58(140.82-311.18) | 229.60 | 3.64(5.14-2.38) | 8.24(11.94-5.35) | 2.937(2.376-3.5) |
| Lao People's Democratic Republic | Female | 249.46(119.95-486.55) | 340.59(201.23-534.83) | 36.53 | 20.95(39.69-10.13) | 13.96(21.8-8.12) | -1.401(-1.51--1.292) |
| Latvia | Female | 152.02(105.57-206.01) | 261.87(169.02-388.03) | 72.26 | 6.82(9.24-4.8) | 12.02(17.9-7.64) | 2.013(1.426-2.603) |
| Lebanon | Female | 63.31(37.16-99.28) | 100.07(53.67-170.79) | 58.06 | 5.34(8.44-3.15) | 3.52(5.94-1.88) | -1.454(-1.642--1.266) |
| Lesotho | Female | 64.37(35.42-112.56) | 133.07(63.91-244.27) | 106.73 | 11.21(19.51-6.12) | 16.8(30.96-8.26) | 1.363(1.104-1.623) |
| Liberia | Female | 69.13(42.85-109.11) | 113.36(64.34-184.39) | 63.98 | 12.65(19.77-7.82) | 10.29(16.43-5.97) | -0.768(-0.984--0.551) |
| Libya | Female | 56.04(32.16-90.3) | 139.53(76.42-232.38) | 148.98 | 6.29(10.13-3.55) | 5.36(8.9-2.93) | -0.493(-0.934--0.051) |
| Lithuania | Female | 180.9(124.43-240.04) | 385.95(253.53-543.94) | 113.35 | 6.68(8.84-4.58) | 12.53(17.48-8.14) | 2.234(1.645-2.827) |
| Luxembourg | Female | 31.12(21.11-42.2) | 57.89(36.75-82.63) | 86.02 | 10.12(13.49-6.93) | 11.75(17.03-7.54) | 0.476(0.272-0.68) |
| Madagascar | Female | 292.21(177.4-459.94) | 591.32(338.66-987.55) | 102.36 | 10.4(15.94-6.41) | 9.09(14.66-5.37) | -0.453(-0.621--0.285) |
| Malawi | Female | 219.29(131.99-327.16) | 287.51(169.67-444.88) | 31.11 | 9.7(14.46-5.81) | 6.77(10.24-4.03) | -1.248(-1.614--0.88) |
| Malaysia | Female | 357.34(220.56-546.09) | 947.22(545.68-1530.17) | 165.08 | 7.35(11.23-4.54) | 6.81(10.86-3.92) | -0.228(-0.385--0.072) |
| Maldives | Female | 2.8(1.23-5.94) | 5.85(3.53-8.98) | 108.93 | 6.99(14.41-3.22) | 4.37(6.67-2.61) | -1.999(-2.212--1.785) |
| Mali | Female | 261.44(158.3-403.98) | 551.82(309.33-913.19) | 111.07 | 11.31(17.53-6.88) | 11.81(19.33-6.75) | 0.172(0.065-0.28) |
| Malta | Female | 9.62(6.25-14.04) | 23.57(14.8-34.93) | 145.01 | 4.04(5.84-2.65) | 5.19(7.37-3.38) | 0.871(0.624-1.119) |
| Marshall Islands | Female | 1.43(0.77-2.54) | 2.8(1.33-5.34) | 95.80 | 16.13(28.9-8.49) | 14.16(26.48-7.16) | -0.457(-0.526--0.387) |
| Mauritania | Female | 57.78(34.64-92.46) | 79.92(45.82-127.77) | 38.32 | 10.63(16.85-6.34) | 7.33(11.68-4.24) | -1.303(-1.441--1.164) |
| Mauritius | Female | 17.99(11.81-25.68) | 35.74(21.72-55.2) | 98.67 | 4.3(6.15-2.83) | 3.74(5.76-2.33) | -0.283(-0.858-0.295) |
| Mexico | Female | 2270.65(1914.48-2679.44) | 9159.46(6958.95-11790.5) | 303.38 | 9.76(11.51-8.19) | 14.39(18.48-10.93) | 1.342(0.951-1.735) |
| Micronesia (Federated States of) | Female | 4.3(2.23-7.45) | 5.47(2.6-10.23) | 27.21 | 17.32(29.79-8.98) | 13.35(24.83-6.57) | -0.904(-1.035--0.773) |
| Monaco | Female | 2.15(1.25-3.3) | 6.54(3.86-10.09) | 204.19 | 6.08(9.24-3.68) | 14.54(22.08-8.65) | 3.042(2.833-3.251) |
| Mongolia | Female | 916.09(544.04-1449.12) | 5117.68(3038.78-8016.82) | 458.64 | 159.23(253.57-94.87) | 394.25(600.23-236.3) | 3.142(2.785-3.501) |
| Montenegro | Female | 109.77(71-155.94) | 132.12(85.21-195.54) | 20.36 | 31.56(44.59-20.55) | 25.37(37.14-16.43) | -0.704(-0.943--0.466) |
| Morocco | Female | 108.67(62.47-177.17) | 235.91(128.52-398.88) | 117.09 | 1.57(2.54-0.89) | 1.48(2.48-0.82) | -0.183(-0.284--0.082) |
| Mozambique | Female | 227.87(138.96-348.4) | 529.77(301.46-866.08) | 132.49 | 6.68(10.02-4.14) | 8.04(12.99-4.64) | 0.682(0.419-0.946) |
| Myanmar | Female | 620.05(323.43-1101.17) | 2312.11(1383.35-3657.5) | 272.89 | 4.77(8.48-2.55) | 8.44(13.27-5.09) | 2.17(1.43-2.914) |
| Namibia | Female | 20.33(11.41-32.32) | 62.86(35.33-101.88) | 209.20 | 4.94(7.83-2.78) | 7.46(12.12-4.23) | 1.494(1.258-1.73) |
| Nauru | Female | 0.34(0.18-0.56) | 0.36(0.17-0.69) | 5.88 | 16.19(26.42-9.22) | 12.67(23.2-6.33) | -0.867(-1.039--0.696) |
| Nepal | Female | 249.04(138.08-438.41) | 634.05(372.9-996.67) | 154.60 | 4.91(8.4-2.76) | 5.16(8.04-3.03) | 0.176(0.117-0.236) |
| Netherlands | Female | 544.08(378.34-736.57) | 1526.01(1023.19-2110.76) | 180.48 | 5.05(6.77-3.54) | 9.34(12.8-6.43) | 2.134(1.878-2.391) |
| New Zealand | Female | 142.74(119.19-170.03) | 389.66(317.17-465.42) | 172.99 | 7.06(8.45-5.88) | 10.48(12.51-8.53) | 1.288(0.782-1.796) |
| Nicaragua | Female | 89.37(59.18-126.12) | 332.65(210.33-486.53) | 272.22 | 10.31(14.77-6.78) | 13.3(19.31-8.39) | 1.037(-0.381-2.475) |
| Niger | Female | 12.75(7.48-20.21) | 38.72(21.44-63.12) | 203.69 | 0.88(1.37-0.52) | 0.89(1.42-0.5) | 0.008(-0.123-0.139) |
| Nigeria | Female | 2179.95(1473.74-3083.55) | 5076.14(3332.32-7233.29) | 132.86 | 10(14.17-6.81) | 10.35(14.45-6.94) | 0.101(-0.046-0.248) |
| Niue | Female | 0.14(0.08-0.23) | 0.1(0.06-0.17) | -28.57 | 12.57(20.05-7.23) | 9.01(14.98-5.09) | -1.143(-1.352--0.934) |
| North Macedonia | Female | 384.17(259.33-538.4) | 521(319.05-799.02) | 35.62 | 38.01(52.45-26.19) | 30.37(46.38-18.89) | -0.857(-1.191--0.521) |
| Northern Mariana Islands | Female | 1.07(0.62-1.78) | 1.74(0.99-2.73) | 62.62 | 11.97(18.73-7) | 5.81(8.83-3.49) | -2.459(-2.863--2.053) |
| Norway | Female | 136.04(113.69-161.22) | 300.29(244.43-357.65) | 120.74 | 4.05(4.77-3.41) | 6.7(8.01-5.46) | 1.814(1.68-1.948) |
| Oman | Female | 9.06(4.75-15.52) | 25.09(14.4-39.57) | 176.93 | 2.93(5.05-1.54) | 3.25(5.11-1.83) | 0.342(-0.132-0.818) |
| Pakistan | Female | 3222.44(1989.24-4803.91) | 6338.18(4382.62-8913.36) | 96.69 | 11.55(17.39-7.02) | 10.32(14.48-7.08) | -0.383(-0.497--0.269) |
| Palau | Female | 0.16(0.09-0.25) | 0.3(0.17-0.48) | 87.50 | 3.11(4.97-1.78) | 2.7(4.24-1.52) | -0.501(-0.582--0.419) |
| Palestine | Female | 41.77(22.73-69.89) | 66.68(40-101.82) | 59.64 | 8.56(14.57-4.62) | 5.31(8.27-3.16) | -1.65(-2.029--1.269) |
| Panama | Female | 100.38(66.96-138.76) | 240.64(149.49-361.94) | 139.73 | 13.19(18.45-8.56) | 11.45(17.18-7.07) | -0.424(-0.967-0.123) |
| Papua New Guinea | Female | 13.71(7.85-22.01) | 32.81(18.95-53.46) | 139.31 | 1.49(2.34-0.86) | 1.46(2.37-0.84) | -0.072(-0.189-0.046) |
| Paraguay | Female | 125.39(83.69-177.67) | 165.89(103.24-256.51) | 32.30 | 10.59(15.12-7.04) | 5.71(8.84-3.55) | -2.025(-2.359--1.691) |
| Peru | Female | 2283.05(1542.92-3238.7) | 2614.53(1602.11-3970.18) | 14.52 | 37.08(53.15-24.97) | 15.92(24.26-9.65) | -2.91(-3.439--2.378) |
| Philippines | Female | 2608.94(1978.27-3320.15) | 5196.63(3734.55-7048.46) | 99.19 | 15.66(20.08-11.85) | 11.76(15.82-8.49) | -0.959(-1.15--0.766) |
| Poland | Female | 9497.01(8097.11-10967.96) | 3016.69(2266.4-3970.75) | -68.24 | 37.07(42.8-31.74) | 7.86(10.31-5.87) | -5.179(-5.993--4.359) |
| Portugal | Female | 414.84(284.33-580.81) | 942.85(615.14-1320.53) | 127.28 | 5.39(7.41-3.73) | 8.06(11.23-5.34) | 1.402(1.074-1.731) |
| Puerto Rico | Female | 515.6(354.45-692.31) | 311.32(200.01-455.01) | -39.62 | 26.1(35.03-17.95) | 8.45(12.58-5.43) | -3.855(-4.718--2.985) |
| Qatar | Female | 4.9(2.77-7.98) | 24.39(13-41.16) | 397.76 | 15.17(25.01-8.27) | 12.77(21.14-7.09) | -0.533(-0.819--0.247) |
| Republic of Korea | Female | 2836.05(1767.96-4361.02) | 10483.36(7022.54-15160.37) | 269.65 | 15.35(23.81-9.54) | 22.02(31.74-15.03) | 1.254(0.644-1.868) |
| Republic of Moldova | Female | 325.32(244.71-406.73) | 406.9(284.68-537.86) | 25.08 | 12.17(15.1-9.17) | 12.29(16.28-8.66) | -0.222(-1.896-1.481) |
| Romania | Female | 1167.57(791.26-1596.6) | 2406.37(1596.86-3323.67) | 106.10 | 7.51(10.16-5.2) | 12.59(17.64-8.37) | 1.801(1.503-2.099) |
| Russian Federation | Female | 7868.55(6572.9-9218.67) | 15945.24(12248.82-20475.46) | 102.65 | 6.89(8.08-5.77) | 11.6(15.01-8.91) | 1.781(1.261-2.303) |
| Rwanda | Female | 411.12(226.6-720.16) | 674.36(419.82-1042.92) | 64.03 | 23.46(39.81-13.32) | 17.81(27.21-11.36) | -0.983(-1.335--0.629) |
| Saint Kitts and Nevis | Female | 10.86(7.25-14.8) | 4.12(2.65-6.05) | -62.06 | 52.78(71.55-35.1) | 11.79(17.14-7.65) | -5.031(-5.767--4.288) |
| Saint Lucia | Female | 15.31(10.89-20.54) | 9.29(6.24-12.95) | -39.32 | 31.32(41.96-22.2) | 8.22(11.44-5.53) | -4.486(-5.217--3.749) |
| Saint Vincent and the Grenadines | Female | 10.58(7.32-14.4) | 7.25(4.94-10.1) | -31.47 | 26.74(36.44-18.38) | 10.97(15.29-7.53) | -3.005(-3.319--2.689) |
| Samoa | Female | 2.82(1.66-4.53) | 4.48(2.45-7.31) | 58.87 | 6.08(9.67-3.61) | 5.83(9.41-3.17) | -0.161(-0.233--0.09) |
| San Marino | Female | 0.77(0.47-1.19) | 1.87(1.01-3.25) | 142.86 | 4.41(6.73-2.73) | 6.42(11.38-3.47) | 1.304(1.07-1.539) |
| Sao Tome and Principe | Female | 1.58(0.95-2.41) | 3.14(1.73-5.21) | 98.73 | 4.52(6.84-2.72) | 5.45(9.05-2.97) | 0.731(0.54-0.922) |
| Saudi Arabia | Female | 61.95(35.55-102.2) | 173.41(93.74-294.98) | 179.92 | 2.45(4.11-1.4) | 2.23(3.75-1.21) | -0.27(-0.627-0.088) |
| Senegal | Female | 106.25(65.2-164.68) | 229.91(131.45-362.64) | 116.39 | 6.37(9.84-3.95) | 5.68(8.89-3.24) | -0.367(-1.087-0.358) |
| Serbia | Female | 1557.41(996.11-2311.01) | 1610.83(991.24-2365.89) | 3.43 | 25.05(36.67-16.29) | 19.2(28.12-12) | -0.849(-1.073--0.624) |
| Seychelles | Female | 3.94(2.47-5.9) | 5.08(3.15-7.61) | 28.93 | 12.77(19.24-7.99) | 8.83(13.28-5.47) | -1.292(-1.628--0.954) |
| Sierra Leone | Female | 105.19(61.03-162.21) | 197.6(111.36-317.94) | 87.85 | 10.83(16.82-6.26) | 10.29(16.72-5.78) | -0.181(-0.361-0) |
| Singapore | Female | 47.93(30.53-71.95) | 181.94(110.31-279.54) | 279.60 | 3.99(6.02-2.53) | 4.55(7-2.76) | 0.513(0.041-0.987) |
| Slovakia | Female | 735.07(520.26-979.84) | 740.19(464.08-1102.05) | 0.70 | 21.65(28.53-15.36) | 14.64(21.62-9.15) | -1.423(-1.75--1.096) |
| Slovenia | Female | 174.57(109.24-255.78) | 286.36(177.14-428.76) | 64.04 | 12.08(17.69-7.58) | 13.2(19.41-8.29) | 0.289(-0.197-0.777) |
| Solomon Islands | Female | 11.52(6.28-19.35) | 22.11(12.37-35.63) | 91.93 | 14.59(24.27-8.09) | 11.11(17.17-6.44) | -0.965(-1.25--0.678) |
| Somalia | Female | 162.64(74.51-303.29) | 418.52(220.65-743.31) | 157.33 | 10.63(19.08-5.11) | 10.01(18.08-5.41) | -0.196(-0.31--0.082) |
| South Africa | Female | 2045.34(1453.16-2724.26) | 2737.08(2063.92-3746.86) | 33.82 | 15.79(21.17-11.02) | 10.19(13.89-7.67) | -1.448(-1.926--0.968) |
| South Sudan | Female | 102.15(61.14-156.56) | 159.52(86.52-270.46) | 56.16 | 9.14(14.03-5.4) | 7.81(13.03-4.2) | -0.537(-0.721--0.352) |
| Spain | Female | 2550.31(1688.97-3587.56) | 3765.1(2409.6-5606.13) | 47.63 | 8.43(11.76-5.66) | 7.97(11.8-5.25) | -0.183(-0.388-0.024) |
| Sri Lanka | Female | 211.33(130.19-317.35) | 732.95(420.73-1180.38) | 246.83 | 3.73(5.62-2.27) | 5.04(8-2.89) | 1.115(0.422-1.812) |
| Sudan | Female | 188.14(89.48-308.85) | 328.02(177.97-523.36) | 74.35 | 4.08(6.74-1.92) | 3.69(5.93-2) | -0.344(-0.411--0.277) |
| Suriname | Female | 40.5(27.38-55.87) | 33.24(20.98-48.64) | -17.93 | 29.68(41.23-20.15) | 10.19(14.87-6.49) | -3.656(-4.106--3.204) |
| Sweden | Female | 706.82(572.15-862.15) | 759.63(613.75-926.31) | 7.47 | 9.77(11.88-7.91) | 8.04(9.83-6.52) | -0.674(-0.954--0.393) |
| Switzerland | Female | 313.49(209.34-425.64) | 871.98(579.96-1243.23) | 178.15 | 5.75(7.79-3.89) | 10.57(14.98-7.09) | 2.089(1.833-2.346) |
| Syrian Arab Republic | Female | 135.04(79.7-215.19) | 269.74(152.31-444.14) | 99.75 | 5.16(8.19-3.02) | 4.29(6.85-2.45) | -0.641(-1.038--0.243) |
| Taiwan (Province of China) | Female | 729.02(463.96-1066.39) | 1203.05(698.56-1876.43) | 65.02 | 8.98(12.99-5.78) | 5.73(8.87-3.36) | -1.833(-2.791--0.865) |
| Tajikistan | Female | 32.98(21.15-48.28) | 245.93(149.39-376.31) | 645.69 | 2.12(3.12-1.34) | 8.69(12.85-5.34) | 4.993(4.548-5.44) |
| Thailand | Female | 9208.47(5735.61-13603.83) | 26474.1(16101.37-41218.33) | 187.50 | 45.91(67.82-28.56) | 47.32(73.15-28.97) | 0.106(-0.223-0.436) |
| Timor-Leste | Female | 21.92(11.37-37.77) | 45.9(26.51-74.05) | 109.40 | 14.04(24.33-7.54) | 10.61(16.91-6.27) | -0.931(-1.043--0.819) |
| Togo | Female | 88.2(54.92-135.43) | 226.71(131.16-349.08) | 157.04 | 12.21(19-7.37) | 10(15.17-5.85) | -0.714(-0.866--0.561) |
| Tokelau | Female | 0.11(0.06-0.19) | 0.08(0.04-0.14) | -27.27 | 15.8(26.6-8.75) | 11.85(19.83-6.74) | -0.995(-1.045--0.945) |
| Tonga | Female | 6.93(4.02-11.15) | 8.65(5.02-13.48) | 24.82 | 22.3(35.52-13.05) | 20.45(32.11-11.78) | -0.33(-0.447--0.213) |
| Trinidad and Tobago | Female | 148.96(104.48-203.44) | 117.28(73.63-175.05) | -21.27 | 33.2(45.63-23.2) | 12.2(18.05-7.57) | -3.341(-3.892--2.786) |
| Tunisia | Female | 40.21(20.24-71) | 92.24(43.24-167.98) | 129.40 | 1.56(2.73-0.78) | 1.39(2.51-0.65) | -0.4(-0.656--0.144) |
| Turkey | Female | 948.51(529.18-1603.93) | 1368.66(811.99-2062.1) | 44.30 | 4.95(8.36-2.76) | 2.93(4.41-1.74) | -1.814(-2.089--1.538) |
| Turkmenistan | Female | 32.05(21.06-46.33) | 444.94(276.26-672.54) | 1288.27 | 2.84(4.11-1.85) | 18.87(28.3-11.91) | 6.724(6.007-7.447) |
| Tuvalu | Female | 0.66(0.34-1.17) | 0.64(0.33-1.09) | -3.03 | 15.91(27.74-8.42) | 11.32(19.19-6.03) | -1.176(-1.265--1.086) |
| Uganda | Female | 452.69(279.64-660.99) | 2105.19(1283.41-3136.28) | 365.04 | 12.81(18.9-7.97) | 24.55(36.26-14.99) | 2.267(2.018-2.516) |
| Ukraine | Female | 1648.25(1363.37-1935.03) | 3763.5(2838.63-4953.92) | 128.33 | 3.6(4.25-2.99) | 8.6(11.44-6.41) | 2.871(2.202-3.545) |
| United Arab Emirates | Female | 7.07(3.64-12.32) | 37.04(14.63-66.69) | 423.90 | 4.22(7.87-1.99) | 2.58(4.78-0.97) | -1.754(-2.848--0.647) |
| United Kingdom | Female | 2755.53(2331.72-3199.53) | 7248.4(6092.67-8514.79) | 163.05 | 5.87(6.83-4.97) | 11.96(13.98-10.08) | 2.48(2.235-2.726) |
| United Republic of Tanzania | Female | 448.75(286.11-661.95) | 1069.59(672.73-1579.12) | 138.35 | 7.37(10.72-4.7) | 7.67(11.23-4.8) | 0.138(-0.021-0.297) |
| United States of America | Female | 7158.96(6077.79-8371.3) | 21100.51(17169.05-25160.91) | 194.74 | 4.21(4.92-3.55) | 7.56(8.97-6.17) | 2.046(1.867-2.225) |
| United States Virgin Islands | Female | 8.48(5.74-12.12) | 8.13(5.21-11.77) | -4.13 | 17.9(25.54-12.21) | 7.78(11.24-5.01) | -2.822(-3.167--2.477) |
| Uruguay | Female | 66.37(43.6-94.3) | 126.23(82.54-181.76) | 90.19 | 3.13(4.41-2.07) | 4.47(6.42-2.95) | 1.245(1.158-1.332) |
| Uzbekistan | Female | 159.73(104.13-233.8) | 2840.36(1766-4238.19) | 1678.23 | 2.44(3.57-1.58) | 22.06(32.06-13.96) | 7.931(7.657-8.205) |
| Vanuatu | Female | 3.96(2.01-7.1) | 10.21(5.24-18.19) | 157.83 | 12.24(22.16-6.45) | 11.29(19.89-5.89) | -0.322(-0.694-0.051) |
| Venezuela (Bolivarian Republic of) | Female | 1655.72(1102.21-2283.55) | 1078.03(653.05-1706.76) | -34.89 | 31.47(43.26-20.7) | 6.87(10.83-4.18) | -5.196(-5.912--4.475) |
| Viet Nam | Female | 1487.51(883.88-2358.87) | 2704.2(1562.66-4269.6) | 81.79 | 6.36(10.1-3.78) | 5.2(8.14-2.95) | -0.694(-0.873--0.514) |
| Yemen | Female | 45.8(21.22-85.76) | 126.4(65.18-223.47) | 175.98 | 1.73(3.24-0.81) | 1.75(3.04-0.92) | 0.046(-0.053-0.144) |
| Zambia | Female | 128.02(76.42-205.18) | 302.48(179.05-477.16) | 136.28 | 8.12(12.81-4.87) | 7.59(11.87-4.53) | -0.229(-0.337--0.121) |
| Zimbabwe | Female | 479.96(288.58-717.22) | 1322.92(729.43-2113.98) | 175.63 | 21.3(31.85-12.74) | 29.54(47.31-16.41) | 1.193(0.651-1.738) |
| Afghanistan | Male | 1303.49(723.53-2077.02) | 1954.52(1149-3068.03) | 49.95 | 33.55(53.18-19.13) | 30.35(46.56-18.13) | -0.345(-0.436--0.253) |
| Albania | Male | 1378.74(995.02-1789.7) | 2156.33(1338.58-3221.28) | 56.40 | 132.25(169.79-96.28) | 104.12(153.75-66.12) | -0.891(-1.323--0.457) |
| Algeria | Male | 374.25(226.88-584.03) | 1399.6(809.91-2209.99) | 273.97 | 5.93(9.24-3.64) | 8.07(12.87-4.72) | 1.037(0.766-1.308) |
| American Samoa | Male | 3.06(1.71-4.86) | 8.06(4.88-12.35) | 163.40 | 23.1(36.21-13.17) | 32.07(48.52-19.9) | 1.111(0.795-1.428) |
| Andorra | Male | 43.5(28.43-64.65) | 96.48(64.48-133.88) | 121.79 | 143.66(211.85-95.38) | 136.97(189.2-92.24) | -0.123(-0.171--0.074) |
| Angola | Male | 297.56(170.22-482.86) | 998.39(647.92-1467.96) | 235.53 | 14.3(22.69-8.24) | 18.11(26.68-11.56) | 0.855(0.713-0.997) |
| Antigua and Barbuda | Male | 24.52(17.56-32.4) | 17.25(12.04-23.51) | -29.65 | 106.72(141.44-75.46) | 34.69(47-24.55) | -3.823(-4.523--3.119) |
| Argentina | Male | 2966.62(2071.54-3915.85) | 6974.69(5103.92-8840.55) | 135.11 | 20.04(26.26-14.17) | 29.09(36.72-21.29) | 1.345(1.093-1.598) |
| Armenia | Male | 125.9(87.61-170.11) | 1454.06(1002.43-1975) | 1054.93 | 10.72(14.18-7.67) | 81.22(109.01-56.99) | 7.091(5.306-8.906) |
| Australia | Male | 3392.27(2657.04-4063.48) | 12357.21(9236.93-15378.68) | 264.28 | 36.97(44.36-28.99) | 67.6(84.29-50.57) | 2.108(1.96-2.257) |
| Austria | Male | 4037.81(3422.76-4642.76) | 7551.4(6186.46-9060.76) | 87.02 | 88.3(101.24-75.02) | 99.49(119.68-81.58) | 0.398(0.012-0.786) |
| Azerbaijan | Male | 206.8(138.25-283.51) | 2023.63(1197.56-3215.08) | 878.54 | 10(13.43-6.89) | 45.05(70.45-27.05) | 5.364(4.761-5.971) |
| Bahamas | Male | 98.59(72.19-129.66) | 83.41(55.71-117.46) | -15.40 | 134.34(177.28-97.7) | 43.94(60.61-29.92) | -3.888(-4.388--3.386) |
| Bahrain | Male | 22.12(13.97-33.68) | 94.7(54.11-151.89) | 328.12 | 22.89(34.39-14.1) | 15.17(23.89-9.16) | -1.494(-2.185--0.798) |
| Bangladesh | Male | 8464.86(5605.56-12153.89) | 15583(9822.27-23062.58) | 84.09 | 29.64(42.38-19.6) | 22.29(32.84-13.98) | -0.922(-1.12--0.724) |
| Barbados | Male | 43.78(24.41-69.34) | 75.29(52.19-101) | 71.97 | 36.77(58.93-20.42) | 33.71(45.14-23.42) | -0.35(-0.715-0.015) |
| Belarus | Male | 1020.13(767.04-1247.66) | 2902.81(1664.98-4613.02) | 184.55 | 20.43(24.88-15.66) | 45.91(72.35-26.95) | 2.94(2.002-3.886) |
| Belgium | Male | 2721.94(2100.86-3282.2) | 5732.34(4322.02-7147.6) | 110.60 | 41.49(49.87-32.28) | 59.58(73.56-45.41) | 1.327(0.815-1.842) |
| Belize | Male | 27.46(20.14-35.96) | 54.9(39.89-72.95) | 99.93 | 57.59(75.42-42.13) | 37.9(50.46-27.42) | -1.381(-1.794--0.968) |
| Benin | Male | 488.18(310.19-780.61) | 931.88(555.45-1502.01) | 90.89 | 49(77.54-31.12) | 37.91(61.34-22.91) | -0.878(-1.021--0.734) |
| Bermuda | Male | 30.51(22.91-39.1) | 16.97(11.94-22.92) | -44.38 | 107.54(137.63-81.96) | 29.45(39.65-20.78) | -4.477(-5.019--3.933) |
| Bhutan | Male | 41.8(22.88-73.18) | 110.43(61.68-176.33) | 164.19 | 30.2(52.33-17.05) | 36.81(58.98-20.7) | 0.673(0.502-0.845) |
| Bolivia (Plurinational State of) | Male | 586.23(371.13-873.33) | 1651.28(1005.24-2536.09) | 181.68 | 37.41(55.19-23.34) | 38.55(59.08-23.71) | 0.135(-0.027-0.298) |
| Bosnia and Herzegovina | Male | 1645.55(1165.23-2156.98) | 2729.46(1862.08-3819.36) | 65.87 | 85.99(110.65-63.05) | 99.29(138.2-68.16) | 0.482(0.2-0.765) |
| Botswana | Male | 21.35(6.21-77.25) | 113.7(66.34-182.16) | 432.55 | 7.41(26.61-2.15) | 15.56(24.38-9.42) | 2.704(2.39-3.02) |
| Brazil | Male | 9177.77(7964.63-10468.55) | 32522.57(27944.82-37184.99) | 254.36 | 20.35(23.14-17.6) | 29.44(33.58-25.35) | 1.261(1.018-1.504) |
| Brunei Darussalam | Male | 20.52(12.11-31.1) | 75.11(44.8-122.19) | 266.03 | 36.73(56.47-22.31) | 43.33(67.47-26.7) | 0.637(0.164-1.111) |
| Bulgaria | Male | 6224.06(4747.94-7679.95) | 5349.9(3853.45-7261.04) | -14.04 | 103.13(126.84-79.25) | 90.55(122.7-64.76) | -0.392(-1.001-0.221) |
| Burkina Faso | Male | 360.03(217.89-534.56) | 598.85(358.59-890.01) | 66.33 | 16.75(24.58-10.07) | 13.76(20.69-8.43) | -0.617(-0.769--0.465) |
| Burundi | Male | 550.44(338.68-844.14) | 782.28(406.09-1553.84) | 42.12 | 49.24(75.86-30.18) | 30.22(59.7-15.77) | -1.678(-1.842--1.514) |
| Cabo Verde | Male | 7.14(4.67-10.15) | 198.12(133.76-283.3) | 2674.79 | 7.47(10.68-4.87) | 98.66(138.12-66.78) | 9.422(8.459-10.394) |
| Cambodia | Male | 1045.5(665.8-1613.59) | 3675.7(2362.17-5343.91) | 251.57 | 49.85(76.66-31.71) | 67.96(97.77-43.42) | 1.095(0.925-1.265) |
| Cameroon | Male | 114.99(61.63-214.84) | 381.62(225.99-605.52) | 231.87 | 4.93(9.05-2.66) | 5.92(9.31-3.58) | 0.659(0.489-0.829) |
| Canada | Male | 6973.43(6098.72-7745.26) | 26963.33(21857.38-32183.09) | 286.66 | 47.04(52.27-41.17) | 88.63(105.25-72.04) | 2.27(1.973-2.568) |
| Central African Republic | Male | 159.57(84.91-261.35) | 252.31(129.06-453.62) | 58.12 | 26.68(41.73-14.5) | 23.83(43.19-12.68) | -0.398(-0.598--0.198) |
| Chad | Male | 533.17(287.15-870.54) | 991.06(561.69-1563.03) | 85.88 | 37.48(62.1-20.25) | 30.96(48.15-17.89) | -0.643(-0.766--0.52) |
| Chile | Male | 1303.89(960.96-1662.47) | 3888.3(2831.19-5022.42) | 198.21 | 27.69(35.22-20.4) | 35.08(45.29-25.62) | 0.817(0.291-1.346) |
| China | Male | 582786.2(325878.29-948101.65) | 509274.43(304931.08-791268.79) | -12.61 | 115.73(185.01-67.64) | 49.99(77.79-30.08) | -2.821(-3.243--2.396) |
| Colombia | Male | 2481.03(1875.31-3109.56) | 7156.77(4637.59-10446.22) | 188.46 | 27.07(33.63-20.32) | 29.93(43.42-19.47) | 0.299(-1.033-1.649) |
| Comoros | Male | 27.22(10.36-65.31) | 49.69(23.83-112.17) | 82.55 | 24.37(58.57-9.7) | 21.7(49.08-10.54) | -0.432(-1.176-0.318) |
| Congo | Male | 146.7(77.69-248.57) | 270.09(151.85-430.23) | 84.11 | 28.32(46.47-15.25) | 19.68(31.06-11.4) | -1.212(-1.464--0.961) |
| Cook Islands | Male | 4.27(2.4-6.57) | 10.23(6.47-14.91) | 139.58 | 59.36(90.4-33.94) | 85.83(125.78-55.04) | 1.288(1.009-1.568) |
| Costa Rica | Male | 598.43(448.76-745.97) | 1681.85(1137.38-2402.66) | 181.04 | 68.17(84.73-51.18) | 69.79(99.34-47.32) | 0.184(-0.636-1.012) |
| Croatia | Male | 1476.34(1113.9-1824.84) | 2532.58(1760.6-3412.97) | 71.54 | 53.76(65.73-41.66) | 68.13(91.7-47.22) | 0.852(0.258-1.449) |
| Cuba | Male | 3515.74(2630.04-4400.68) | 2896.44(1968.72-3985.55) | -17.62 | 68.86(86.42-51.42) | 32.64(44.87-22.4) | -2.406(-3.275--1.529) |
| Cyprus | Male | 189.45(132.92-255.23) | 426.44(313.09-558.31) | 125.09 | 47.22(63.38-33.31) | 45.66(59.64-33.83) | -0.209(-0.326--0.091) |
| Czechia | Male | 5152.35(4213.29-6065.82) | 5144.97(3770.39-6753.6) | -0.14 | 87.58(103-71.76) | 55.79(73.51-41.27) | -1.527(-2.075--0.977) |
| C么te d'Ivoire | Male | 1487.64(831.23-2247.84) | 2468.95(1465.44-3872.69) | 65.96 | 60.37(92.35-35.85) | 39.69(62.67-24.12) | -1.451(-1.764--1.137) |
| Democratic People's Republic of Korea | Male | 6174.37(3125.68-10797.74) | 7903.45(4182.51-13344.35) | 28.00 | 74.26(127.18-40.07) | 50.79(84.11-28.11) | -1.292(-1.412--1.173) |
| Democratic Republic of the Congo | Male | 1059.24(665.01-1589.49) | 2134.5(1233.33-3396.12) | 101.51 | 13.17(19.66-8.34) | 11.55(17.86-6.73) | -0.476(-0.697--0.255) |
| Denmark | Male | 1113.7(880.02-1343.32) | 2647.65(1961.65-3331.98) | 137.73 | 32.2(38.93-25.59) | 51.64(65.34-38.18) | 1.681(1.531-1.831) |
| Djibouti | Male | 22.55(9.12-57.55) | 100.33(46.51-217.02) | 344.92 | 27.49(68.7-11.57) | 28.21(58.43-13.81) | 0.112(-0.183-0.407) |
| Dominica | Male | 33.4(23.79-44.54) | 19.38(12.89-27.58) | -41.98 | 113.95(153.13-79.74) | 43.37(60.87-29.39) | -3.254(-3.714--2.791) |
| Dominican Republic | Male | 927.86(665.03-1250.08) | 3006.57(1666.75-5677.26) | 224.03 | 46.91(63.57-33.19) | 64.75(120.12-36.41) | 1.113(0.772-1.455) |
| Ecuador | Male | 526.79(370.21-721.96) | 1992.93(1313.16-2920.64) | 278.32 | 19.42(26.61-13.58) | 27.38(40.23-18.04) | 1.168(0.9-1.437) |
| Egypt | Male | 9761.45(5714.53-15652.61) | 30659.14(16756.8-54600.39) | 214.08 | 59.3(93.91-34.42) | 78.35(139.72-42.82) | 1.025(0.826-1.224) |
| El Salvador | Male | 462.7(338.45-599.85) | 681.92(438.76-986.47) | 47.38 | 32.29(41.79-23.58) | 27.6(40.02-17.75) | -0.611(-1.094--0.127) |
| Equatorial Guinea | Male | 17.76(9.81-29.01) | 60.13(29.3-105.69) | 238.57 | 18.62(29.84-10.56) | 27.39(47.1-13.12) | 1.378(1.155-1.6) |
| Eritrea | Male | 146.89(63.03-320.45) | 349.47(155.76-711.23) | 137.91 | 31.12(67.1-12.85) | 27.14(54.21-12.32) | -0.435(-0.793--0.076) |
| Estonia | Male | 275.65(205.58-345.2) | 661.23(467.94-915.2) | 139.88 | 34.74(42.95-26.37) | 68.26(94.63-48.47) | 2.714(1.89-3.545) |
| Eswatini | Male | 71.25(31.34-202.11) | 773.52(155.31-1616.51) | 985.64 | 48.27(134.68-21.86) | 281.61(583.7-59.35) | 6.353(5.875-6.833) |
| Ethiopia | Male | 2784.43(1760.58-4114.59) | 5288.98(3814.88-7470.38) | 89.95 | 25.09(36.39-16.29) | 24.65(34.51-17.79) | -0.039(-0.174-0.097) |
| Fiji | Male | 53.97(33.12-84.62) | 138.92(82.07-219.55) | 157.40 | 26.84(41.85-16.37) | 34.96(53.37-21.16) | 0.977(0.741-1.214) |
| Finland | Male | 1358.51(1052.51-1656.24) | 3157.41(2374.01-3983.07) | 132.42 | 46.67(56.85-36.24) | 59.04(74.29-44.93) | 0.812(0.613-1.012) |
| France | Male | 31966.49(23470.87-40509.54) | 47791.14(33408.83-63556.63) | 49.50 | 92.37(116.6-67.79) | 86.72(115.95-60.92) | -0.276(-0.516--0.036) |
| Gabon | Male | 68.11(33.59-106.29) | 142.78(71.87-265.83) | 109.63 | 25.11(39.53-12.49) | 26.95(49.55-14) | 0.25(0.048-0.453) |
| Gambia | Male | 513.48(311.79-803.95) | 1881.33(1049.37-3006.44) | 266.39 | 233.44(359.38-145.75) | 354.23(551.91-201.98) | 1.487(0.954-2.022) |
| Georgia | Male | 320.39(213.94-433.07) | 1429.91(983.98-1987.64) | 346.30 | 12.79(16.99-8.78) | 60.38(82.9-42.01) | 5.567(4.061-7.095) |
| Germany | Male | 24429.78(19946.39-29000.15) | 62547.88(51368.95-74293.67) | 156.03 | 49.2(58.28-40.31) | 77.77(92.26-63.8) | 1.615(1.44-1.79) |
| Ghana | Male | 1783.49(1112.79-2810.16) | 5257.62(3267.76-7861.35) | 194.79 | 54.26(85.32-33.52) | 67.25(99.34-42.31) | 0.742(0.529-0.956) |
| Greece | Male | 1764(1299.03-2224.07) | 3974.88(2961.58-5143.24) | 125.33 | 24.87(31.35-18.41) | 40.54(52.38-29.64) | 1.718(1.378-2.06) |
| Greenland | Male | 19.42(13.9-25.72) | 45.55(29.95-64.61) | 134.55 | 86.62(112.54-64.93) | 109.13(150.93-73.89) | 0.762(0.53-0.995) |
| Grenada | Male | 34.51(25.15-44.94) | 23.72(17.13-31.07) | -31.27 | 117.49(153.61-85.46) | 41.99(54.23-30.96) | -3.523(-4.066--2.976) |
| Guam | Male | 8.45(5.14-12.62) | 37.49(23.29-57.01) | 343.67 | 18.67(27.88-11.38) | 38.18(57.21-24.04) | 2.497(2.088-2.907) |
| Guatemala | Male | 1564.32(1090.58-2113.15) | 2210.75(1470.73-3187.3) | 41.32 | 80.46(108.48-56.36) | 42.86(62.53-28.26) | -2.231(-2.893--1.565) |
| Guinea | Male | 3684.16(2362.36-5449) | 7557.31(4197.79-11814.84) | 105.13 | 212.92(312.71-136.5) | 257.16(397.94-143.12) | 0.67(0.52-0.82) |
| Guinea-Bissau | Male | 127.15(71.52-213.22) | 168.53(99.86-267.99) | 32.54 | 60(99.33-34.26) | 46.29(73.41-27.58) | -0.96(-1.165--0.755) |
| Guyana | Male | 162.26(113.76-217.48) | 125.05(80.85-178.3) | -22.93 | 81.49(109.33-58.04) | 40.04(56.26-26.56) | -2.328(-2.796--1.857) |
| Haiti | Male | 1227.04(623.7-2258.81) | 1710.05(777.91-3407.25) | 39.36 | 73.75(137.6-36.93) | 50.21(100.38-22.88) | -1.35(-1.687--1.013) |
| Honduras | Male | 1296.41(431.81-2139.01) | 5177.88(2166.83-8566.38) | 299.40 | 123.81(204.59-39.91) | 177.1(287.85-74.46) | 1.278(1.017-1.539) |
| Hungary | Male | 7622.43(6222.03-9003.48) | 3550.12(2551.83-4726.47) | -53.43 | 121.32(144.28-99.32) | 45.65(61.4-32.87) | -3.447(-4.154--2.735) |
| Iceland | Male | 33.03(24.45-42.53) | 109.02(80.45-140.6) | 230.06 | 25.29(32.51-18.76) | 42.68(55.02-31.71) | 1.804(1.316-2.295) |
| India | Male | 63385.79(47403.71-81152.75) | 171248.62(131996.8-216382.13) | 170.17 | 25.14(31.98-18.66) | 29.12(36.78-22.47) | 0.582(0.218-0.948) |
| Indonesia | Male | 9071.02(7056.97-11527.8) | 19051.65(14328.45-24858.87) | 110.03 | 17.75(22.51-13.75) | 17.63(22.57-13.56) | -0.018(-0.168-0.132) |
| Iran (Islamic Republic of) | Male | 2272.19(1626.11-3093.26) | 4903.29(3636.74-6532.22) | 115.80 | 16(21.6-11.69) | 13.7(18.13-10.12) | -0.591(-1.174--0.004) |
| Iraq | Male | 627.14(363.81-983.84) | 2561.7(1493.07-3979.18) | 308.47 | 16.02(25.13-9.32) | 22.19(34.3-13.01) | 1.132(0.527-1.74) |
| Ireland | Male | 374.37(286.24-467.65) | 1624.78(1209.28-2050.53) | 334.00 | 19.78(24.74-15.16) | 46.06(58.03-34.2) | 2.885(2.43-3.343) |
| Israel | Male | 528.79(363.57-712.64) | 1558.99(1111.06-2053.98) | 194.82 | 23.75(32.07-16.34) | 30.14(39.66-21.59) | 0.746(0.607-0.885) |
| Italy | Male | 31925.7(27605.57-36061.3) | 30903.45(25797.15-35936.06) | -3.20 | 81.99(92.49-71.18) | 54.11(63.02-45.09) | -1.531(-2.205--0.852) |
| Jamaica | Male | 401.81(298.9-515.57) | 462.37(314.09-652.85) | 15.07 | 48.81(63.01-36.46) | 32.42(45.78-22.16) | -1.441(-2.89-0.03) |
| Japan | Male | 66647.82(53443.76-86375.99) | 59044.91(47506.72-74498.44) | -11.41 | 82.71(106.69-66.51) | 42.54(54.09-34.22) | -2.214(-2.619--1.806) |
| Jordan | Male | 76.65(44.81-119.74) | 329.91(185.71-532.06) | 330.41 | 11.06(16.92-6.61) | 9.53(15.45-5.48) | -0.515(-0.738--0.291) |
| Kazakhstan | Male | 2916.92(2086.99-3747.28) | 7230.58(5083.39-9618.96) | 147.88 | 55.66(70.05-40.54) | 93.96(122.73-67.53) | 1.661(1.026-2.301) |
| Kenya | Male | 994.42(517.31-2078.07) | 3536.6(1997.5-5985.78) | 255.64 | 23.35(48.77-12.05) | 30.94(51.68-17.58) | 0.834(0.419-1.251) |
| Kiribati | Male | 12.33(7.07-20.23) | 19.66(10.88-31.42) | 59.45 | 61.44(100.25-35.38) | 51.83(79.64-30.25) | -0.576(-0.7--0.453) |
| Kuwait | Male | 40.36(25.42-60.3) | 123.3(71.08-195.63) | 205.50 | 9.66(14.36-6.06) | 8.18(12.94-4.84) | -0.25(-1.048-0.555) |
| Kyrgyzstan | Male | 185(129.76-245.22) | 699.3(480.41-944.39) | 278.00 | 14.93(19.6-10.62) | 33.26(44.04-23.27) | 2.857(2.23-3.488) |
| Lao People's Democratic Republic | Male | 999.55(569.47-1657.4) | 1670.46(1003.92-2581.75) | 67.12 | 91.74(148.91-53.41) | 71.46(109.29-43.59) | -0.865(-0.911--0.82) |
| Latvia | Male | 372.35(276.13-465.15) | 723.37(507.45-984.91) | 94.27 | 27.48(33.98-20.69) | 51.2(69.53-35.93) | 2.221(1.515-2.931) |
| Lebanon | Male | 190.99(114.32-291.84) | 353.15(200.81-602.41) | 84.90 | 15.99(24.15-9.65) | 14.95(25.38-8.45) | -0.254(-0.423--0.084) |
| Lesotho | Male | 208.88(87.45-617.44) | 1192.98(351.87-2045.32) | 471.13 | 43.98(127.96-18.82) | 202.85(352.7-60.93) | 5.545(5.175-5.916) |
| Liberia | Male | 308.28(185.7-494.74) | 414.56(242.27-719.42) | 34.48 | 49.31(78.71-29.87) | 35.61(60.69-20.79) | -1.143(-1.418--0.867) |
| Libya | Male | 176.67(102.31-285.31) | 505.06(286.77-839.52) | 185.88 | 16.86(27.45-9.92) | 18.17(30.34-10.4) | 0.277(-0.076-0.63) |
| Lithuania | Male | 466.41(344.52-579.66) | 1321.75(927.26-1746.11) | 183.39 | 25.97(31.98-19.31) | 63.84(84.35-45.08) | 3.093(2.208-3.987) |
| Luxembourg | Male | 119.08(91.46-146.59) | 239.57(164.04-327.92) | 101.18 | 51.31(62.74-39.36) | 52.1(71.24-35.89) | -0.021(-0.408-0.369) |
| Madagascar | Male | 698.07(288.97-1703.49) | 1225.43(509.83-2613.3) | 75.55 | 25.38(61.99-10.38) | 21.33(45.52-9.23) | -0.559(-0.925--0.193) |
| Malawi | Male | 577.47(302.91-1151.1) | 1033.26(655.28-1509.88) | 78.93 | 29.29(56.84-15.41) | 30.85(44.72-19.94) | 0.167(-0.292-0.627) |
| Malaysia | Male | 1391.27(902.18-2075.55) | 4592.48(2824.89-7222.96) | 230.09 | 29.42(44.44-18.86) | 32.55(50.98-19.8) | 0.435(0.238-0.632) |
| Maldives | Male | 29.83(15.66-54.67) | 83.46(53.42-122.69) | 179.79 | 53.4(94.01-29.27) | 47.3(68.8-30.36) | -0.44(-0.733--0.147) |
| Mali | Male | 2796.62(1700.3-4245.7) | 6010.25(3523.56-9410.97) | 114.91 | 120.92(182.98-74.75) | 120.78(188.29-71.62) | -0.007(-0.206-0.192) |
| Malta | Male | 42.3(30.93-53.66) | 129.74(94.15-170.33) | 206.71 | 21.57(27.44-15.8) | 31.12(39.87-22.82) | 1.293(0.873-1.716) |
| Marshall Islands | Male | 5.26(2.83-8.82) | 11.68(6.38-20.41) | 122.05 | 59.21(99.7-30.83) | 55.72(96.3-31.02) | -0.187(-0.336--0.037) |
| Mauritania | Male | 209.42(123.12-330.01) | 238.17(134.58-386.86) | 13.73 | 41.29(64.47-24.93) | 22.21(35.63-12.69) | -2.108(-2.363--1.851) |
| Mauritius | Male | 56.07(39.13-76.11) | 151.82(90.7-231.72) | 170.77 | 15.93(21.57-11.09) | 18.29(27.76-11.35) | 0.489(-0.229-1.213) |
| Mexico | Male | 4244.54(3744.93-4774.95) | 20754.4(15977.52-26269.48) | 388.97 | 19.97(22.43-17.58) | 37.46(47.34-28.96) | 2.359(2.12-2.599) |
| Micronesia (Federated States of) | Male | 15.22(8.82-25.02) | 23.53(12.02-40.26) | 54.60 | 57.73(94.55-33.42) | 59.69(99.3-31.86) | 0.115(0.005-0.226) |
| Monaco | Male | 13.91(9.36-19.47) | 44.49(30.7-61.49) | 219.84 | 50.01(69.19-34.06) | 112.52(155.47-77.17) | 2.853(2.507-3.201) |
| Mongolia | Male | 4002.87(2435.17-5740.61) | 14848.51(9476.03-21779.5) | 270.95 | 783.18(1110.7-482.23) | 1295.31(1835.51-866.52) | 1.747(1.637-1.857) |
| Montenegro | Male | 303.38(220.3-390.5) | 459.74(321.3-625.71) | 51.54 | 105.34(134.54-77.1) | 100.76(137.05-71.2) | -0.11(-0.39-0.171) |
| Morocco | Male | 540.49(315.9-871.44) | 1485(836.03-2372.51) | 174.75 | 7.94(12.51-4.64) | 9.68(15.09-5.66) | 0.689(0.296-1.084) |
| Mozambique | Male | 437.09(206.26-1017.39) | 2050.51(1091.94-3111.19) | 369.13 | 15.23(35.06-7.3) | 41.41(61.88-22.56) | 3.524(3.342-3.705) |
| Myanmar | Male | 2290.94(1355.09-3944.86) | 7427.49(4653.13-11005.49) | 224.21 | 20.88(34.55-12.54) | 35.94(52.43-22.83) | 1.941(1.312-2.573) |
| Namibia | Male | 52.16(20.32-143.1) | 264.99(166.29-395.88) | 408.03 | 14.68(39.98-5.7) | 39.62(58.32-25.31) | 3.534(3.188-3.882) |
| Nauru | Male | 1.36(0.71-2.29) | 1.58(0.78-2.79) | 16.18 | 53.84(86.79-30.37) | 60.14(98.21-33.19) | 0.401(0.33-0.471) |
| Nepal | Male | 873.68(579.67-1266.24) | 2474.8(1369.25-4388.31) | 183.26 | 17.68(25.23-11.67) | 22.91(40.33-13.04) | 0.92(0.762-1.079) |
| Netherlands | Male | 1975.9(1607.99-2334.13) | 6307.81(5000.66-7641.28) | 219.24 | 22.83(27.03-18.53) | 41.35(49.96-32.75) | 2.059(1.549-2.571) |
| New Zealand | Male | 728.12(626.39-827.66) | 2123.8(1842.76-2432.09) | 191.68 | 40.74(46.45-34.91) | 63.87(73.26-55.46) | 1.547(1.355-1.738) |
| Nicaragua | Male | 271.64(183.46-367.78) | 960.56(649.94-1356.38) | 253.62 | 35.43(48.4-23.87) | 46.43(64.97-31.57) | 0.889(0.378-1.403) |
| Niger | Male | 68.69(40.41-106.57) | 167.82(95.03-259.26) | 144.32 | 4.28(6.6-2.59) | 4.11(6.39-2.37) | -0.114(-0.288-0.062) |
| Nigeria | Male | 5219.02(3372.14-7648.26) | 9727.07(6618.52-13994.66) | 86.38 | 22.26(32.44-14.51) | 24.47(34.68-16.96) | 0.316(0.149-0.484) |
| Niue | Male | 0.44(0.27-0.69) | 0.49(0.3-0.74) | 11.36 | 46.14(71.99-28.12) | 48.11(71.66-29.54) | 0.15(0.095-0.205) |
| North Macedonia | Male | 1155.72(845.41-1486.96) | 1940.02(1297.15-2736.23) | 67.86 | 122.46(157.04-90.39) | 119.77(167.85-80.66) | -0.021(-0.381-0.341) |
| Northern Mariana Islands | Male | 3.67(2.02-6) | 13.8(8.29-21.93) | 276.02 | 25.59(39.17-14.83) | 43.56(65.11-27.66) | 1.817(1.479-2.155) |
| Norway | Male | 574.84(509.27-646.8) | 1366.78(1129.49-1651.35) | 137.77 | 20.48(22.97-18.08) | 32.04(38.7-26.46) | 1.554(1.314-1.795) |
| Oman | Male | 69.33(37.81-112.6) | 222.31(131.31-356.49) | 220.65 | 16.89(26.81-9.33) | 18.7(29.65-11.5) | 0.334(0.126-0.542) |
| Pakistan | Male | 5506.77(3392.63-7973) | 12225.83(8124.97-17821.19) | 122.01 | 16.48(23.93-10.13) | 18.12(25.9-12.07) | 0.304(0.125-0.483) |
| Palau | Male | 3.69(2-6.12) | 9.23(5.47-14.4) | 150.14 | 67.55(112.9-36.6) | 71.8(108.31-43.53) | 0.245(0.078-0.412) |
| Palestine | Male | 105.96(59.7-167.69) | 262.95(161.18-399.95) | 148.16 | 27(42.74-15.3) | 22.58(33.82-13.84) | -0.559(-0.899--0.217) |
| Panama | Male | 301.09(228.64-381.27) | 765.75(512.66-1113.3) | 154.33 | 38.19(48.53-28.86) | 37.83(54.89-25.4) | 0.02(-0.39-0.433) |
| Papua New Guinea | Male | 64.68(37.22-103.28) | 189.13(110.57-308.32) | 192.41 | 6.8(10.81-4.05) | 8(12.9-4.79) | 0.57(0.501-0.64) |
| Paraguay | Male | 326.91(229.75-427.65) | 764.15(482.62-1108.33) | 133.75 | 29.85(38.65-20.91) | 28.27(40.85-17.83) | -0.173(-0.458-0.113) |
| Peru | Male | 3269.4(2280.76-4447.79) | 3887.56(2423.1-5853.75) | 18.91 | 54.74(74.52-38.16) | 25.21(37.81-15.67) | -2.56(-3.52--1.591) |
| Philippines | Male | 17381.67(11854.15-23511.21) | 30985.39(22022.07-42063.04) | 78.26 | 102.63(138.27-68.32) | 74.43(100.41-53.08) | -1.089(-1.235--0.943) |
| Poland | Male | 17309.96(15416.83-19182.97) | 10752.37(8337.41-13810.42) | -37.88 | 92.57(102.47-83.05) | 36.13(46.43-28) | -3.079(-3.741--2.413) |
| Portugal | Male | 2005.81(1550.75-2463.45) | 8119.3(6099.78-10181.47) | 304.79 | 32.54(39.68-25.24) | 88.67(112.04-65.45) | 3.547(3.187-3.908) |
| Puerto Rico | Male | 1473.71(1104.79-1857.39) | 1111.62(708.74-1626.48) | -24.57 | 86.82(109.47-64.91) | 38.08(55.7-24.23) | -2.752(-3.349--2.151) |
| Qatar | Male | 36.78(20.45-59.07) | 263.72(142.34-433.58) | 617.02 | 56.27(91.91-31.71) | 41.04(67.42-22.54) | -1.093(-1.97--0.208) |
| Republic of Korea | Male | 13289.56(8007.47-20102.52) | 54683.58(37923.28-75262.21) | 311.48 | 84.27(126.44-52.34) | 127.69(174.31-89.5) | 1.459(1.093-1.825) |
| Republic of Moldova | Male | 643.48(537.39-744.77) | 1142.49(890.59-1444.26) | 77.55 | 33.6(38.84-28.27) | 47.61(59.56-37.31) | 1.108(-0.813-3.066) |
| Romania | Male | 3886.1(2892.09-5016.69) | 9096.8(6493.71-12088.62) | 134.09 | 29.86(37.91-22.49) | 59.23(79.17-42.67) | 2.434(1.838-3.034) |
| Russian Federation | Male | 19390.26(16426.06-22510.05) | 46771.24(34310.5-62862.24) | 141.21 | 28.07(32.17-24.21) | 50.65(67.83-37.31) | 2.152(1.24-3.074) |
| Rwanda | Male | 914.53(563.74-1406.38) | 1694.89(1084.37-2541.2) | 85.33 | 65.37(100.62-40.72) | 59.66(89.33-39.49) | -0.281(-0.616-0.055) |
| Saint Kitts and Nevis | Male | 25.24(18.36-33.31) | 17.37(11.92-24.52) | -31.18 | 153.46(202.64-111.2) | 49.68(68.93-34.9) | -4.003(-4.761--3.239) |
| Saint Lucia | Male | 33.94(25.63-43.11) | 34.58(24.8-45.72) | 1.89 | 84.59(107.78-63.63) | 33.25(43.86-24.12) | -3.204(-3.845--2.56) |
| Saint Vincent and the Grenadines | Male | 31.01(22.61-40.18) | 31.59(22.84-41.47) | 1.87 | 94.67(122.98-68.72) | 44.17(57.33-32.35) | -2.455(-3.179--1.727) |
| Samoa | Male | 17.48(9.88-28.28) | 23.9(14.16-36.78) | 36.73 | 37.26(59.58-21.49) | 30.21(45.43-18.22) | -0.731(-0.831--0.63) |
| San Marino | Male | 4.84(3.45-6.53) | 11.59(6.53-18.17) | 139.46 | 31.39(42.09-22.47) | 41.85(66.52-23.79) | 1.025(0.94-1.11) |
| Sao Tome and Principe | Male | 5.68(3.51-8.45) | 13.98(7.65-22.28) | 146.13 | 17.97(26.73-11.44) | 23.97(37.37-12.72) | 0.993(0.581-1.407) |
| Saudi Arabia | Male | 588.88(335.88-941.58) | 1395.77(776.08-2295.45) | 137.02 | 16.77(27.51-9.63) | 13.4(21.79-7.8) | -0.808(-1.047--0.568) |
| Senegal | Male | 147.7(91.66-220.62) | 305.17(178.03-482.93) | 106.61 | 8.64(12.9-5.4) | 8.06(12.81-4.66) | -0.167(-0.969-0.641) |
| Serbia | Male | 4769.25(3396.05-6282.92) | 5737.64(3887.59-8088.95) | 20.30 | 84.42(109.4-61.47) | 78.51(110.61-53.61) | -0.205(-0.412-0.002) |
| Seychelles | Male | 19.83(12.94-29.02) | 37.62(23.8-54.29) | 89.71 | 77.43(113.13-50.39) | 64.23(91.53-41.9) | -0.643(-0.777--0.51) |
| Sierra Leone | Male | 445.73(278.21-705.46) | 635.24(369.85-1013.29) | 42.52 | 43.62(67.96-27.23) | 32.91(51.27-19.2) | -0.955(-1.233--0.676) |
| Singapore | Male | 304.13(202.29-433.65) | 986.83(639.21-1458.35) | 224.48 | 27.28(39.1-18.29) | 25.03(37.12-16.18) | -0.348(-0.732-0.037) |
| Slovakia | Male | 2339.51(1815.85-2823) | 2554.02(1718.72-3563.13) | 9.17 | 88.78(107.67-69.35) | 62.61(86.44-42.56) | -1.25(-1.465--1.035) |
| Slovenia | Male | 518.8(356.04-710.11) | 1600.62(1059.95-2285.12) | 208.52 | 51(69.65-35.35) | 85.03(121.16-56.73) | 1.901(1.5-2.303) |
| Solomon Islands | Male | 19.44(11.14-32.6) | 36.71(22.17-56.81) | 88.84 | 23.21(38.43-13.37) | 21.69(33.08-13.11) | -0.246(-0.499-0.006) |
| Somalia | Male | 397.89(186.19-1021.66) | 888.28(431.06-1997.34) | 123.25 | 30.79(79.38-14.84) | 28.89(63.14-14.23) | -0.198(-0.328--0.068) |
| South Africa | Male | 5681.45(3078.61-12387.71) | 12955.02(10114.47-16094.04) | 128.02 | 54.72(119.01-29.22) | 60.13(73.81-47.31) | 0.389(-0.214-0.997) |
| South Sudan | Male | 358.07(144.41-905.48) | 465.44(188.82-1103.68) | 29.99 | 25.29(62.78-10.25) | 22.16(50.33-9.33) | -0.471(-0.619--0.323) |
| Spain | Male | 12910.31(9302.19-16635.36) | 24462.42(17104.32-32136.2) | 89.48 | 53.26(68.13-38.8) | 62.98(83.04-44.39) | 0.551(0.412-0.689) |
| Sri Lanka | Male | 916.97(605.7-1309.8) | 3576.92(2160.4-5542.31) | 290.08 | 16.34(23.26-10.6) | 29.68(45.64-17.96) | 2.192(1.666-2.721) |
| Sudan | Male | 861.98(435.53-1768.07) | 1906.48(937.32-3793.47) | 121.17 | 17.17(34.83-8.68) | 19.18(38.3-9.44) | 0.341(0.198-0.483) |
| Suriname | Male | 106.22(75.74-138.77) | 106.47(72.95-154.02) | 0.24 | 81.19(106.04-58.06) | 36.82(53.1-25.64) | -2.714(-3.207--2.22) |
| Sweden | Male | 2490.35(2178.93-2801.44) | 4905.98(4218.95-5567.62) | 97.00 | 39.38(44.23-34.74) | 55.59(63.54-47.99) | 1.19(0.892-1.49) |
| Switzerland | Male | 2046.72(1599.55-2442.18) | 5502.57(4087.3-6984.96) | 168.85 | 47.15(56.55-36.74) | 72.66(92.15-54.13) | 1.447(1.146-1.75) |
| Syrian Arab Republic | Male | 458.04(269.75-703.63) | 914.07(515.07-1498.37) | 99.56 | 16.17(25.31-9.49) | 13.71(21.98-7.72) | -0.589(-0.82--0.358) |
| Taiwan (Province of China) | Male | 7006.45(4330.53-10101.34) | 5824.13(3498.9-9105.52) | -16.87 | 72.99(104.78-45.75) | 31.53(48.8-18.91) | -3.172(-3.98--2.357) |
| Tajikistan | Male | 116.36(76.52-162.31) | 837.2(540.61-1228.44) | 619.49 | 9.4(13.08-6.26) | 32.56(47.16-21.12) | 4.385(4.183-4.587) |
| Thailand | Male | 45446.19(31205.74-62629.53) | 159532.42(101659.37-242984.85) | 251.04 | 241.96(333.56-164.97) | 326.9(489.5-210.42) | 0.919(0.635-1.204) |
| Timor-Leste | Male | 91.54(45.95-161.55) | 225.44(105.34-382.73) | 146.27 | 54.96(93.29-27.93) | 52.59(89.39-25.21) | -0.125(-0.331-0.081) |
| Togo | Male | 294.68(177.16-465.13) | 691.51(409.97-1077.23) | 134.66 | 45.74(71.9-27.75) | 39.5(60.68-24.34) | -0.511(-0.62--0.402) |
| Tokelau | Male | 0.21(0.11-0.36) | 0.24(0.12-0.38) | 14.29 | 35.94(60.83-17.64) | 34.23(55.38-17.9) | -0.165(-0.193--0.138) |
| Tonga | Male | 38.31(20.22-62.46) | 62.07(35-98.78) | 62.02 | 131.82(213.19-70.24) | 160.45(251.63-90.29) | 0.71(0.557-0.863) |
| Trinidad and Tobago | Male | 308.16(229.51-394.72) | 276.36(180.15-400.08) | -10.32 | 74.02(94.9-54.76) | 30.13(43.3-19.73) | -3.081(-3.517--2.643) |
| Tunisia | Male | 211.47(117.82-364.39) | 535.45(264.4-970.56) | 153.20 | 7.73(13.07-4.33) | 8.42(15.27-4.19) | 0.302(0.113-0.492) |
| Turkey | Male | 3093.16(1873.07-4731.64) | 6210.73(3798.77-9545.07) | 100.79 | 17.82(27.37-10.81) | 14.71(22.6-9.1) | -0.65(-0.874--0.425) |
| Turkmenistan | Male | 88.86(62.04-118.77) | 1591.04(1019.45-2275.91) | 1690.50 | 11.12(14.79-7.81) | 78.88(111-51.84) | 6.955(5.893-8.027) |
| Tuvalu | Male | 1.7(0.92-2.89) | 2.38(1.31-3.97) | 40.00 | 51.54(86.92-27.71) | 45.73(75.41-25.53) | -0.422(-0.529--0.315) |
| Uganda | Male | 2371.24(1531.29-3314.87) | 6381.79(4198.44-9494.79) | 169.13 | 68.53(94.88-44.61) | 87.06(123.15-58.49) | 0.821(0.629-1.014) |
| Ukraine | Male | 3239.05(2783.52-3675.32) | 12752.96(9663.37-16288.38) | 293.73 | 12.33(13.88-10.68) | 44.18(56.21-33.77) | 4.353(3.834-4.875) |
| United Arab Emirates | Male | 64.02(24.66-134.17) | 713.2(213.08-1953.76) | 1014.03 | 18.98(44.19-6.99) | 18.38(50.37-5.8) | -0.115(-0.51-0.282) |
| United Kingdom | Male | 10560.73(9362.37-11702.15) | 29601.34(26141.92-33181.7) | 180.30 | 27.67(30.59-24.58) | 53.96(60.48-47.56) | 2.312(2.121-2.504) |
| United Republic of Tanzania | Male | 1084.53(694.22-1636.29) | 2851.57(1754.2-4355.47) | 162.93 | 19.53(29.43-12.51) | 23.7(35.98-14.85) | 0.672(0.242-1.102) |
| United States of America | Male | 37211.67(33180.36-41133.14) | 156220.08(125409.09-187765.54) | 319.81 | 27.44(30.32-24.47) | 62.82(75.67-50.67) | 2.959(2.865-3.053) |
| United States Virgin Islands | Male | 20.22(13.72-27.6) | 31.26(21.68-43.92) | 54.60 | 47.19(64-32.64) | 36.84(52.18-25.72) | -0.748(-1.12--0.374) |
| Uruguay | Male | 301.72(206.42-414.67) | 698.77(498.76-907.47) | 131.60 | 17.36(23.76-11.97) | 31.82(41.52-22.8) | 2.181(1.649-2.715) |
| Uzbekistan | Male | 421.13(293.78-551.11) | 8013.61(5240.43-11169.84) | 1802.88 | 8.89(11.57-6.28) | 74.61(101.81-51.05) | 7.671(7.16-8.185) |
| Vanuatu | Male | 17.44(8.55-31.4) | 49.05(25.27-85.4) | 181.25 | 43.66(79.61-20.91) | 49.71(86.49-25.75) | 0.446(0.297-0.596) |
| Venezuela (Bolivarian Republic of) | Male | 3795.87(2901.78-4684.28) | 4582.54(3034.41-6666.37) | 20.72 | 79.49(97.38-60.45) | 32.85(47.76-21.59) | -3.268(-4.295--2.231) |
| Viet Nam | Male | 5455.14(3298.27-8685.74) | 11237.12(6888.21-17230.74) | 105.99 | 30.97(48.71-19.02) | 27.46(40.86-16.91) | -0.491(-0.655--0.327) |
| Yemen | Male | 204.88(102.17-388.09) | 586.54(325.83-1035.02) | 186.28 | 8.4(15.9-4.33) | 8.87(15.5-4.95) | 0.174(0.12-0.227) |
| Zambia | Male | 328.32(175.94-653.07) | 1023.14(653.04-1445.49) | 211.63 | 21.66(42.76-11.55) | 31.44(44.12-20.63) | 1.254(1.061-1.447) |
| Zimbabwe | Male | 1964.46(1062.9-4172.38) | 3263.99(1832.8-6018.12) | 66.15 | 86.31(181.41-46.59) | 92.64(168.09-53.33) | 0.314(-0.216-0.846) |

**Table** S9 Age distribution of death rate (per 100,000) for cirrhosis attributable to alcohol use in different region by sex in 2019.

|  | female | | | | | | male | | | | | |  |
| --- | --- | --- | --- | --- | --- | --- | --- | --- | --- | --- | --- | --- | --- |
|  |  |  |  |  |  |  |  |  |  |  |  |  |  |
| age group | 15-39 | | 40-64 | | 65+ | | 15-39 | | 40-64 | | 65+ | |  |
| Location | Death cases No. | Death rate per 100,000 | Death cases No. *103 | Death rate per 100,000 | Death cases No. *103 | Death rate per 100,000 | Death cases No. *103 | Death rate per 100,000 | Death cases No. *103 | Death rate per 100,000 | Death cases No. *103 | Death rate per 100,000 |  |
| Global | 10024.37 | 0.68 | 65461.32 | 6.25 | 72136.70 | 18.25 | 67218.32 | 4.47 | 316375.08 | 30.43 | 181599.59 | 55.53 |  |
| High SDI | 1127.47 | 0.71 | 14857.49 | 8.90 | 22024.49 | 21.74 | 2755.35 | 1.61 | 44888.10 | 26.52 | 37712.51 | 46.43 |  |
| High middle SDI | 2876.88 | 1.15 | 16627.40 | 6.83 | 15937.93 | 15.10 | 12844.72 | 4.81 | 68751.48 | 28.70 | 39989.57 | 50.08 |  |
| Middle SDI | 1831.91 | 0.40 | 13881.45 | 3.93 | 16818.26 | 15.29 | 19156.74 | 4.06 | 94571.28 | 27.09 | 53129.40 | 55.49 |  |
| Low middle SDI | 2455.82 | 0.67 | 12741.90 | 6.40 | 10986.74 | 18.73 | 23233.97 | 6.29 | 77239.30 | 39.45 | 34615.07 | 66.90 |  |
| Low SDI | 1726.98 | 0.76 | 7318.06 | 8.62 | 6328.98 | 32.40 | 9200.08 | 4.15 | 30751.16 | 36.01 | 16051.33 | 87.67 |  |
| Andean Latin America | 81.33 | 0.64 | 652.55 | 8.45 | 990.13 | 39.59 | 416.27 | 3.22 | 2733.98 | 37.30 | 2091.10 | 92.13 |  |
| Australasia | 17.66 | 0.36 | 210.08 | 4.51 | 300.65 | 11.79 | 37.37 | 0.77 | 646.92 | 14.59 | 617.18 | 27.85 |  |
| Caribbean | 91.30 | 1.00 | 525.50 | 8.04 | 518.00 | 20.83 | 289.48 | 3.21 | 2440.93 | 39.23 | 1514.13 | 71.73 |  |
| Central Asia | 497.51 | 2.65 | 2696.68 | 21.74 | 1863.65 | 61.53 | 2482.17 | 12.96 | 8199.35 | 72.44 | 2769.18 | 138.91 |  |
| Central Europe | 159.35 | 0.92 | 2376.70 | 11.86 | 2723.78 | 21.63 | 839.78 | 4.61 | 10993.85 | 55.86 | 7173.11 | 82.90 |  |
| Central Latin America | 328.60 | 0.64 | 3210.25 | 9.54 | 3609.80 | 33.31 | 3407.37 | 6.87 | 18398.47 | 61.02 | 10217.60 | 113.20 |  |
| Central Sub-Saharan Africa | 323.23 | 1.24 | 1064.10 | 10.90 | 624.30 | 31.18 | 1256.40 | 4.88 | 4634.10 | 48.75 | 1931.87 | 135.95 |  |
| East Asia | 222.57 | 0.09 | 3801.79 | 1.43 | 7120.47 | 7.25 | 5922.55 | 2.23 | 47406.81 | 17.40 | 33734.29 | 39.06 |  |
| Eastern Europe | 2217.42 | 6.52 | 9694.46 | 24.84 | 3802.07 | 17.84 | 6187.81 | 17.88 | 21971.90 | 66.50 | 6565.99 | 60.23 |  |
| Eastern Sub-Saharan Africa | 833.04 | 0.98 | 3485.09 | 12.06 | 3571.17 | 58.55 | 3760.86 | 4.62 | 14292.05 | 50.69 | 8672.38 | 163.71 |  |
| High-income Asia Pacific | 107.99 | 0.43 | 1170.88 | 3.55 | 4989.87 | 19.79 | 286.67 | 1.06 | 7061.40 | 21.01 | 8170.66 | 41.65 |  |
| High-income North America | 656.02 | 1.09 | 7808.90 | 13.12 | 7835.12 | 23.59 | 1056.63 | 1.72 | 19085.87 | 33.37 | 12656.32 | 47.11 |  |
| North Africa and Middle East | 96.23 | 0.08 | 899.87 | 1.33 | 1377.86 | 8.62 | 640.07 | 0.47 | 5230.44 | 7.00 | 3553.22 | 22.17 |  |
| Oceania | 3.11 | 0.12 | 19.41 | 1.58 | 8.82 | 4.01 | 73.57 | 2.65 | 202.61 | 15.33 | 42.10 | 18.28 |  |
| South Asia | 1634.23 | 0.43 | 9359.91 | 4.66 | 8519.57 | 14.55 | 24647.00 | 6.28 | 63292.06 | 30.88 | 27235.20 | 49.54 |  |
| Southeast Asia | 753.41 | 0.56 | 5516.75 | 5.81 | 5769.54 | 22.24 | 7322.35 | 5.34 | 37154.99 | 40.18 | 15375.70 | 76.45 |  |
| Southern Latin America | 73.89 | 0.58 | 815.73 | 8.43 | 1452.97 | 32.72 | 284.79 | 2.25 | 3975.88 | 44.06 | 3602.06 | 109.93 |  |
| Southern Sub-Saharan Africa | 165.43 | 0.98 | 554.42 | 6.17 | 496.19 | 18.69 | 737.05 | 4.39 | 2445.33 | 30.80 | 1204.06 | 70.55 |  |
| Tropical Latin America | 334.28 | 0.75 | 1759.10 | 5.27 | 1613.67 | 13.79 | 1860.84 | 4.20 | 12240.72 | 39.93 | 5480.72 | 60.65 |  |
| Western Europe | 336.50 | 0.52 | 5900.61 | 7.86 | 11799.68 | 24.14 | 939.46 | 1.41 | 17609.81 | 23.84 | 19761.53 | 51.12 |  |
| Western Sub-Saharan Africa | 1091.26 | 1.16 | 3938.54 | 11.50 | 3149.37 | 46.60 | 4769.81 | 5.59 | 16357.62 | 51.80 | 9231.20 | 147.29 |  |

**Table** S10 Age distribution of DALY rate (per 100,000) for cirrhosis attributable to alcohol use in different region by sex in 2019.

|  | female | | | | | | male | | | | | |  |
| --- | --- | --- | --- | --- | --- | --- | --- | --- | --- | --- | --- | --- | --- |
|  |  |  |  |  |  |  |  |  |  |  |  |  |  |
| age group | 15-39 | | 40-64 | | 65+ | | 15-39 | | 40-64 | | 65+ | |  |
| Location | DALYs number | DALY rate per 100,000 | DALYs number | DALY rate per 100,000 | DALYs number | DALY rate per 100,000 | DALYs number | DALY rate per 100,000 | DALYs number | DALY rate per 100,000 | DALYs number | DALY rate per 100,000 |  |
| Global | 577786.05 | 39.43 | 2318007.86 | 221.19 | 1187708.25 | 300.51 | 3814379.96 | 253.84 | 11575140.20 | 1113.40 | 3293996.37 | 1007.24 |  |
| High SDI | 64126.25 | 40.11 | 519661.18 | 311.16 | 326590.86 | 322.38 | 153192.97 | 89.37 | 1564088.89 | 924.02 | 645888.49 | 795.11 |  |
| High middle SDI | 161945.37 | 64.88 | 594524.51 | 244.08 | 263981.66 | 250.16 | 718996.02 | 269.19 | 2513005.25 | 1049.21 | 730356.23 | 914.70 |  |
| Middle SDI | 106371.66 | 22.98 | 485119.64 | 137.41 | 286424.32 | 260.35 | 1084488.20 | 229.64 | 3484299.28 | 998.11 | 963529.62 | 1006.42 |  |
| Low middle SDI | 142645.82 | 38.97 | 454455.23 | 228.42 | 197255.67 | 336.28 | 1322386.87 | 358.11 | 2883870.04 | 1472.93 | 645983.90 | 1248.54 |  |
| Low SDI | 102386.47 | 45.31 | 263015.87 | 309.97 | 112798.08 | 577.54 | 533757.13 | 240.73 | 1123581.25 | 1315.72 | 306416.91 | 1673.52 |  |
| Andean Latin America | 4789.87 | 37.53 | 22361.67 | 289.48 | 16602.16 | 663.77 | 23843.31 | 184.59 | 97135.35 | 1325.14 | 37128.72 | 1635.89 |  |
| Australasia | 1006.04 | 20.67 | 7436.46 | 159.50 | 4338.85 | 170.12 | 2075.97 | 42.81 | 22497.79 | 507.43 | 10273.64 | 463.54 |  |
| Caribbean | 5354.09 | 58.68 | 18460.59 | 282.45 | 8732.81 | 351.19 | 16441.41 | 182.59 | 87334.03 | 1403.75 | 27198.37 | 1288.41 |  |
| Central Asia | 28254.15 | 150.78 | 94338.44 | 760.48 | 33536.33 | 1107.18 | 141648.91 | 739.82 | 301655.30 | 2665.25 | 53354.85 | 2676.45 |  |
| Central Europe | 9034.21 | 51.93 | 81949.64 | 408.80 | 48308.50 | 383.61 | 46656.23 | 256.11 | 386353.27 | 1962.97 | 138993.58 | 1606.33 |  |
| Central Latin America | 18980.88 | 36.96 | 111540.55 | 331.62 | 62438.28 | 576.24 | 191424.19 | 385.73 | 673560.49 | 2233.96 | 183389.67 | 2031.78 |  |
| Central Sub-Saharan Africa | 19341.30 | 74.19 | 39112.18 | 400.54 | 11411.89 | 569.99 | 73815.48 | 286.89 | 169534.11 | 1783.37 | 37504.77 | 2639.25 |  |
| East Asia | 12917.70 | 5.16 | 133606.64 | 50.08 | 118114.32 | 120.23 | 331758.41 | 125.07 | 1754536.06 | 644.06 | 606190.33 | 701.90 |  |
| Eastern Europe | 123546.39 | 363.19 | 352832.13 | 904.00 | 73262.90 | 343.75 | 343245.70 | 991.69 | 817114.27 | 2473.22 | 133557.54 | 1225.22 |  |
| Eastern Sub-Saharan Africa | 49348.29 | 57.82 | 125010.62 | 432.62 | 63886.91 | 1047.47 | 220875.98 | 271.33 | 521483.17 | 1849.41 | 165117.35 | 3117.03 |  |
| High-income Asia Pacific | 6487.16 | 25.56 | 43342.90 | 131.53 | 62960.98 | 249.67 | 16233.92 | 59.77 | 252266.63 | 750.41 | 131664.85 | 671.10 |  |
| High-income North America | 36717.27 | 61.04 | 270879.97 | 455.24 | 127279.83 | 383.18 | 58241.22 | 94.93 | 650743.66 | 1137.76 | 227429.10 | 846.53 |  |
| North Africa and Middle East | 5811.16 | 4.70 | 30375.81 | 44.98 | 23434.85 | 146.66 | 36929.21 | 27.36 | 184496.33 | 246.97 | 63707.96 | 397.46 |  |
| Oceania | 177.00 | 6.63 | 707.40 | 57.41 | 162.95 | 73.99 | 4169.43 | 150.40 | 7761.58 | 587.22 | 826.22 | 358.79 |  |
| South Asia | 93898.79 | 24.95 | 333394.91 | 165.82 | 155267.15 | 265.13 | 1403574.76 | 357.58 | 2393269.95 | 1167.63 | 508786.79 | 925.38 |  |
| Southeast Asia | 43222.92 | 32.17 | 194100.39 | 204.55 | 98370.94 | 379.21 | 411536.40 | 299.91 | 1377522.02 | 1489.68 | 283493.94 | 1409.59 |  |
| Southern Latin America | 4423.95 | 34.69 | 27857.97 | 287.99 | 23175.01 | 521.83 | 15945.91 | 125.76 | 138938.15 | 1539.76 | 64610.32 | 1971.73 |  |
| Southern Sub-Saharan Africa | 9686.76 | 57.40 | 19880.16 | 221.40 | 8313.08 | 313.19 | 42035.41 | 250.14 | 89405.43 | 1126.23 | 22682.12 | 1329.02 |  |
| Tropical Latin America | 19475.40 | 43.46 | 63048.21 | 188.81 | 26290.80 | 224.68 | 103864.05 | 234.57 | 446289.50 | 1455.76 | 101223.74 | 1120.21 |  |
| Western Europe | 19544.27 | 30.39 | 206345.42 | 274.91 | 167984.80 | 343.66 | 52813.82 | 79.14 | 614714.20 | 832.36 | 325162.78 | 841.23 |  |
| Western Sub-Saharan Africa | 65768.45 | 70.16 | 141425.83 | 413.12 | 53834.91 | 796.57 | 277250.24 | 324.64 | 588528.92 | 1863.83 | 171699.71 | 2739.63 |  |

**Table** S11 Age distribution of death rate (per 100,000) for liver cancer attributable to alcohol use in different region by sex in 2019.

|  | female | | | | | | male | | | | | |  |
| --- | --- | --- | --- | --- | --- | --- | --- | --- | --- | --- | --- | --- | --- |
|  |  |  |  |  |  |  |  |  |  |  |  |  |  |
| age group | 15-39 | | 40-64 | | 65+ | | 15-39 | | 40-64 | | 65+ | |  |
| Location | Death cases No. | Death rate per 100,000 | Death cases No. *103 | Death rate per 100,000 | Death cases No. *103 | Death rate per 100,000 | Death cases No. *103 | Death rate per 100,000 | Death cases No. *103 | Death rate per 100,000 | Death cases No. *103 | Death rate per 100,000 |  |
| Global | 301.1483 | 0.0205531 | 6087.86 | 0.580909 | 12842.8 | 3.249466 | 1819.085 | 0.1210591 | 31017.47 | 2.98354 | 43984.28 | 13.44959 |  |
| High SDI | 33.44505 | 0.0209175 | 933.2731 | 0.558821 | 3635.521 | 3.588617 | 163.6726 | 0.0954831 | 7268.529 | 4.294044 | 15937.53 | 19.61962 |  |
| High middle SDI | 48.8642 | 0.0195755 | 1263.592 | 0.518756 | 2922.758 | 2.769739 | 410.6625 | 0.1537488 | 6200.963 | 2.588977 | 8668.042 | 10.85586 |  |
| Middle SDI | 93.01669 | 0.0200973 | 2415.127 | 0.684107 | 4122.441 | 3.747173 | 895.4071 | 0.1896006 | 12226.01 | 3.502271 | 12674.1 | 13.23821 |  |
| Low middle SDI | 81.33551 | 0.0222213 | 1094.732 | 0.55025 | 1697.295 | 2.893574 | 247.3357 | 0.06698 | 3901.812 | 1.992836 | 4900.937 | 9.472378 |  |
| Low SDI | 44.30942 | 0.0196068 | 377.949 | 0.44542 | 457.9886 | 2.344944 | 101.3587 | 0.0457146 | 1405.125 | 1.645413 | 1777.054 | 9.705507 |  |
| Andean Latin America | 2.9708 | 0.023278 | 63.97813 | 0.828232 | 183.7239 | 7.345479 | 6.740628 | 0.052185 | 91.29584 | 1.245473 | 245.4654 | 10.81521 |  |
| Australasia | 1.530428 | 0.03144 | 34.09936 | 0.731364 | 98.1522 | 3.848461 | 6.203227 | 0.12791 | 214.9432 | 4.84796 | 410.5538 | 18.52405 |  |
| Caribbean | 2.823265 | 0.030944 | 46.25523 | 0.707718 | 94.60656 | 3.804565 | 6.787112 | 0.075374 | 147.517 | 2.371098 | 296.7318 | 14.05642 |  |
| Central Asia | 10.60859 | 0.056612 | 211.247 | 1.702912 | 266.6405 | 8.802993 | 29.14411 | 0.152218 | 745.9372 | 6.590665 | 600.6355 | 30.12985 |  |
| Central Europe | 4.485311 | 0.025784 | 160.7006 | 0.801645 | 548.7412 | 4.357514 | 14.59793 | 0.080133 | 739.4403 | 3.756917 | 1624.731 | 18.77682 |  |
| Central Latin America | 10.57003 | 0.020581 | 234.6539 | 0.69764 | 446.8114 | 4.123598 | 27.59381 | 0.055603 | 626.5896 | 2.078177 | 1241.476 | 13.75438 |  |
| Central Sub-Saharan Africa | 3.540473 | 0.01358 | 27.16187 | 0.278162 | 19.1851 | 0.958245 | 5.762722 | 0.022398 | 68.96585 | 0.725467 | 60.18445 | 4.235246 |  |
| East Asia | 56.16809 | 0.022428 | 1909.652 | 0.715833 | 3097.638 | 3.153028 | 946.3272 | 0.356755 | 9309.298 | 3.417304 | 7321.003 | 8.476864 |  |
| Eastern Europe | 12.89008 | 0.037893 | 286.2888 | 0.733506 | 713.2567 | 3.346653 | 45.85723 | 0.132488 | 1111.507 | 3.364278 | 1472.88 | 13.51178 |  |
| Eastern Sub-Saharan Africa | 17.24557 | 0.020205 | 147.8403 | 0.511626 | 175.4144 | 2.876044 | 33.36549 | 0.040986 | 448.2008 | 1.58952 | 559.579 | 10.56356 |  |
| High-income Asia Pacific | 6.45098 | 0.025414 | 182.6891 | 0.5544 | 1135.408 | 4.502451 | 31.27483 | 0.115155 | 1447.154 | 4.304822 | 3934.375 | 20.05353 |  |
| High-income North America | 11.26878 | 0.018733 | 341.06 | 0.573179 | 900.7597 | 2.711768 | 55.78187 | 0.090918 | 3123.631 | 5.461347 | 4442.249 | 16.53481 |  |
| North Africa and Middle East | 9.653571 | 0.007806 | 165.7076 | 0.245356 | 219.3174 | 1.372492 | 40.95018 | 0.030344 | 1026.005 | 1.373424 | 1110.406 | 6.927567 |  |
| Oceania | 0.23152 | 0.008672 | 2.815214 | 0.228467 | 2.413104 | 1.095684 | 0.685468 | 0.024725 | 11.45808 | 0.866884 | 12.58662 | 5.465731 |  |
| South Asia | 78.01476 | 0.020729 | 815.3944 | 0.405545 | 1336.636 | 2.282389 | 186.9584 | 0.047631 | 3222.582 | 1.572233 | 4437.518 | 8.070973 |  |
| Southeast Asia | 25.97845 | 0.019333 | 645.9345 | 0.680716 | 1175.984 | 4.533255 | 197.802 | 0.144151 | 4139.71 | 4.476776 | 5038.137 | 25.05064 |  |
| Southern Latin America | 1.417587 | 0.011116 | 30.6982 | 0.317348 | 94.95211 | 2.138026 | 4.324102 | 0.034103 | 146.9089 | 1.628098 | 373.9179 | 11.41098 |  |
| Southern Sub-Saharan Africa | 7.662239 | 0.0454 | 66.6094 | 0.741816 | 90.16399 | 3.396895 | 47.28757 | 0.281392 | 299.5239 | 3.773078 | 291.0004 | 17.05064 |  |
| Tropical Latin America | 8.631989 | 0.019261 | 158.2798 | 0.473989 | 270.0225 | 2.307557 | 20.40623 | 0.046087 | 508.3043 | 1.65805 | 856.6364 | 9.480133 |  |
| Western Europe | 14.71833 | 0.022888 | 396.0669 | 0.527677 | 1775.753 | 3.632778 | 65.64371 | 0.098365 | 2979.141 | 4.033917 | 8826.383 | 22.83469 |  |
| Western Sub-Saharan Africa | 14.2875 | 0.015241 | 160.7285 | 0.469507 | 197.2166 | 2.918136 | 45.59167 | 0.053384 | 609.3562 | 1.929793 | 827.8322 | 13.20885 |  |

**Table** S12 Age distribution of DALY rate (per 100,000) for liver cancer attributable to alcohol use in different region by sex in 2019.

|  | female | | | | | | male | | | | | |  |
| --- | --- | --- | --- | --- | --- | --- | --- | --- | --- | --- | --- | --- | --- |
|  |  |  |  |  |  |  |  |  |  |  |  |  |  |
| age group | 15-39 | | 40-64 | | 65+ | | 15-39 | | 40-64 | | 65+ | |  |
| Location | DALYs number | DALY rate per 100,000 | DALYs number | DALY rate per 100,000 | DALYs number | DALY rate per 100,000 | DALYs number | DALY rate per 100,000 | DALYs number | DALY rate per 100,000 | DALYs number | DALY rate per 100,000 |  |
| Global | 16683.48 | 1.14 | 204626.78 | 19.53 | 217286.96 | 54.98 | 100236.79 | 6.67 | 1058450.94 | 101.81 | 781787.45 | 239.06 |  |
| High SDI | 1865.85 | 1.17 | 30620.83 | 18.33 | 56037.55 | 55.31 | 9056.94 | 5.28 | 236899.52 | 139.95 | 272415.15 | 335.35 |  |
| High middle SDI | 2683.62 | 1.08 | 42101.02 | 17.28 | 49105.20 | 46.53 | 22556.22 | 8.44 | 212772.62 | 88.84 | 153919.25 | 192.77 |  |
| Middle SDI | 5122.24 | 1.11 | 81107.14 | 22.97 | 73352.41 | 66.68 | 49221.23 | 10.42 | 425431.06 | 121.87 | 232427.97 | 242.77 |  |
| Low middle SDI | 4528.08 | 1.24 | 37431.35 | 18.81 | 30309.57 | 51.67 | 13707.76 | 3.71 | 134394.58 | 68.64 | 89567.10 | 173.11 |  |
| Low SDI | 2473.84 | 1.09 | 13258.82 | 15.63 | 8369.48 | 42.85 | 5658.74 | 2.55 | 48450.87 | 56.74 | 32990.93 | 180.18 |  |
| Andean Latin America | 165.99 | 1.30 | 2121.52 | 27.46 | 3019.79 | 120.73 | 384.27 | 2.97 | 3023.23 | 41.24 | 4124.27 | 181.72 |  |
| Australasia | 85.35 | 1.75 | 1141.04 | 24.47 | 1580.06 | 61.95 | 343.06 | 7.07 | 7110.33 | 160.37 | 7027.63 | 317.08 |  |
| Caribbean | 156.75 | 1.72 | 1571.24 | 24.04 | 1577.57 | 63.44 | 377.27 | 4.19 | 4916.89 | 79.03 | 5160.67 | 244.47 |  |
| Central Asia | 587.28 | 3.13 | 7046.54 | 56.80 | 4831.87 | 159.52 | 1611.23 | 8.42 | 25269.10 | 223.26 | 11247.50 | 564.21 |  |
| Central Europe | 246.71 | 1.42 | 5244.76 | 26.16 | 9048.60 | 71.85 | 804.70 | 4.42 | 23873.67 | 121.30 | 28926.20 | 334.30 |  |
| Central Latin America | 586.62 | 1.14 | 7850.31 | 23.34 | 7706.89 | 71.13 | 1547.14 | 3.12 | 20665.23 | 68.54 | 21760.07 | 241.08 |  |
| Central Sub-Saharan Africa | 198.73 | 0.76 | 968.55 | 9.92 | 354.27 | 17.69 | 335.21 | 1.30 | 2371.45 | 24.95 | 1151.53 | 81.03 |  |
| East Asia | 3061.74 | 1.22 | 64346.34 | 24.12 | 56277.43 | 57.28 | 52005.44 | 19.61 | 334800.49 | 122.90 | 136196.09 | 157.70 |  |
| Eastern Europe | 708.10 | 2.08 | 9388.71 | 24.06 | 11890.03 | 55.79 | 2507.25 | 7.24 | 36916.95 | 111.74 | 26851.65 | 246.33 |  |
| Eastern Sub-Saharan Africa | 960.80 | 1.13 | 5190.15 | 17.96 | 3192.76 | 52.35 | 1875.36 | 2.30 | 15386.14 | 54.57 | 10482.34 | 197.88 |  |
| High-income Asia Pacific | 359.83 | 1.42 | 6041.67 | 18.33 | 16348.95 | 64.83 | 1709.19 | 6.29 | 48484.11 | 144.22 | 64597.13 | 329.25 |  |
| High-income North America | 630.76 | 1.05 | 11104.60 | 18.66 | 14860.26 | 44.74 | 3094.05 | 5.04 | 100853.87 | 176.33 | 79283.95 | 295.11 |  |
| North Africa and Middle East | 534.54 | 0.43 | 5619.02 | 8.32 | 3975.03 | 24.88 | 2251.79 | 1.67 | 34540.61 | 46.24 | 20646.55 | 128.81 |  |
| Oceania | 12.80 | 0.48 | 96.62 | 7.84 | 44.13 | 20.04 | 38.21 | 1.38 | 391.63 | 29.63 | 239.92 | 104.19 |  |
| South Asia | 4352.70 | 1.16 | 28039.98 | 13.95 | 23811.37 | 40.66 | 10281.52 | 2.62 | 109737.40 | 53.54 | 81623.76 | 148.46 |  |
| Southeast Asia | 1439.48 | 1.07 | 21601.55 | 22.76 | 20494.83 | 79.00 | 10866.77 | 7.92 | 139923.55 | 151.32 | 91775.44 | 456.33 |  |
| Southern Latin America | 78.74 | 0.62 | 1020.23 | 10.55 | 1570.59 | 35.36 | 241.70 | 1.91 | 4770.19 | 52.86 | 6550.46 | 199.90 |  |
| Southern Sub-Saharan Africa | 424.85 | 2.52 | 2303.29 | 25.65 | 1603.31 | 60.40 | 2619.52 | 15.59 | 10532.93 | 132.68 | 5411.74 | 317.09 |  |
| Tropical Latin America | 477.39 | 1.07 | 5341.87 | 16.00 | 4595.35 | 39.27 | 1131.68 | 2.56 | 16877.18 | 55.05 | 15277.85 | 169.08 |  |
| Western Europe | 821.08 | 1.28 | 13047.29 | 17.38 | 26871.76 | 54.97 | 3666.22 | 5.49 | 96805.99 | 131.08 | 148558.34 | 384.33 |  |
| Western Sub-Saharan Africa | 793.24 | 0.85 | 5541.49 | 16.19 | 3632.12 | 53.74 | 2545.20 | 2.98 | 21200.01 | 67.14 | 14894.35 | 237.65 |  |

**Table S13** Prediction the burden of cirrhosis and liver cancer attributable to alcohol use.

|  | Prediction of cirrhosis death cases attributable to alcohol use | | | Prediction of liver cancer death cases attributable to alcohol use | | |
| --- | --- | --- | --- | --- | --- | --- |
| year | Female cases | Male cases | Both cases | Female cases | Male cases | Both cases |
| 2020 | 148231 | 578612 | 725117 | 19622 | 79201 | 98372 |
| 2021 | 150747 | 590395 | 739455 | 20220 | 81697 | 101481 |
| 2022 | 152843 | 602251 | 753451 | 20634 | 84131 | 104538 |
| 2023 | 154153 | 613836 | 766234 | 20937 | 86521 | 107556 |
| 2024 | 154973 | 625152 | 778156 | 21242 | 88956 | 110610 |
| 2025 | 155705 | 636589 | 790132 | 21595 | 91523 | 113781 |
| 2026 | 157748 | 648831 | 804511 | 22155 | 94404 | 117428 |
| 2027 | 160066 | 661617 | 819825 | 22778 | 97433 | 121246 |
| 2028 | 162515 | 674879 | 835772 | 23459 | 100631 | 125258 |
| 2029 | 164950 | 688539 | 852084 | 24189 | 104032 | 129496 |
| 2030 | 167370 | 702693 | 868866 | 24961 | 107694 | 134020 |
| 2031 | 171052 | 717714 | 888104 | 25946 | 111844 | 139262 |
| 2032 | 174974 | 733444 | 908434 | 26995 | 116324 | 144912 |
| 2033 | 179001 | 749800 | 929563 | 28108 | 121170 | 151008 |
| 2034 | 183013 | 766700 | 951256 | 29281 | 126434 | 157605 |
| 2035 | 187069 | 784281 | 973725 | 30514 | 132211 | 164806 |
| 2036 | 192501 | 802923 | 999102 | 32022 | 138854 | 173228 |
| 2037 | 198267 | 822522 | 1026032 | 33637 | 146206 | 182535 |
| 2038 | 204269 | 843045 | 1054331 | 35369 | 154364 | 192843 |
| 2039 | 210394 | 864426 | 1083771 | 37219 | 163438 | 204278 |
| 2040 | 216729 | 886828 | 1114617 | 39197 | 173610 | 217050 |
| 2041 | 224926 | 910807 | 1149601 | 41611 | 185553 | 232251 |
| 2042 | 233746 | 936200 | 1187110 | 44246 | 199118 | 249500 |
| 2043 | 243053 | 962998 | 1226927 | 47124 | 214588 | 269140 |
| 2044 | 252757 | 991183 | 1268903 | 50270 | 232303 | 291586 |

**Figure S1** The rank of ASR burden for cirrhosis and liver cancer attributable to alcohol use over the past 30 years. **a**: ASDR in cirrhosis; b: age-standardized DALY rate in cirrhosis; **c**: ASDR in liver cancer; **d**: age-standardized DALY rate in liver cancer.

ASR: age-standardized rate; ASDR: age-standardized death rate.


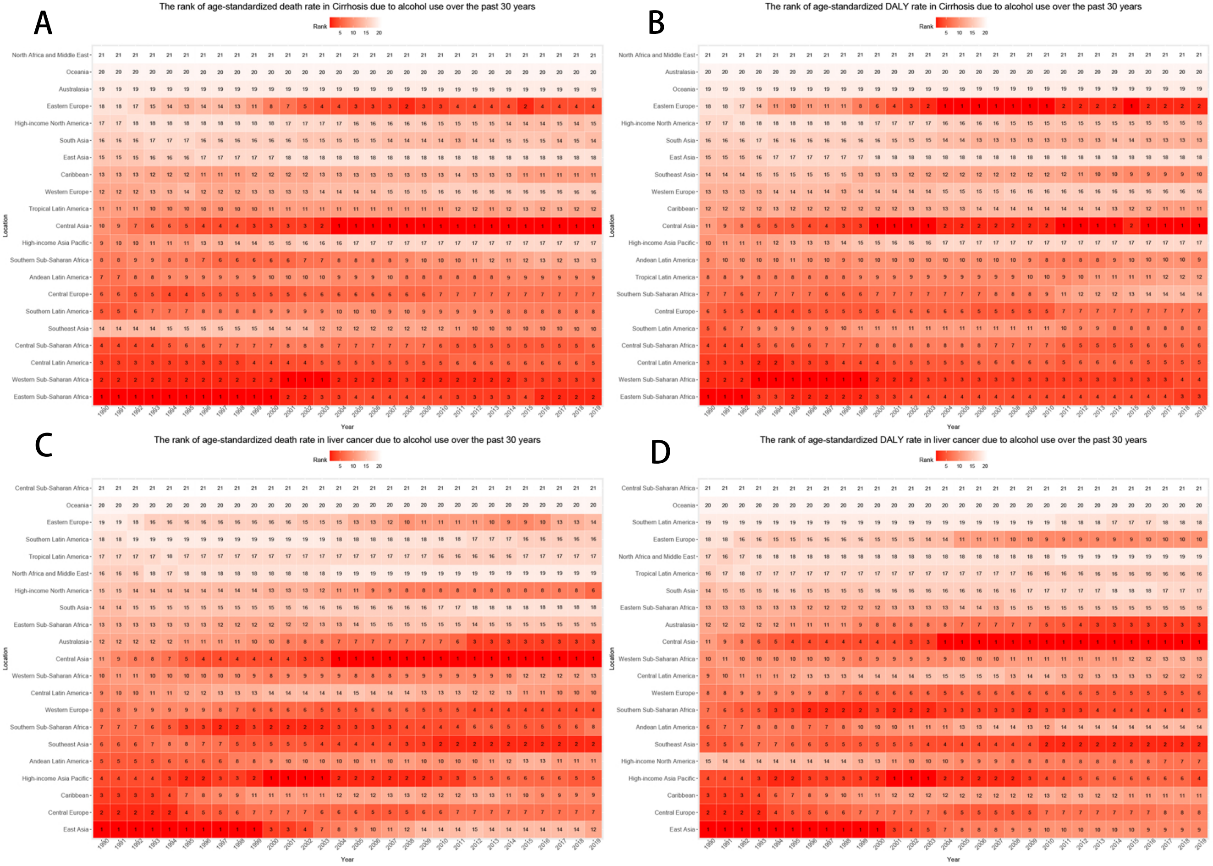


**Figure S2** The proportion of deaths or DALYs of cirrhosis(A, b) and liver cancer(C, D) attributable to alcohol use in different age groups across global, SDI regions, and 21 GBD regions.

GBD: Global Burden of Diseases, Injuries, and Risk Factors Study; SDI: socio-demographic index


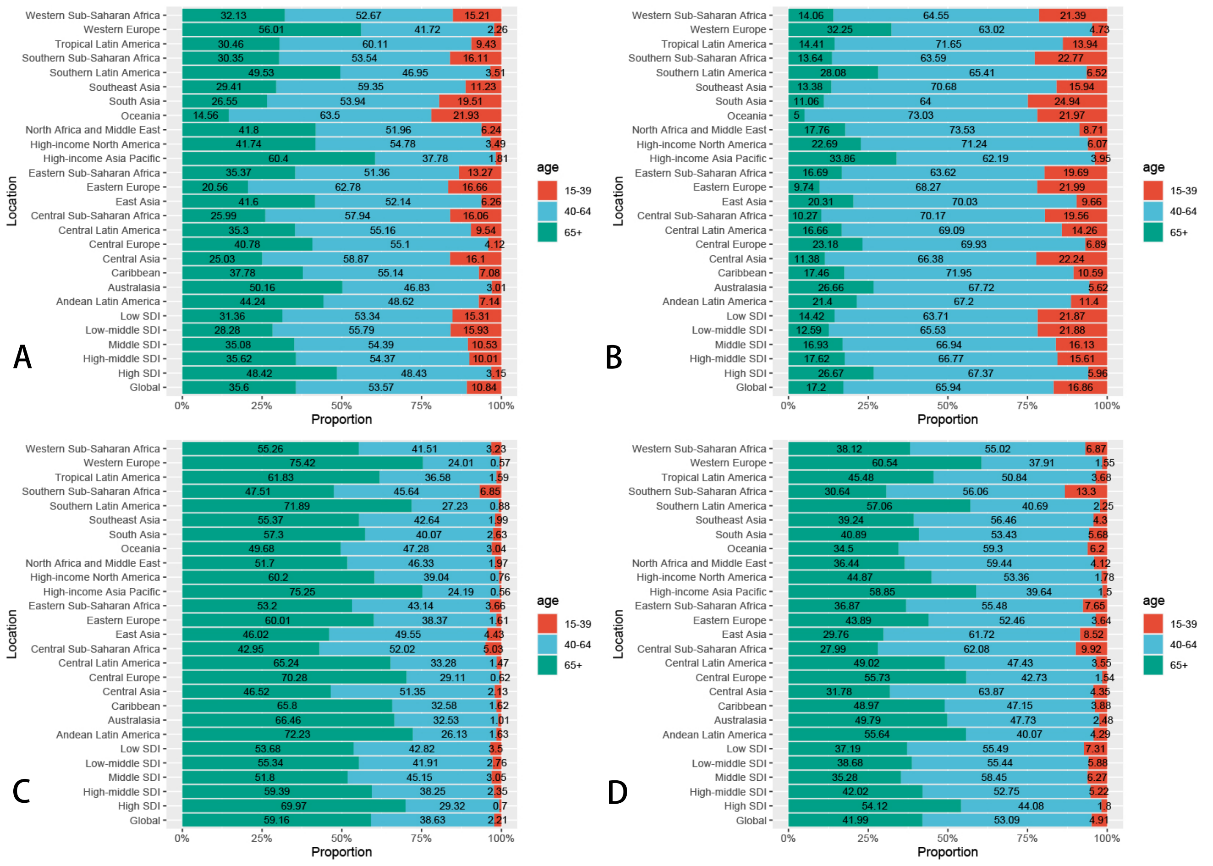


**Figure S3** The percentage change in death and DALY rate of cirrhosis(A,B) and liver cancer(C,D) attributable to alcohol use in different age groups for global, SDI and 21 GBD regions.

GBD: Global Burden of Diseases, Injuries, and Risk Factors Study; SDI: socio-demographic index


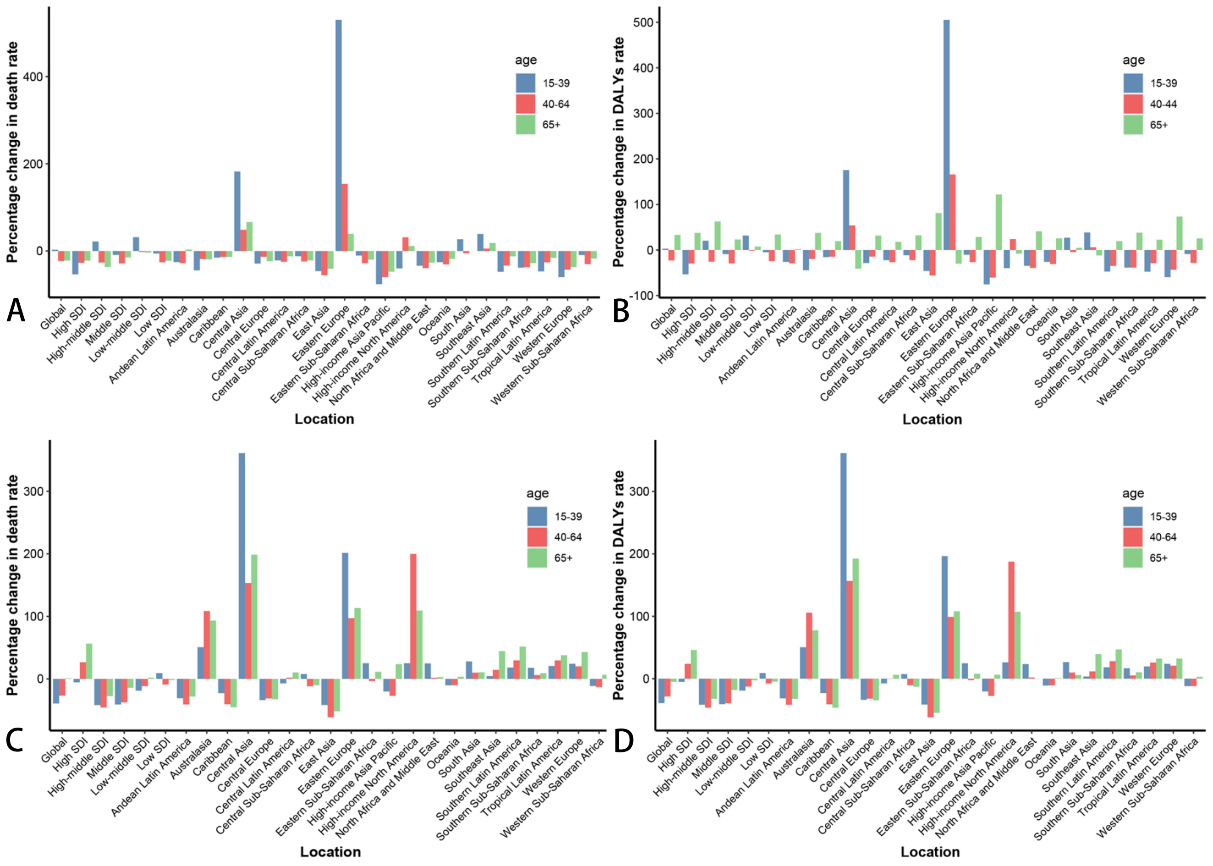


**Figure S4 The** predicted trend of age-standardized death rate of cirrhosis and liver cancer attributable to alcohol use by sex until 2044. **A** : male’s age-standardized death rate of cirrhosis attributable to alcohol use; **B** : female’s age-standardized death rate of cirrhosis attributable to alcohol use; **C** : male’s age-standardized death rate of liver cancer attributable to alcohol use; **D** : female’s age-standardized death rate of liver cancer attributable to alcohol use


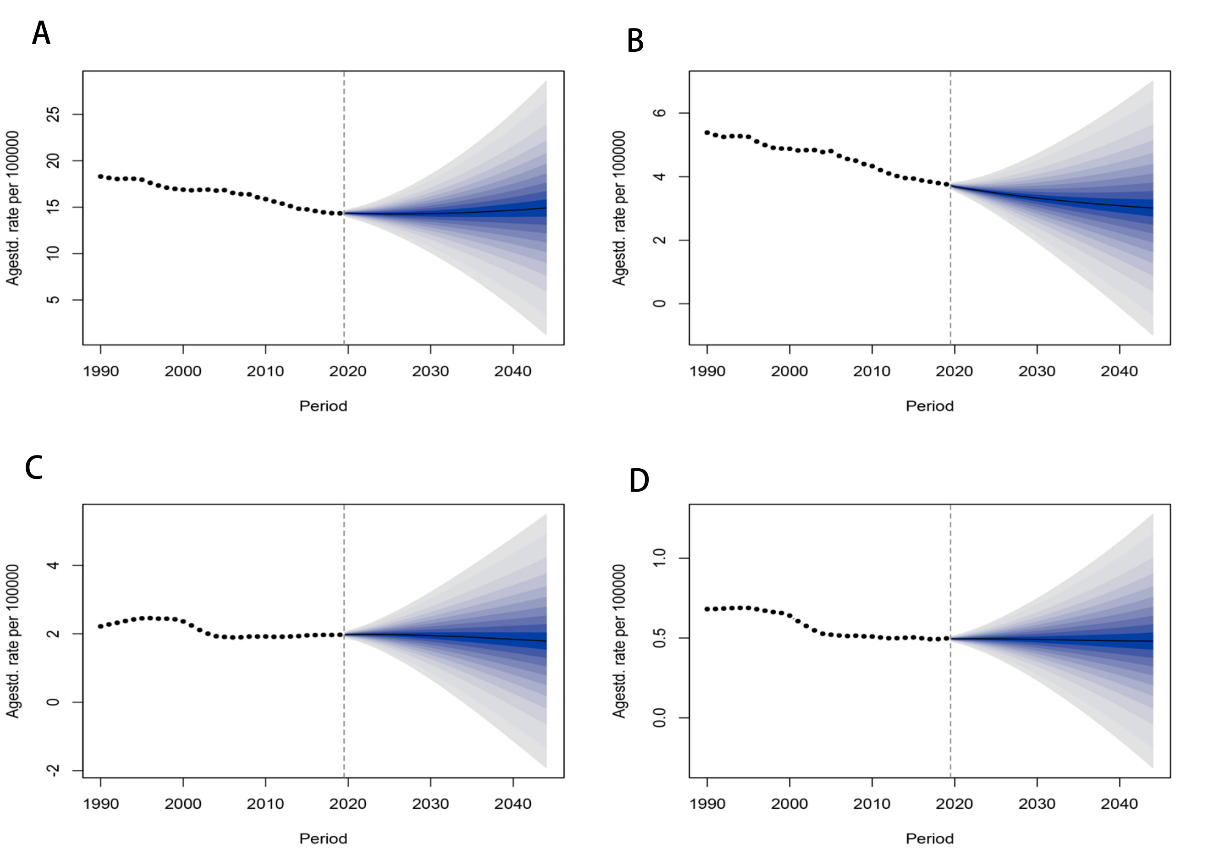


**Figure S5** The trend of cirrhosis and liver cancer burden attributable to alcohol use cross 21 GBD regions, 1990-2019. **A**: The trend of ASDR of cirrhosis; **B**: The trend of ASDR of liver cancer; **C**: The trend of age-standardized DALY rate in cirrhosis; **D**:The trend of age-standardized DALY rate in liver cancer. For each region, points from left to right depict estimates from each year from 1990 to 2019.

GBD: Global Burden of Diseases, Injuries, and Risk Factors Study; ASDR: age-standardized death rate.


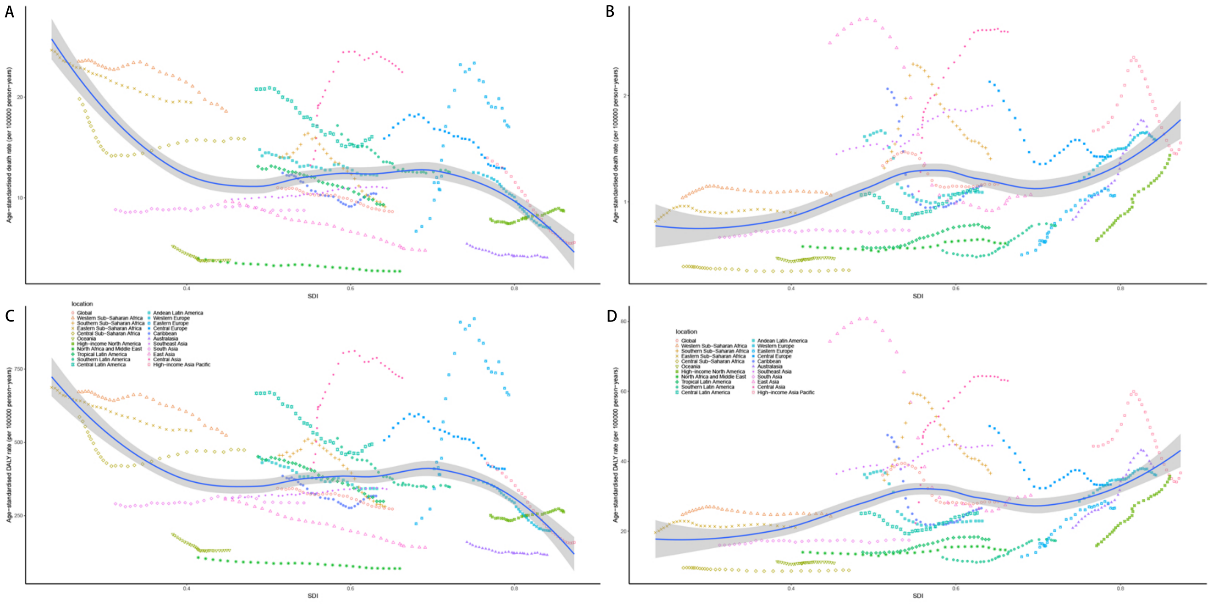

Supplement: Supplementary file 1 — Supplementary file1 (DOCX 2846 KB) [file 12072_2023_10503_MOESM1_ESM.docx]
